# Supplementary material for: Total synthesis of mycobacterial arabinogalactan containing 92 monosaccharide units
Source: Nat Commun. 2017 Mar 16;8:14851. doi: 10.1038/ncomms14851 (PMC5357306; doi:10.1038/ncomms14851)
Supplement: Supplementary Information — Supplementary figures, supplementary tables, supplementary methods and supplementary references. [file ncomms14851-s1.pdf]

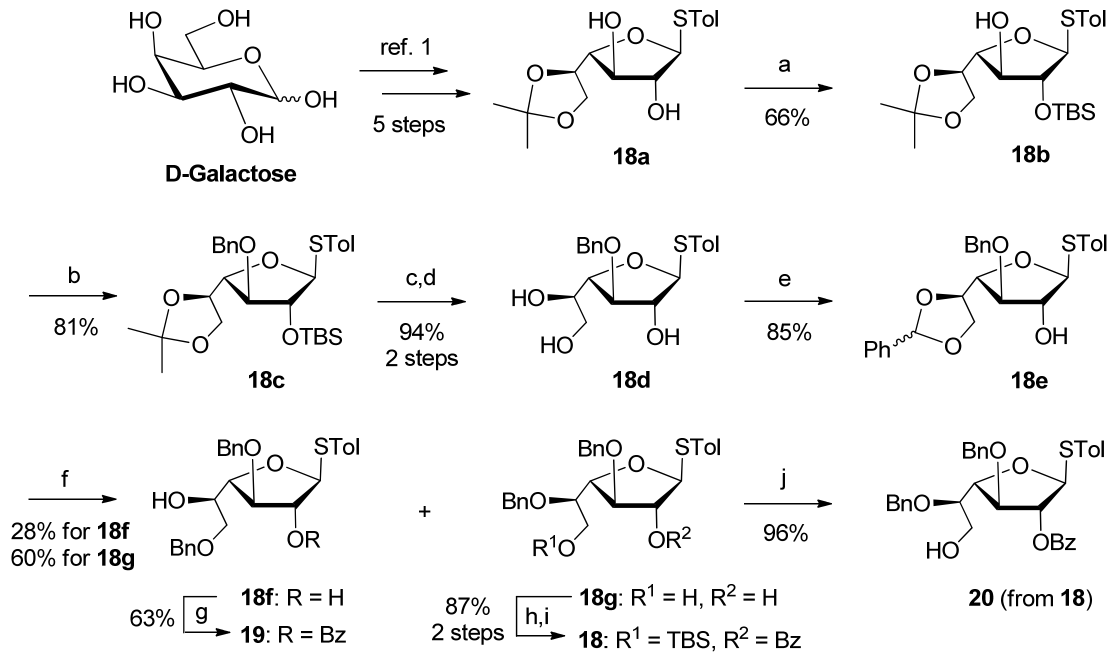

Supplementary Figure 1. Synthesis of galactofuranosyl building blocks 18–20. Regents and conditions: (a) TBSCl, imidazole, CH_2Cl_2, 0 °C, 66%; (b) BnBr, NaH, DMF, 0 °C to r.t., 81%; (c) TBAF, THF; (d) AcOH/H_2O (7:3), 50 °C, 94% over two steps; (e) $PhCH(OMe)_2$, PPTS, DMF, 60 °C, 85%; (f) DIBAL-H, CH_2Cl_2/Toluene (1:1), -40 °C, 88% (**18f** : **18g** = 1 : 2.1); (g) Bz_2O, DMAP, pyridine, 63%; (h) TBSCl, imidazole, CH_2Cl_2, 0 °C; (i) BzCl, DMAP, pyridine, 87% over two steps; (j) HF-pyridine, THF/H_2O (10:1), 35 °C, 96%.

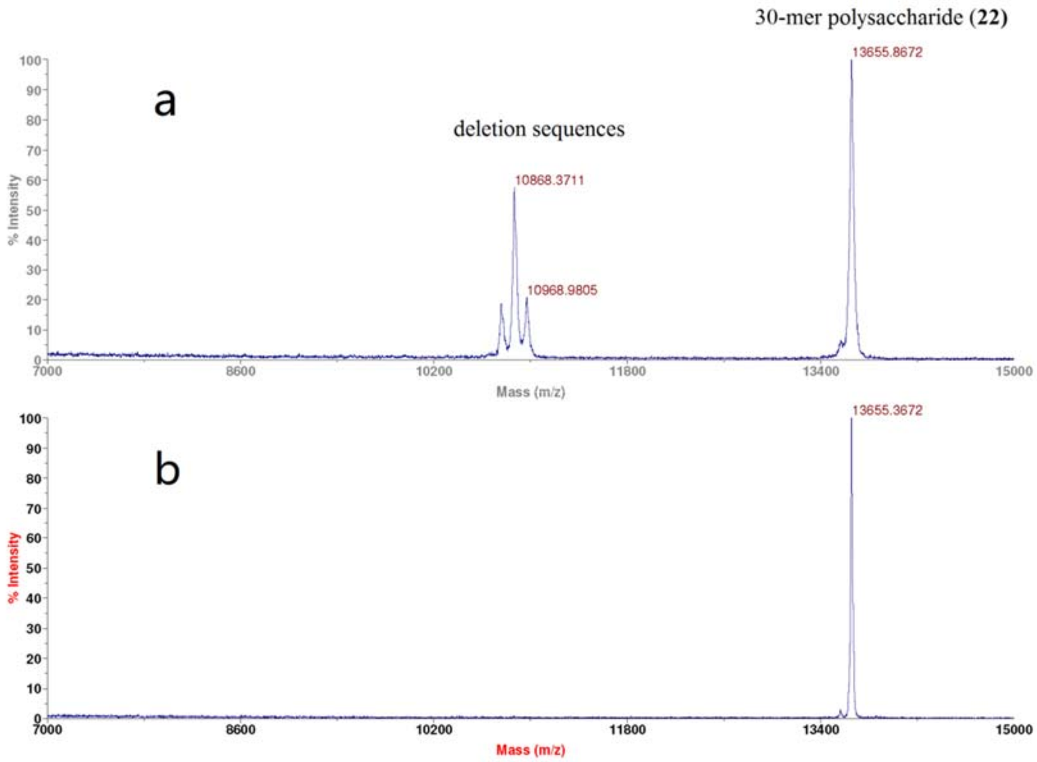

Supplementary Figure 2. MALDI-TOF mass spectra of 30-mer polysaccharide (22) before and after purification by size exclusion chromatography. **a**, Some deletion sequences were difficult to be removed by column chromatography on silica gel; **b**, All deletion sequences were removed by size exclusion chromatography to obtain the pure 30-mer polysaccharide (**22**).

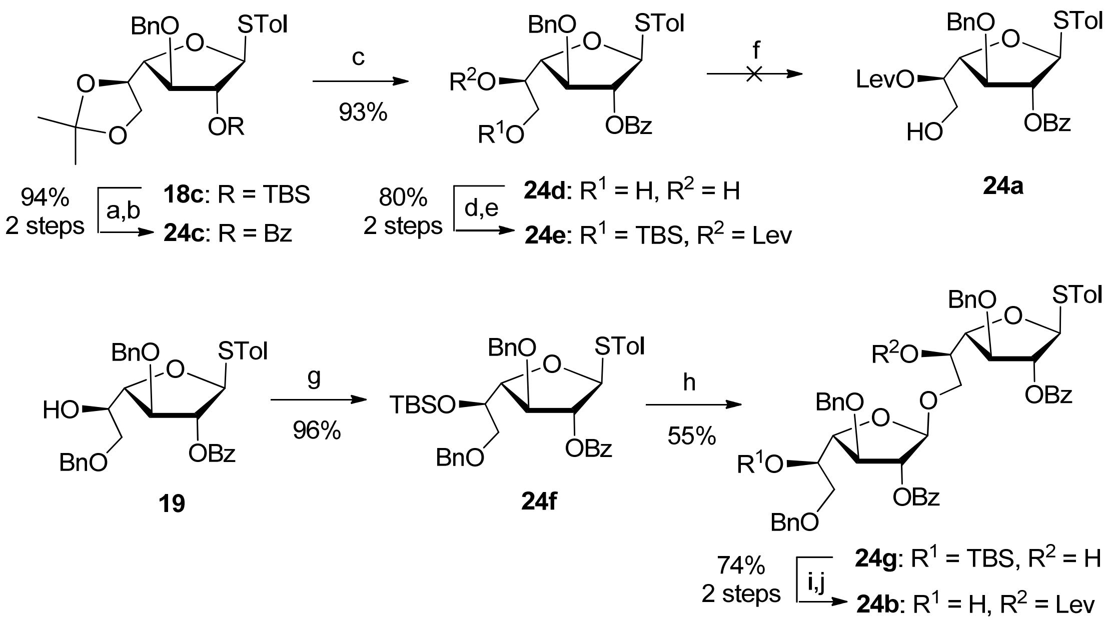

Supplementary Figure 3. Synthesis of galactofuranosyl building block 24b. Regents and conditions: (a) TBAF, THF; (b) BzCl, DMAP, pyridine, 94% over two steps; (c) AcOH/H_2O (7:3), 50 °C, 93%; (d) TBSCl, imidazole, CH_2Cl_2, 0 °C; (e) Levulinoyl acid, EDC·HCl, DMAP, CH_2Cl_2, 80% over two steps; (f) HF-pyridine, THF/H_2O (10:1), 35 °C; (g) TBSCl, imidazole, CH_2Cl_2, reflux, 96%; (h) *p*-TolSCl, AgOTf, TTBP, then **24d**, 4 Å MS, CH_2Cl_2, -78 °C, 55%; (i) Levulinoyl acid, EDC·HCl, DMAP, CH_2Cl_2, 35 °C; (j) HF-pyridine, THF/H_2O (10:1), 35 °C, 74% over two steps.

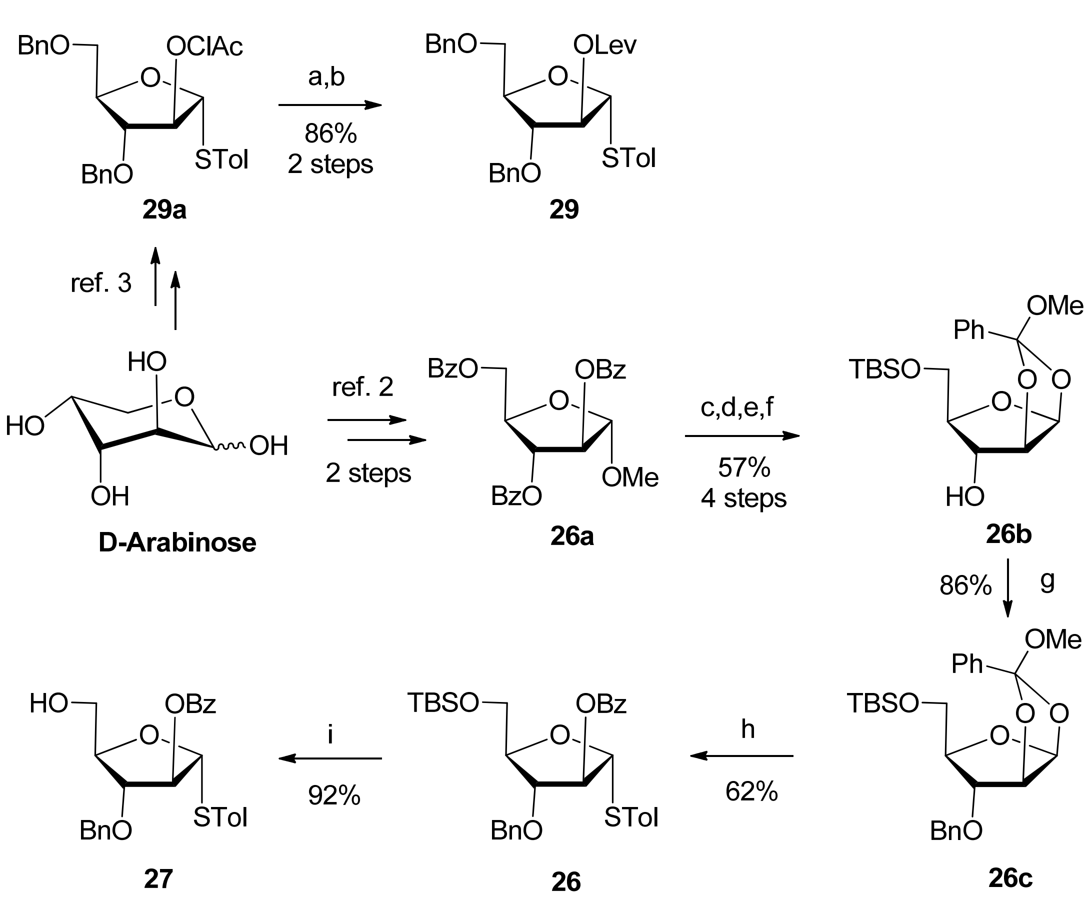

Supplementary Figure 4. Synthesis of arabinofuranosyl building blocks 26, 27 and 29. Regents and conditions: (a) CH_3ONa, CH_3OH; (b) Levulinoyl acid, EDC·HCl, DMAP, CH_2Cl_2, 86% for two steps; (c) AcBr, CH_3OH, CH_2Cl_2, 0 °C to r.t.; (d) 2,6-Lutidine, CH_3OH, 0 °C to r.t.; (e) CH_3ONa, CH_3OH; (f) TBSCl, DMAP, Et_3N, pyridine, 57% for four steps; (g) BnBr, NaH, DMF, 0 °C, 86%; (h) $SnCl_4$, *p*-TolSH, 4 Å MS, CH_2Cl_2, 0 °C, 62%; (i) TBAF, AcOH, THF, 35 °C, 92%.

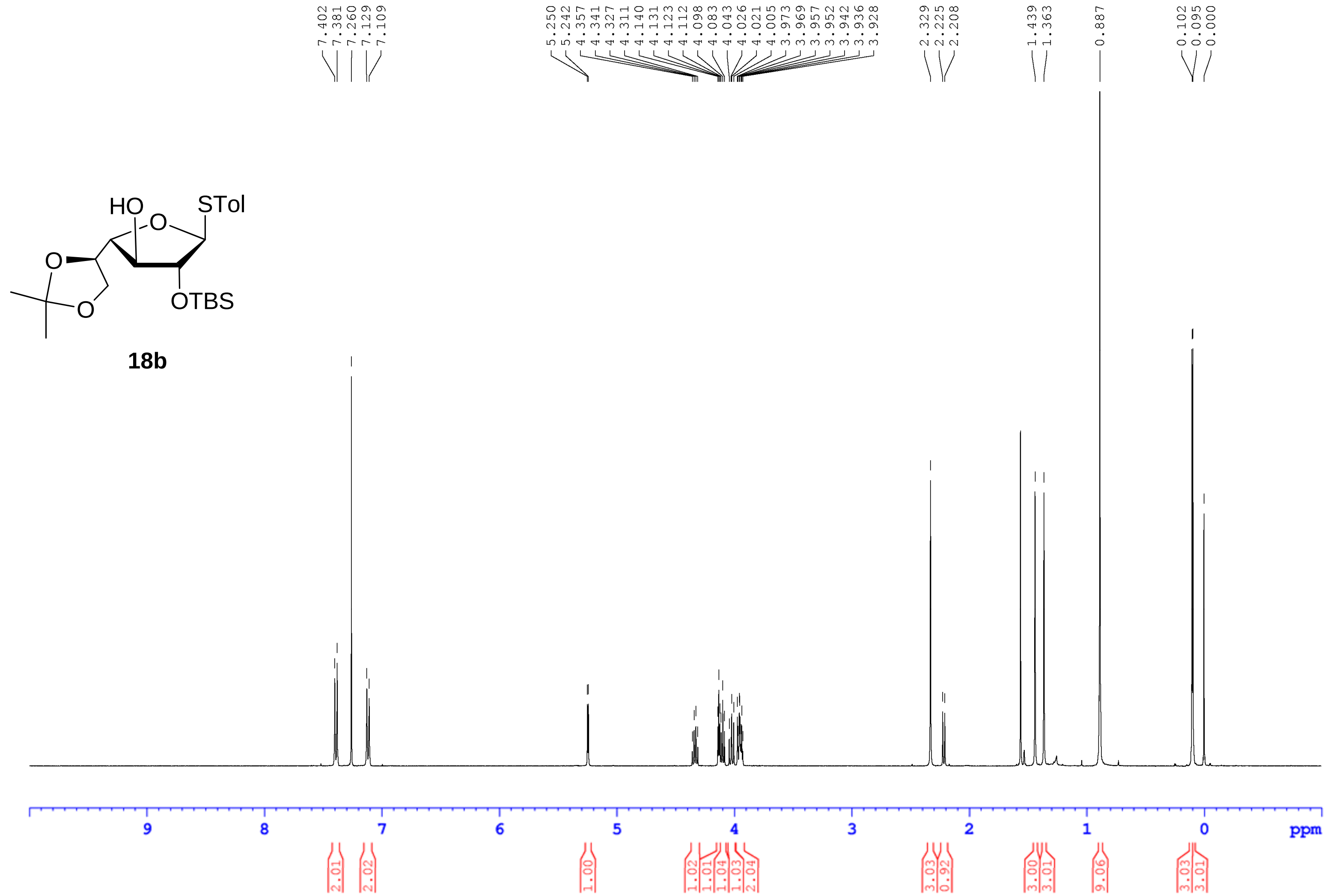

Supplementary Figure 5. ^{1}H NMR spectrum of compound 18b

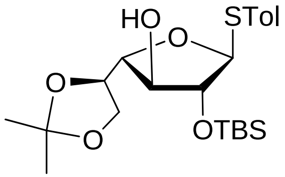

18b

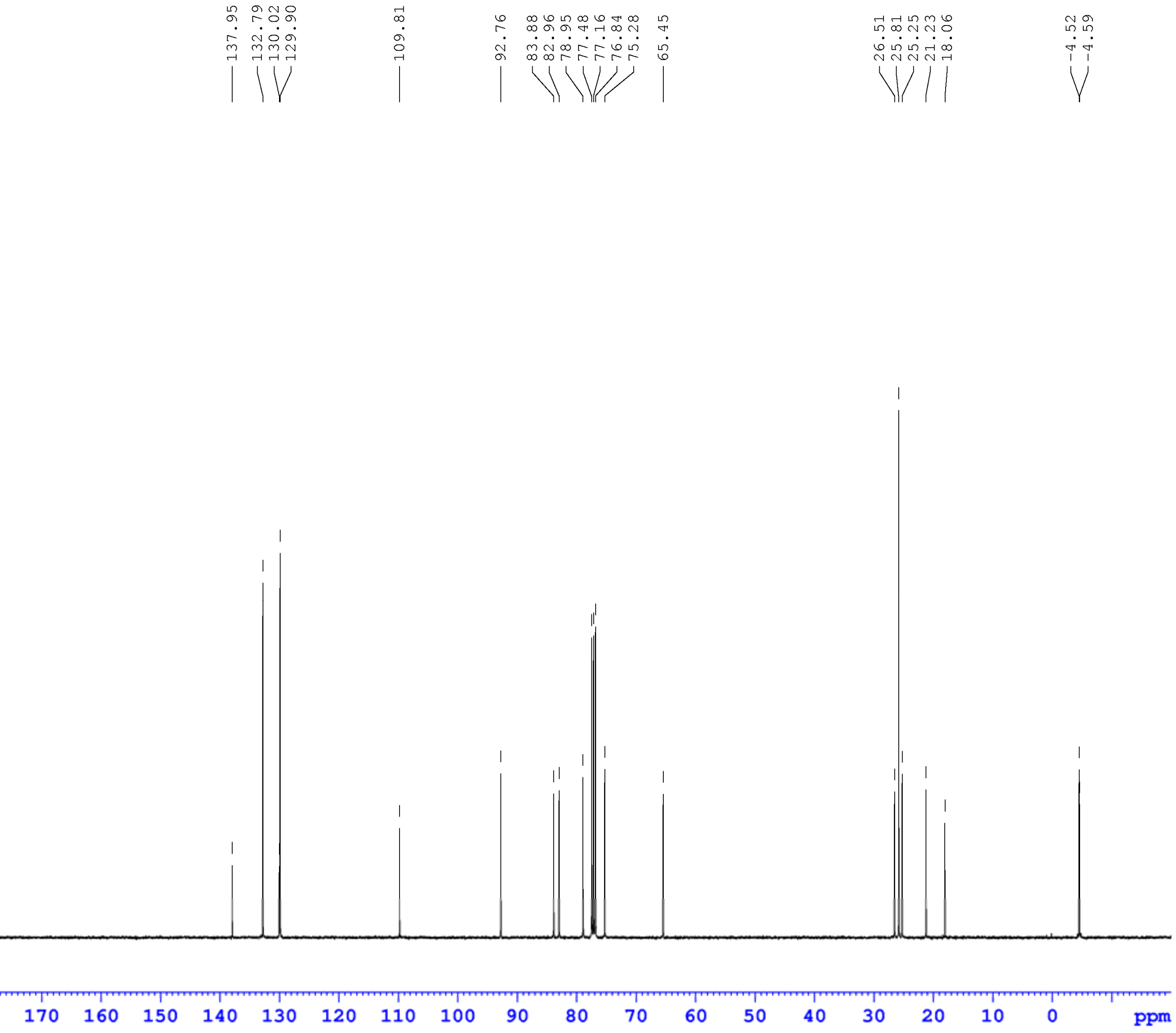

Supplementary Figure 6. ^{13}C NMR spectrum of compound 18b

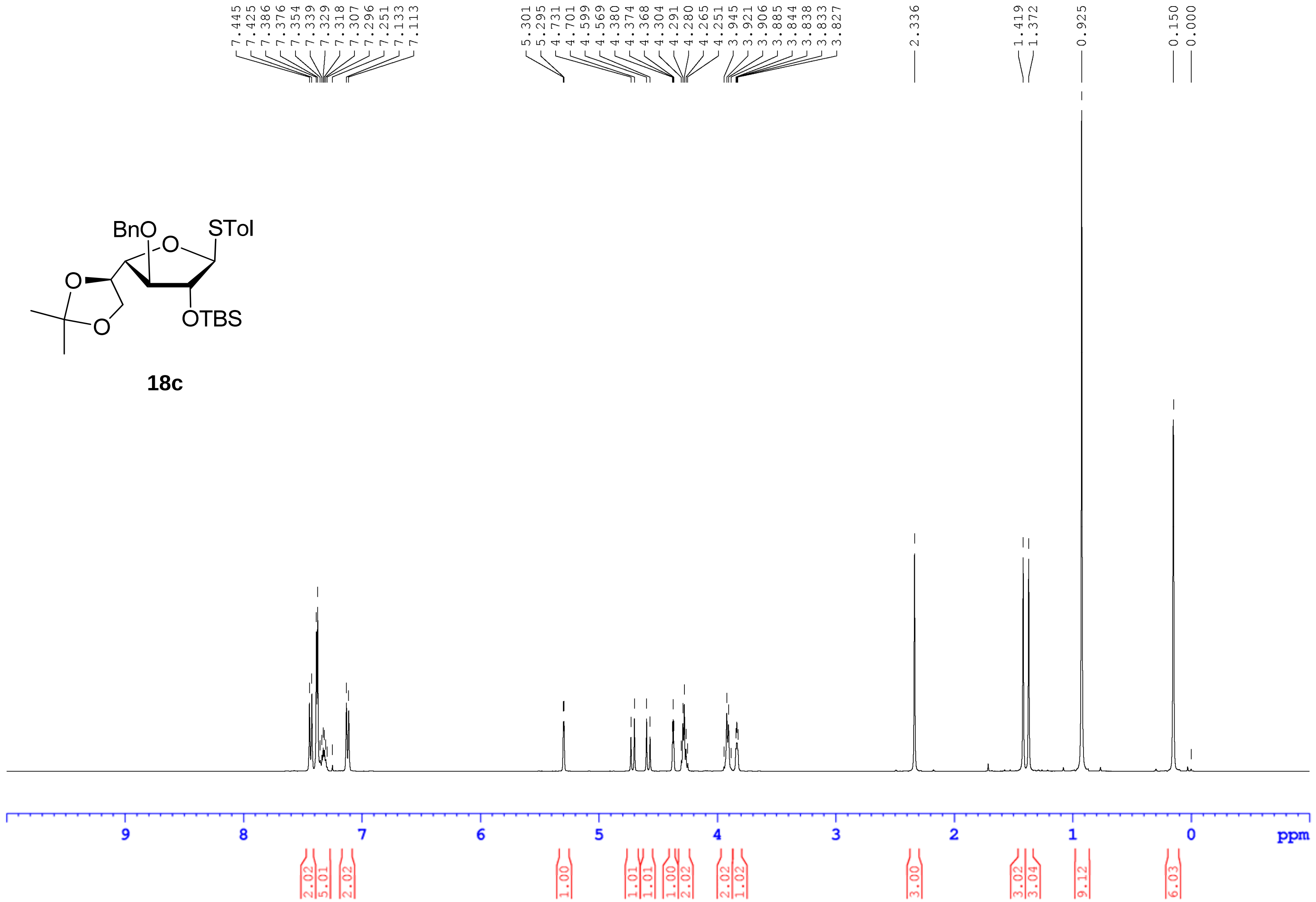

Supplementary Figure 7. ^{1}H NMR spectrum of compound 18c

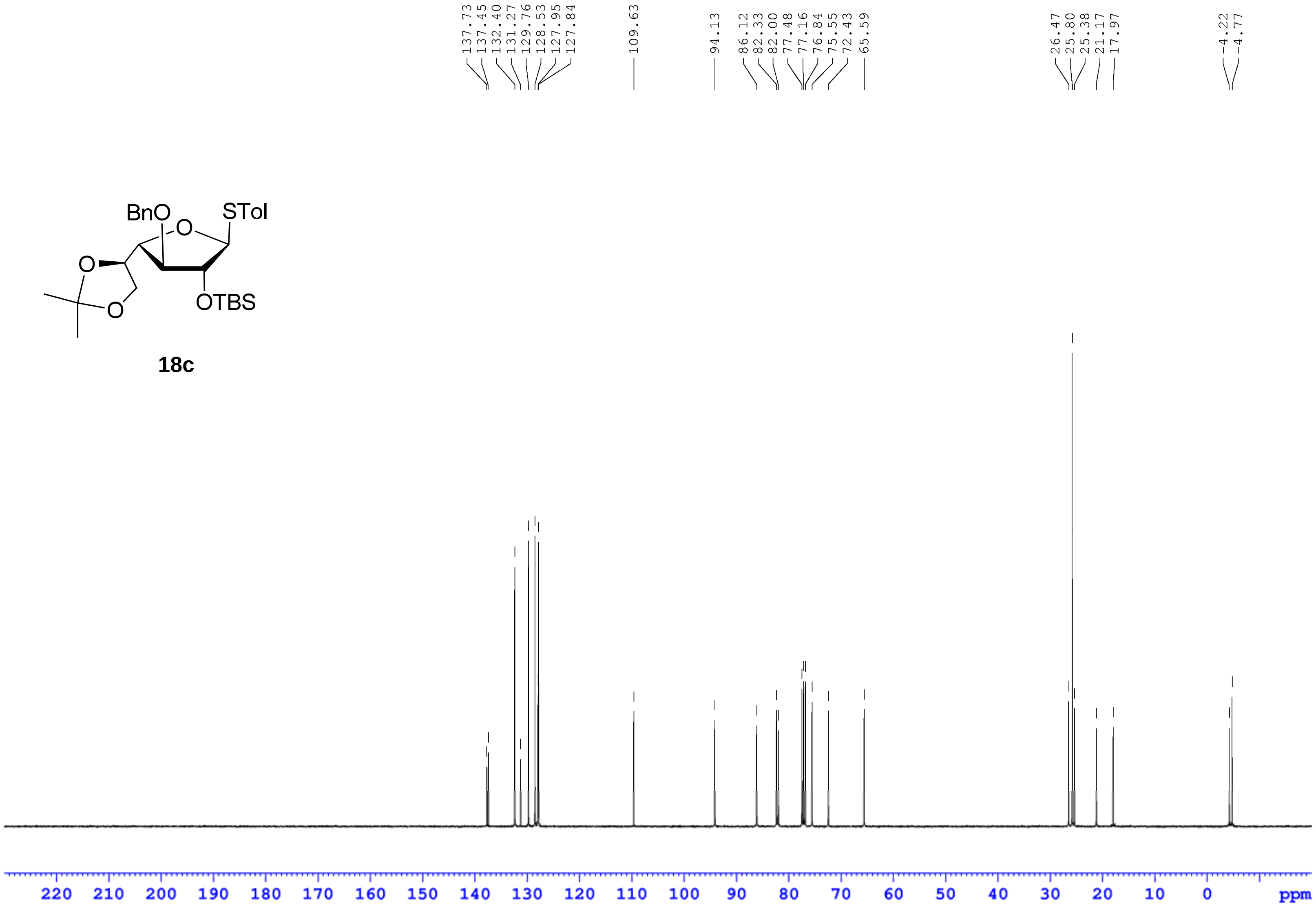

Supplementary Figure 8. ^{13}C NMR spectrum of compound 18c

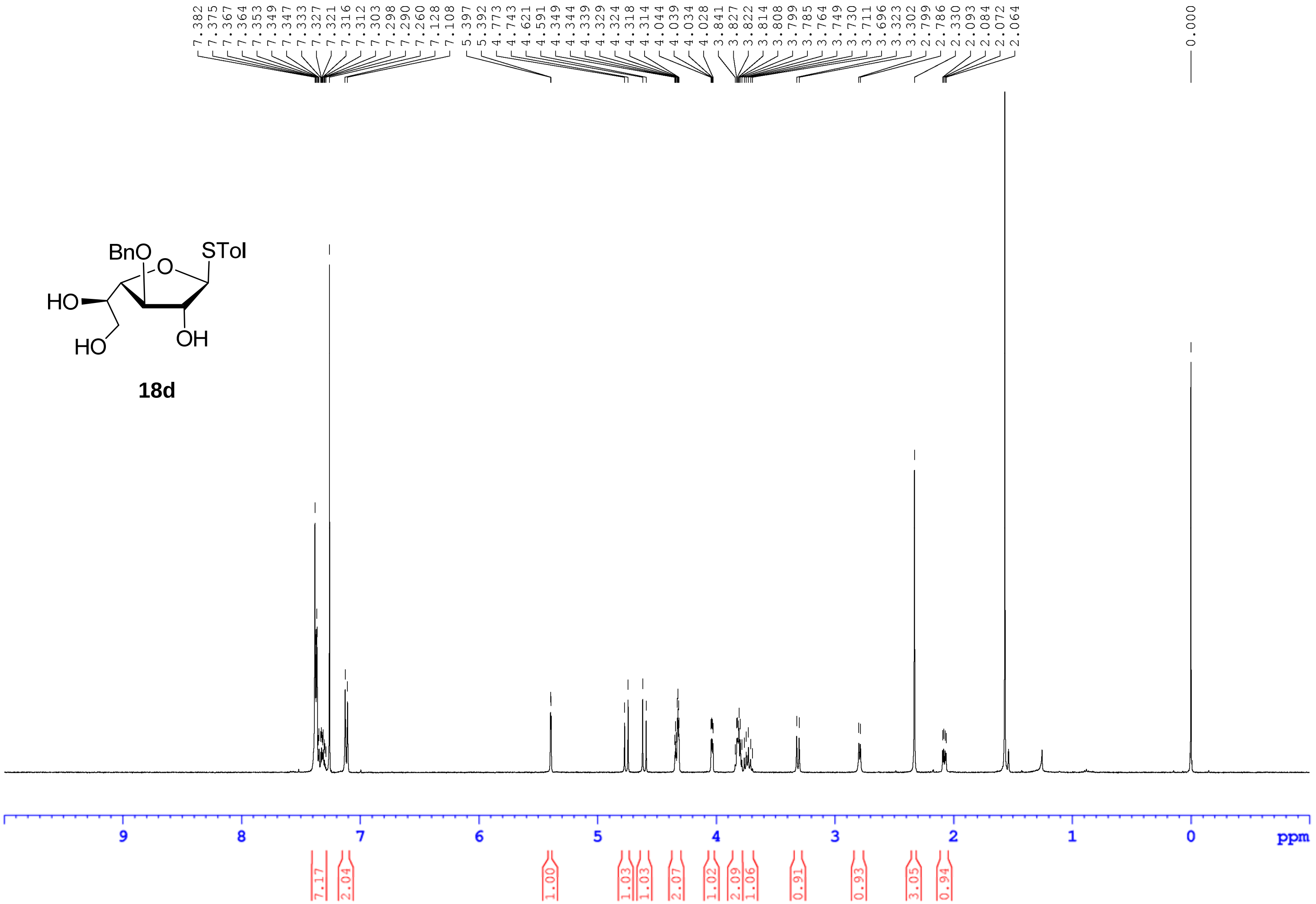

Supplementary Figure 9. ^{1}H NMR spectrum of compound 18d

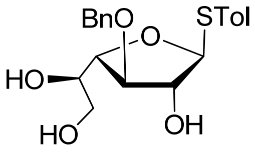

18d

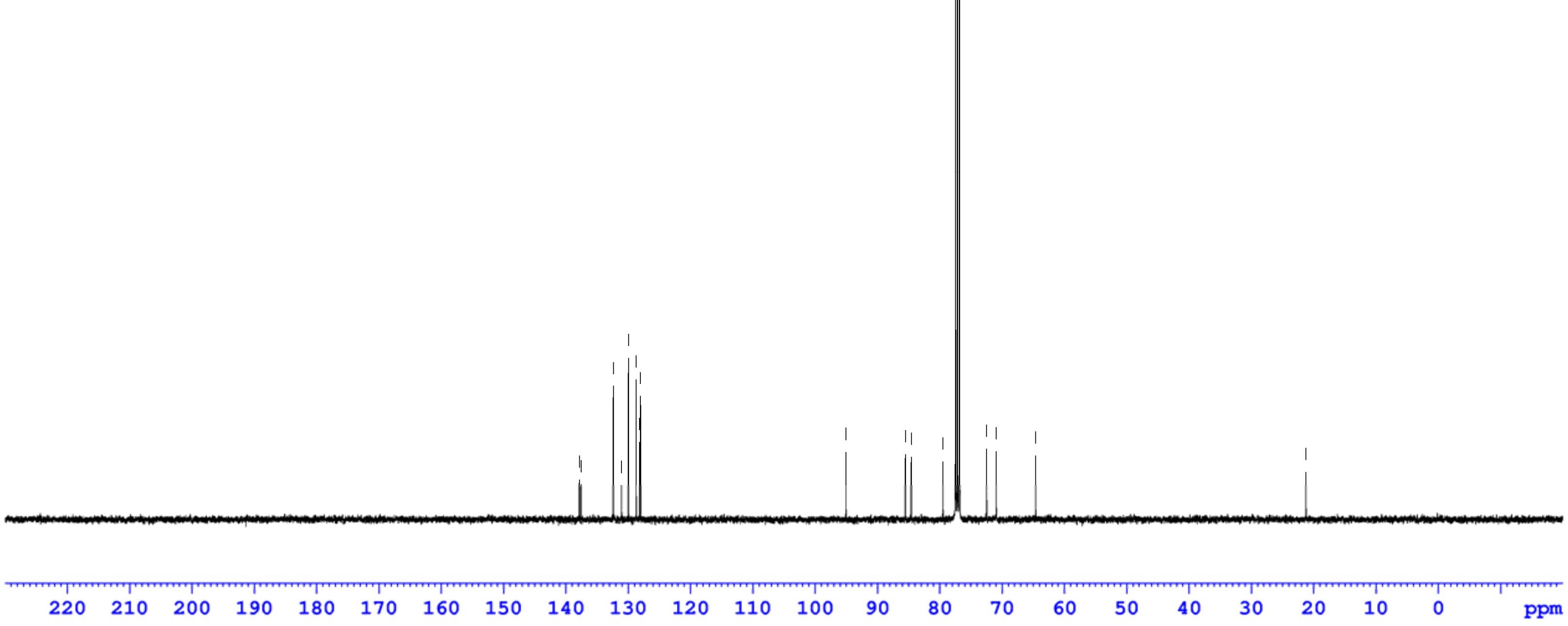

Supplementary Figure 10. ^{13}C NMR spectrum of compound 18d

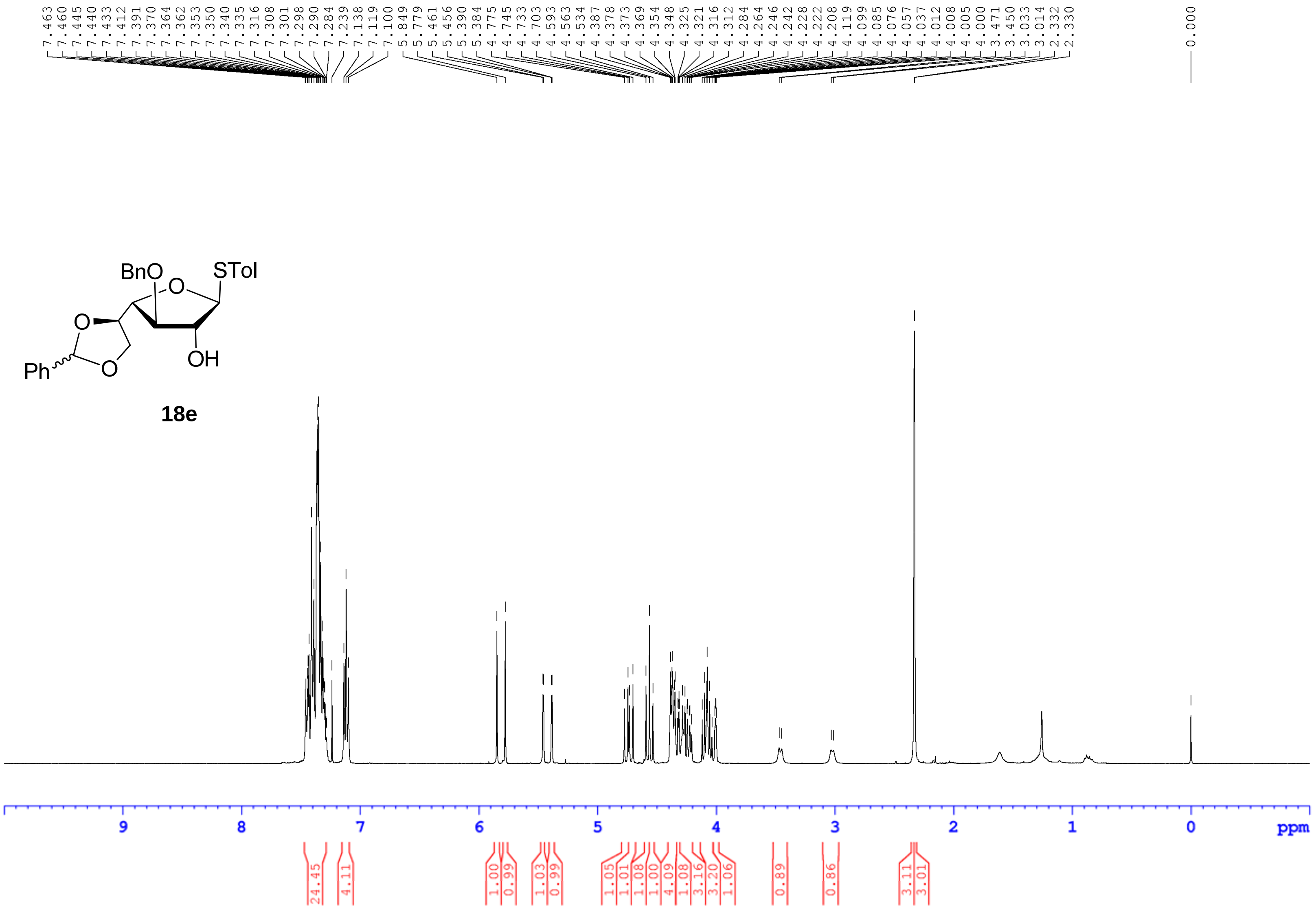

Supplementary Figure 11. ¹H NMR spectrum of compound 18e

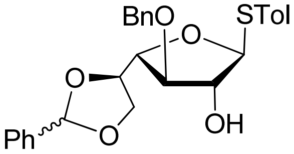

18e

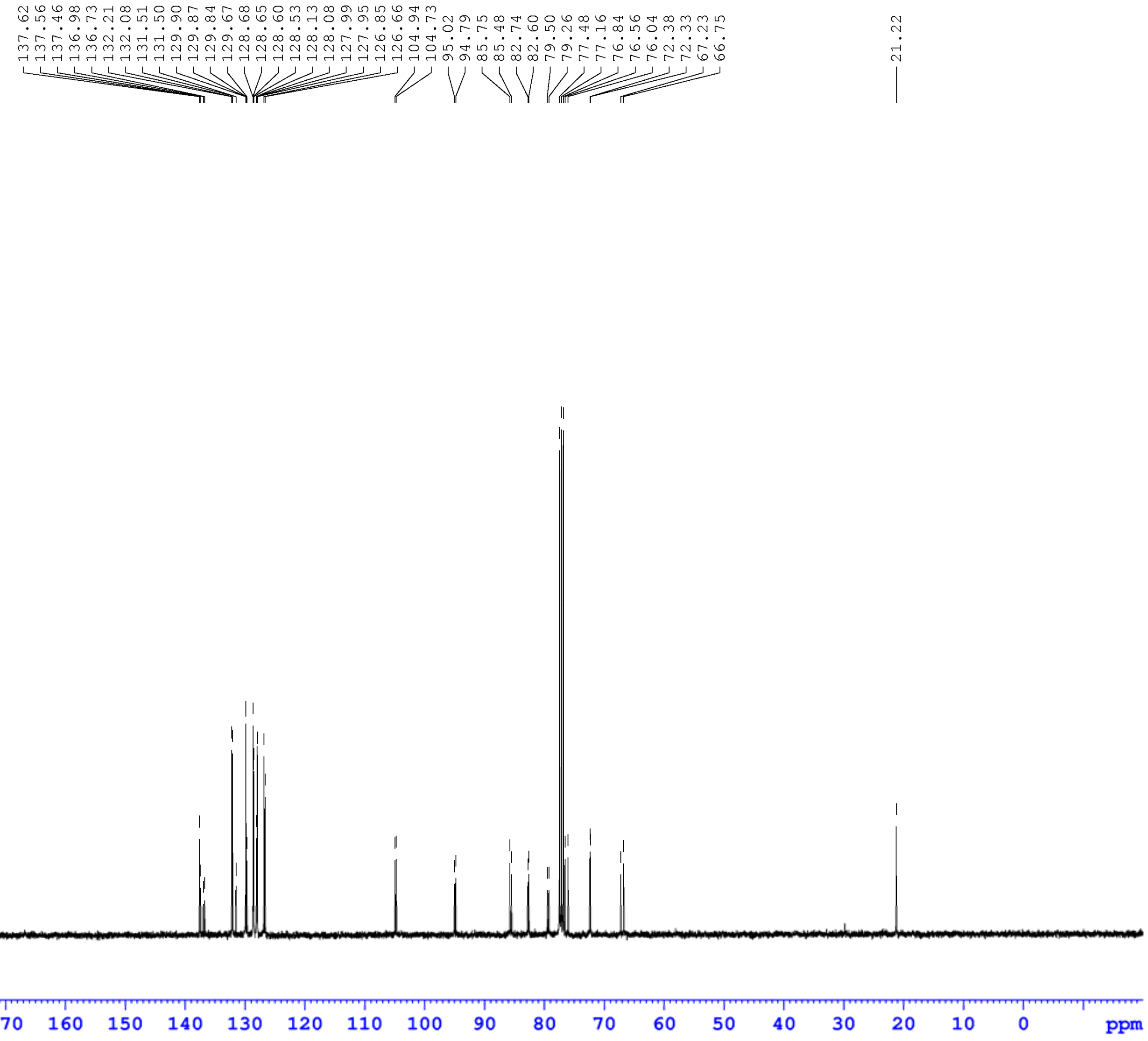

Supplementary Figure 12. ^{13}C NMR spectrum of compound 18e

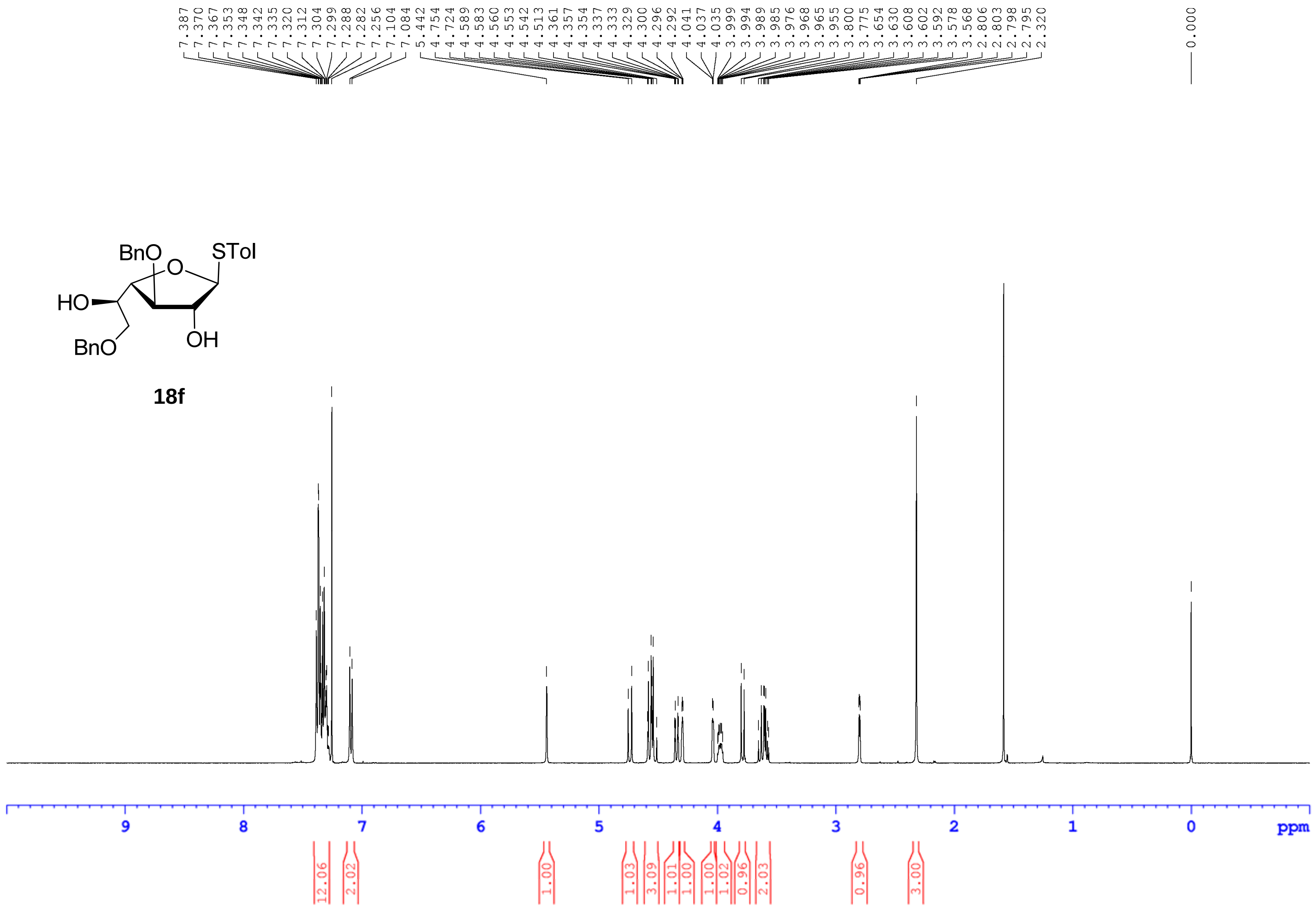

18f

Supplementary Figure 13. ^{1}H NMR spectrum of compound 18f

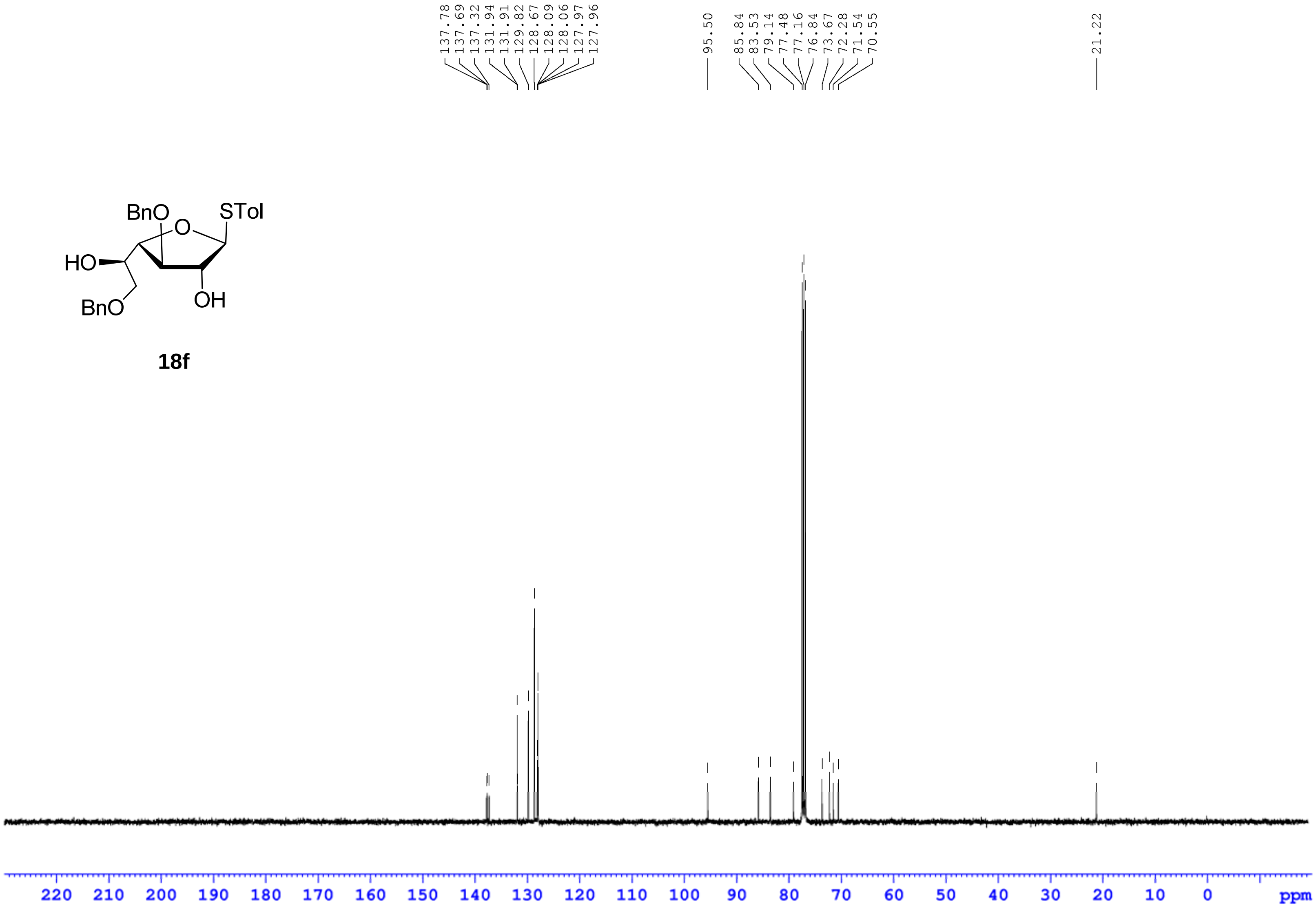

18f

Supplementary Figure 14. ^{13}C NMR spectrum of compound 18f

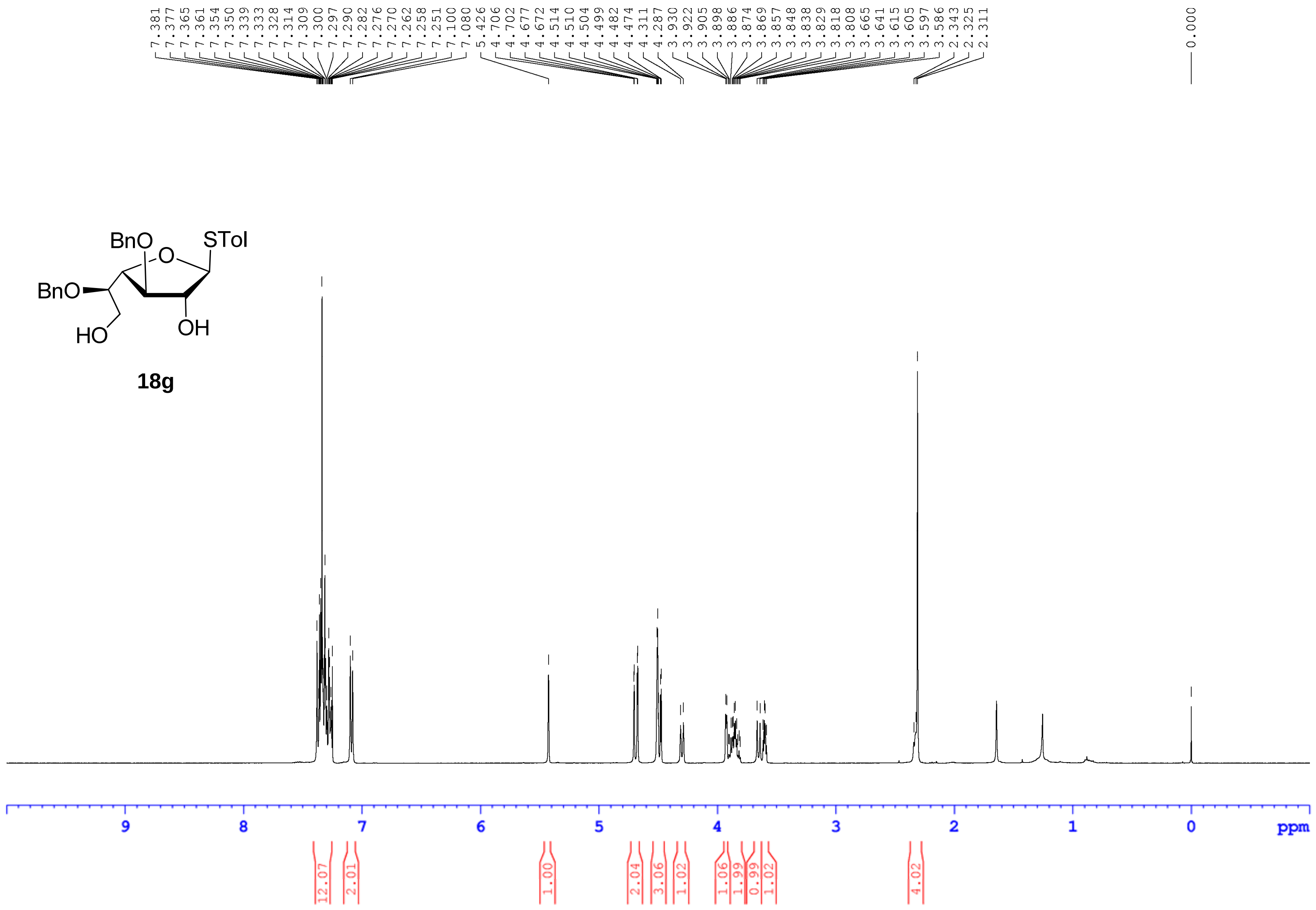

18g

Supplementary Figure 15. ^{1}H NMR spectrum of compound 18g

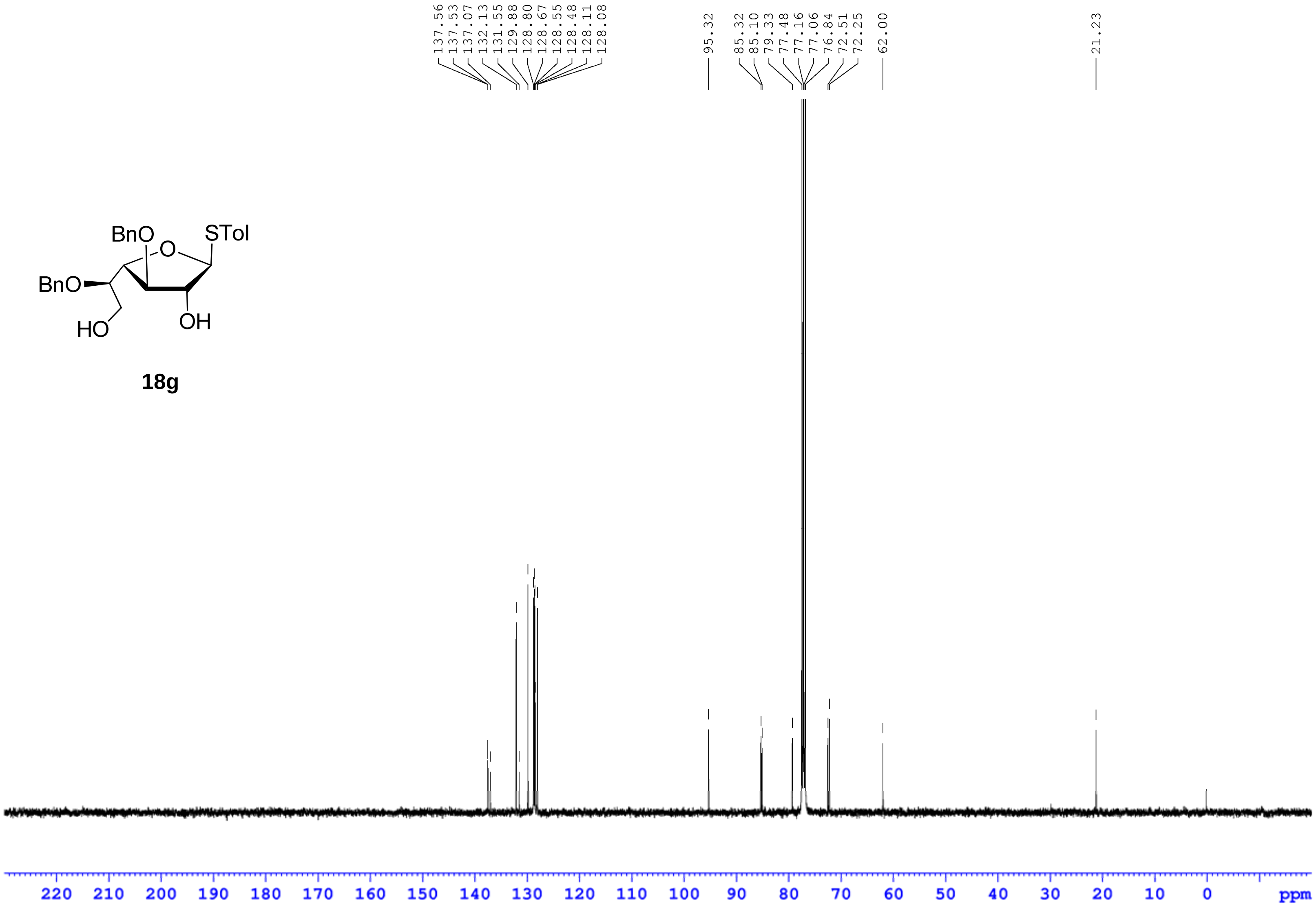

Supplementary Figure 16. ^{13}C NMR spectrum of compound 18g

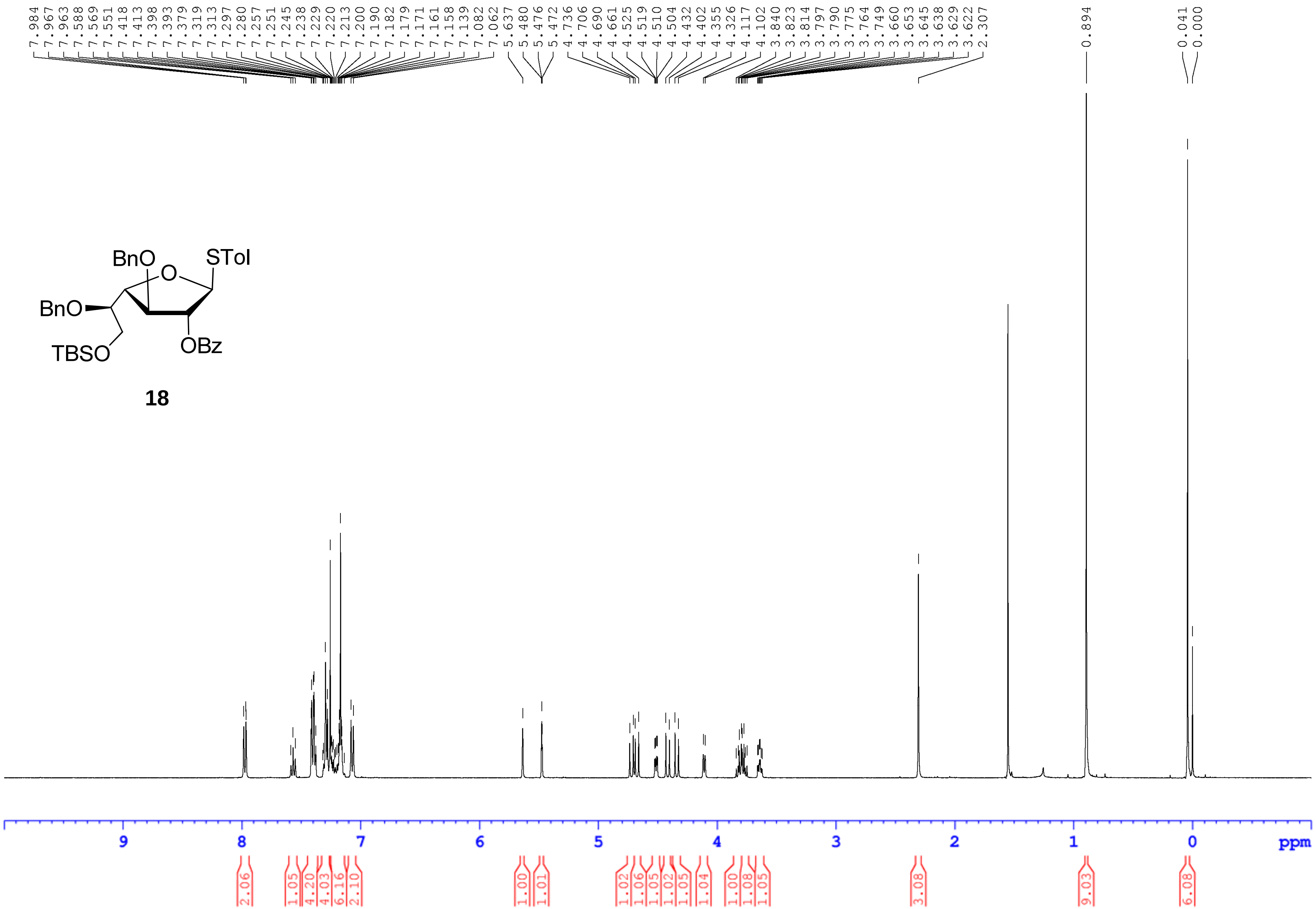

Supplementary Figure 17. ^{1}H NMR spectrum of compound 18

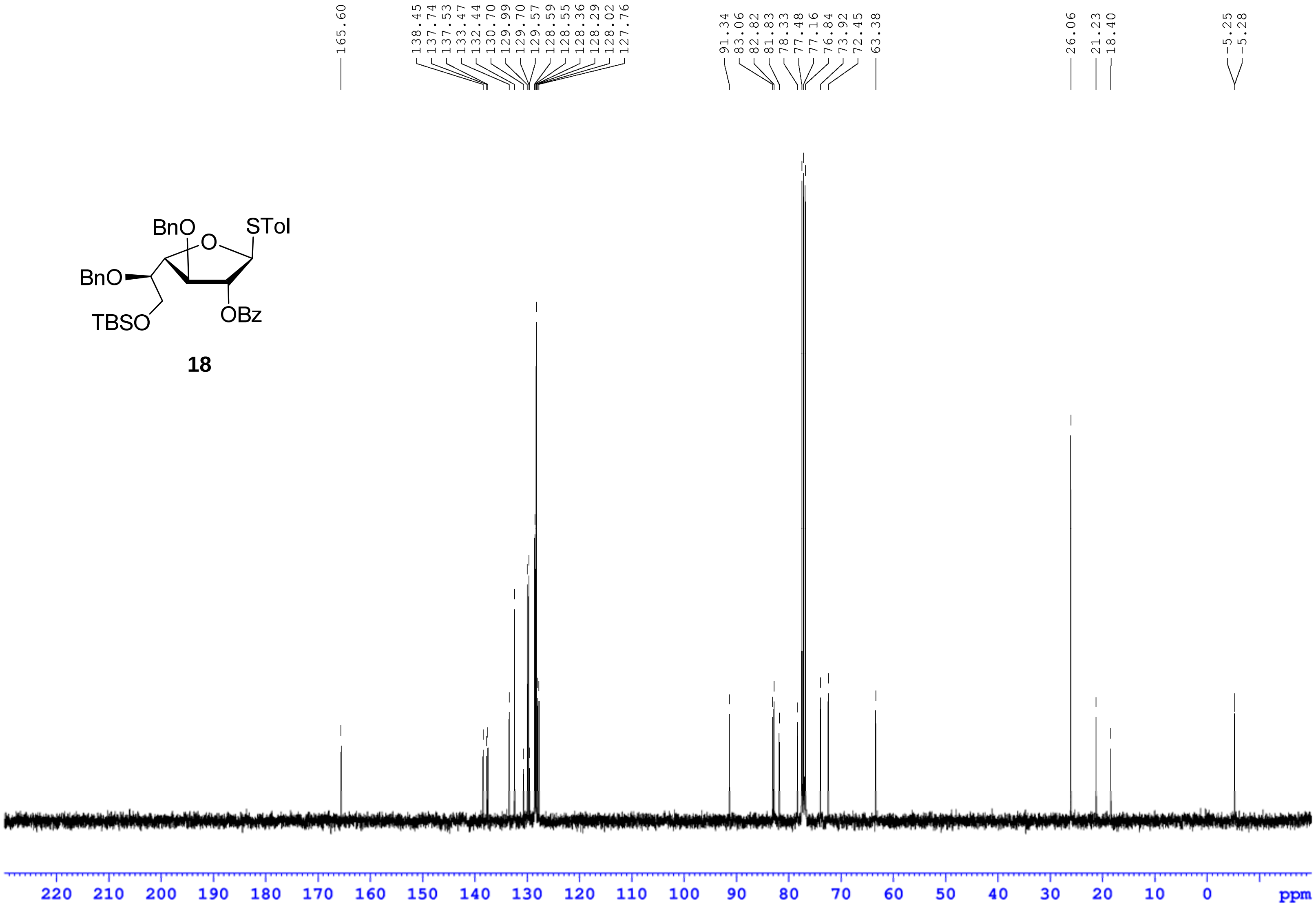

18

Supplementary Figure 18. ^{13}C NMR spectrum of compound 18

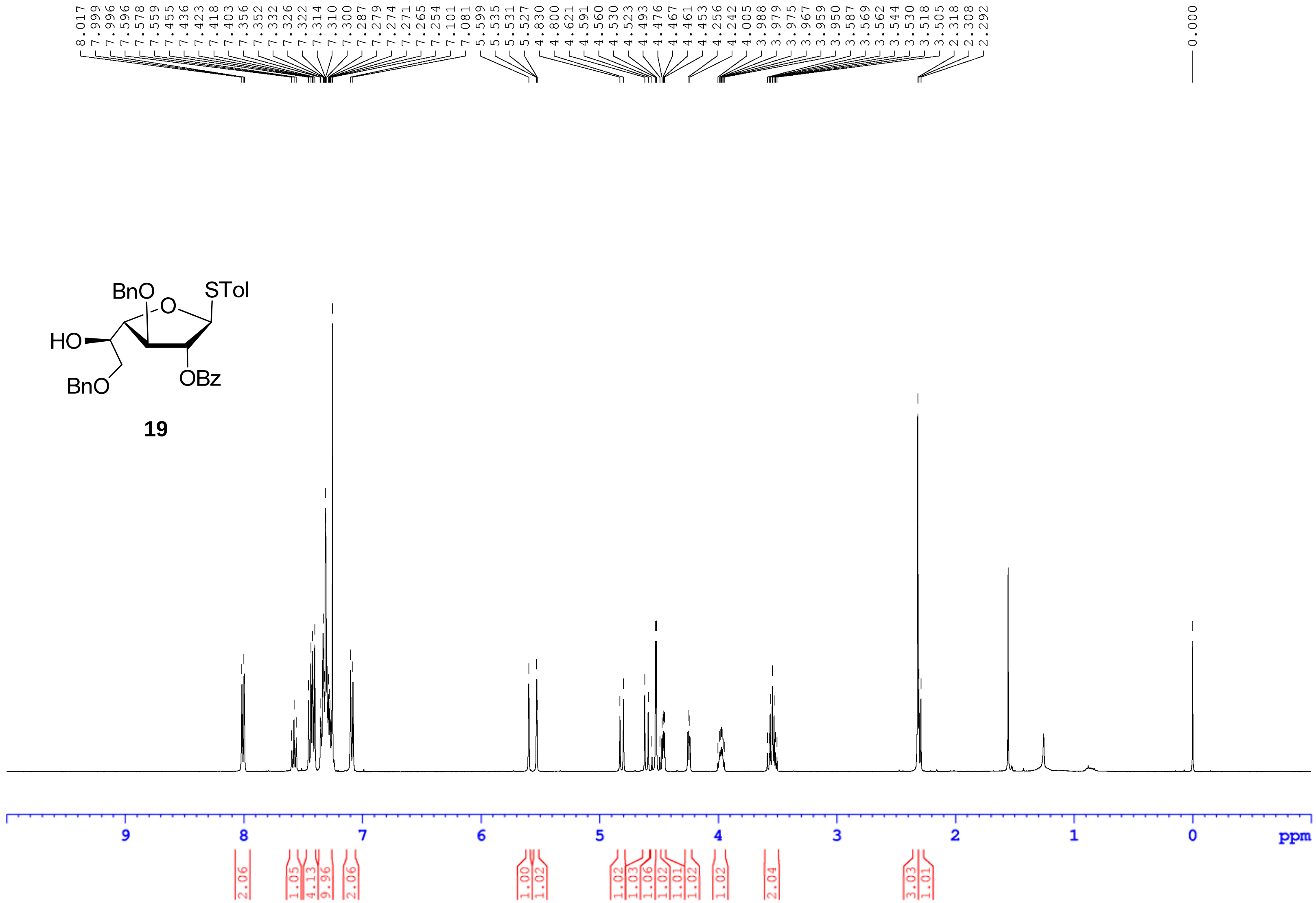

Supplementary Figure 19. ^{1}**H NMR spectrum of compound 19**

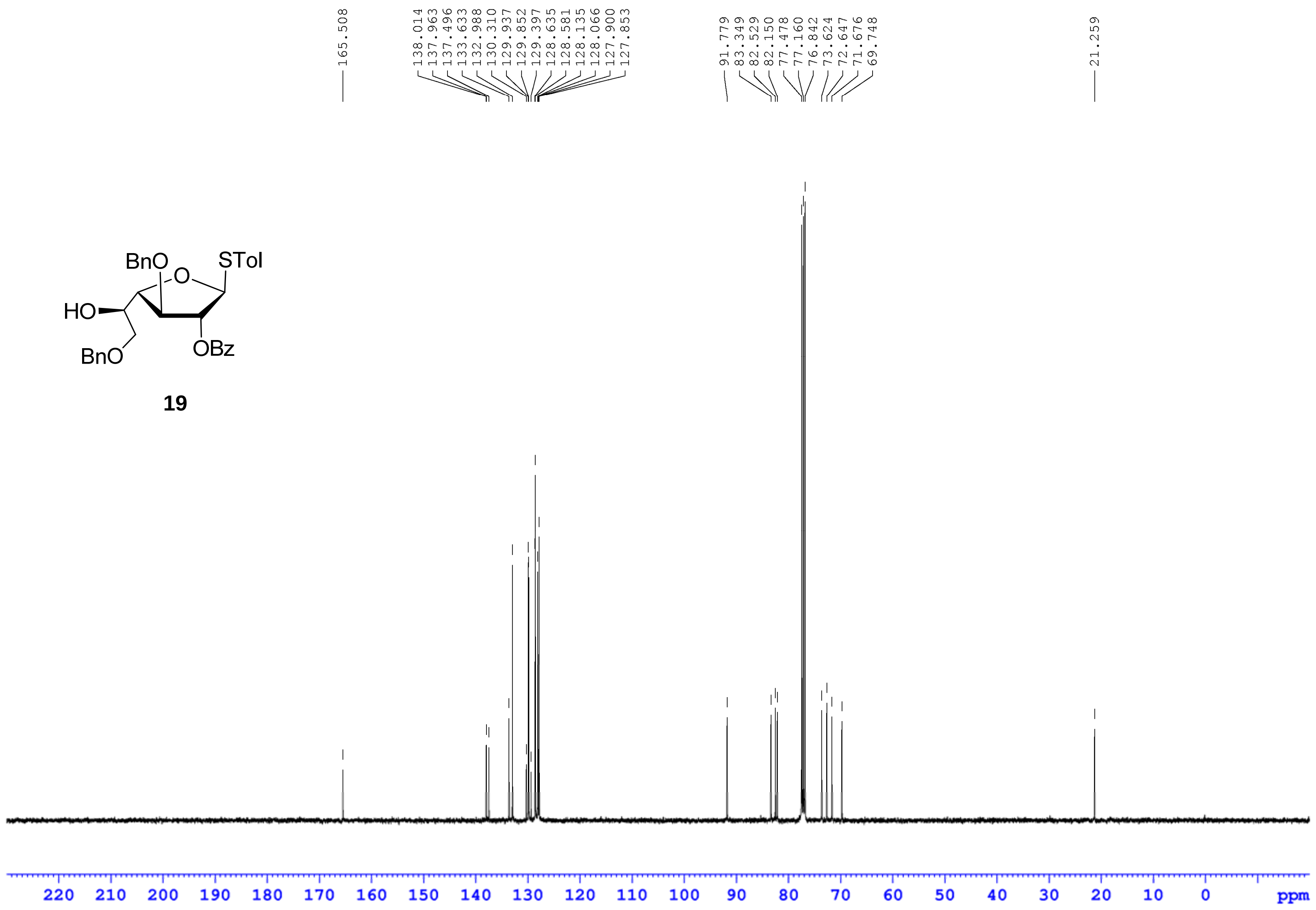

Supplementary Figure 20. ^{13}C NMR spectrum of compound 19

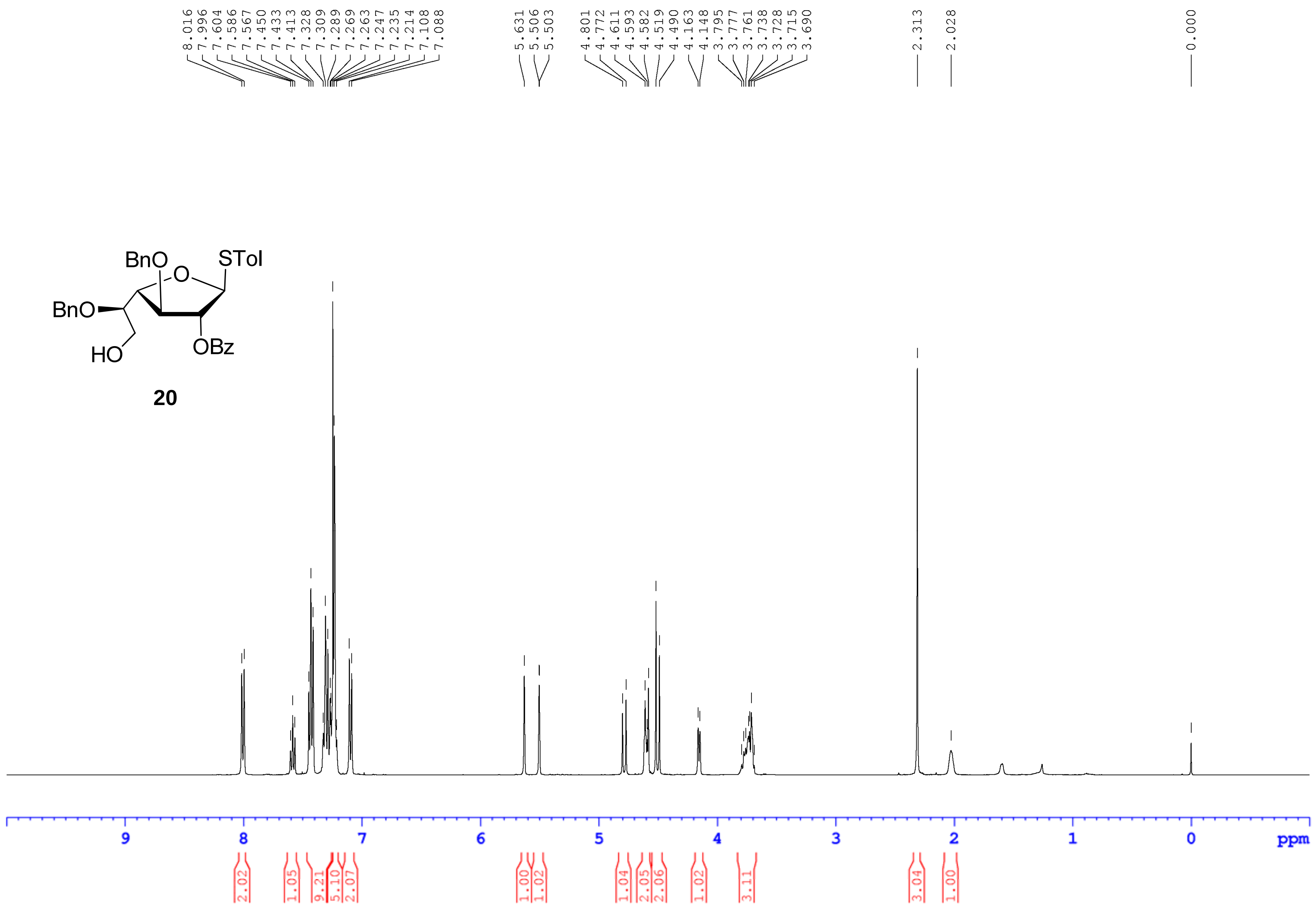

Supplementary Figure 21. ^{1}**H NMR spectrum of compound 20**

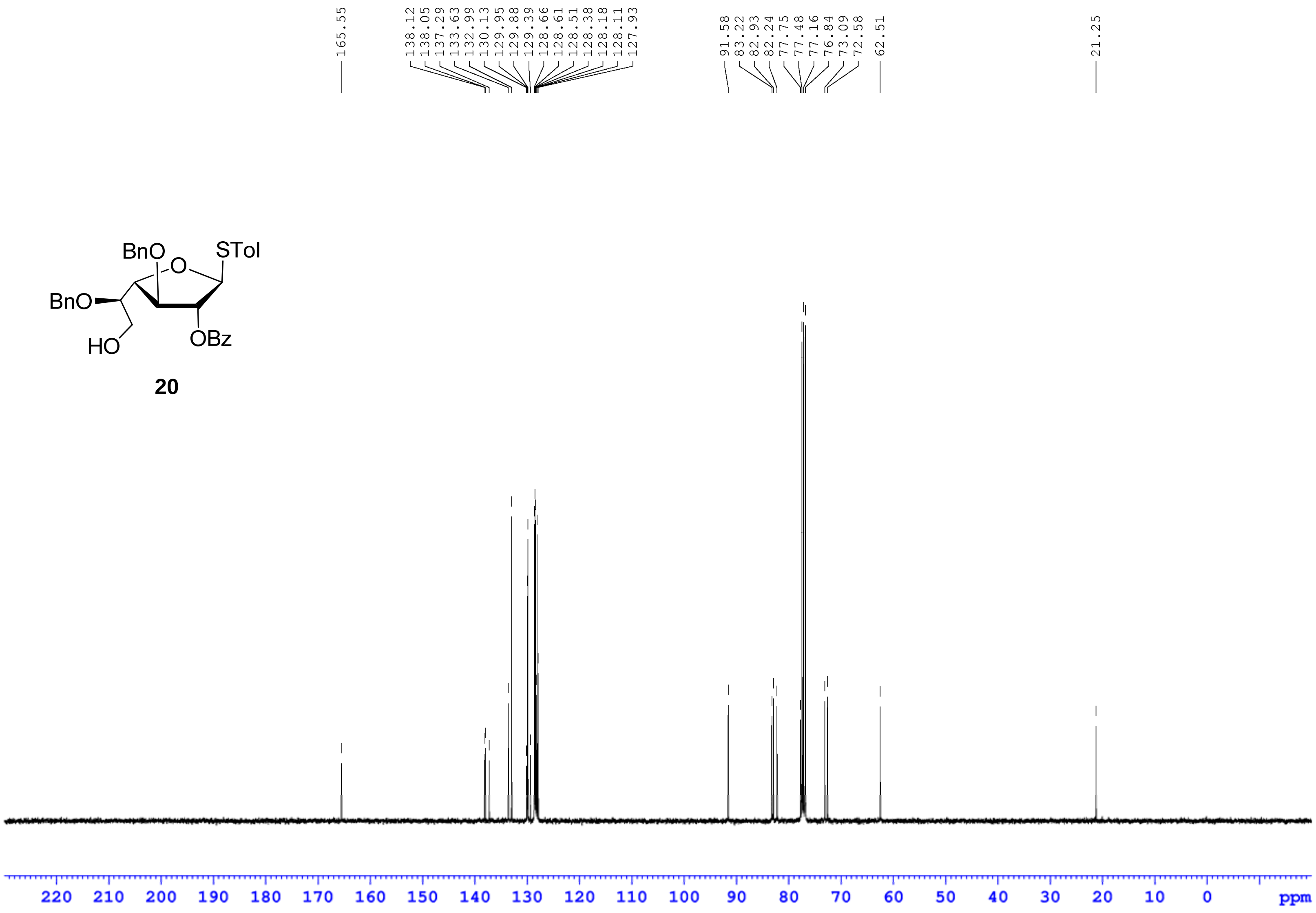

Supplementary Figure 22. ^{13}C NMR spectrum of compound 20

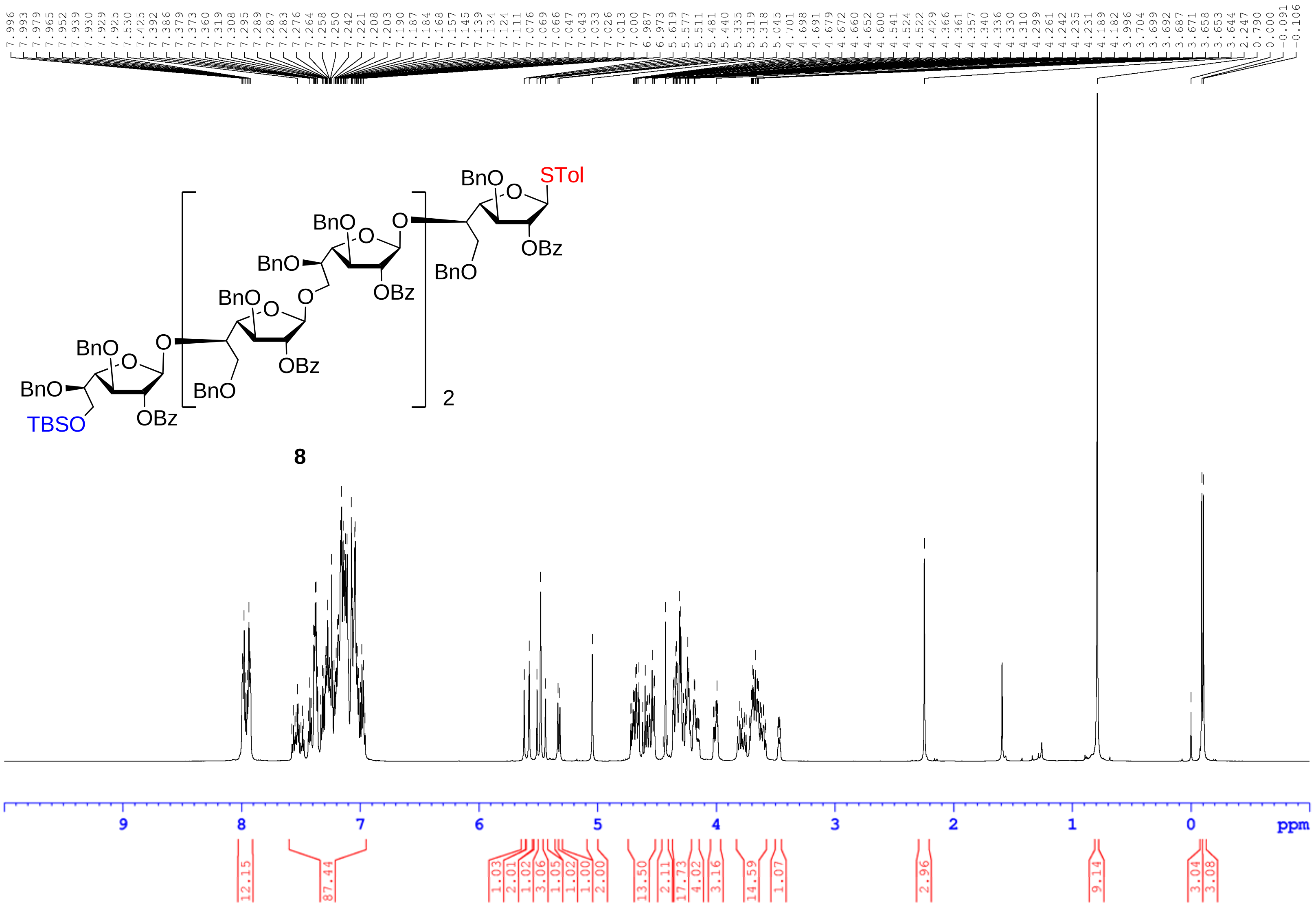

Supplementary Figure 23. ^{1}H NMR spectrum of compound 8

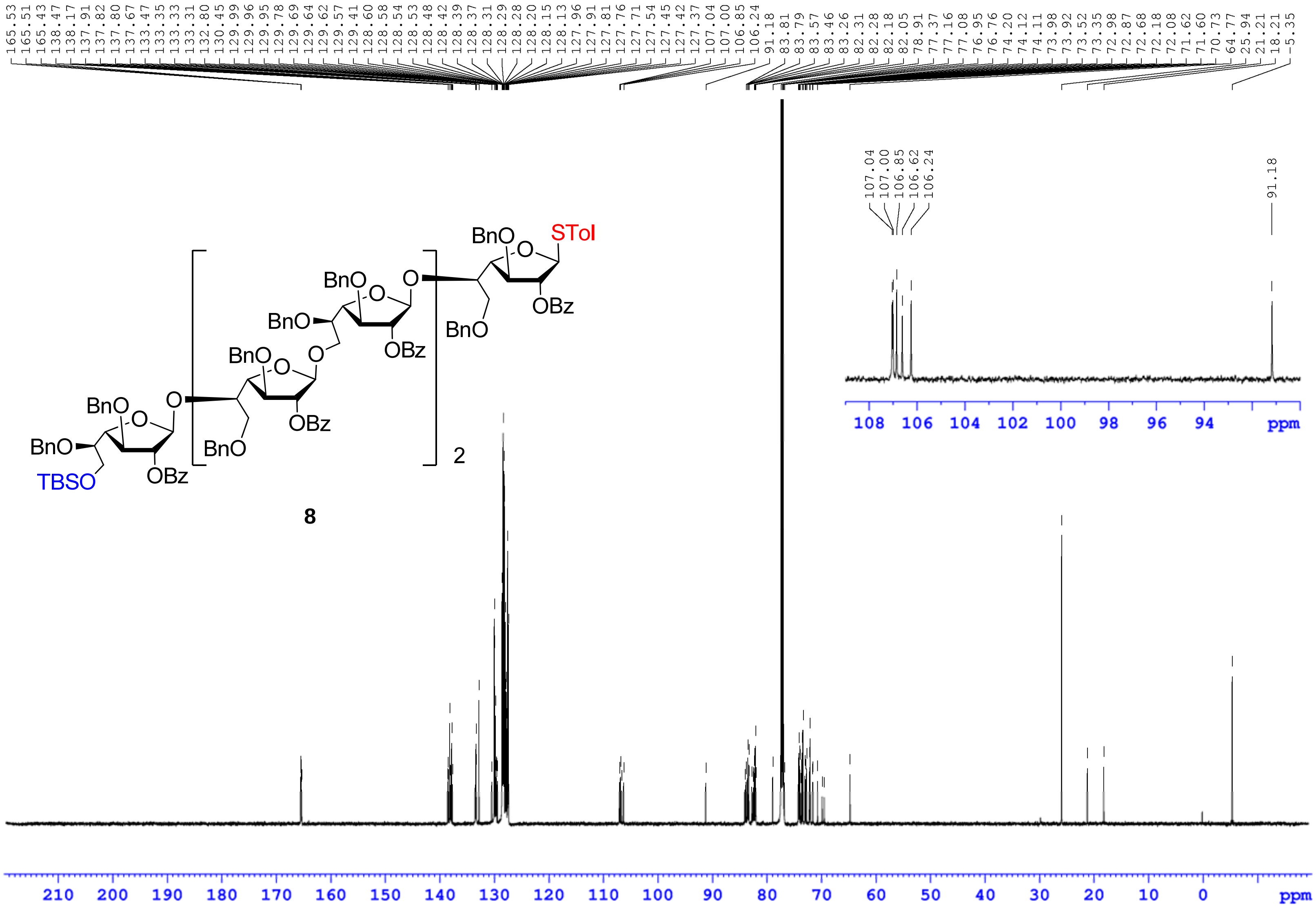

Supplementary Figure 24. **^{13}C NMR spectrum of compound 8**

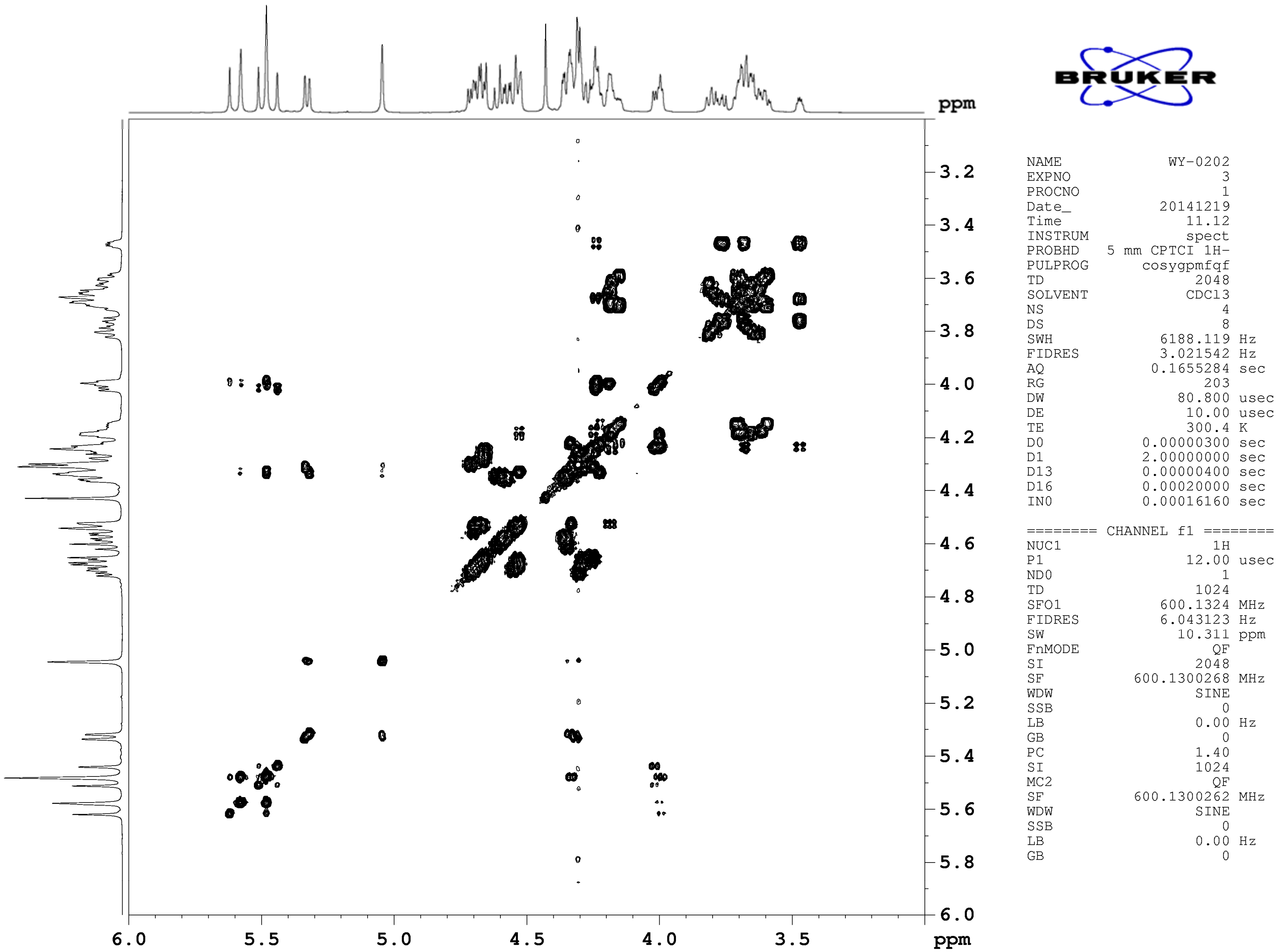

Supplementary Figure 25. COSY NMR spectrum of compound 8

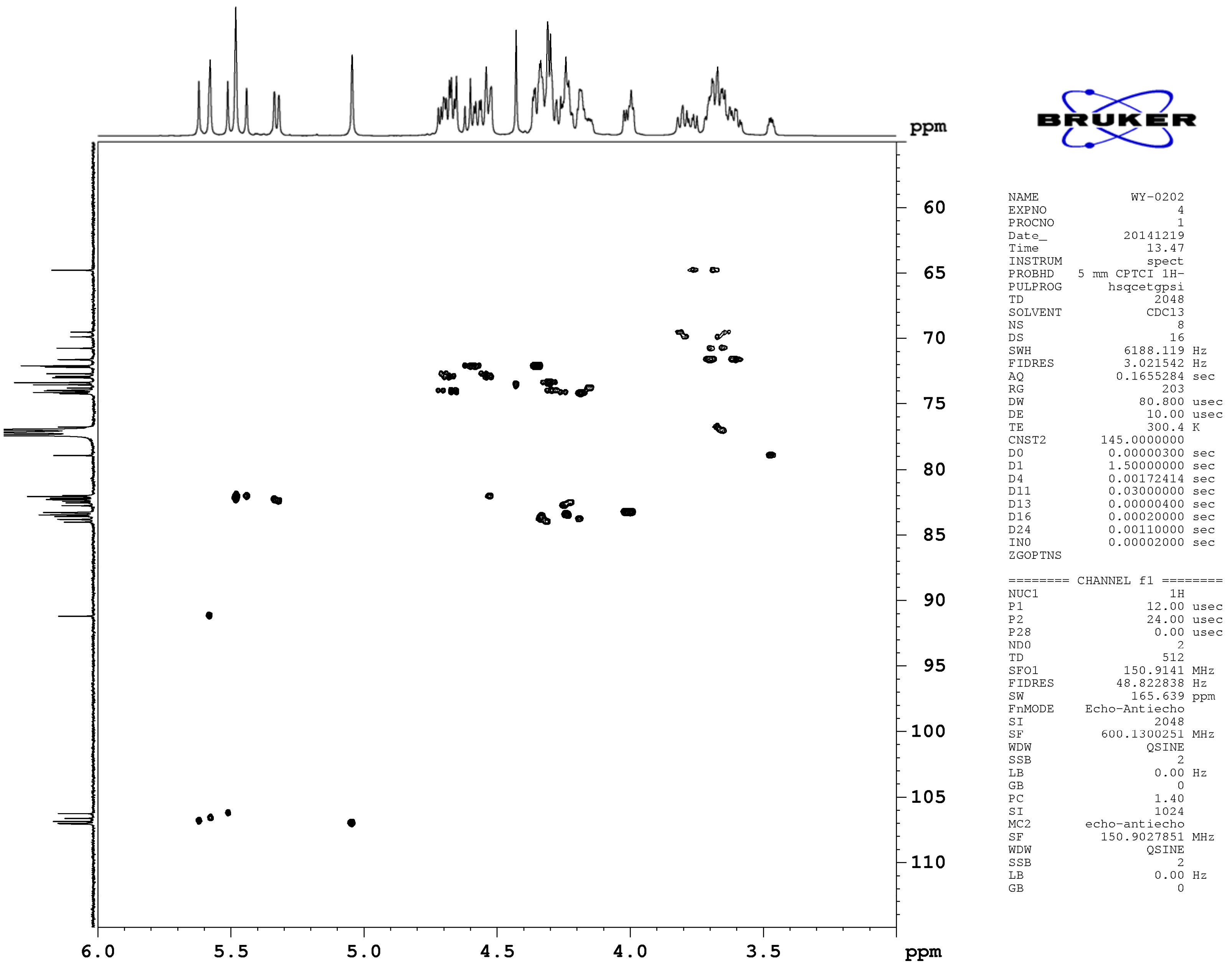

Supplementary Figure 26. HSQC NMR spectrum of compound 8

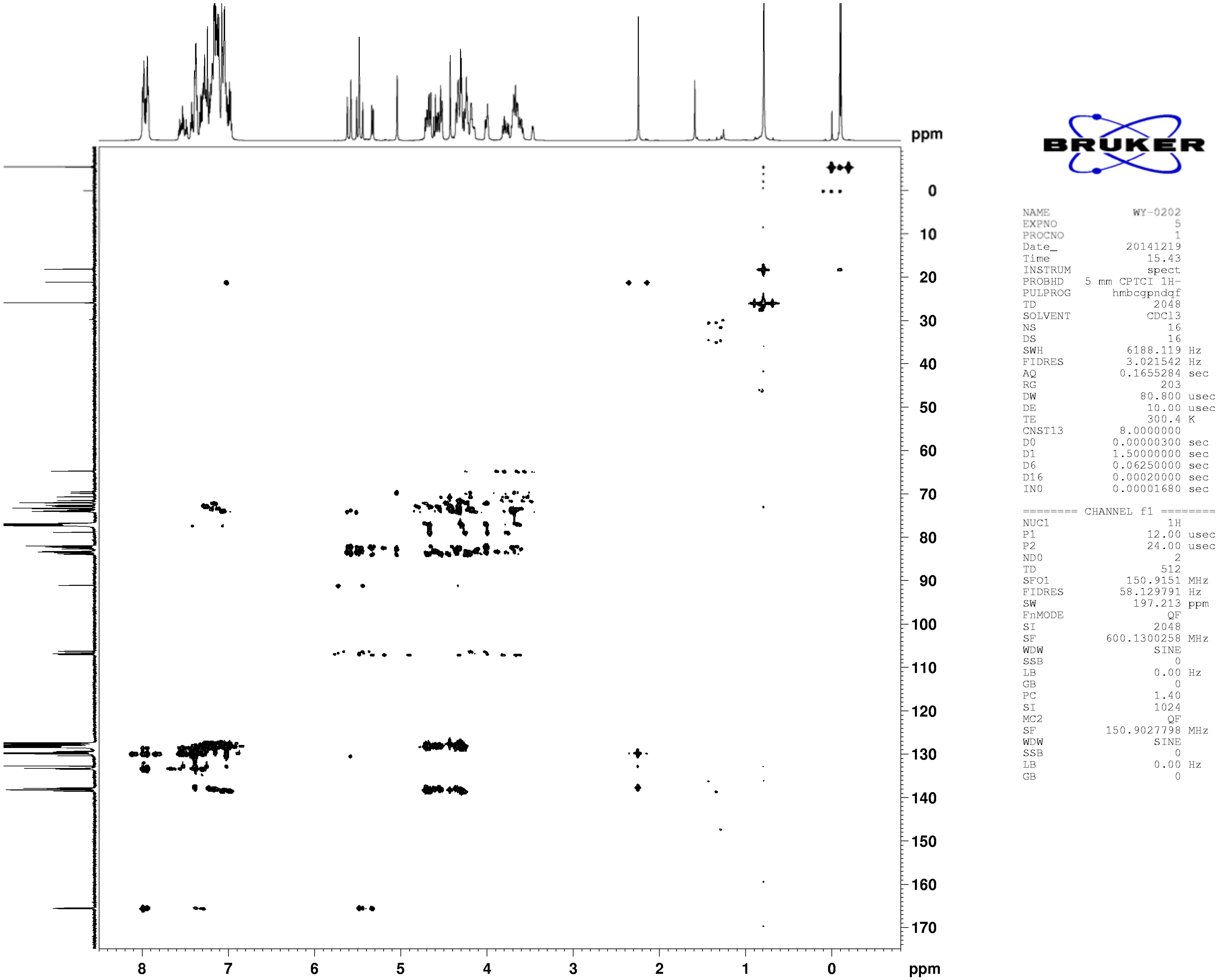

Supplementary Figure 27. HMBC NMR spectrum of compound 8

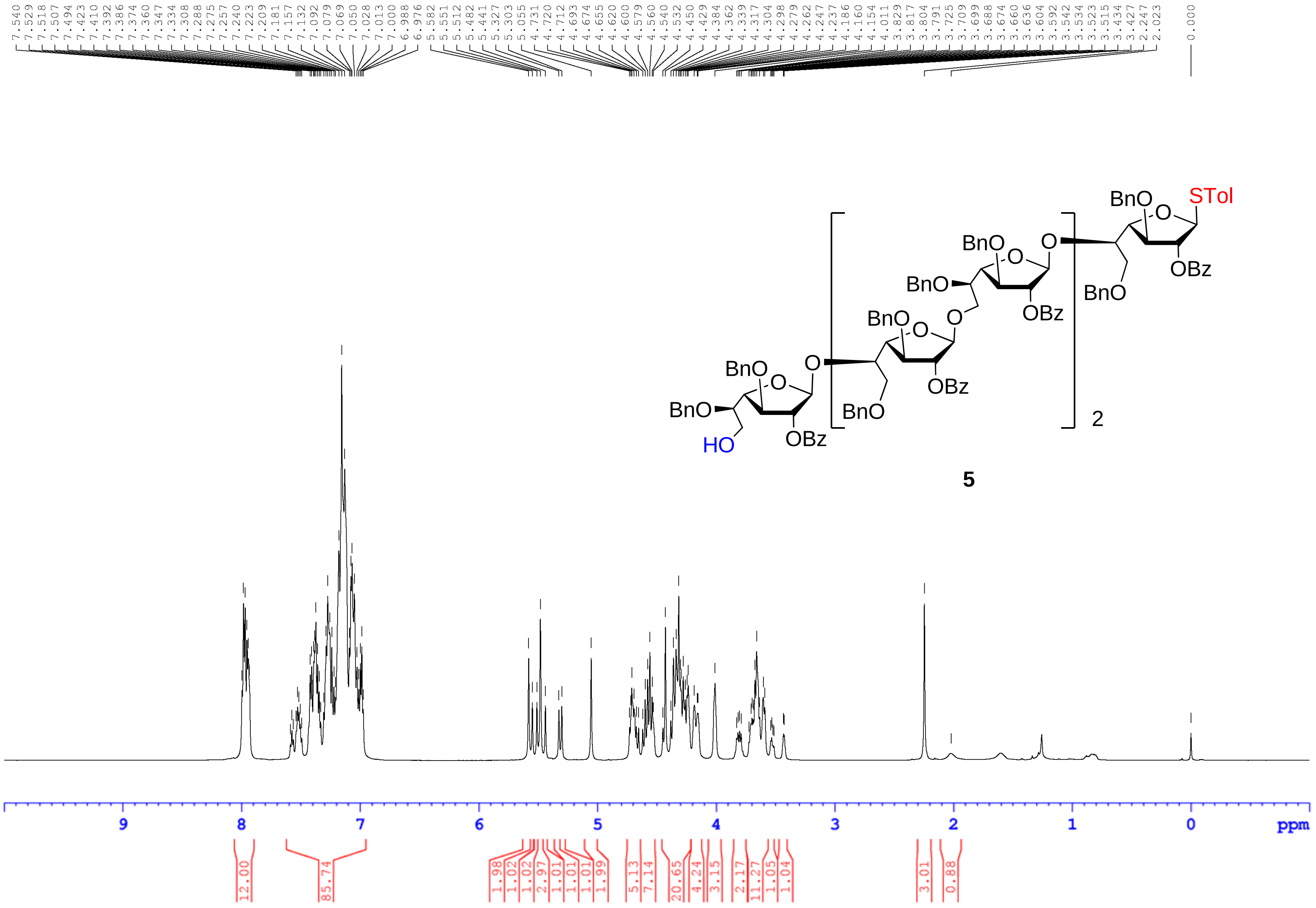

Supplementary Figure 28. ^{1}H NMR spectrum of compound 5

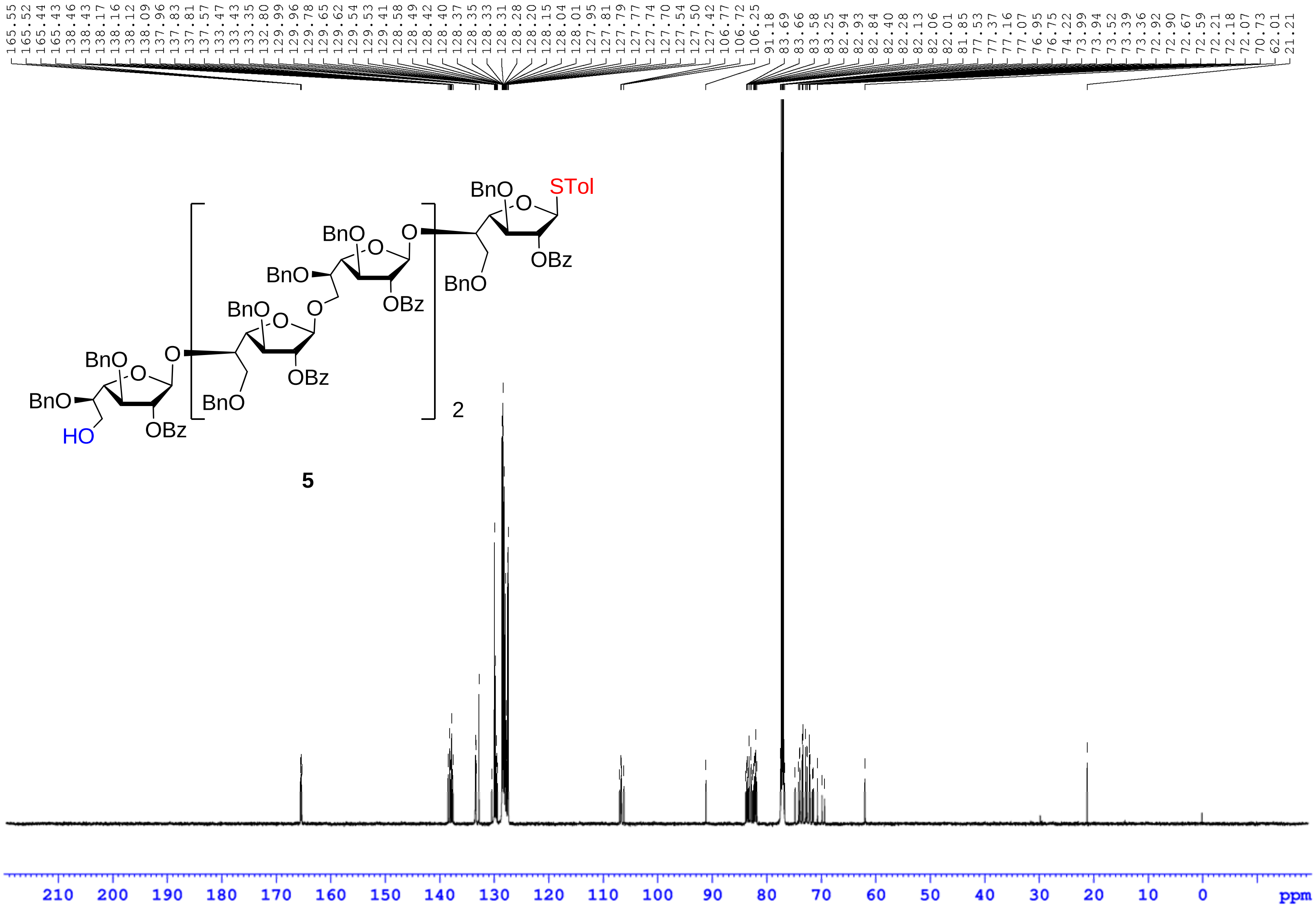

Supplementary Figure 29. ^{13}C **NMR spectrum of compound 5**

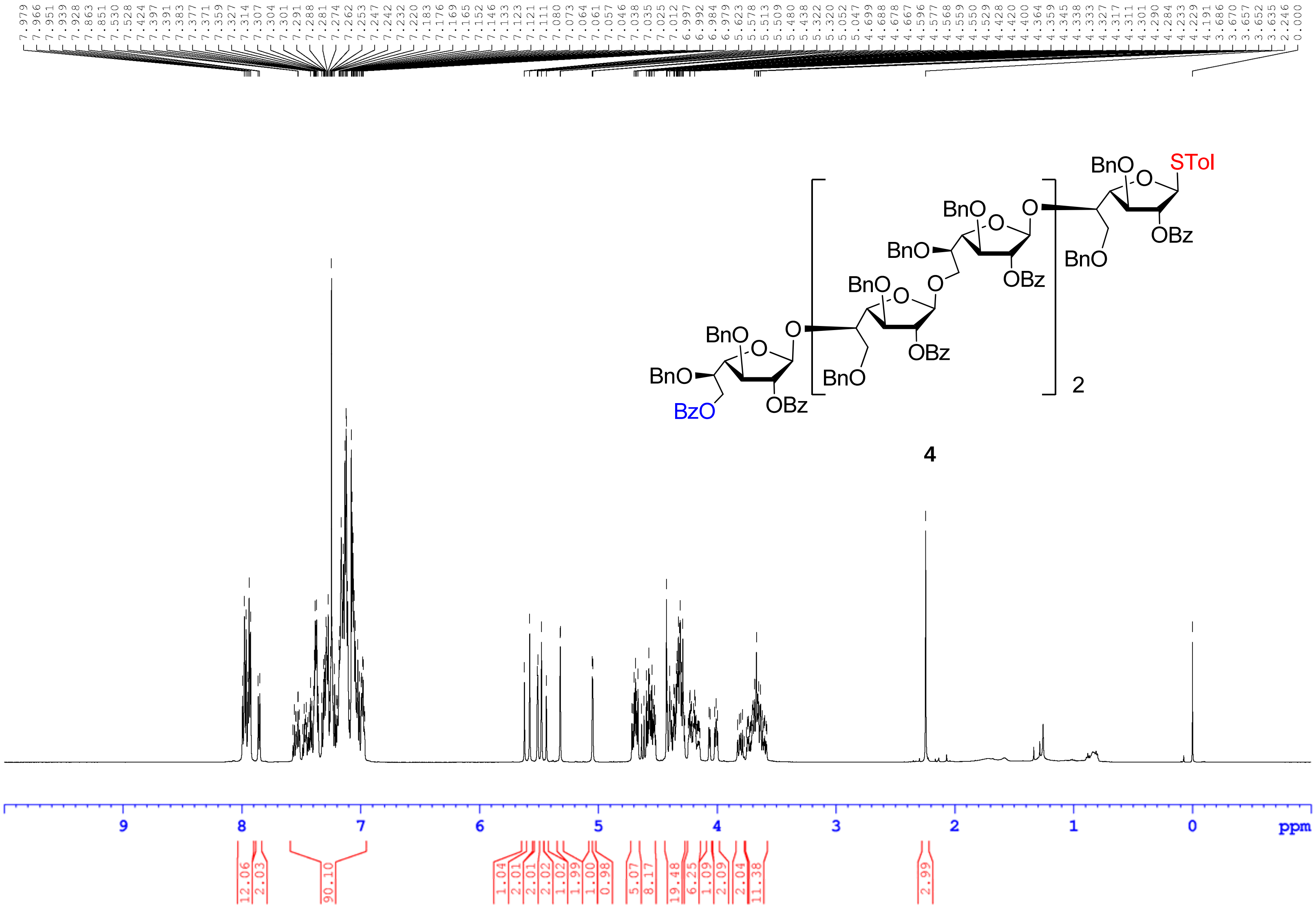

Supplementary Figure 30. ^{1}H **NMR spectrum of compound 4**

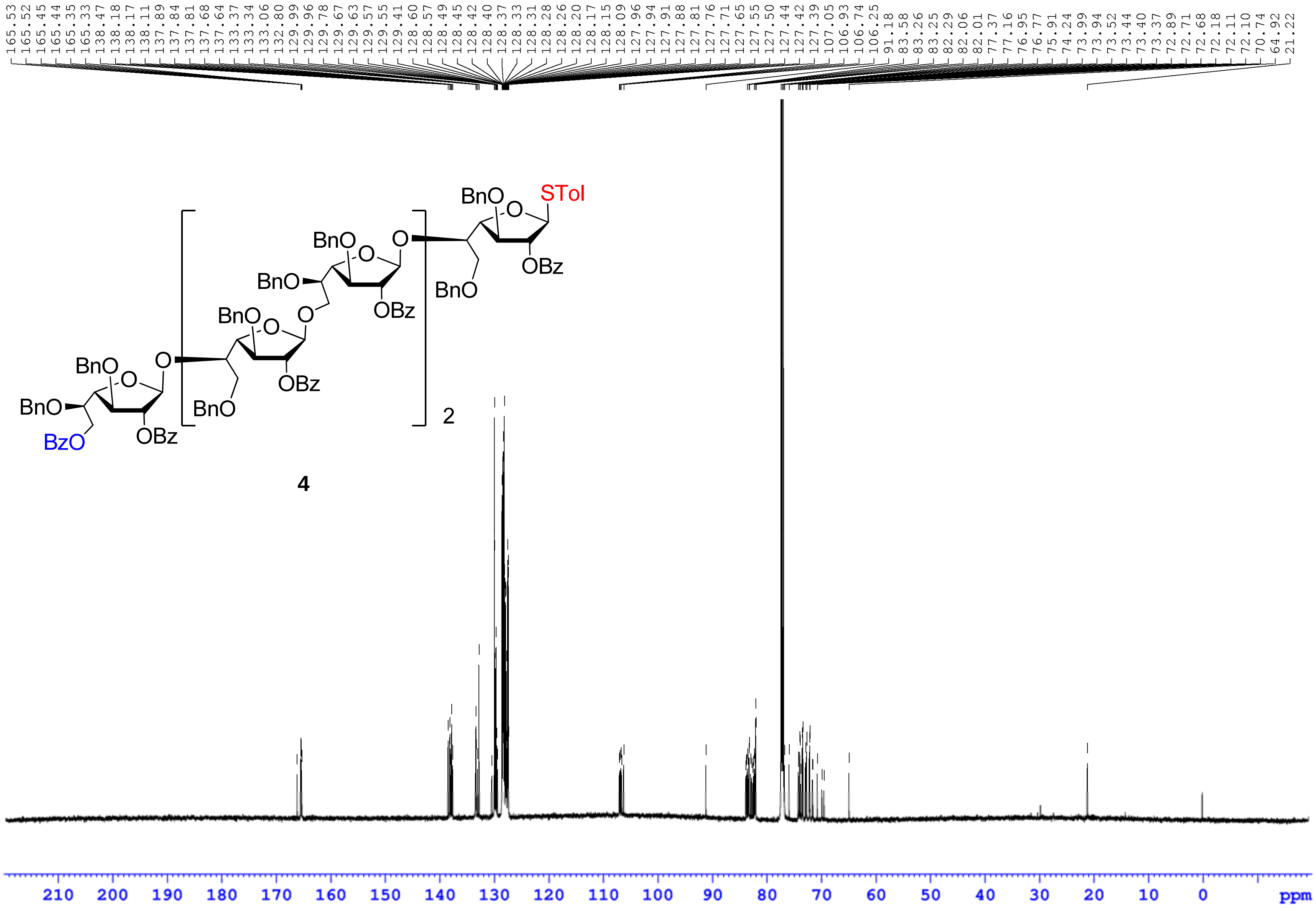

Supplementary Figure 31. ^{13}C NMR spectrum of compound 4

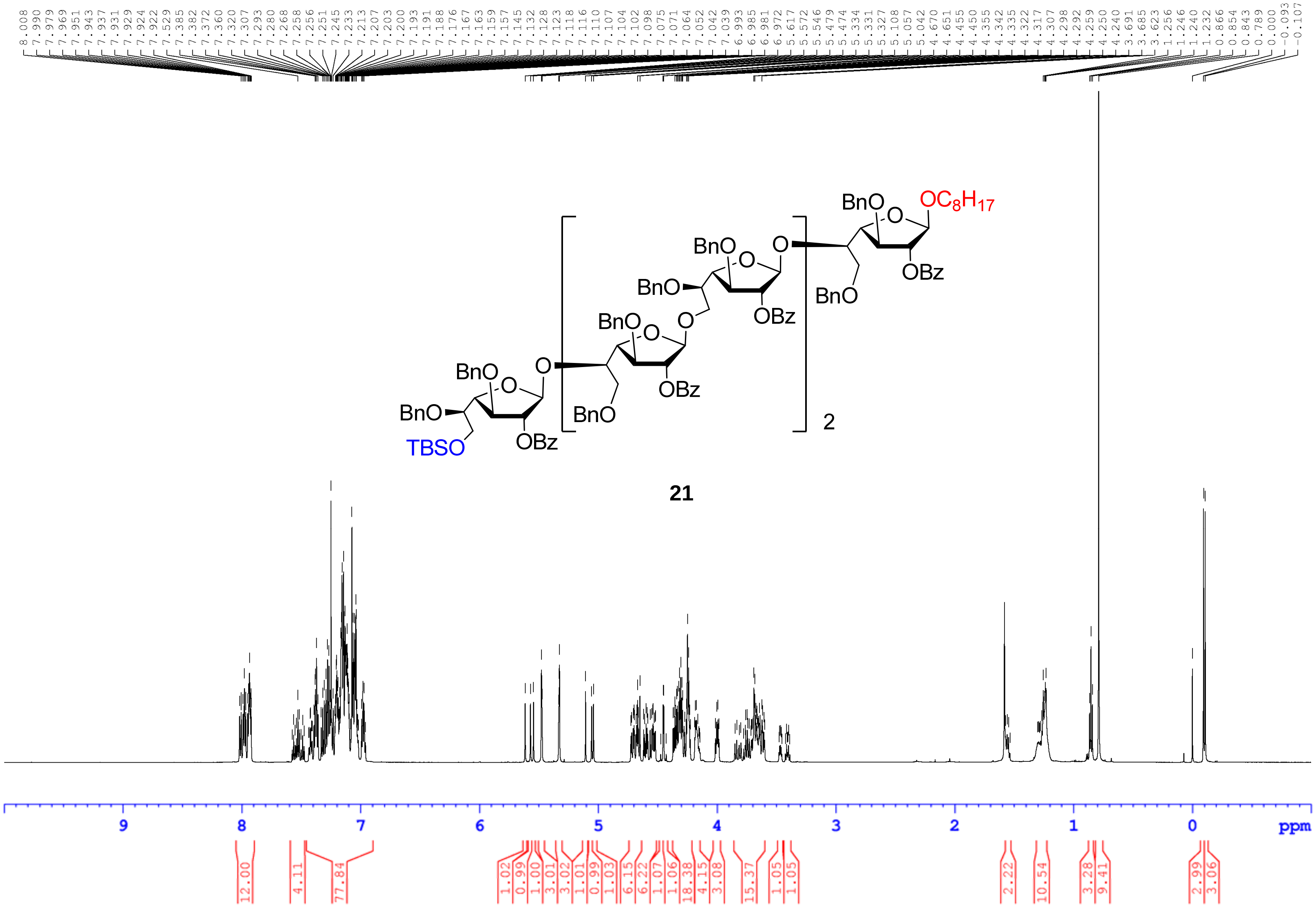

Supplementary Figure 32. ^{1}H NMR spectrum of compound 21

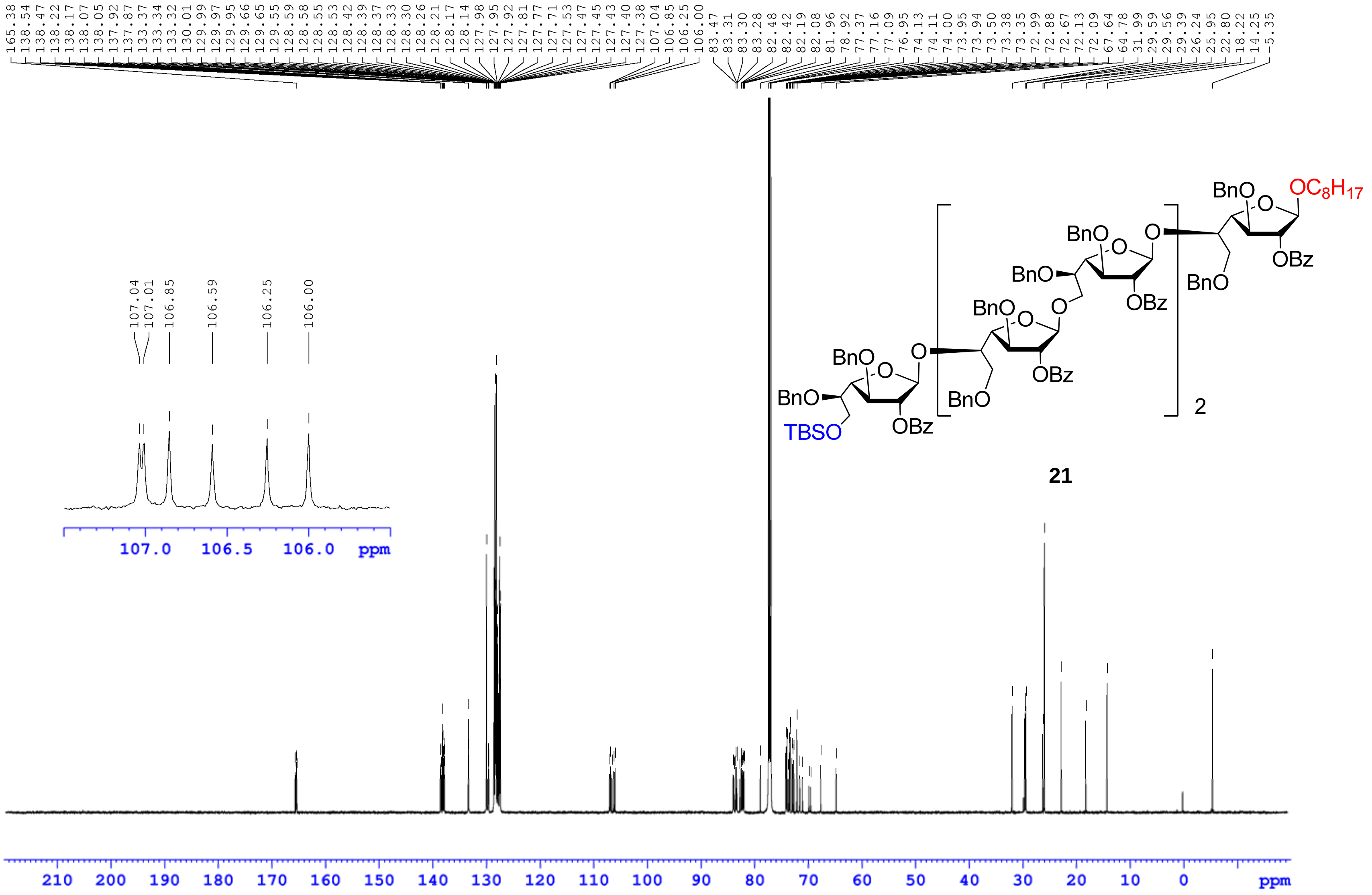

Supplementary Figure 33. ^{13}**C NMR spectrum of compound 21**

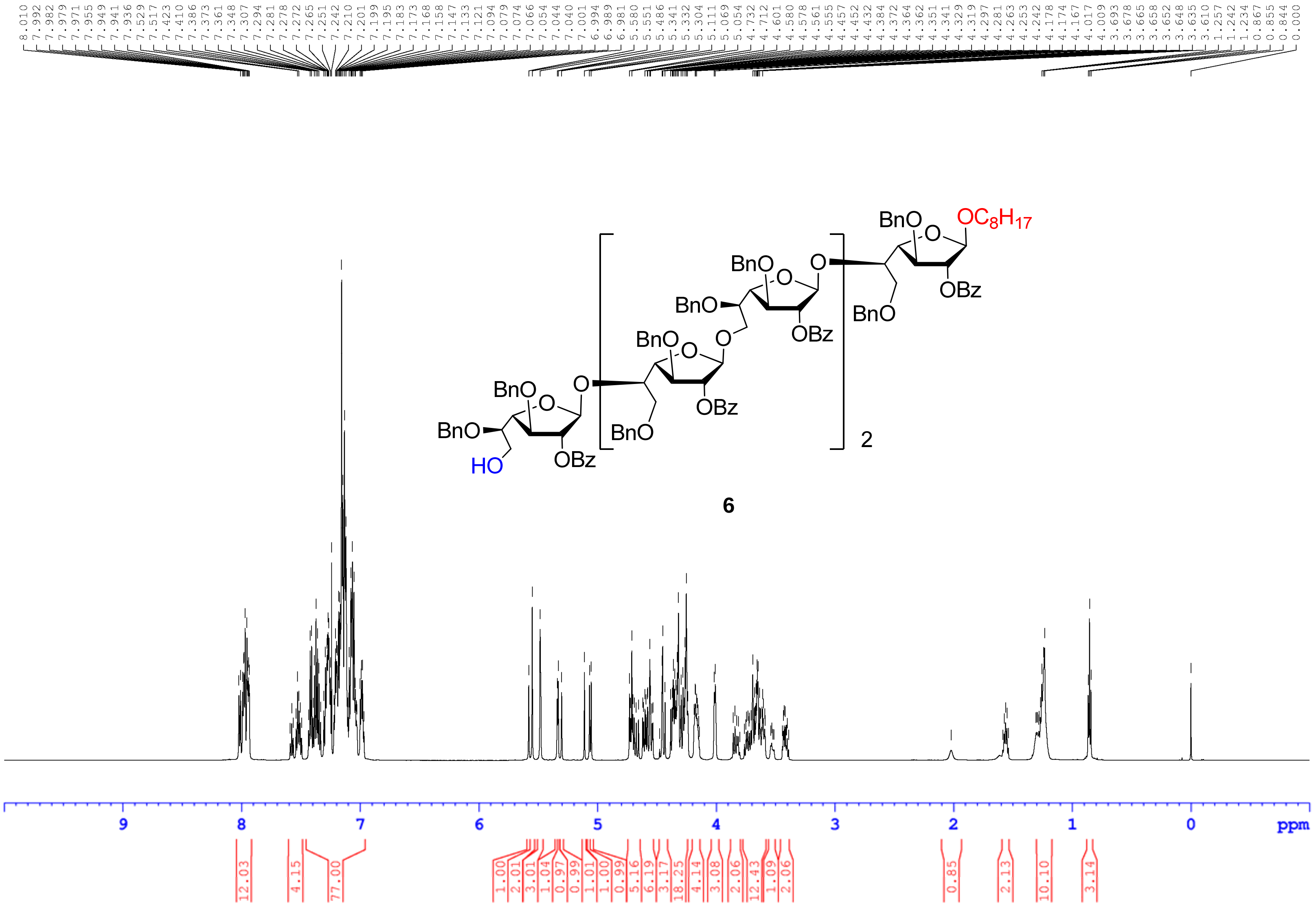

Supplementary Figure 34. ^{1}H NMR spectrum of compound 6

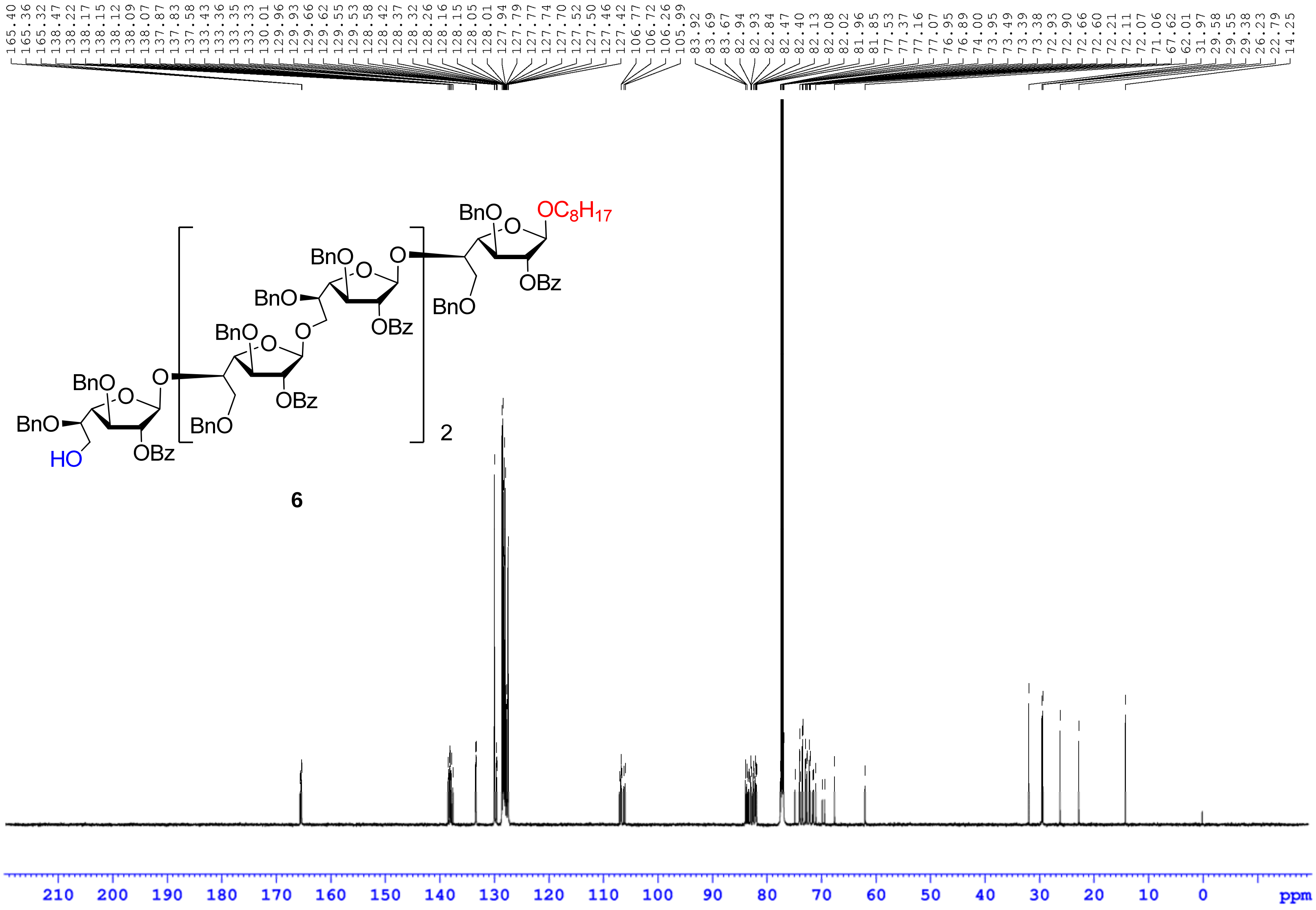

Supplementary Figure 35. ^{13}**C NMR spectrum of compound 6**

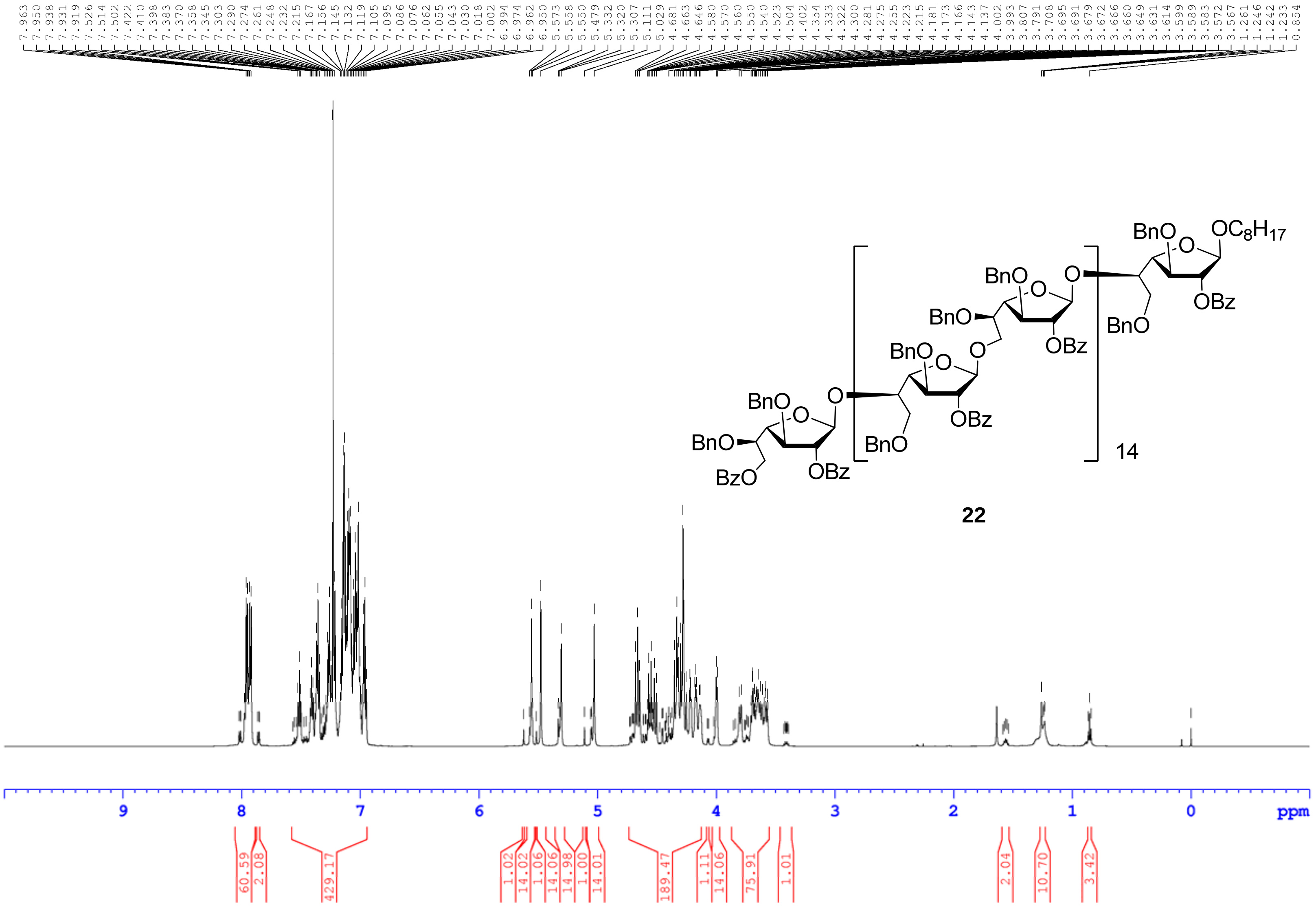

Supplementary Figure 36. ^{1}**H NMR spectrum of compound 22**

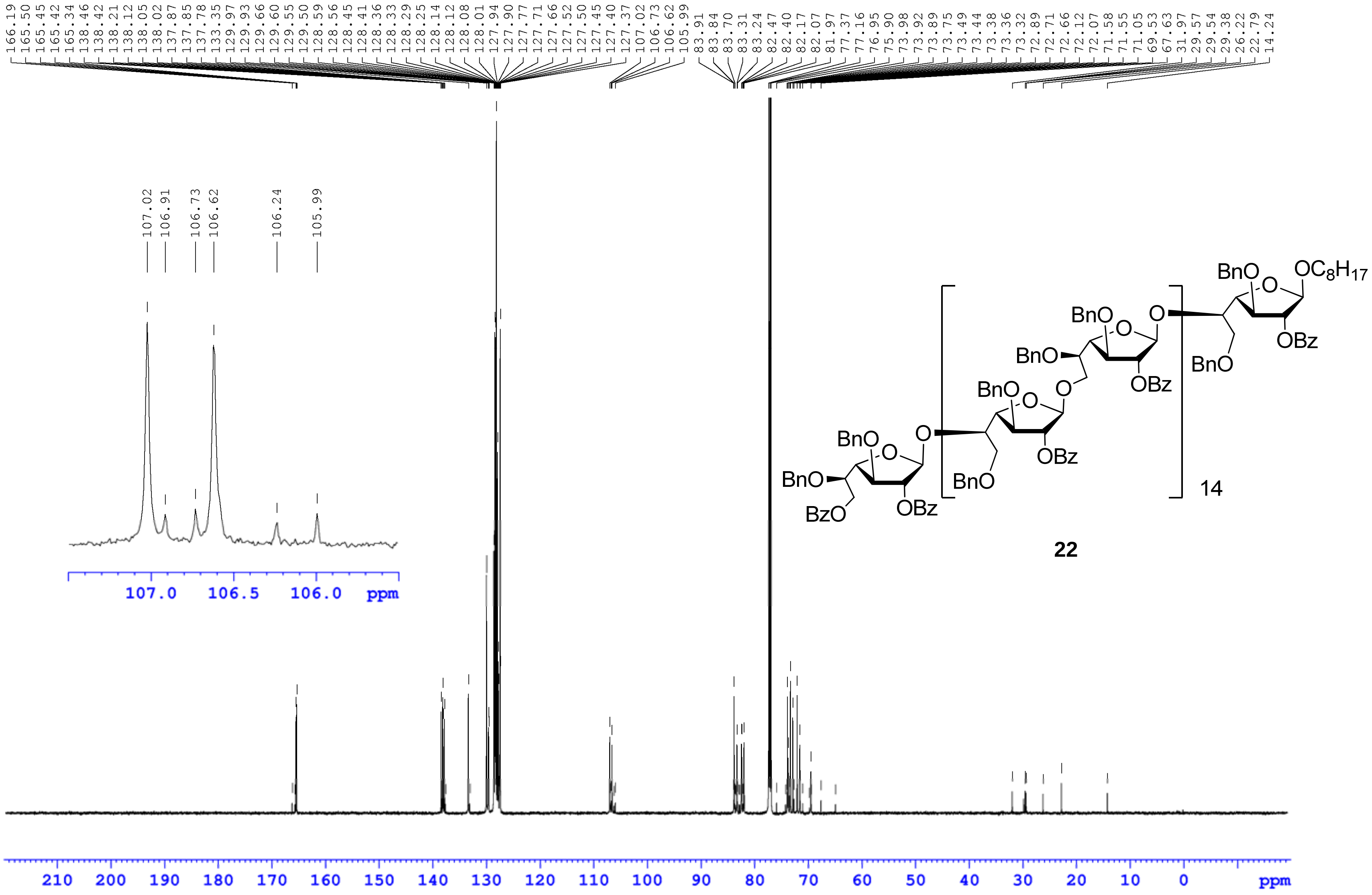

Supplementary Figure 37. ^{13}**C NMR spectrum of compound 22**

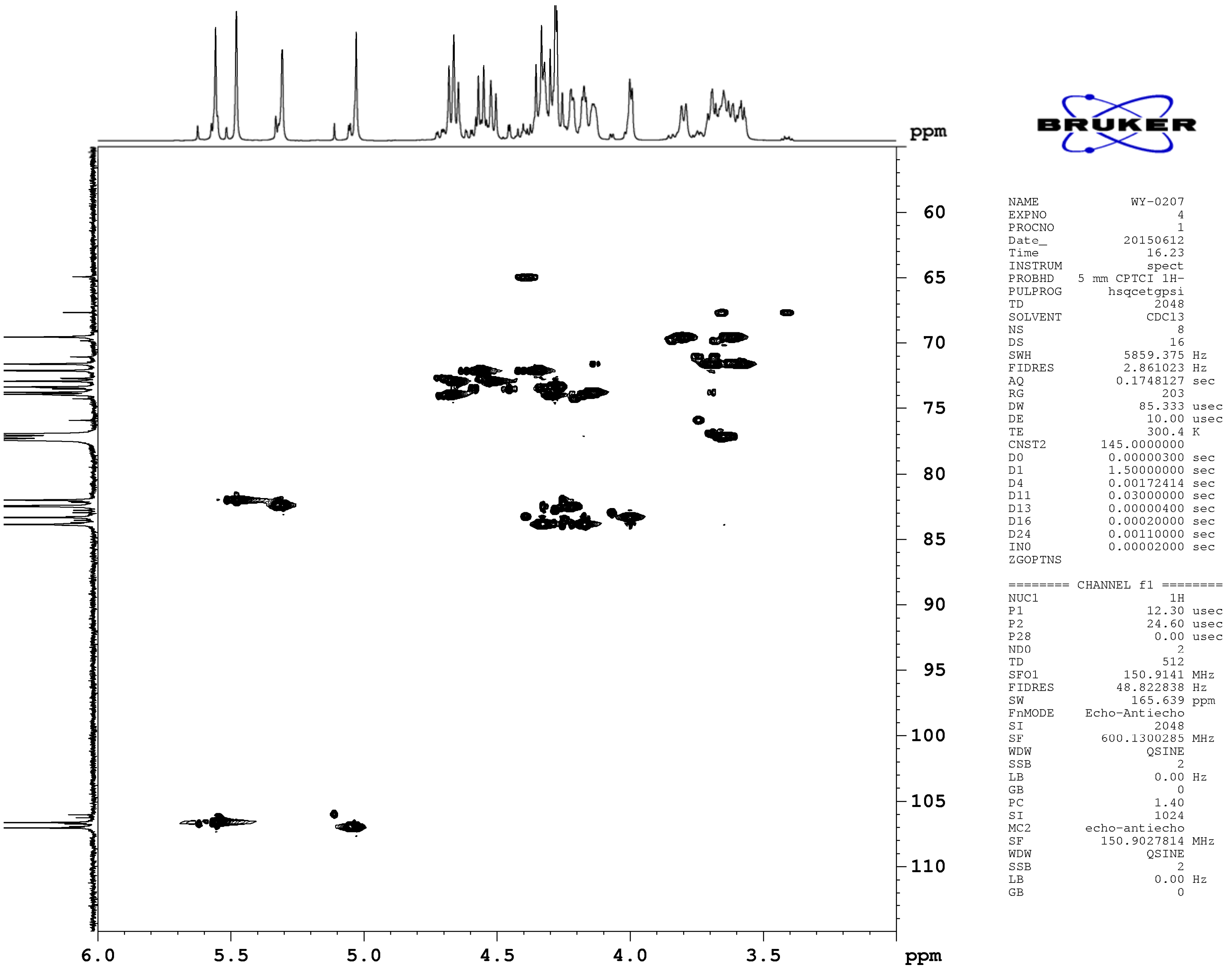

Supplementary Figure 38. HSQC NMR spectrum of compound 22

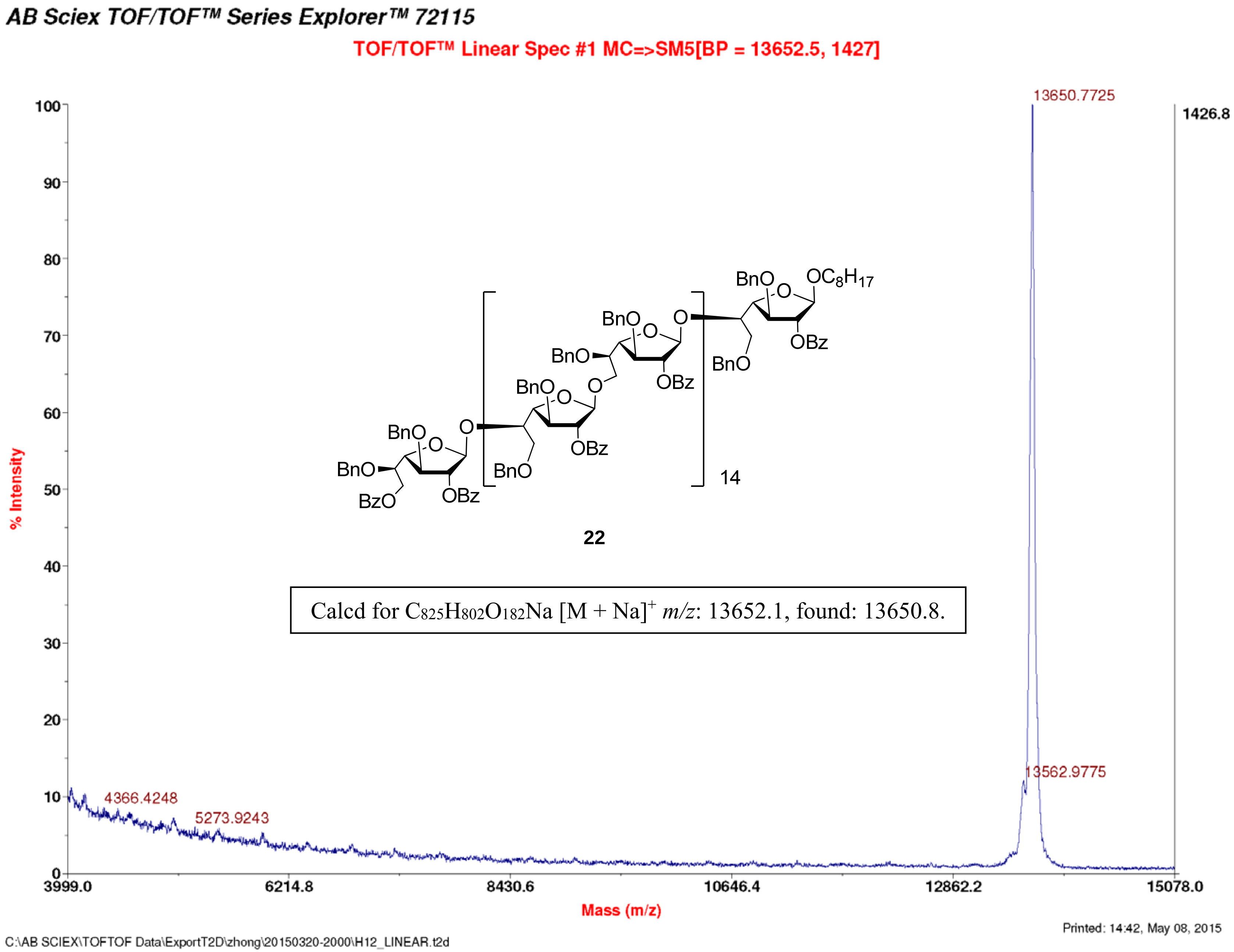

Supplementary Figure 39. MALDI-TOF MS spectrum of compound 22

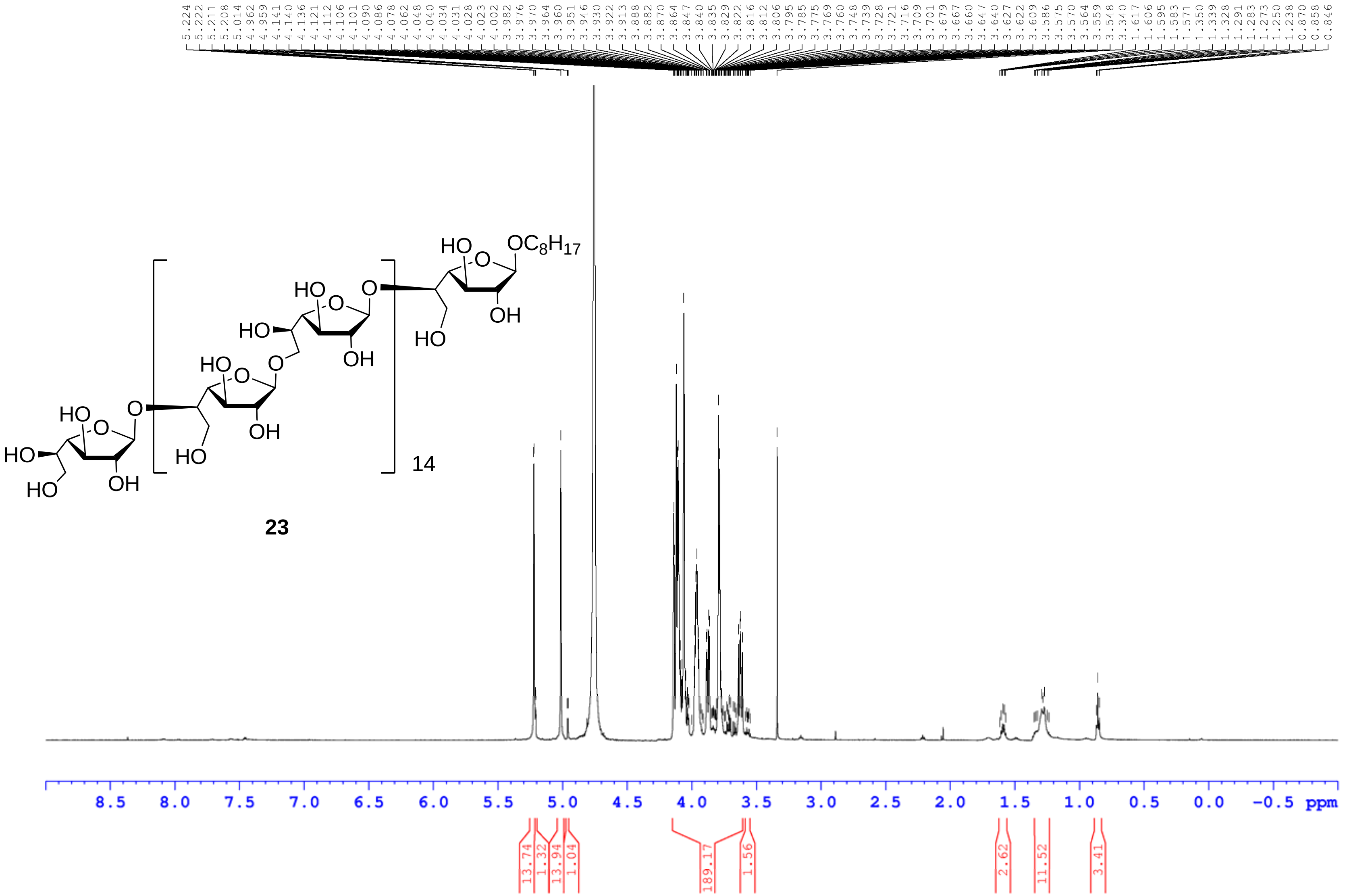

23

Supplementary Figure 40. ^{1}**H NMR spectrum of compound 23**

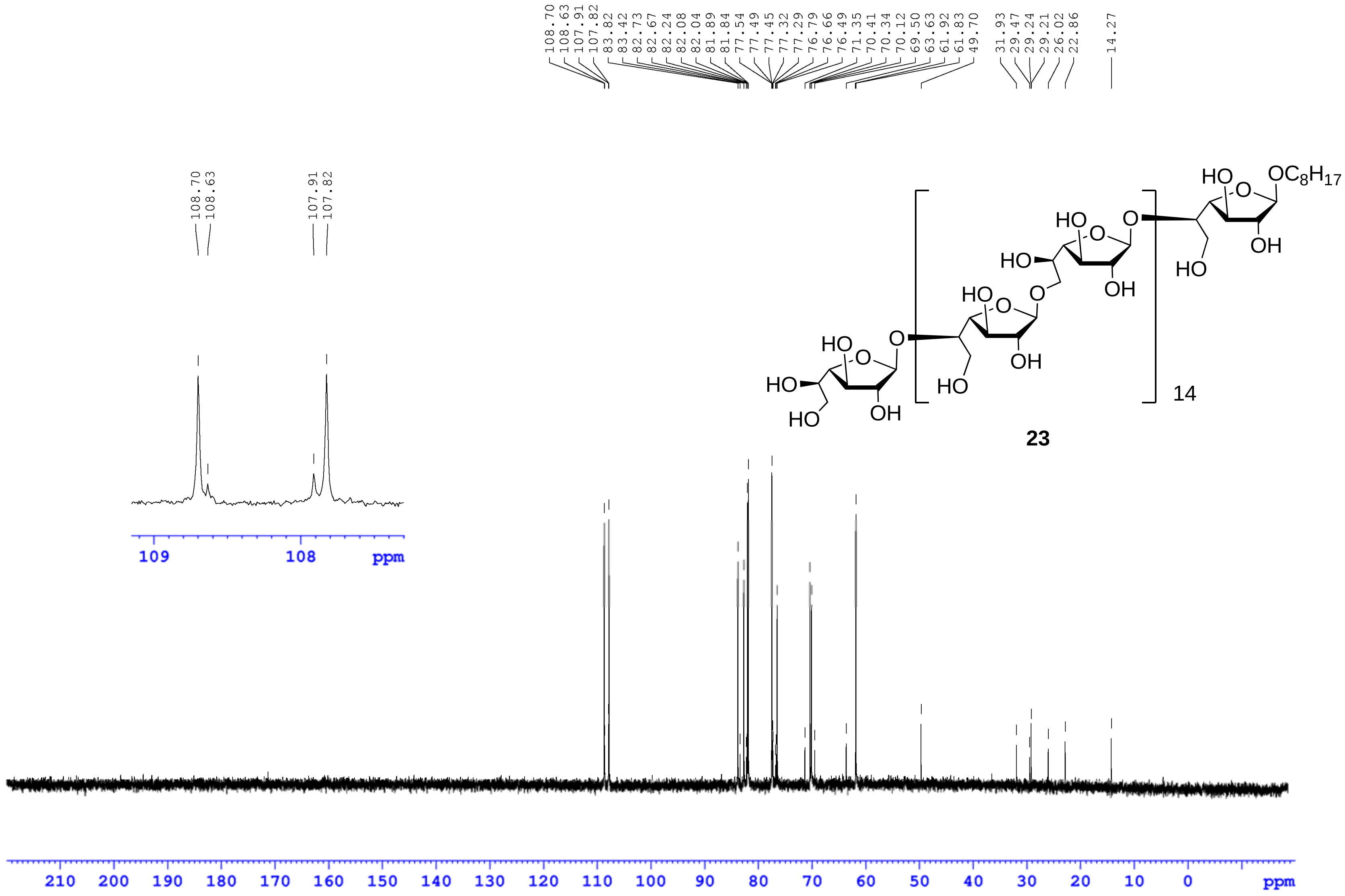

Supplementary Figure 41. ^{13}C NMR spectrum of compound 23

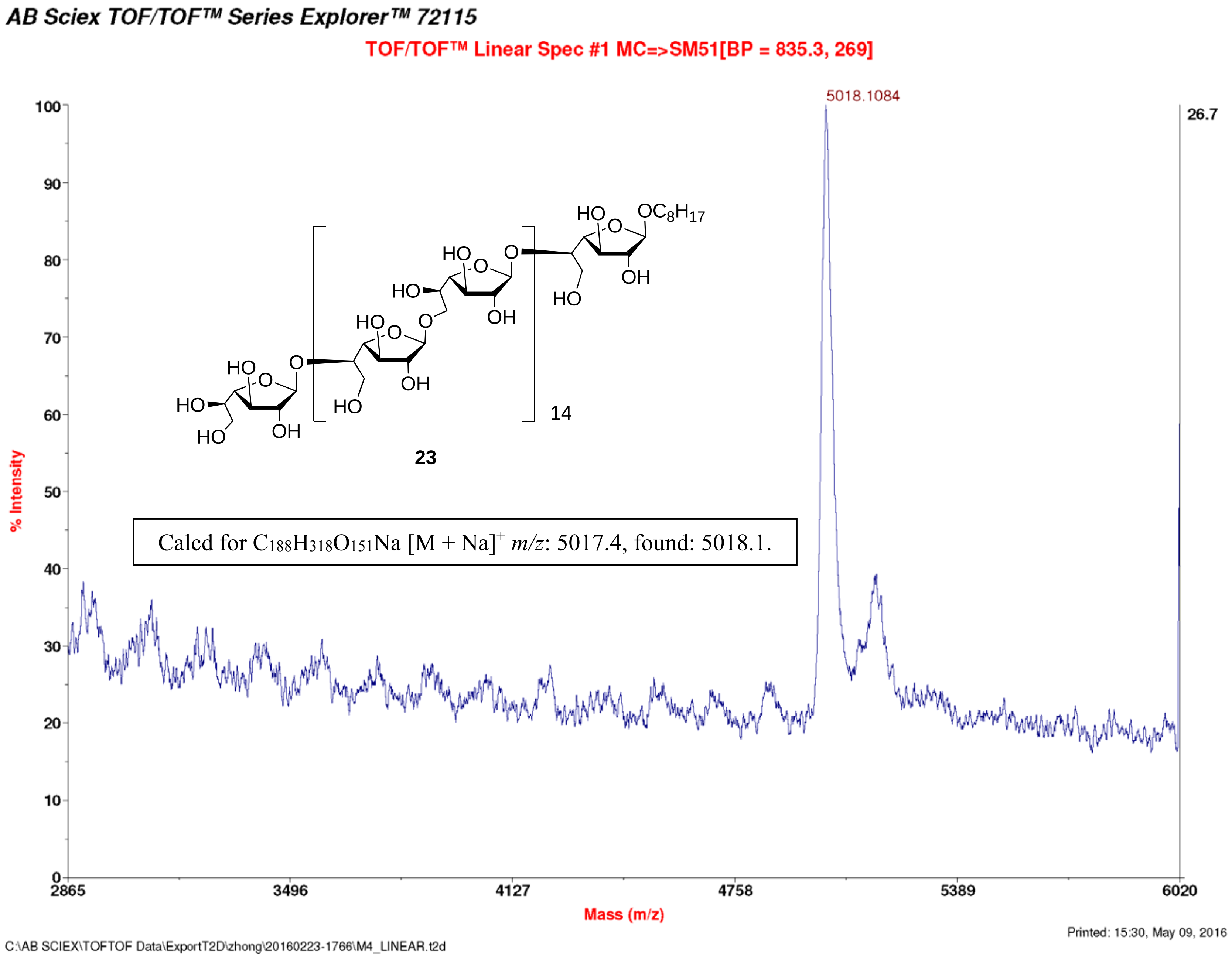

Supplementary Figure 42. MALDI-TOF MS spectrum of compound 23

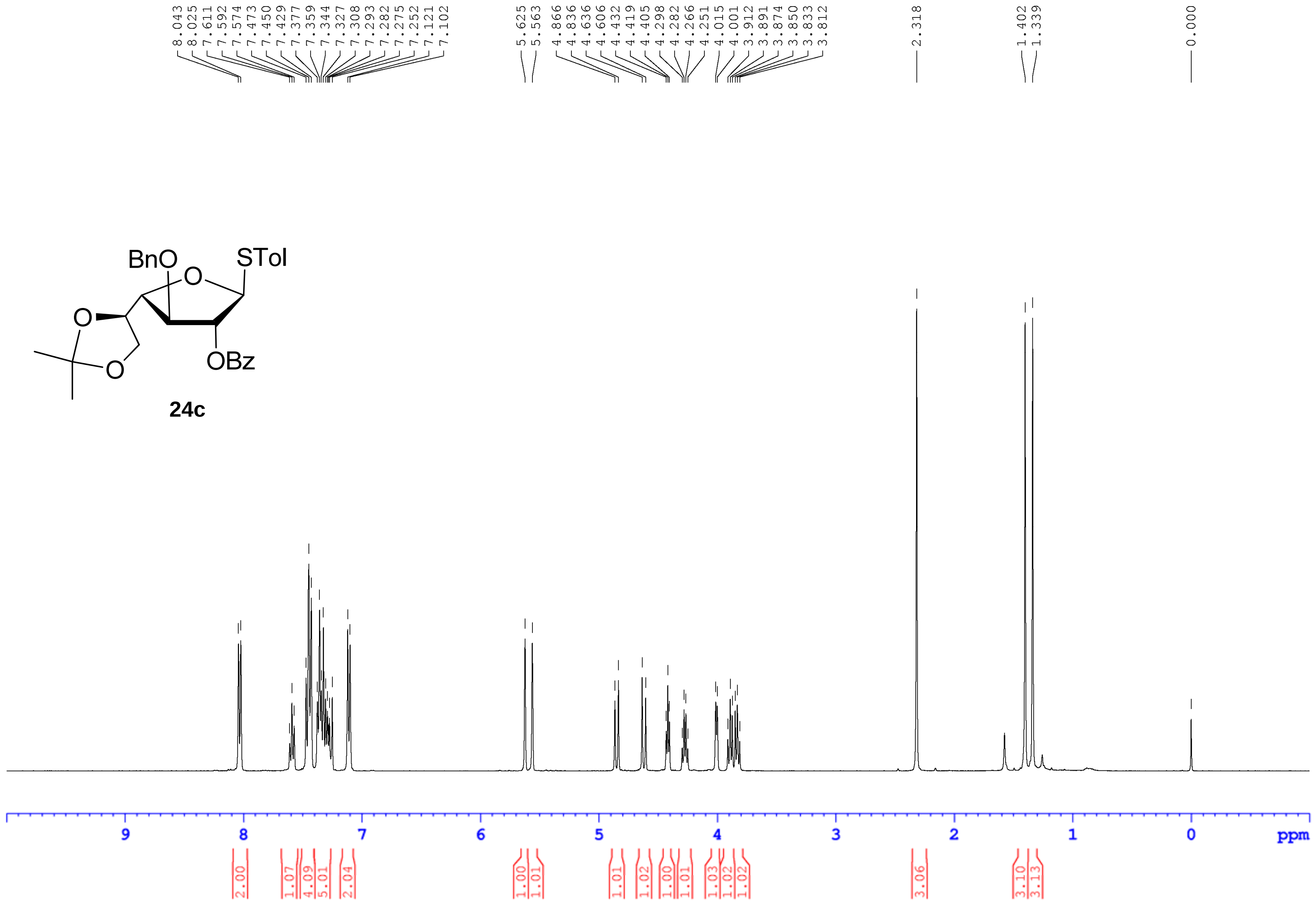

Supplementary Figure 43. ^{1}H NMR spectrum of compound 24c

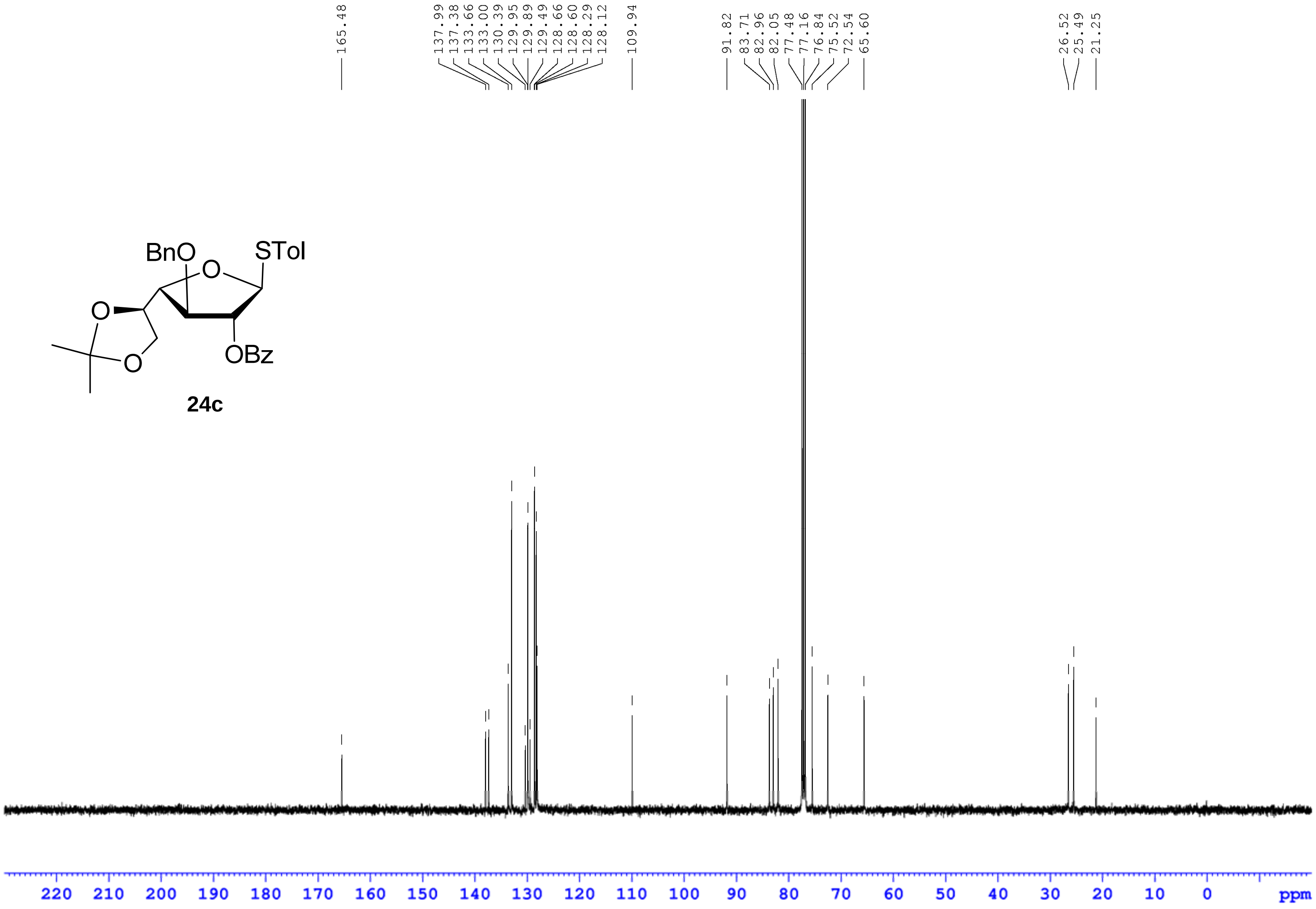

Supplementary Figure 44. ^{13}C NMR spectrum of compound 24c

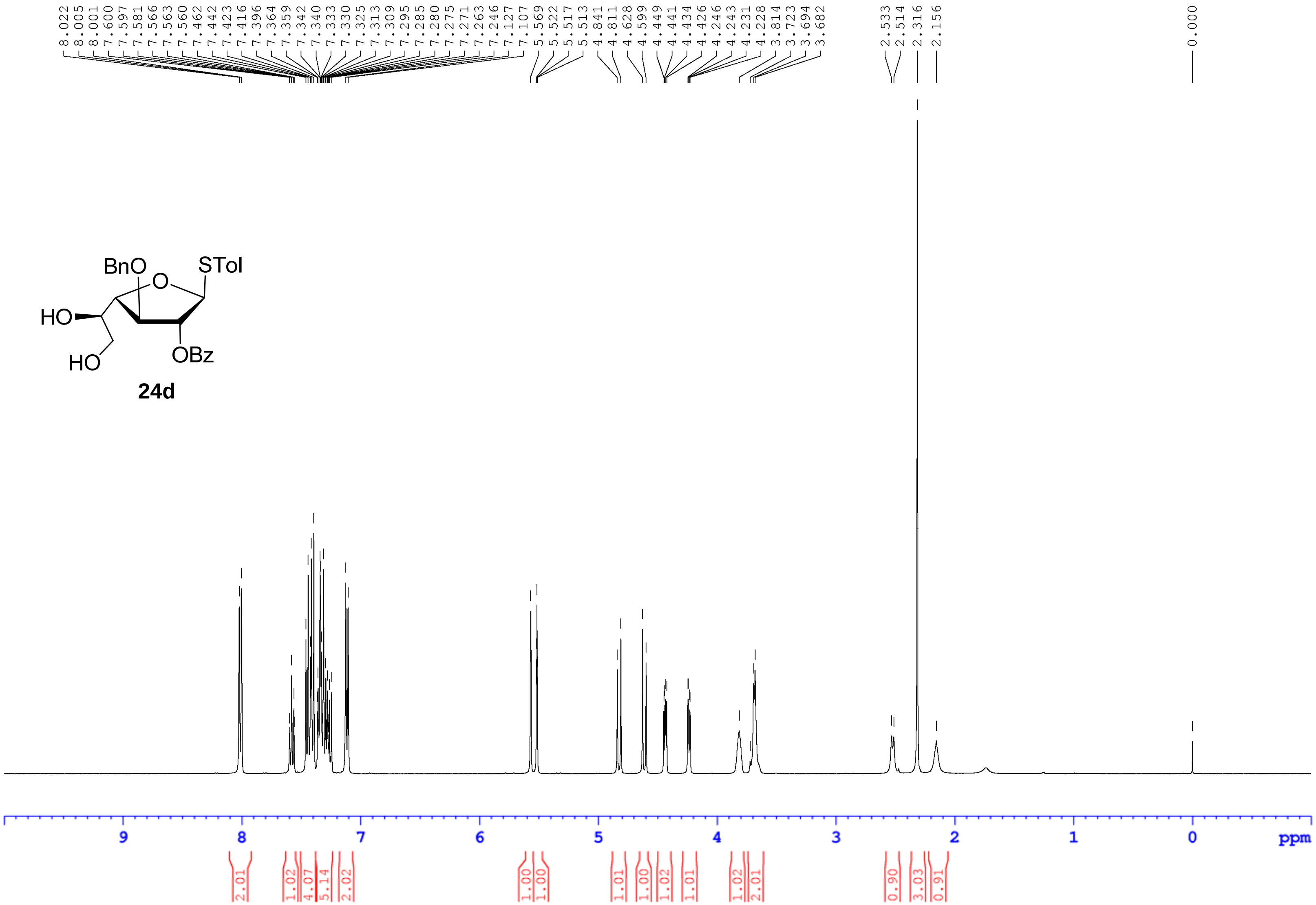

Supplementary Figure 45. ¹H NMR spectrum of compound 24d

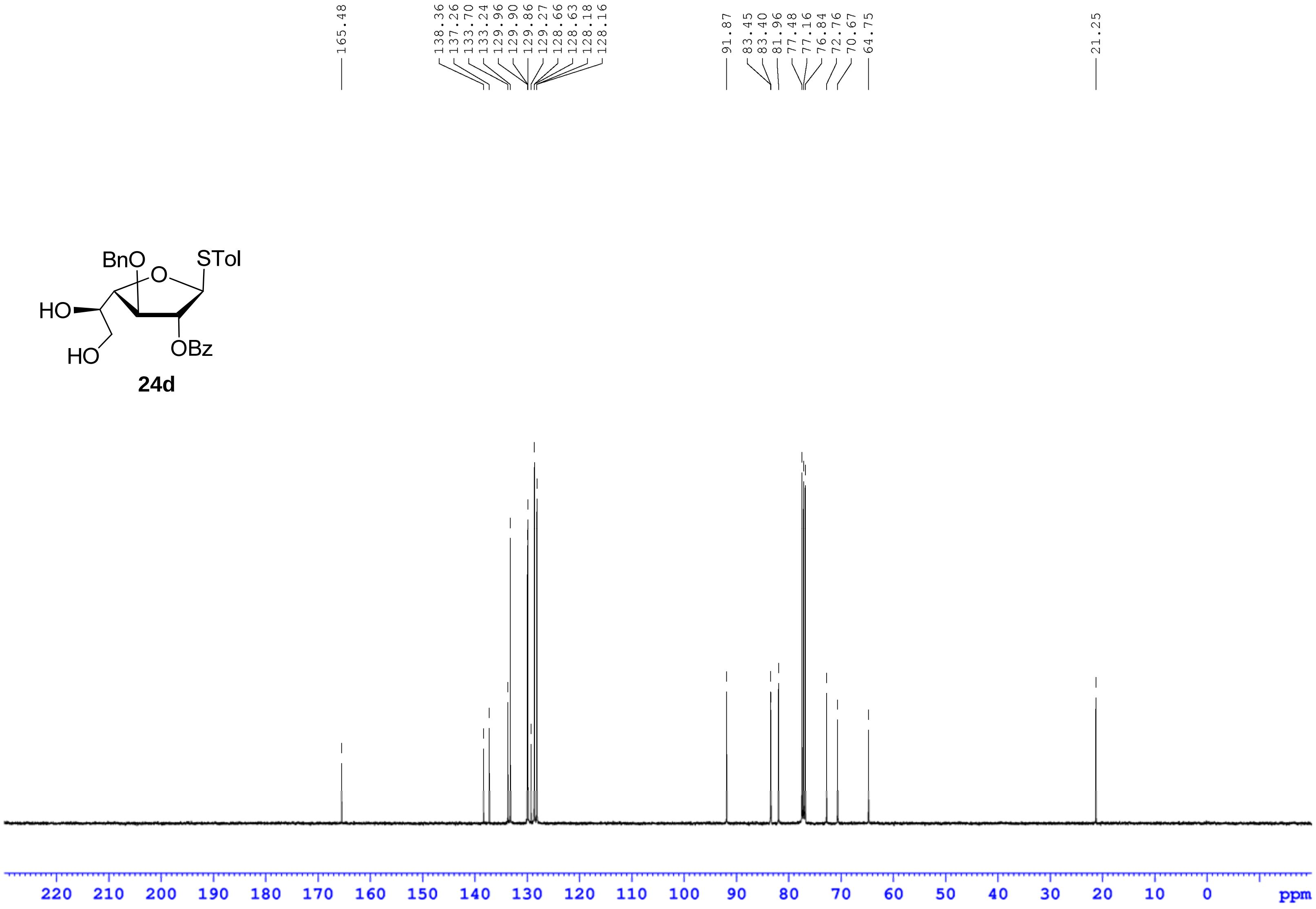

Supplementary Figure 46. ^{13}C NMR spectrum of compound 24d

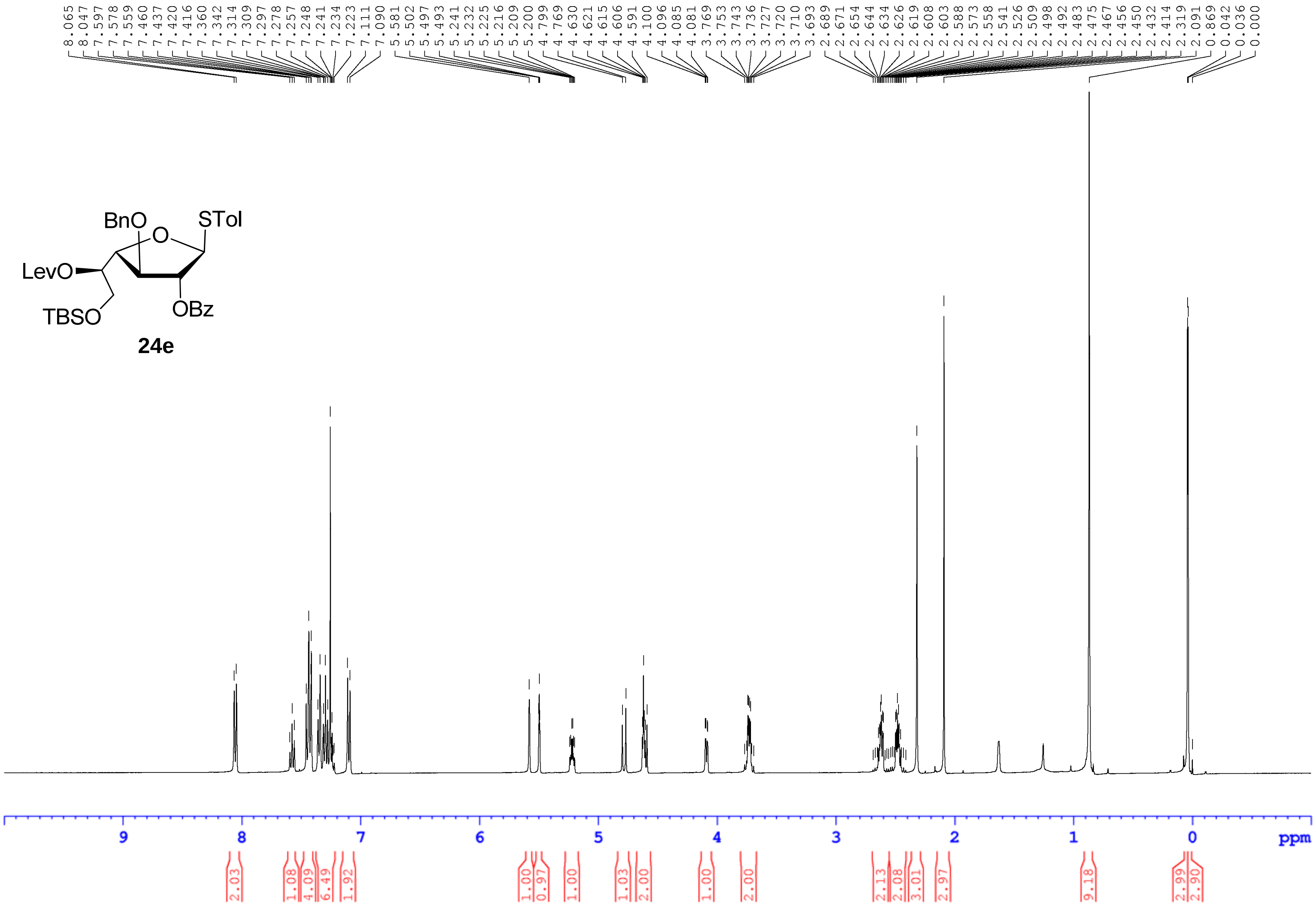

24e

Supplementary Figure 47. ^{1}H NMR spectrum of compound 24e

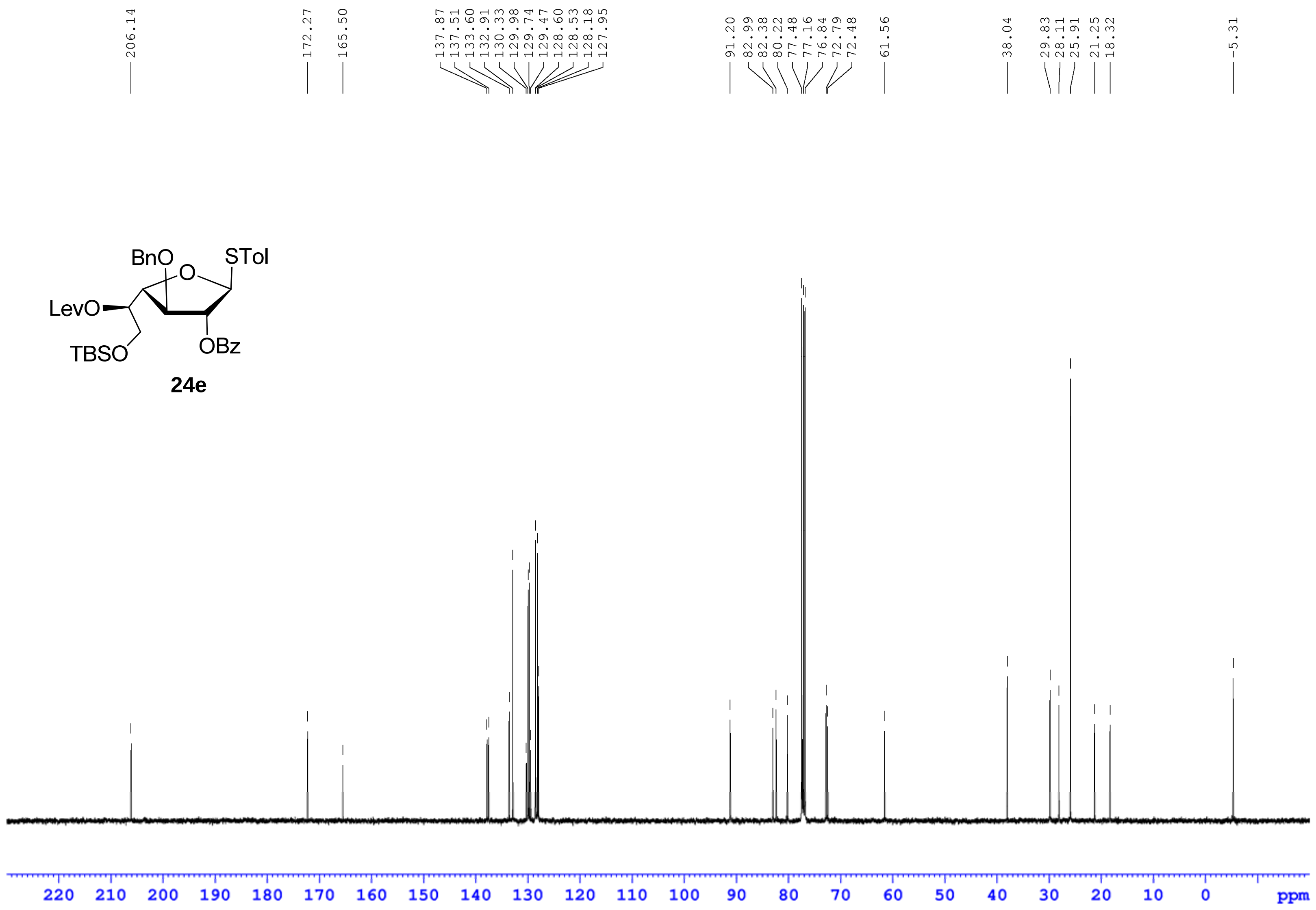

Supplementary Figure 48. ^{13}C **NMR spectrum of compound 24e**

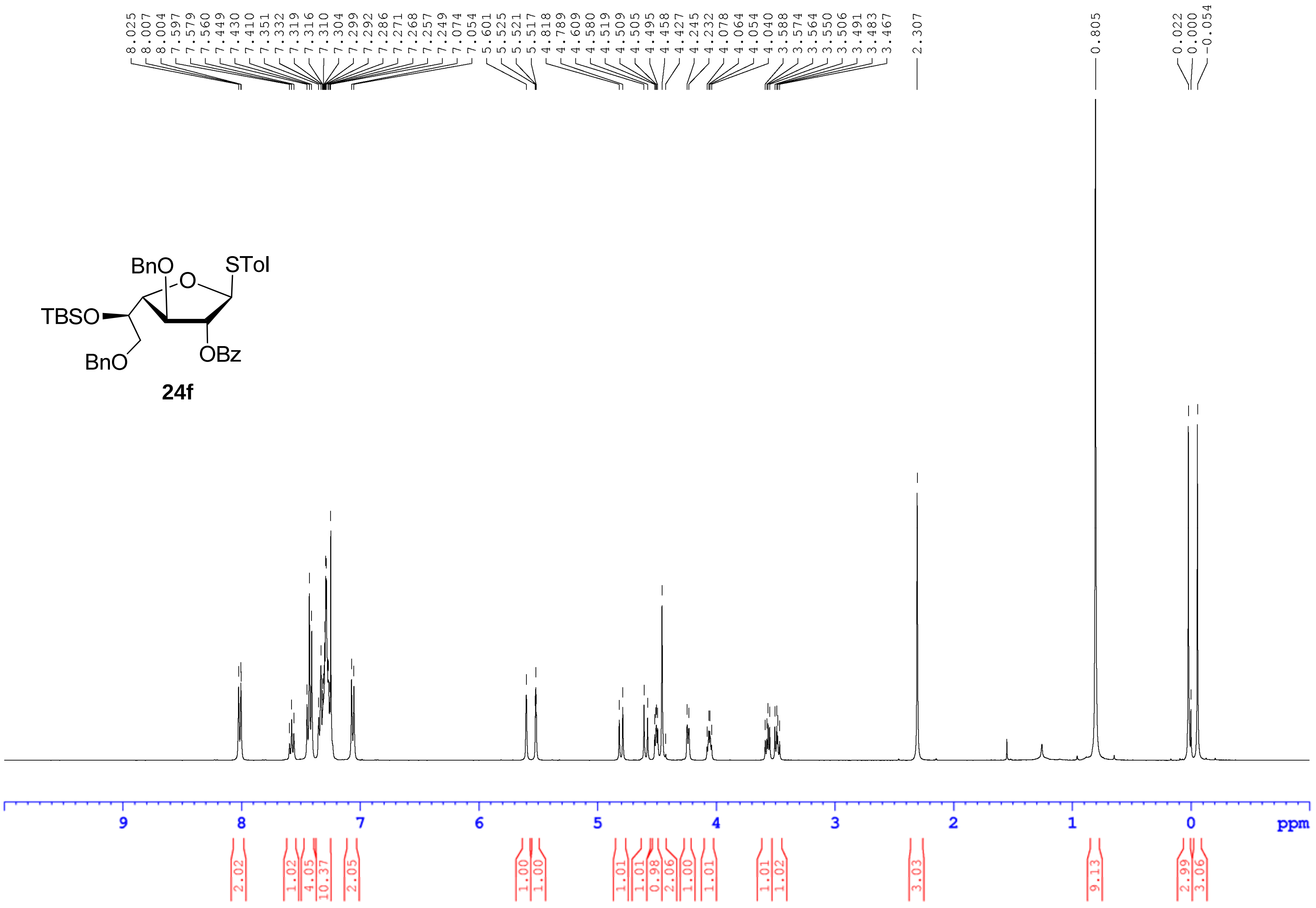

Supplementary Figure 49. ^{1}H NMR spectrum of compound 24f

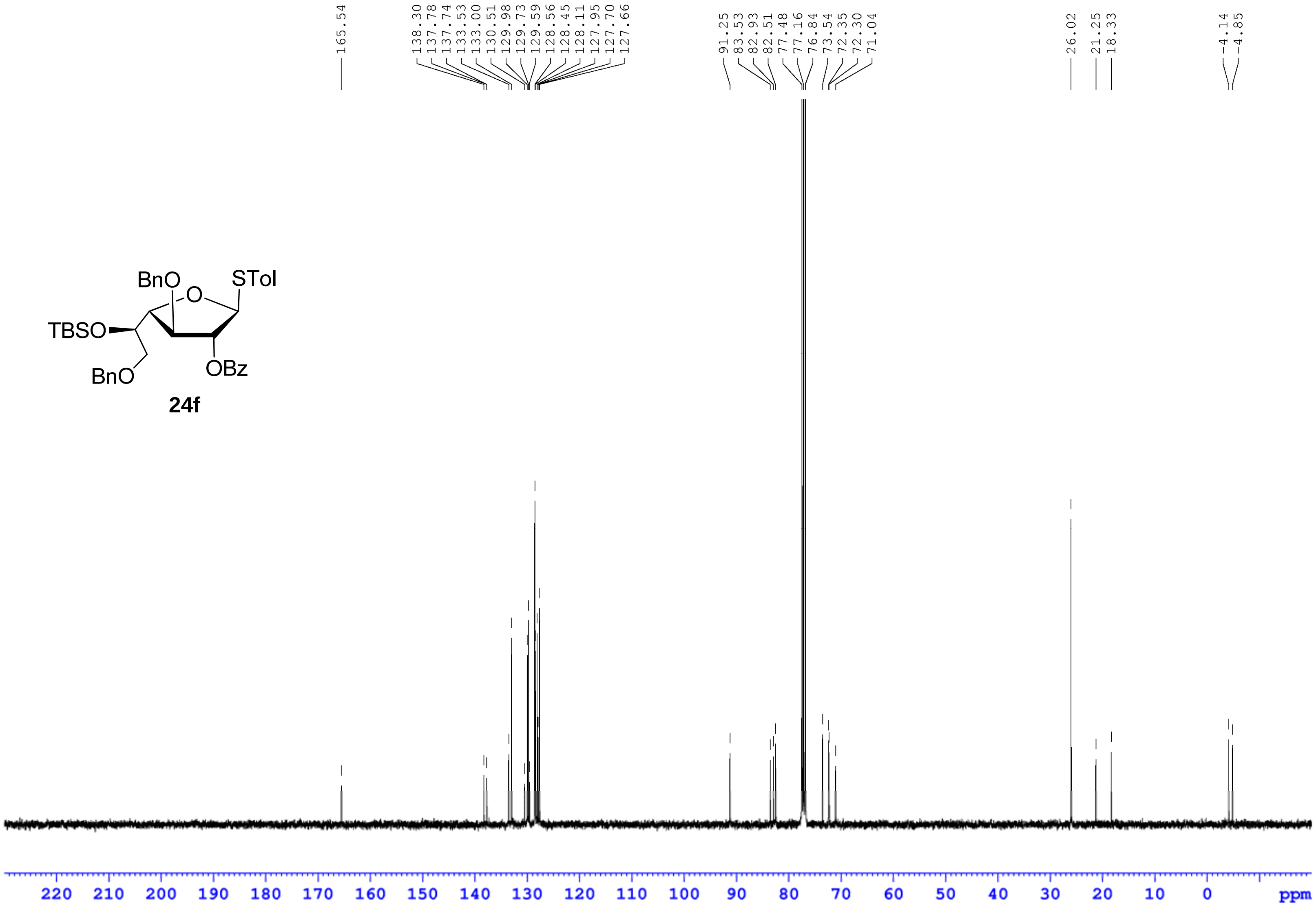

Supplementary Figure 50. ^{13}**C NMR spectrum of compound 24f**

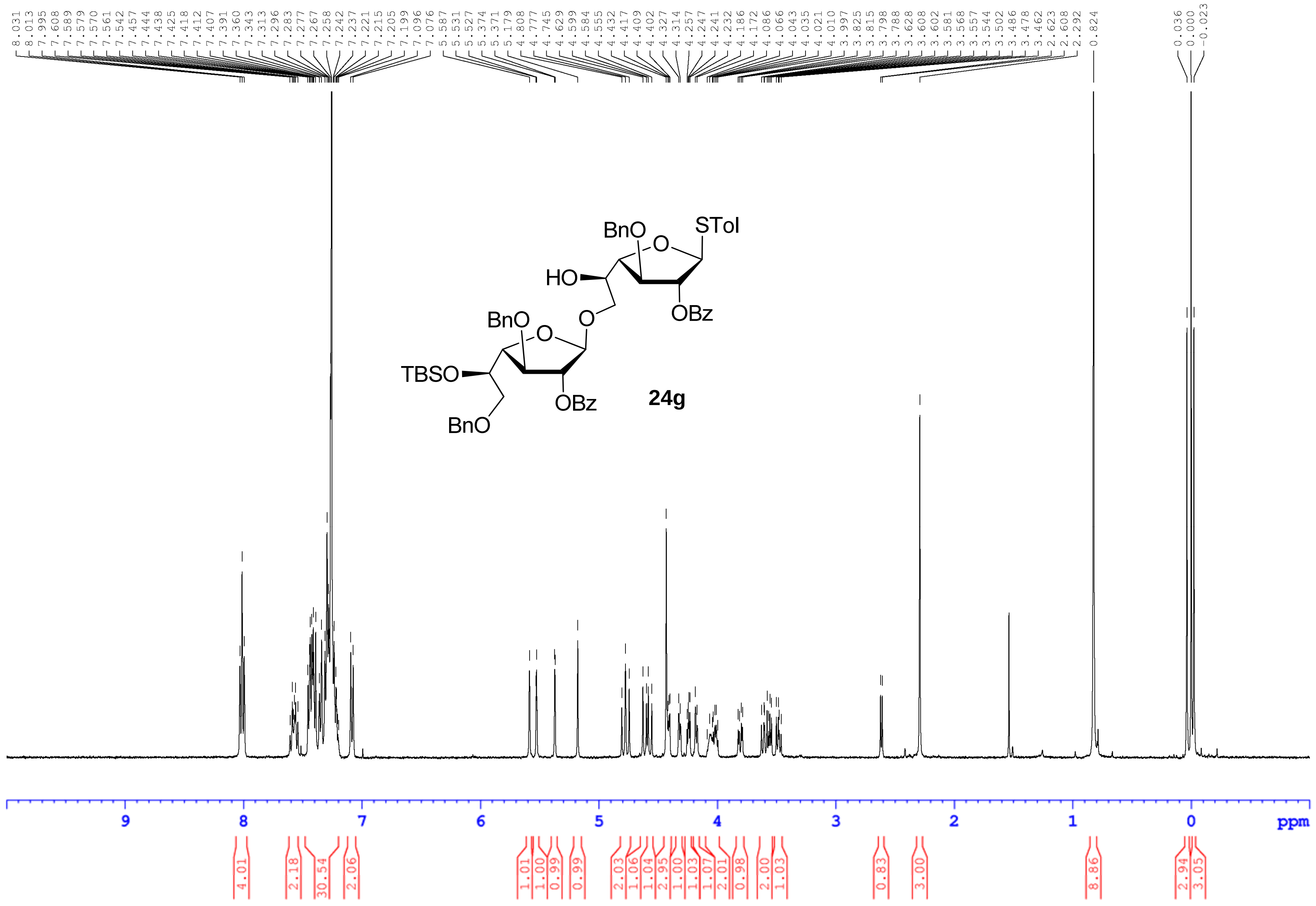

Supplementary Figure 51. ^{1}H NMR spectrum of compound 24g

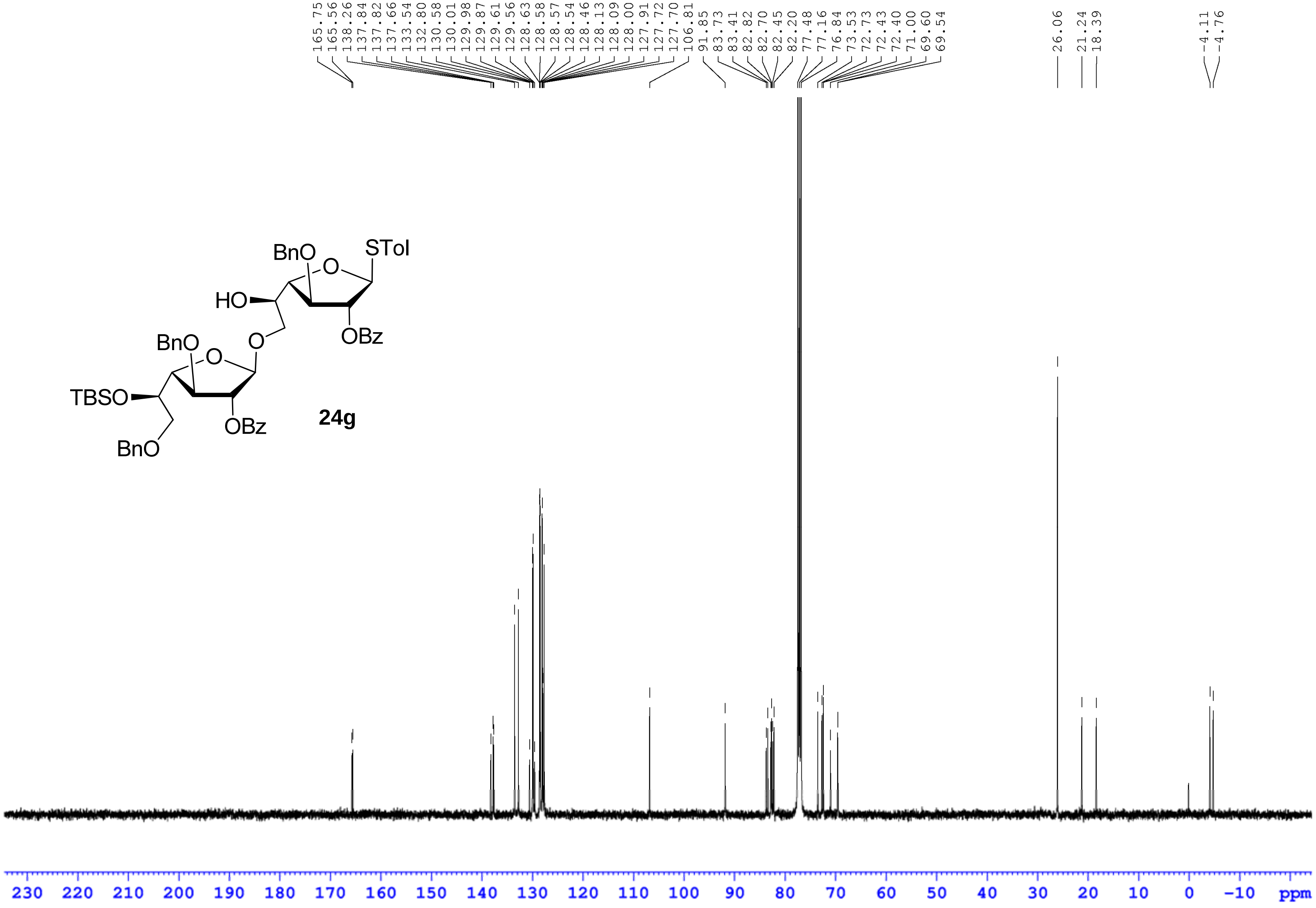

Supplementary Figure 52. ^{13}**C NMR spectrum of compound 24g**

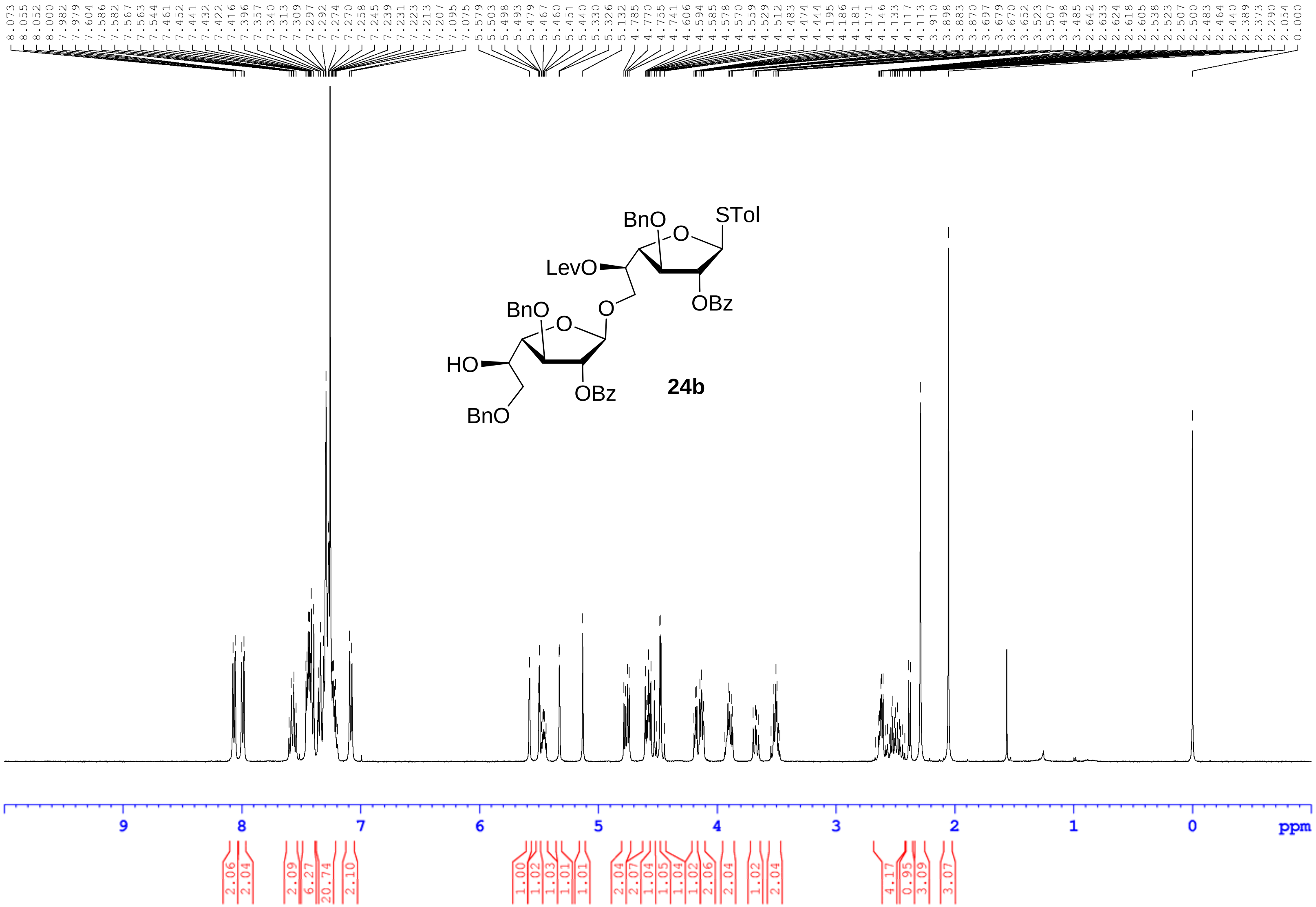

Supplementary Figure 53. ^{1}H NMR spectrum of compound 24b

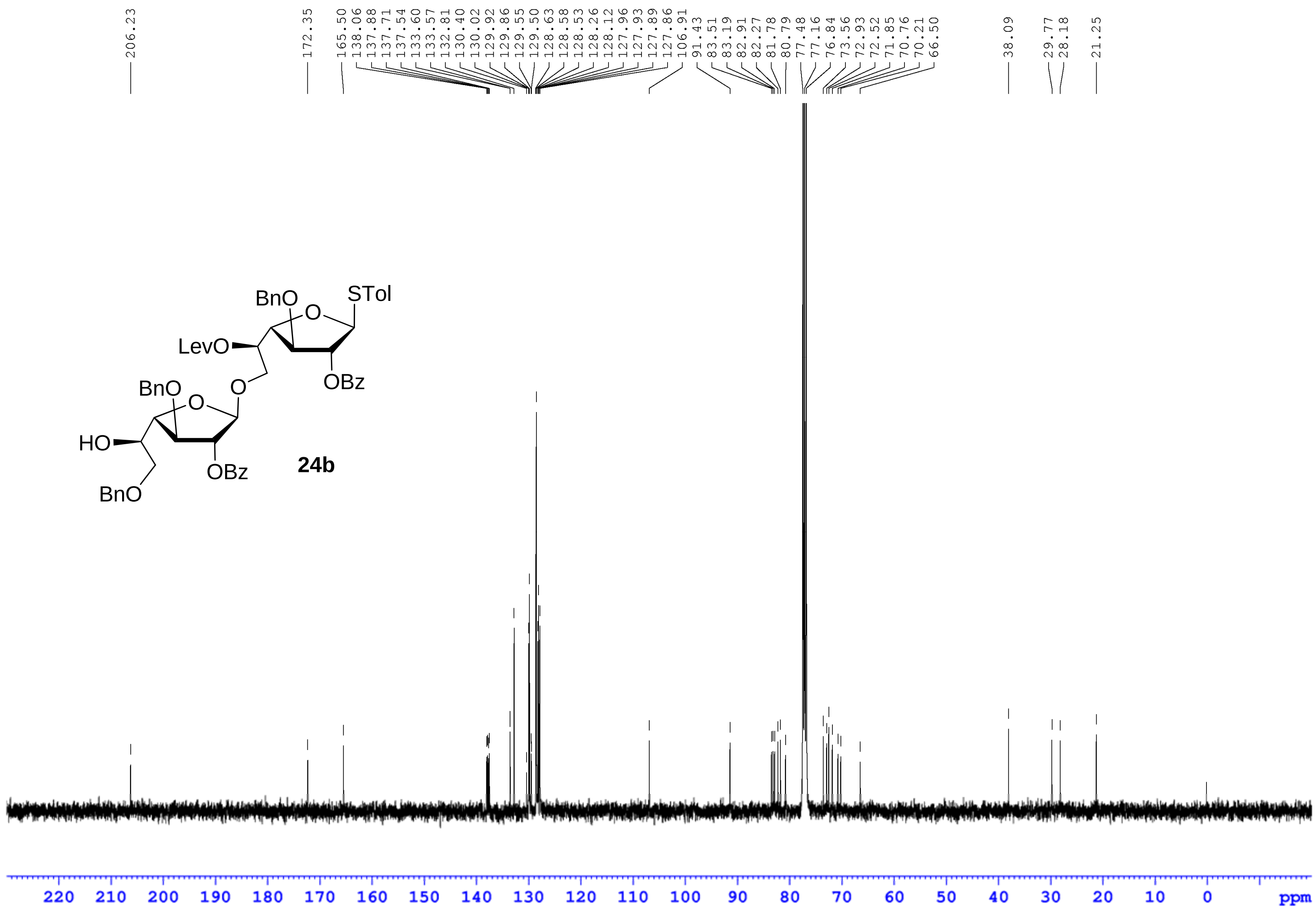

Supplementary Figure 54. ^{13}C NMR spectrum of compound 24b

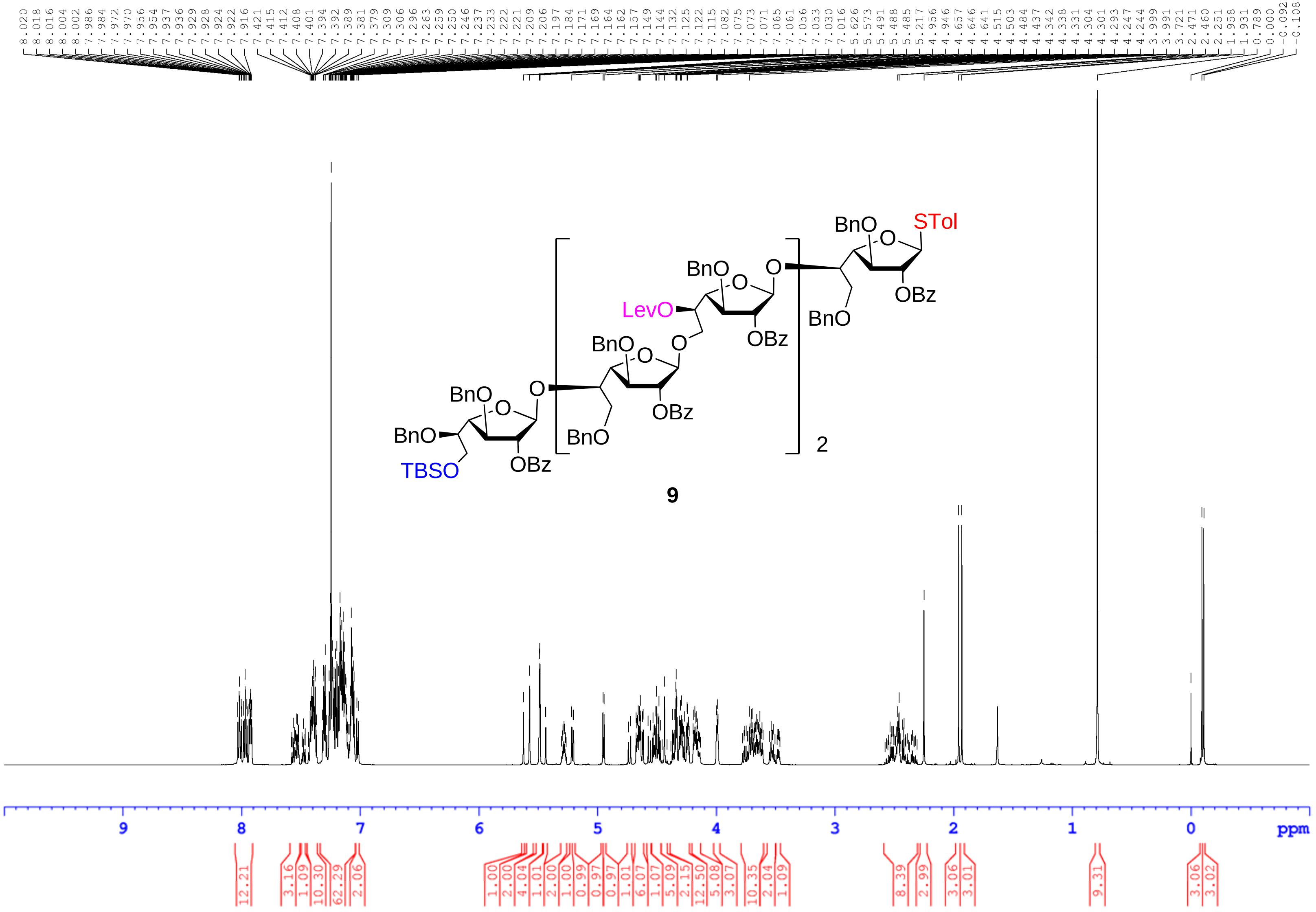

Supplementary Figure 55. ^{1}H NMR spectrum of compound 9

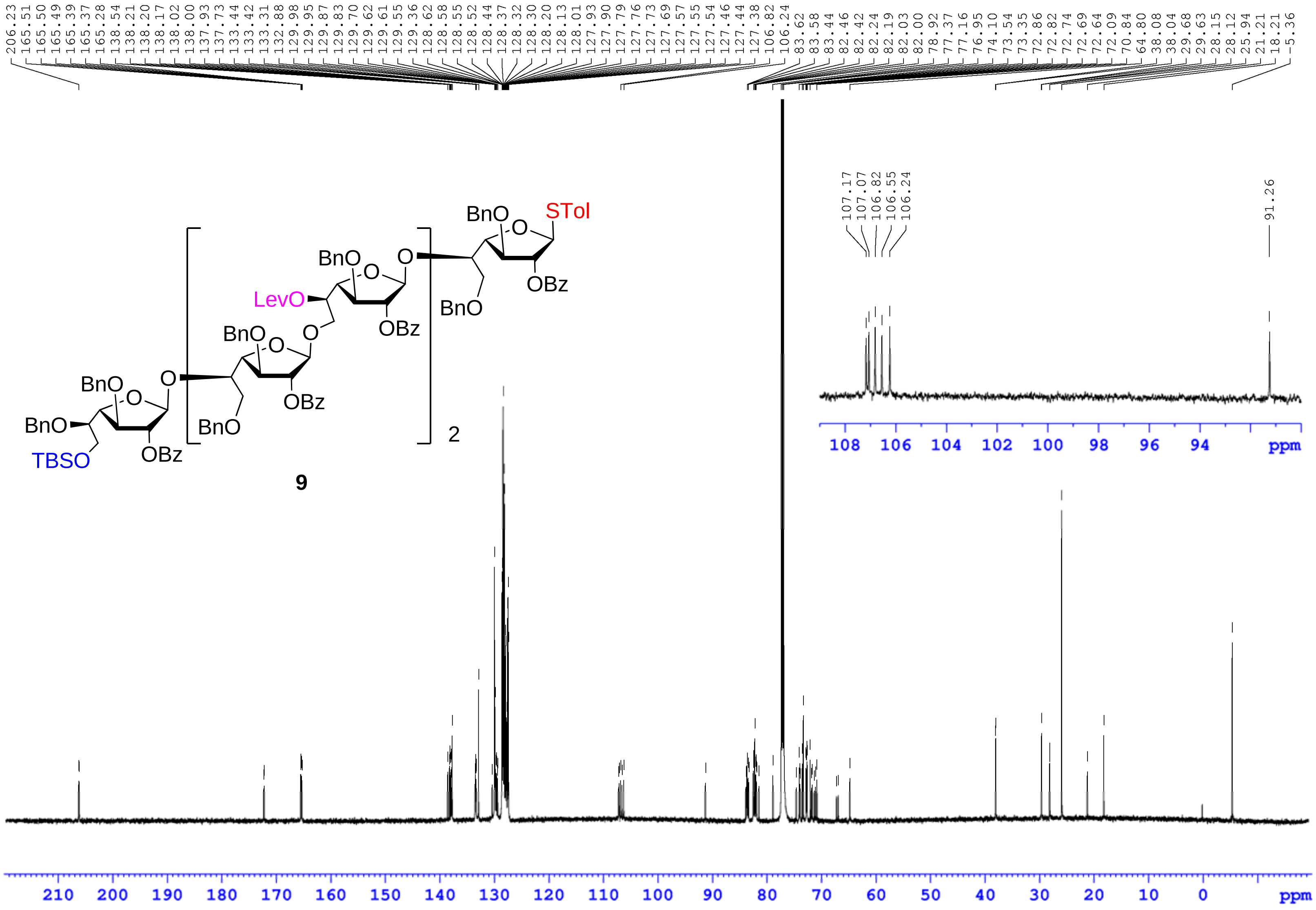

Supplementary Figure 56. ^{13}**C NMR spectrum of compound 9**

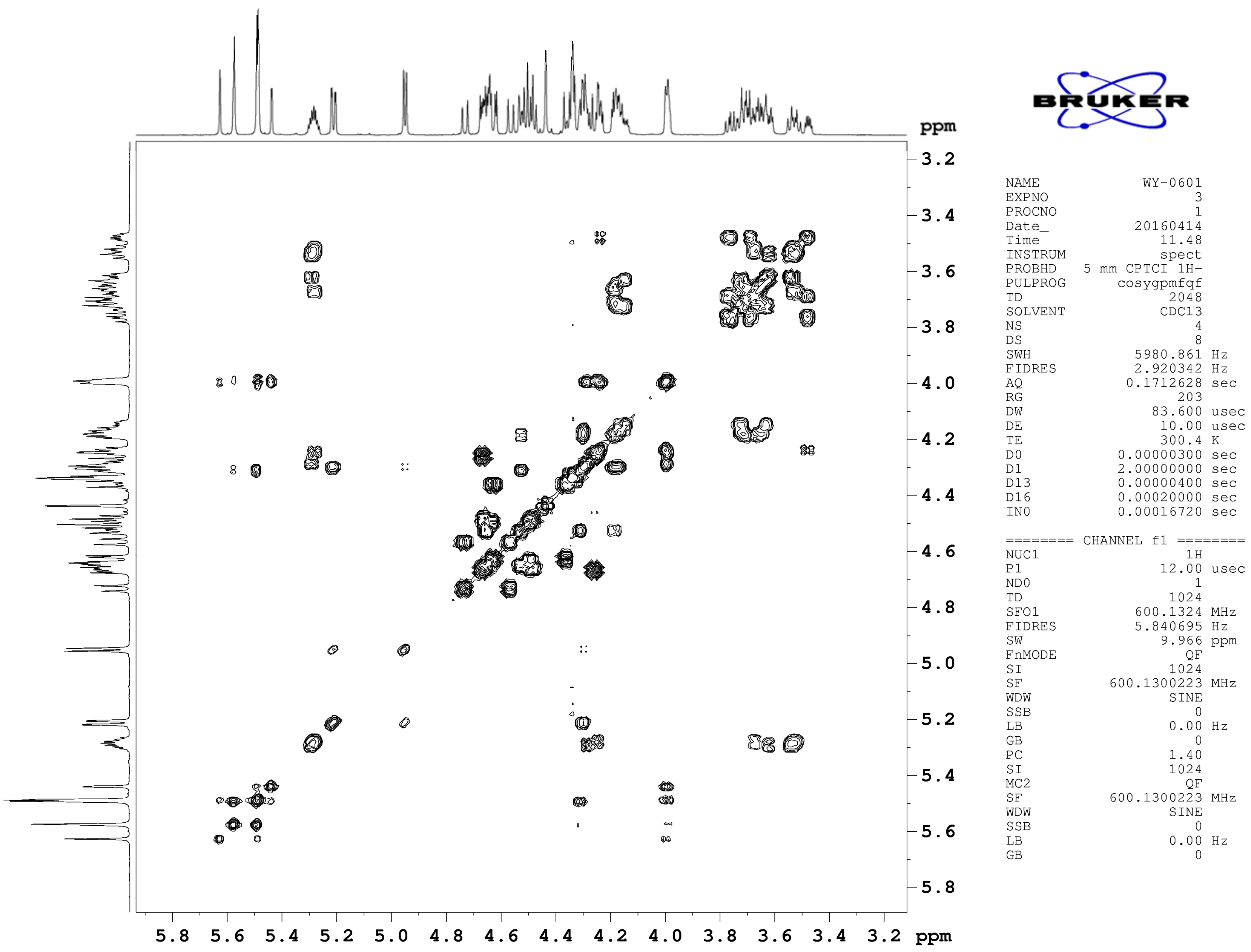

Supplementary Figure 57. COSY NMR spectrum of compound 9

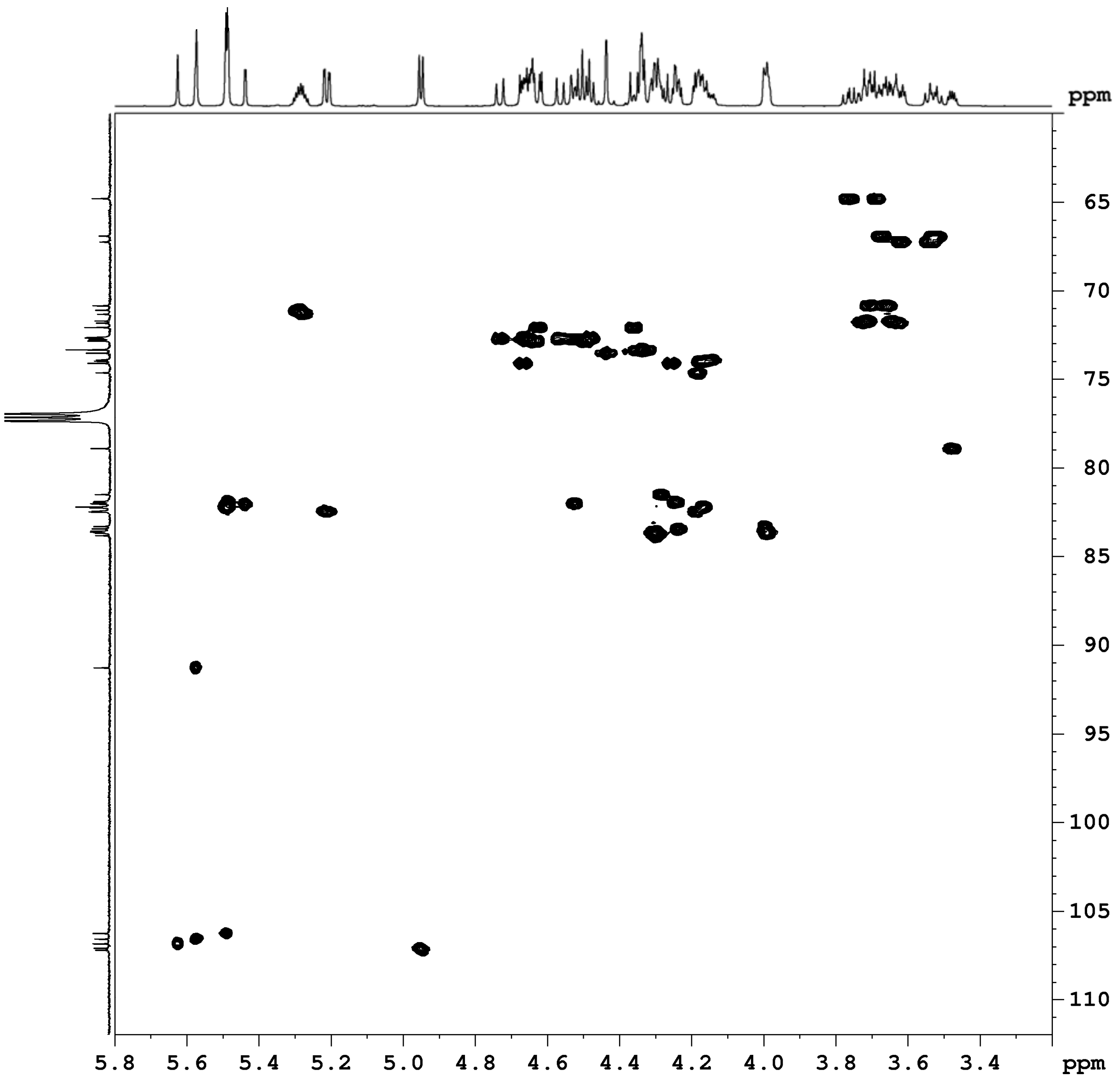

NAME	WY-0601	
EXPNO	4	
PROCNO	1	
Date_	20160415	
Time	16.17	
INSTRUM	spect	
PROBHD	5 mm CPTCI 1H-	
PULPROG	hsqcetgp	
TD	2048	
SOLVENT	CDCl3	
NS	8	
DS	16	
SWH	5980.861	Hz
FIDRES	2.920342	Hz
AQ	0.1712628	sec
RG	203	
DW	83.600	usec
DE	10.00	usec
TE	300.4	K
CNST2	145.0000000	
D0	0.00000300	sec
D1	1.50000000	sec
D4	0.00172414	sec
D11	0.03000000	sec
D13	0.00000400	sec
D16	0.00020000	sec
IN0	0.00002000	sec
ZGOPTNS		
======== CHANNEL f1 ========		
NUC1	1H	
P1	12.00	usec
P2	24.00	usec
P28	0.00	usec
ND0	2	
TD	512	
SFO1	150.9141	MHz
FIDRES	48.822838	Hz
SW	165.639	ppm
FnMODE	Echo-Antiecho	
SI	1024	
SF	600.1300224	MHz
WDW	QSINE	
SSB	2	
LB	0.00	Hz
GB	0	
PC	1.40	
SI	1024	
MC2	echo-antiecho	
SF	150.9027821	MHz
WDW	QSINE	
SSB	2	
LB	0.00	Hz
GB	0	

Supplementary Figure 58. HSQC NMR spectrum of compound 9

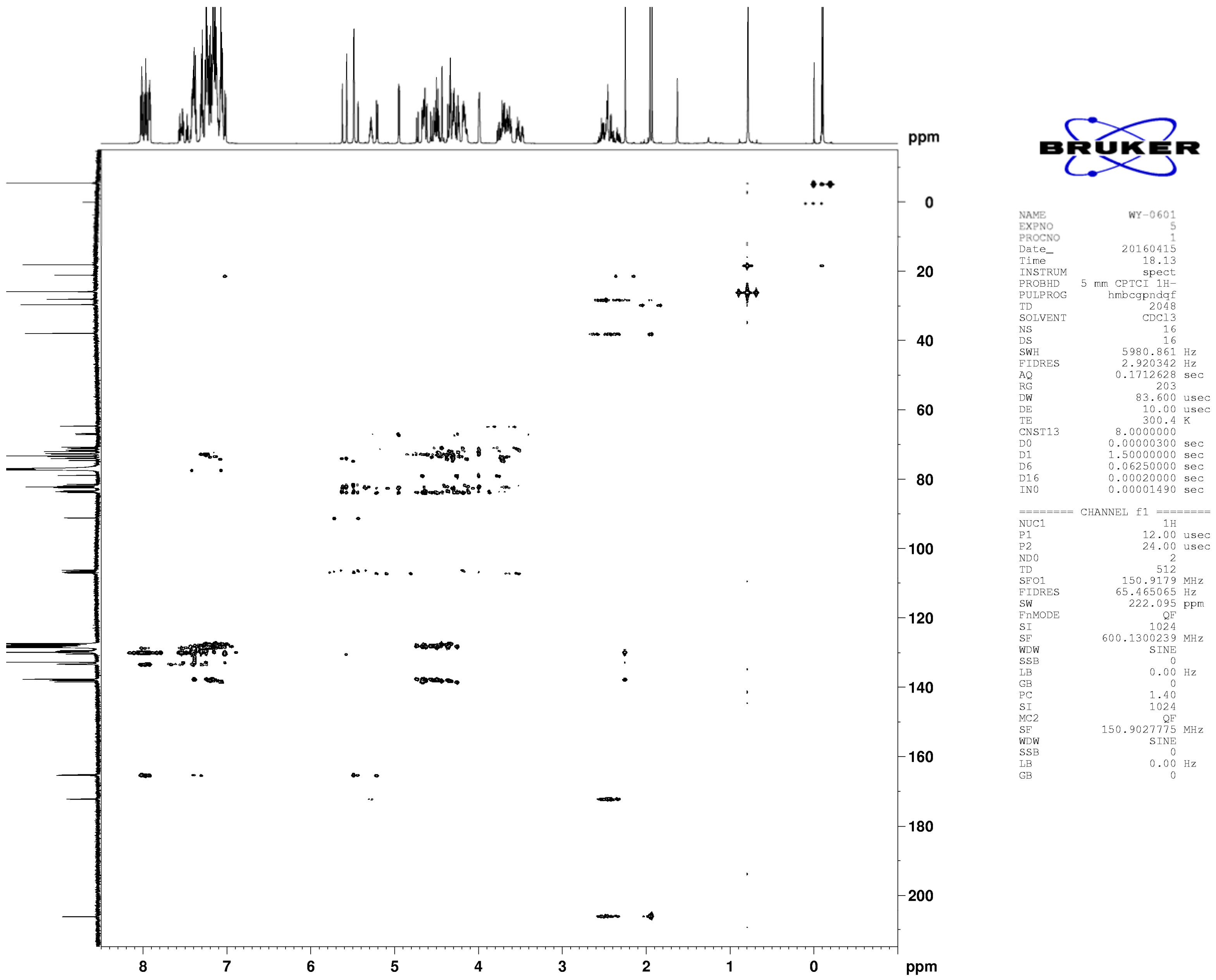

Supplementary Figure 59. HMBC NMR spectrum of compound 9

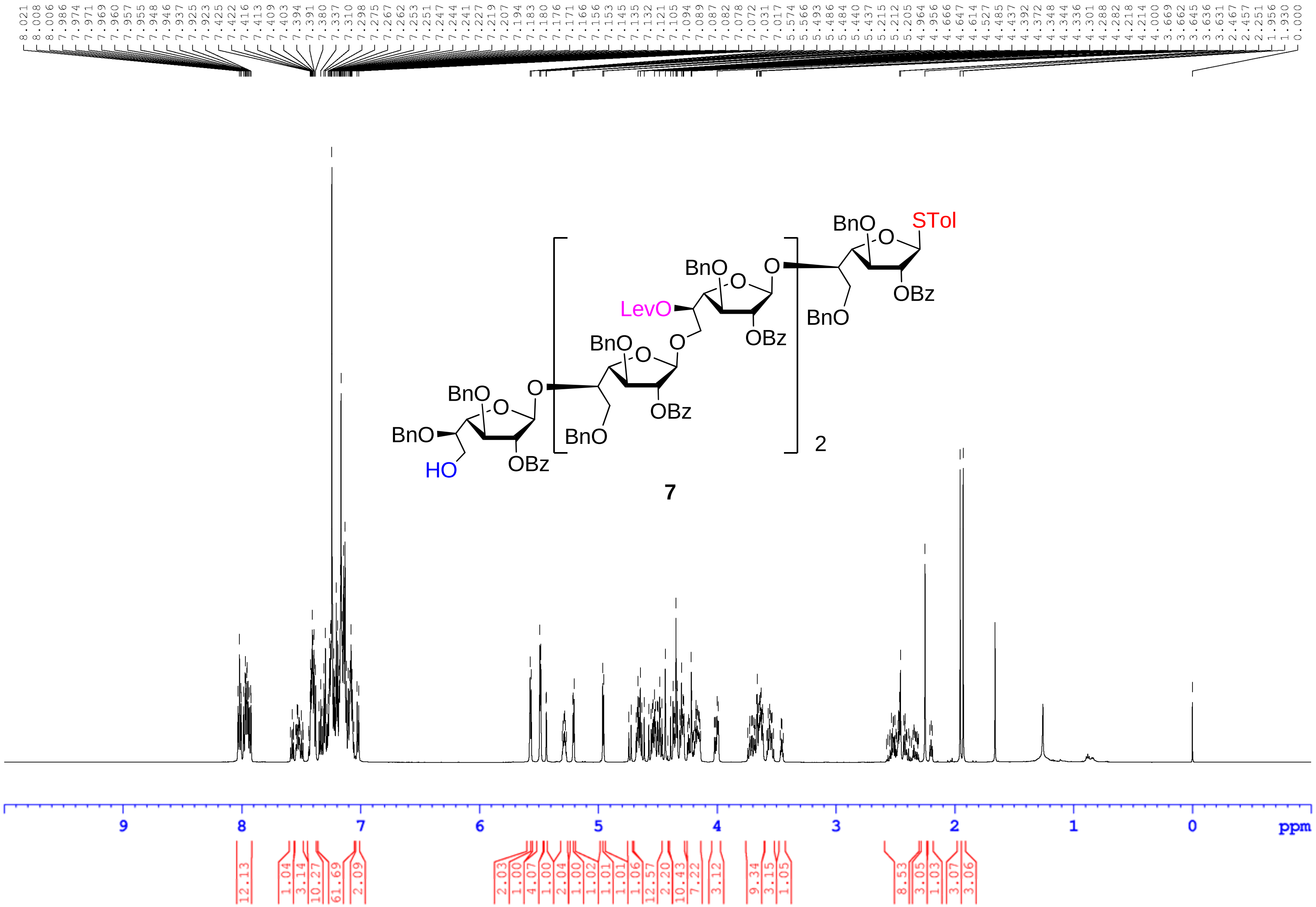

Supplementary Figure 60. ^{1}H NMR spectrum of compound 7

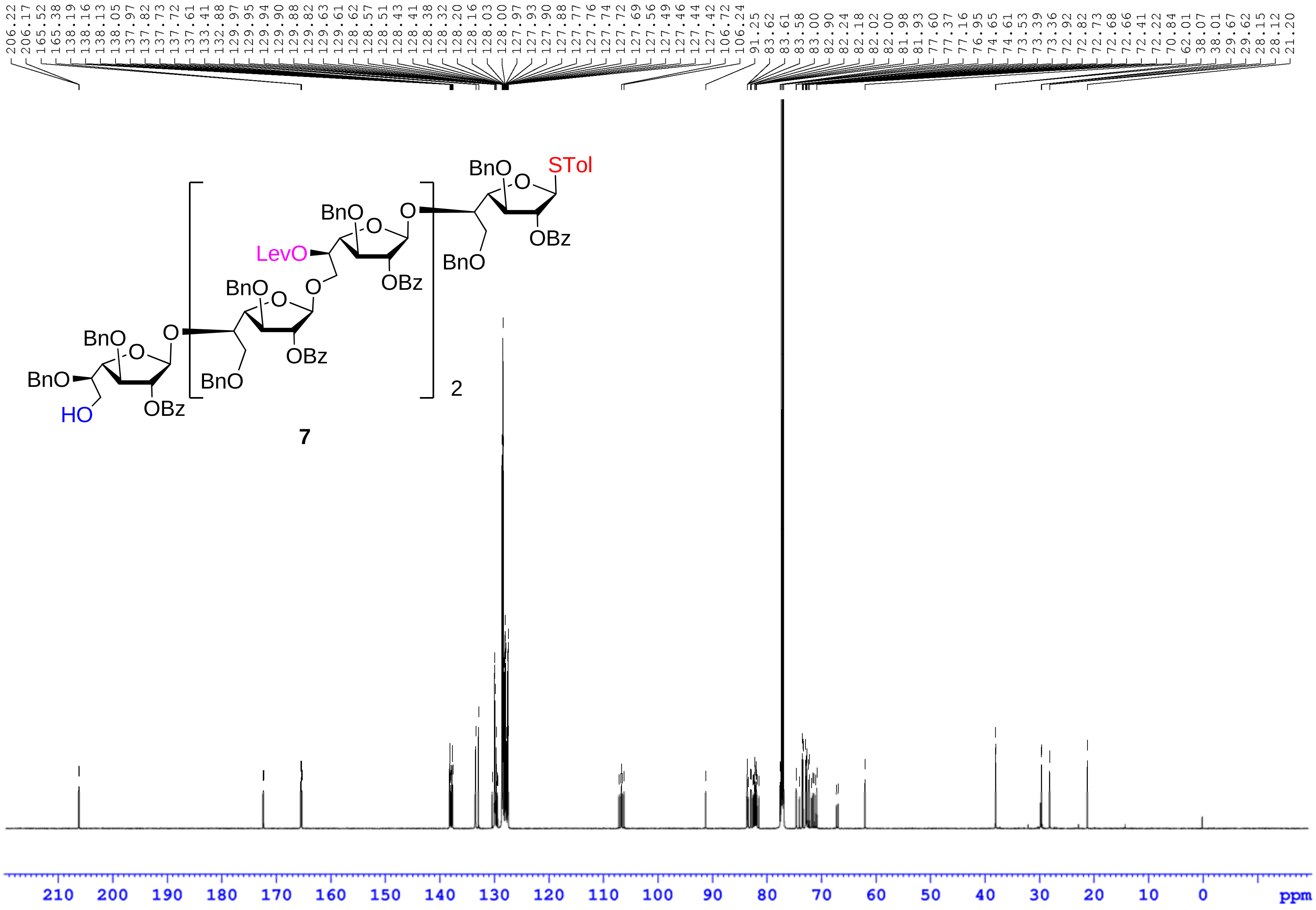

Supplementary Figure 61. ^{13}**C NMR spectrum of compound 7**

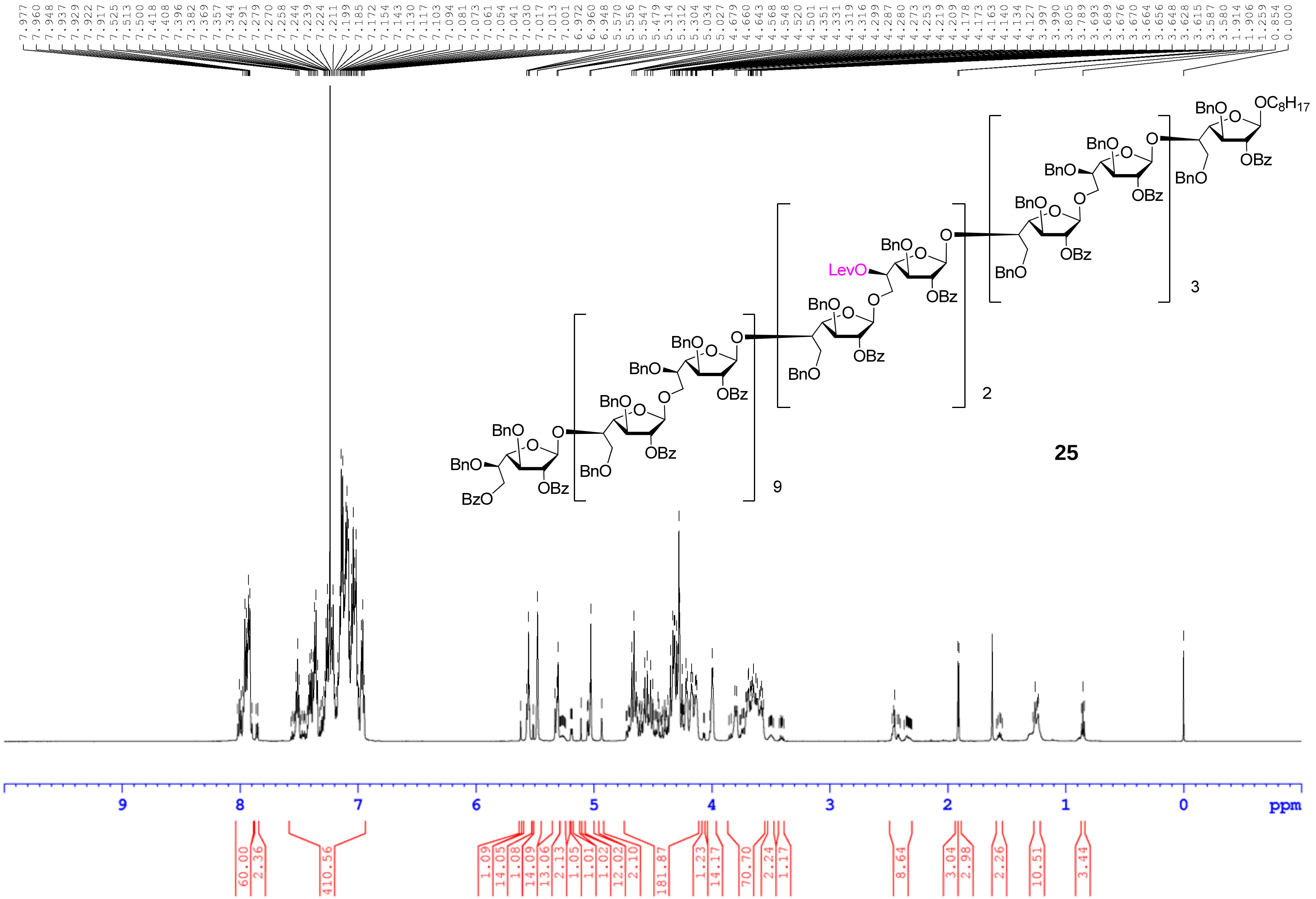

Supplementary Figure 62. ^{1}**H NMR spectrum of compound 25**

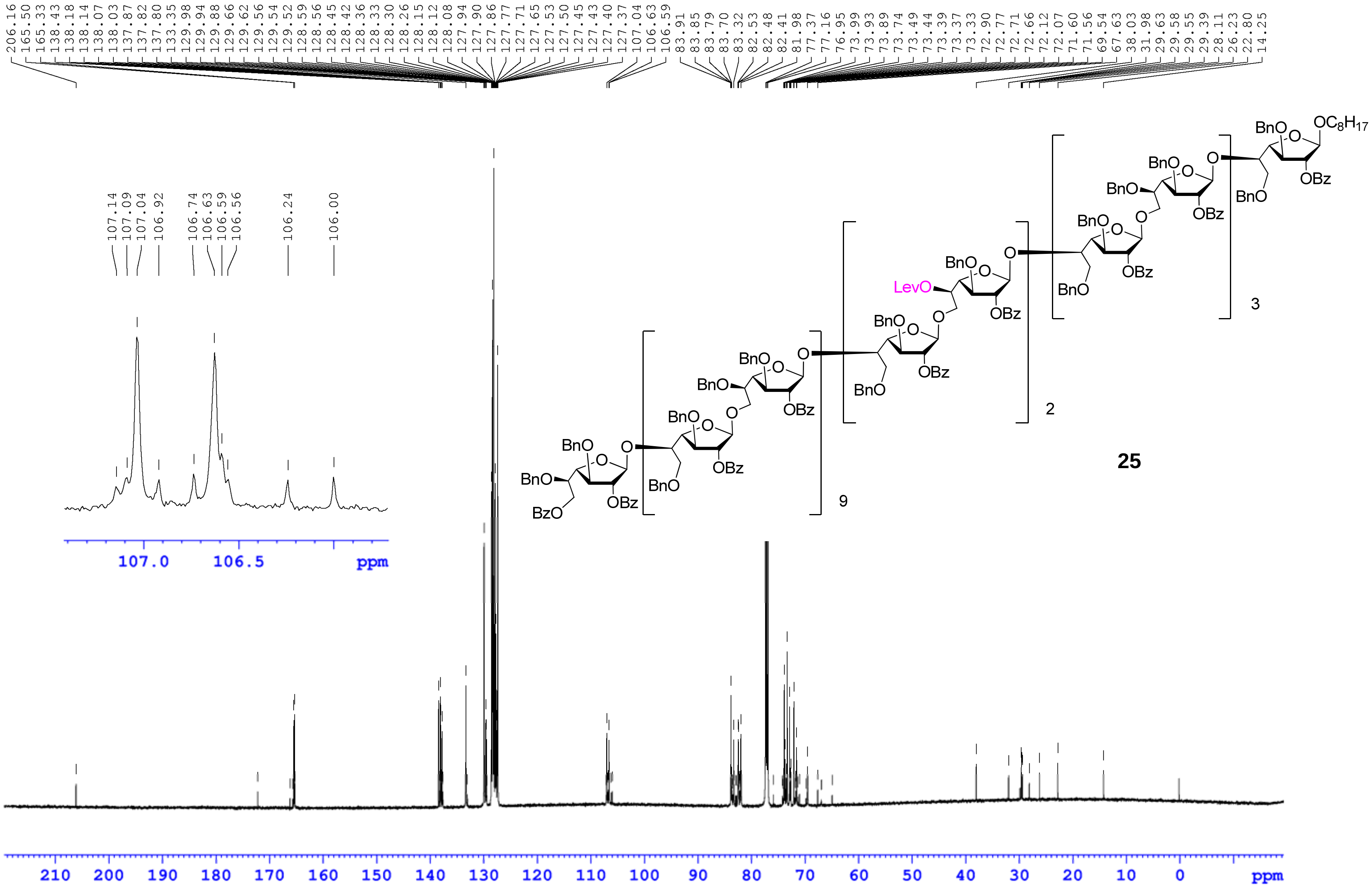

Supplementary Figure 63. ^{13}C NMR spectrum of compound 25

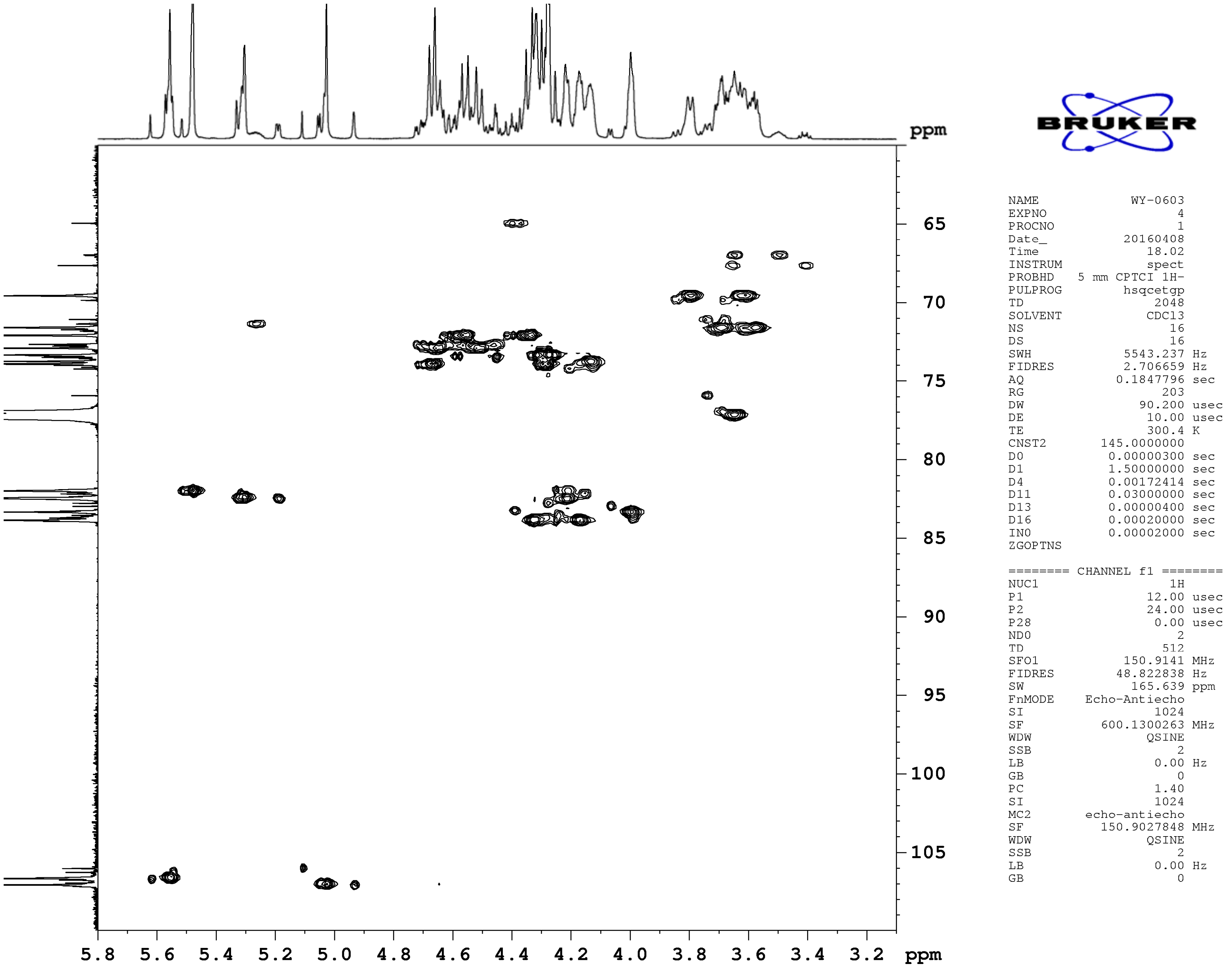

Supplementary Figure 64. HSQC NMR spectrum of compound 25

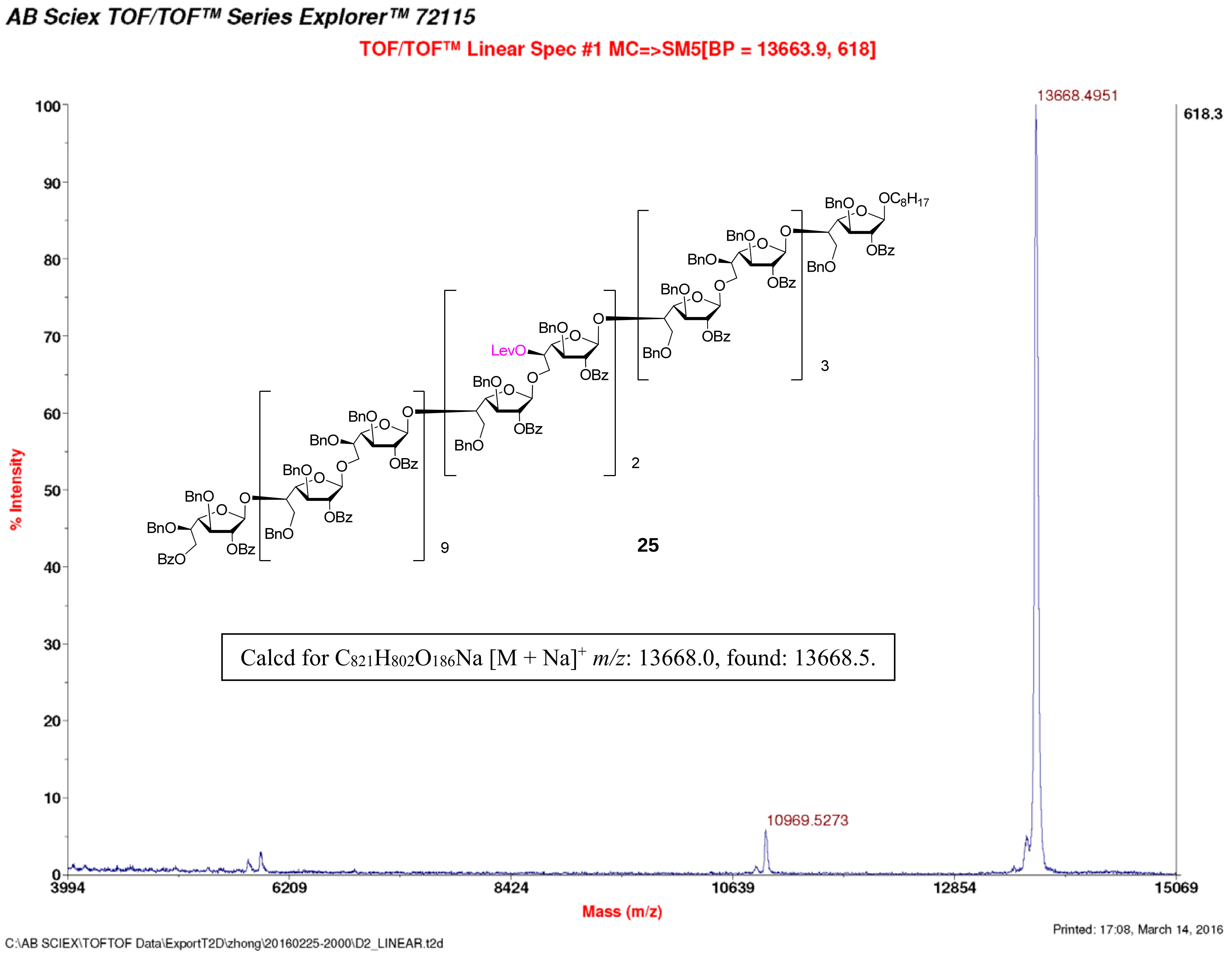

Supplementary Figure 65. MALDI-TOF MS spectrum of compound 25

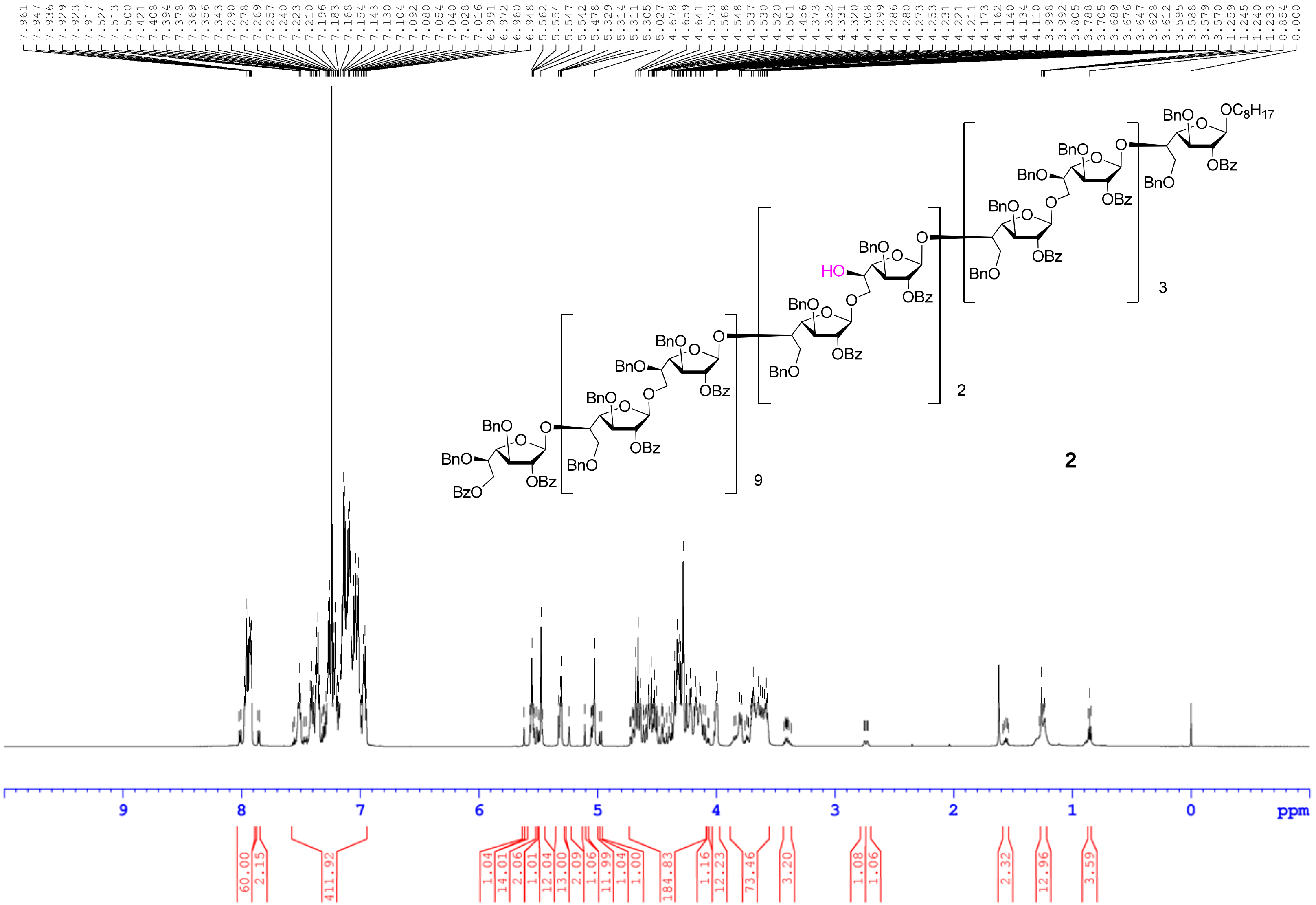

2

Supplementary Figure 66. ^{1}H NMR spectrum of compound 2

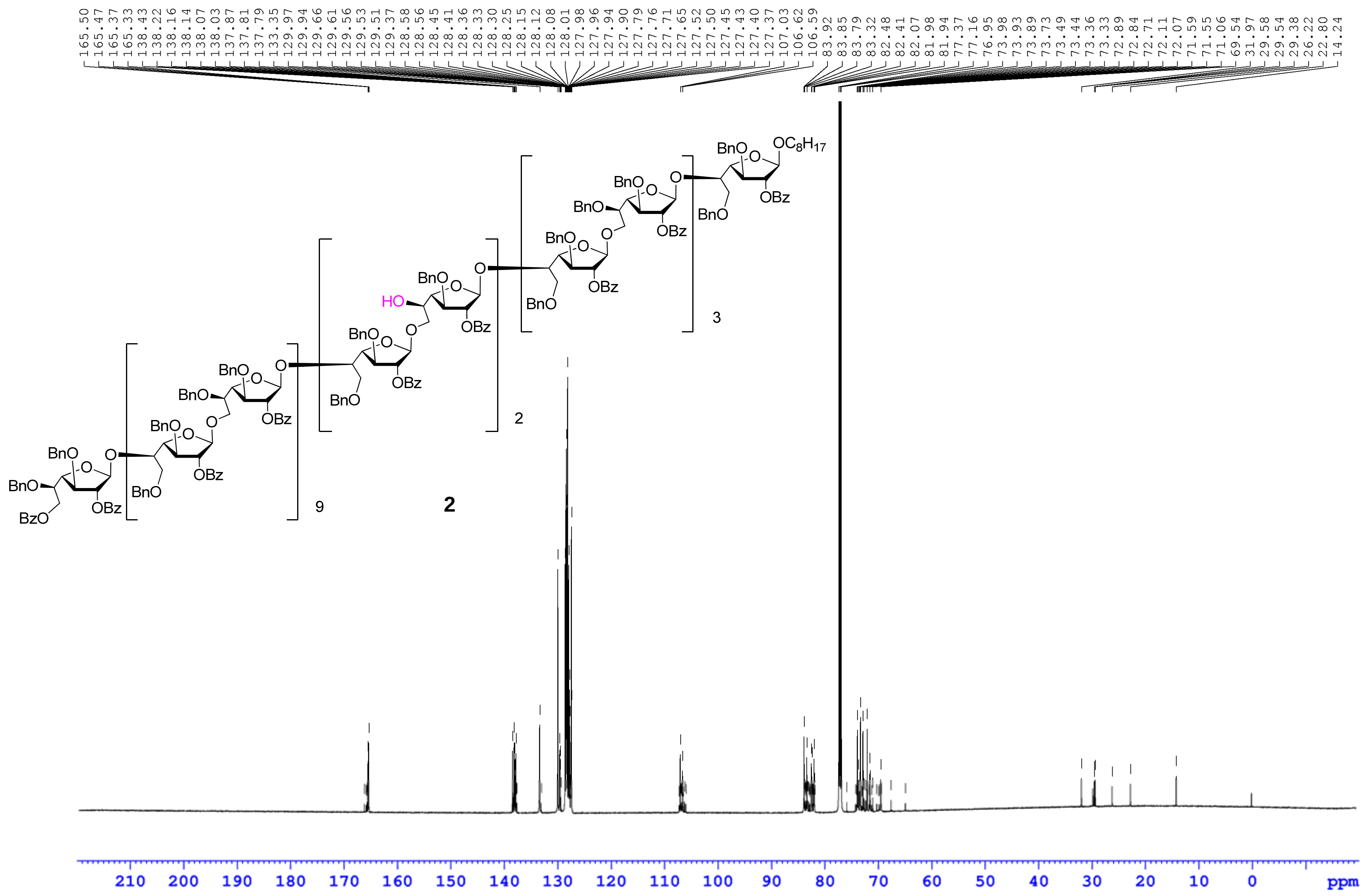

Supplementary Figure 67. ^{13}C **NMR spectrum of compound 2**

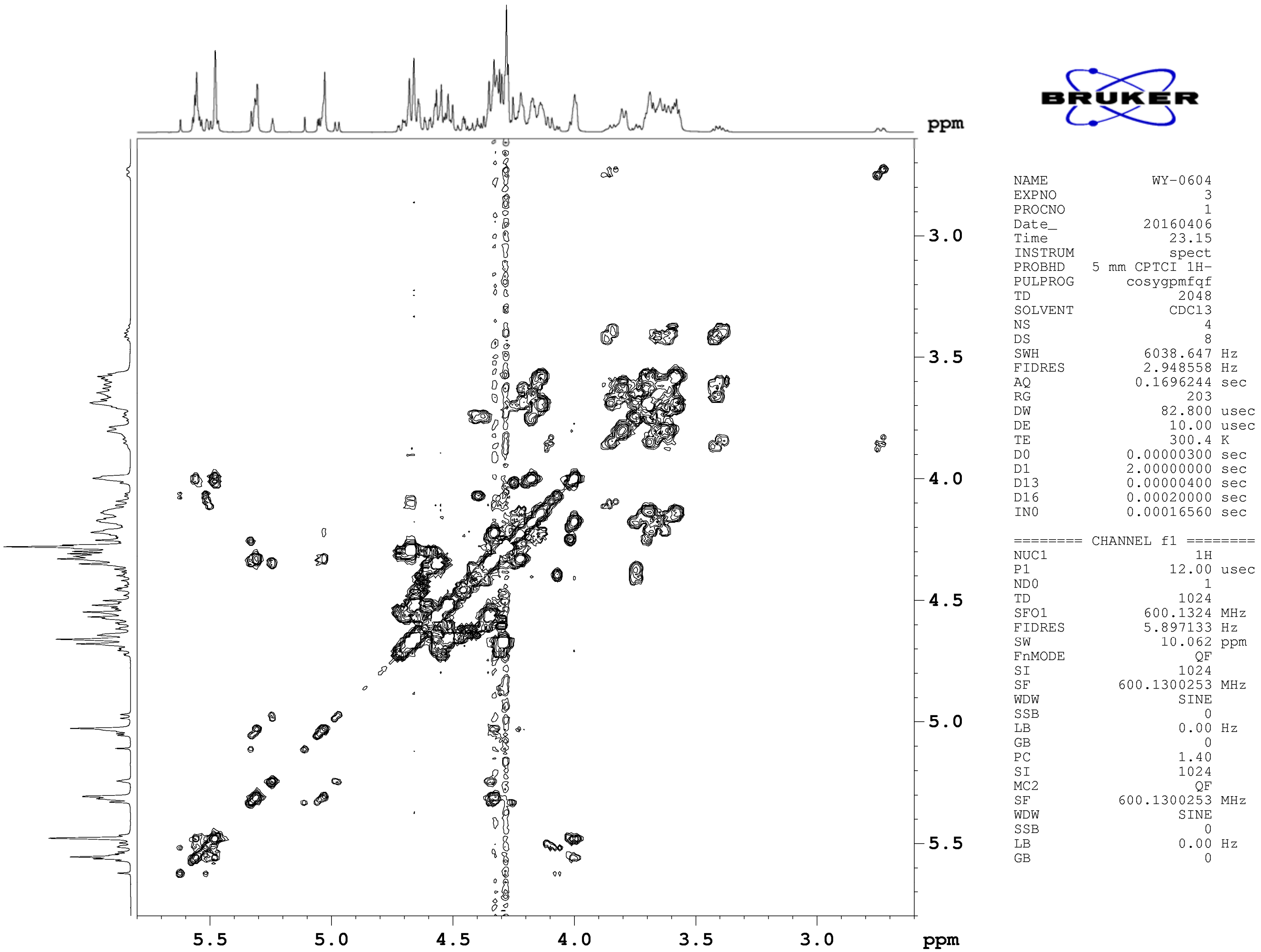

Supplementary Figure 68. COSY NMR spectrum of compound 2

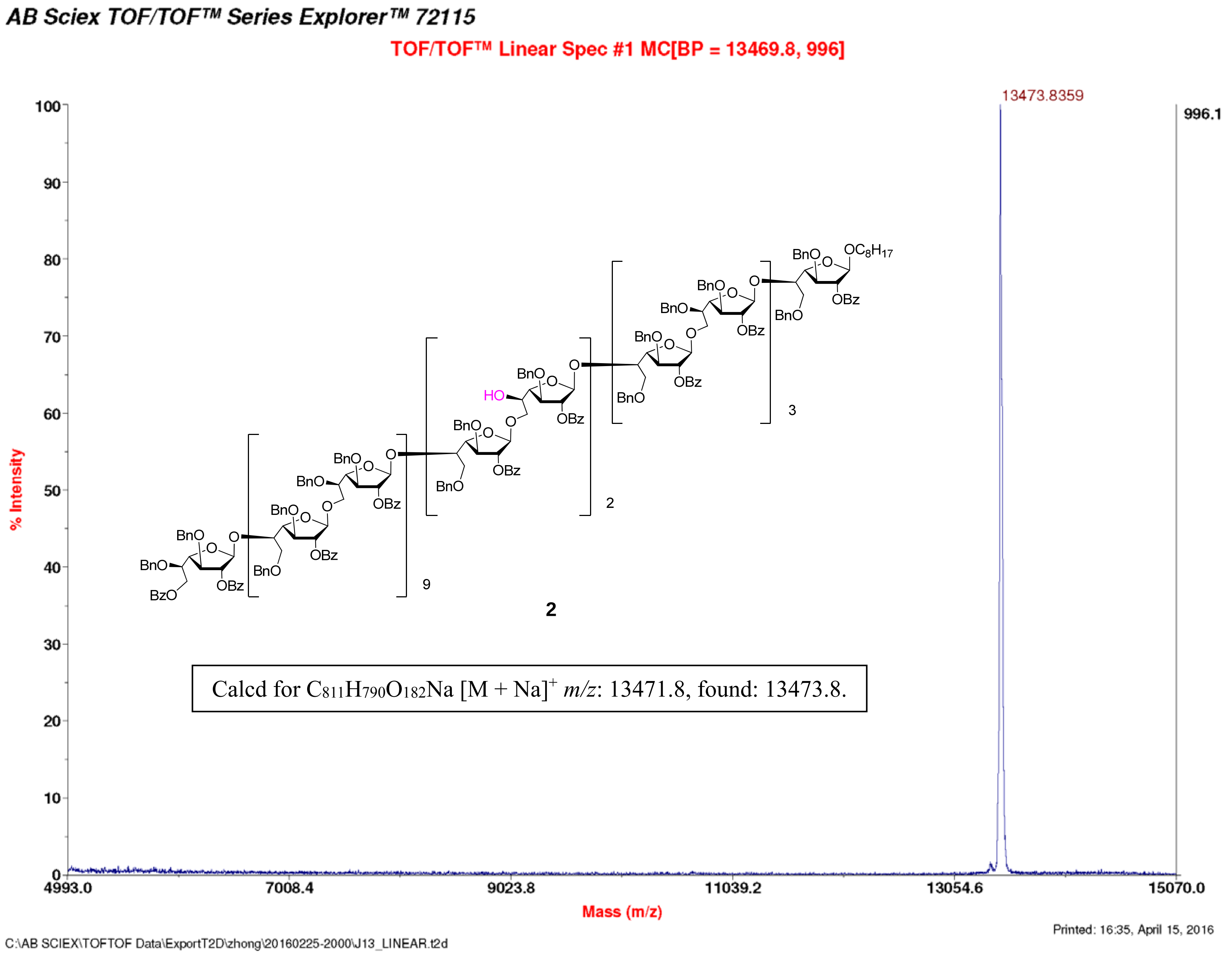

Supplementary Figure 69. MALDI-TOF MS spectrum of compound 2

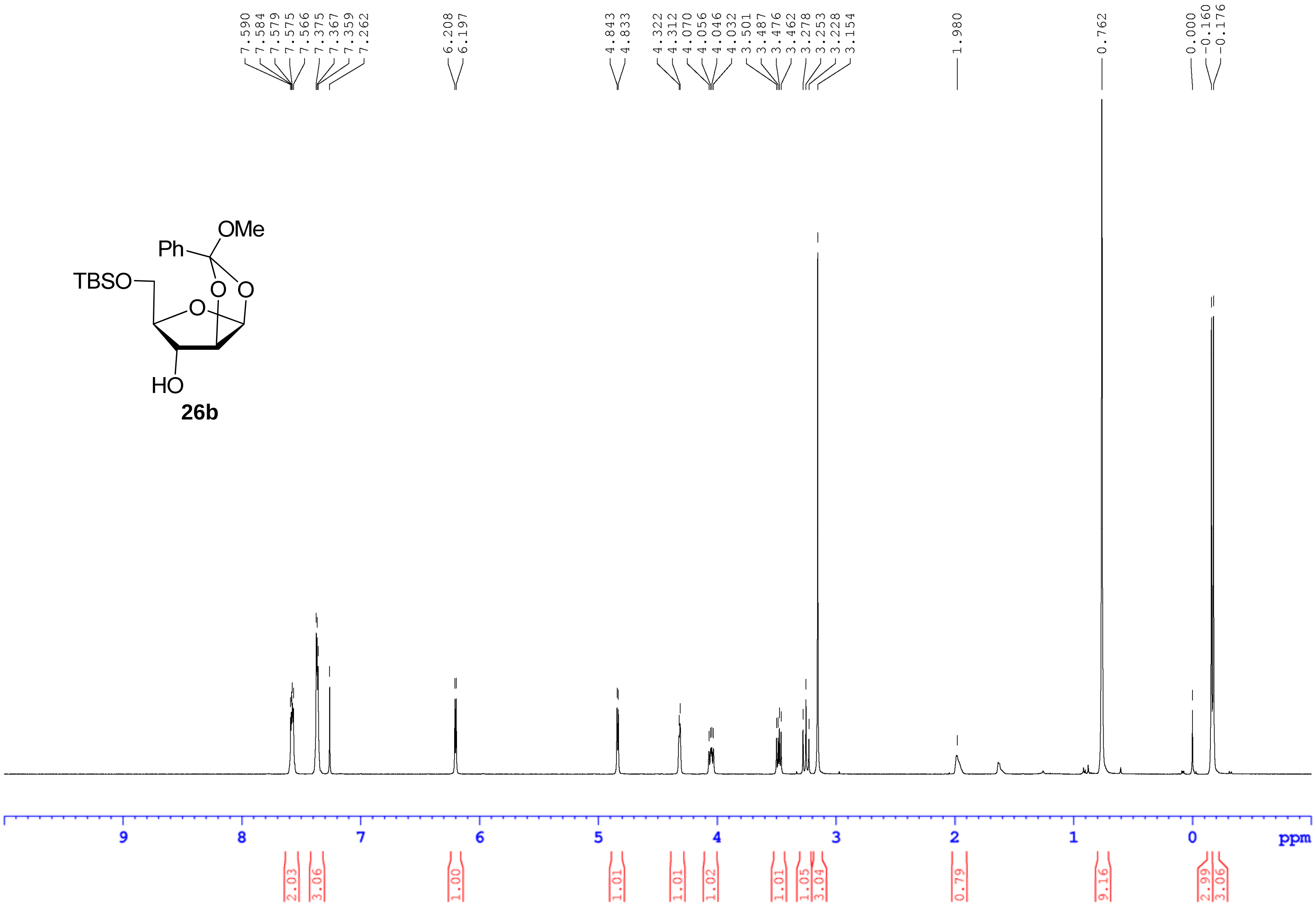

Supplementary Figure 70. ^{1}H NMR spectrum of compound 26b

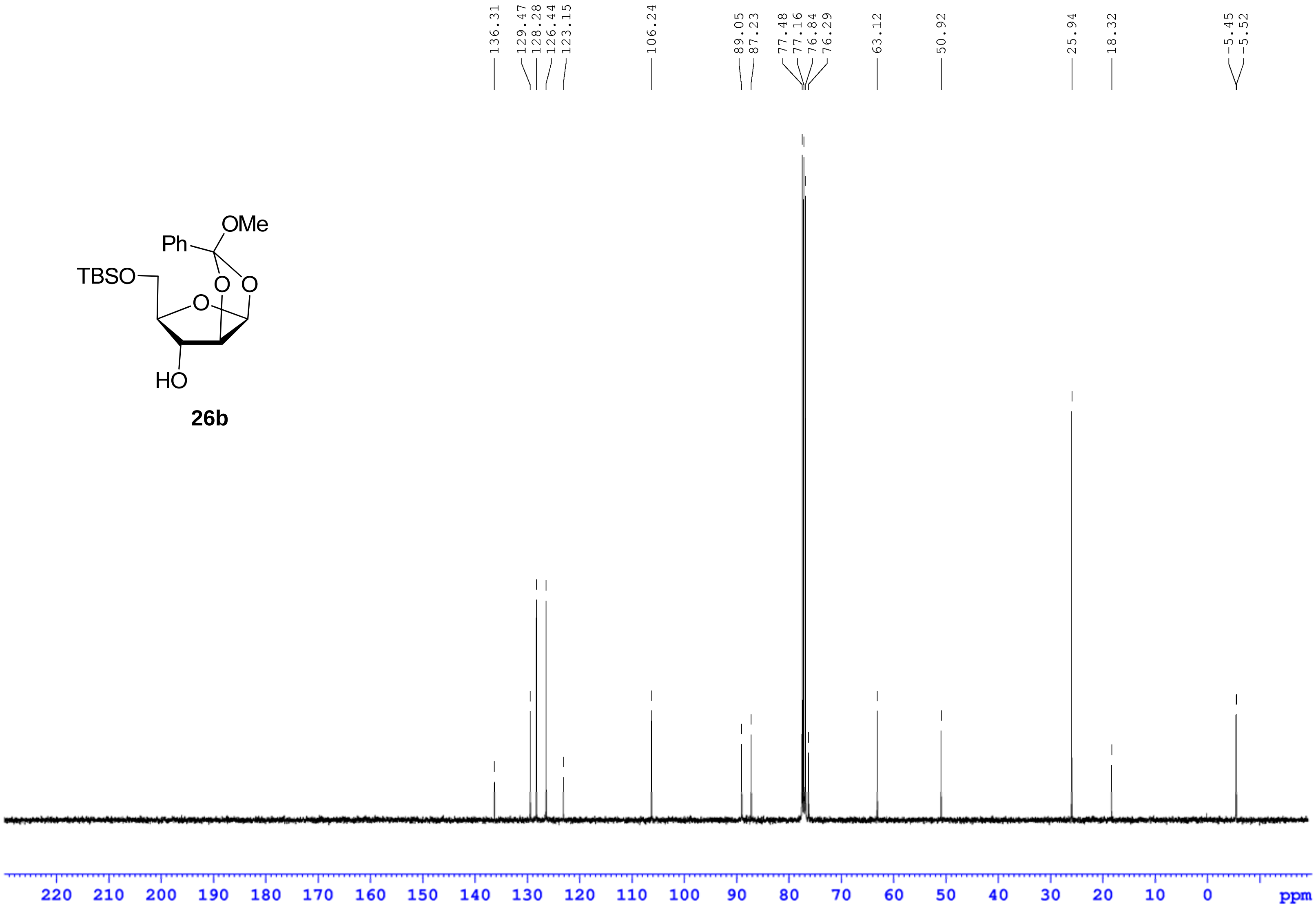

Supplementary Figure 71. ^{13}C NMR spectrum of compound 26b

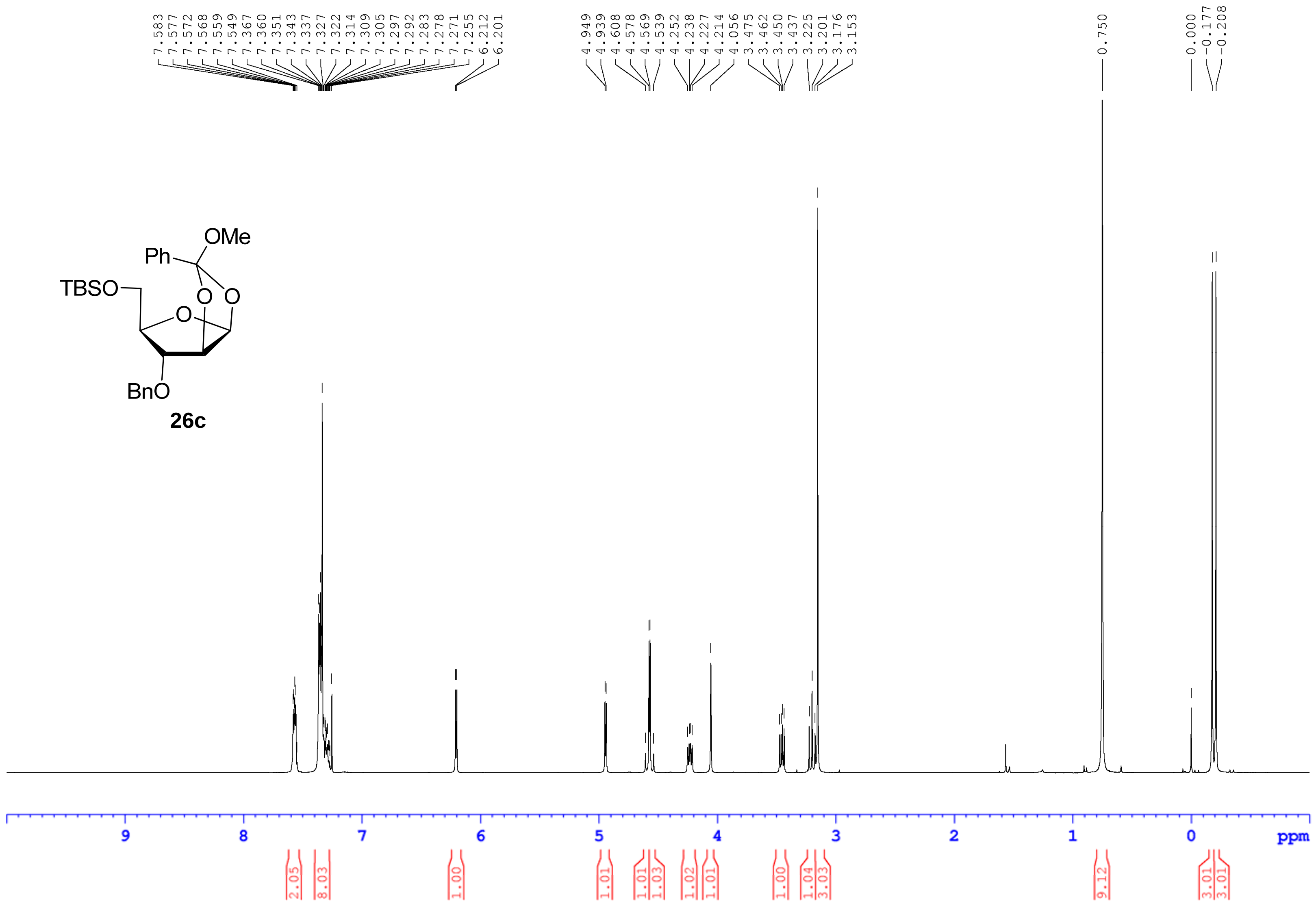

Supplementary Figure 72. ^{1}H NMR spectrum of compound 26c

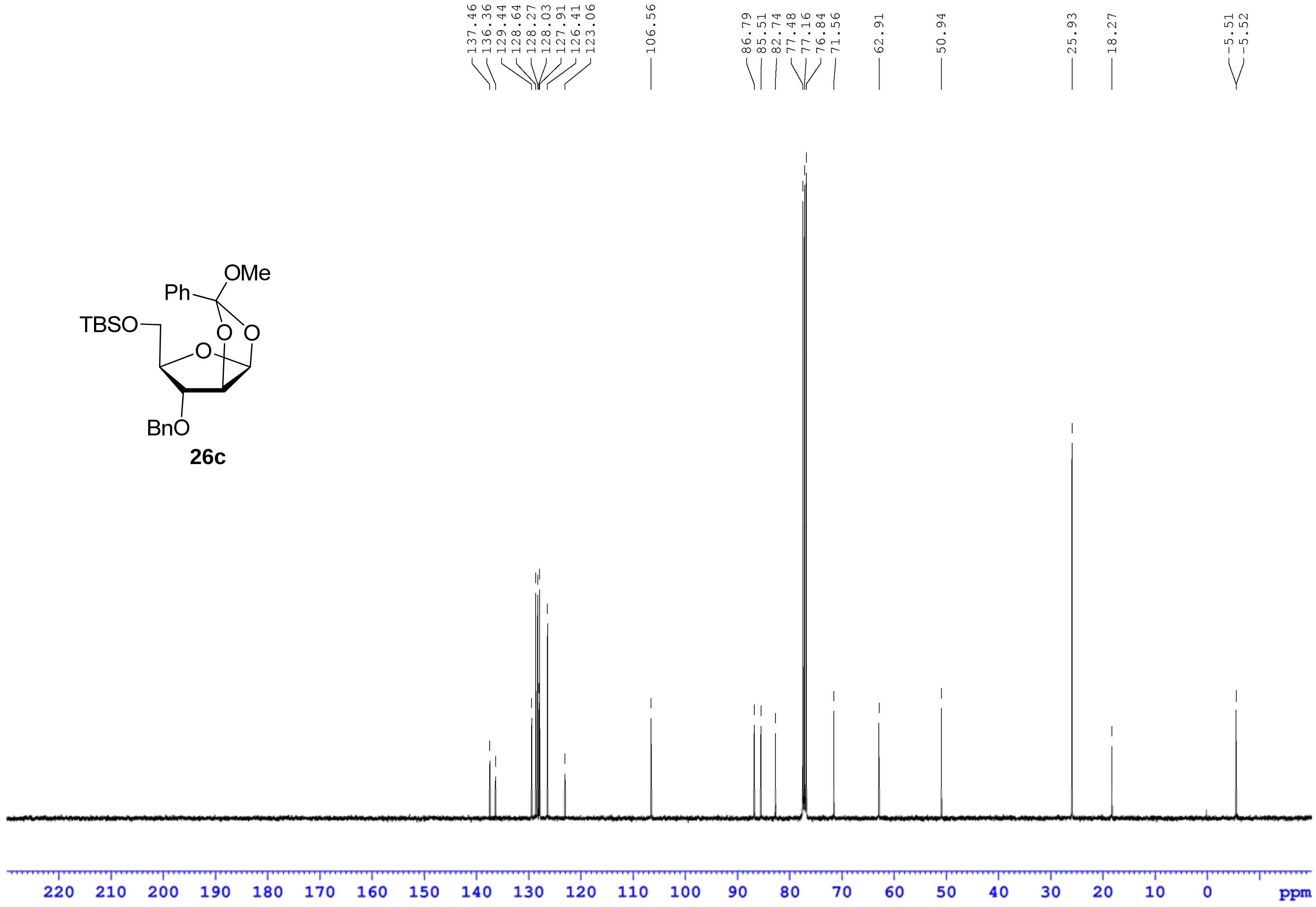

Supplementary Figure 73. ^{13}C NMR spectrum of compound 26c

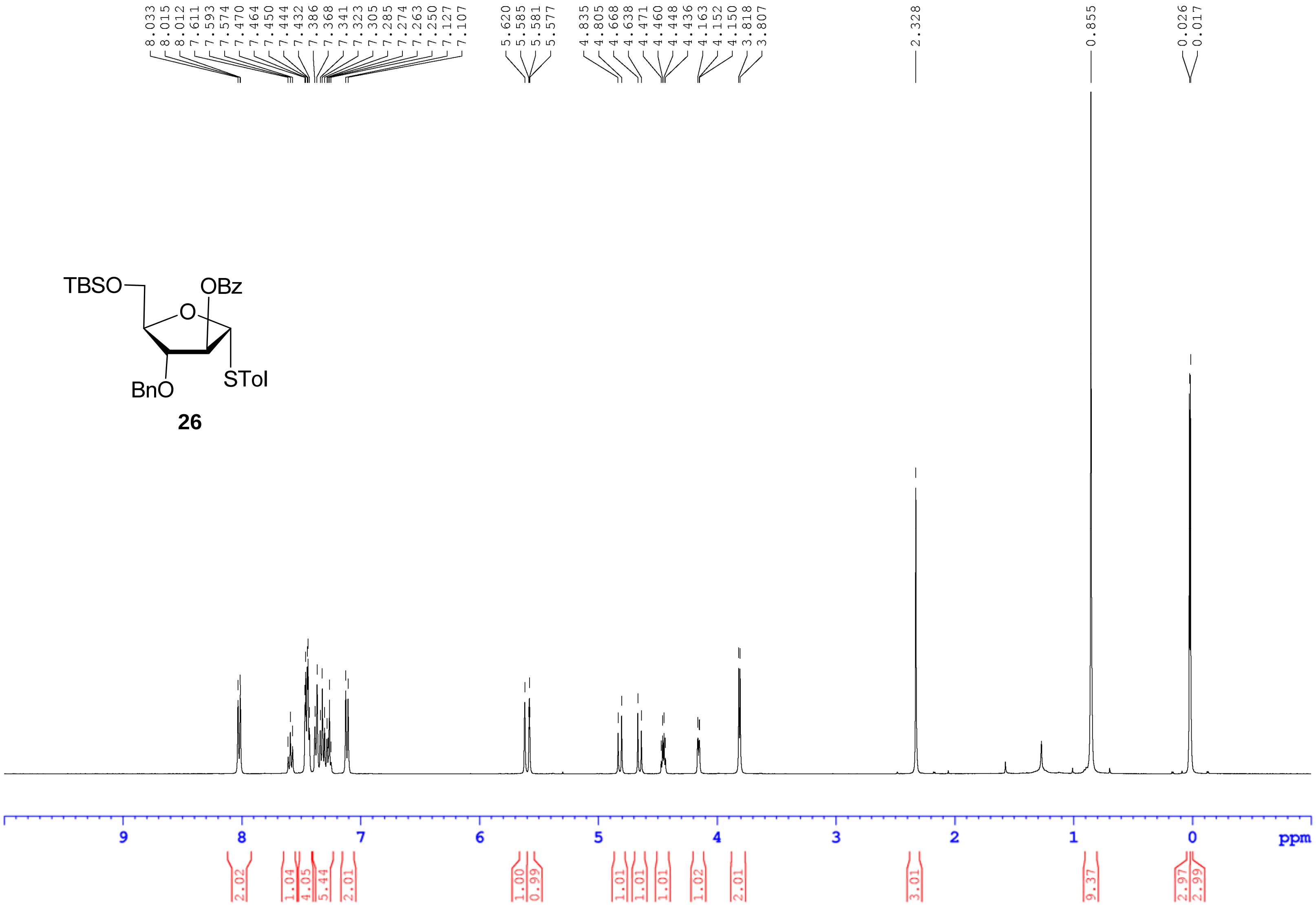

Supplementary Figure 74. ^{1}H NMR spectrum of compound 26

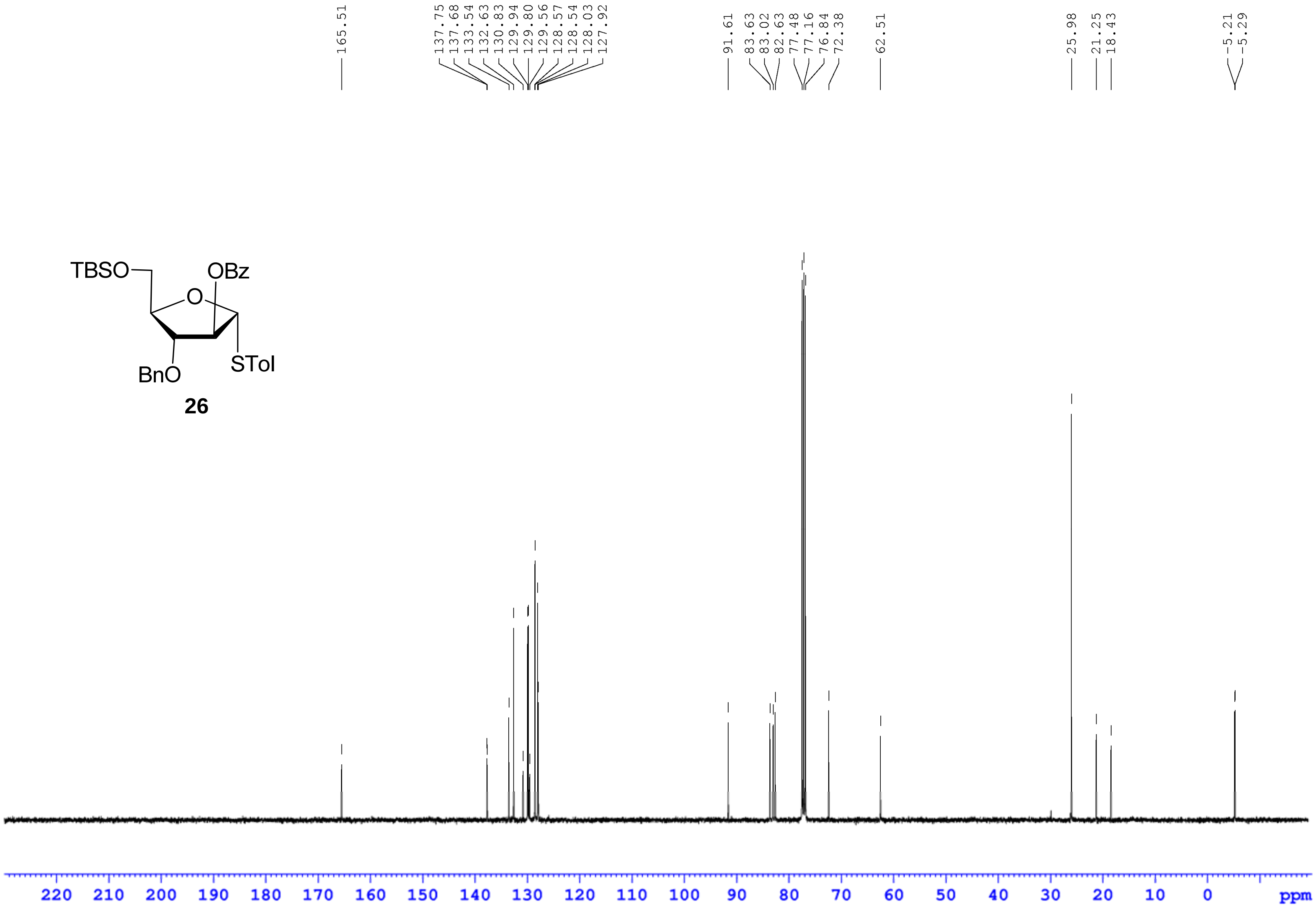

Supplementary Figure 75. ^{13}C NMR spectrum of compound 26

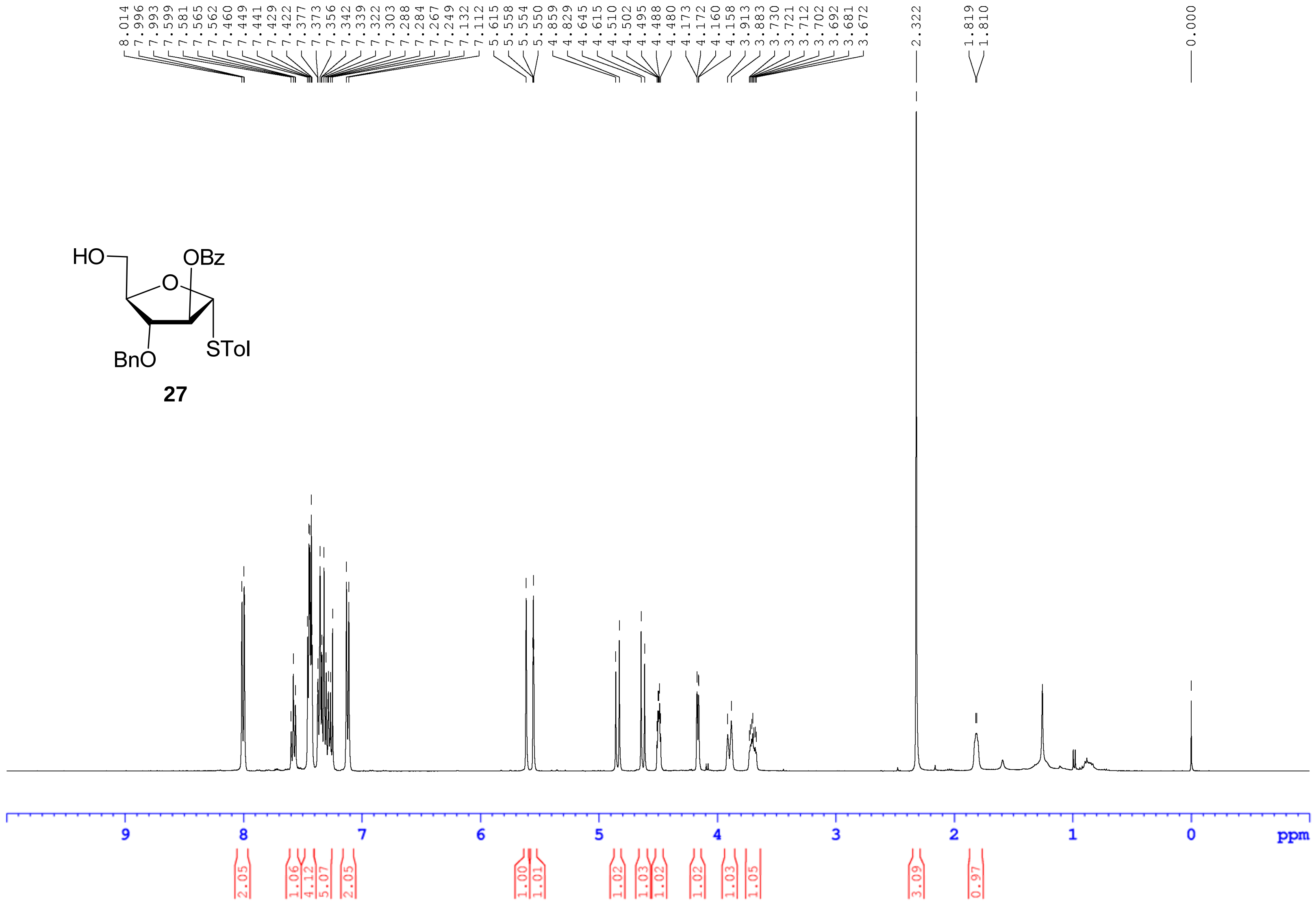

Supplementary Figure 76. ^{1}H NMR spectrum of compound 27

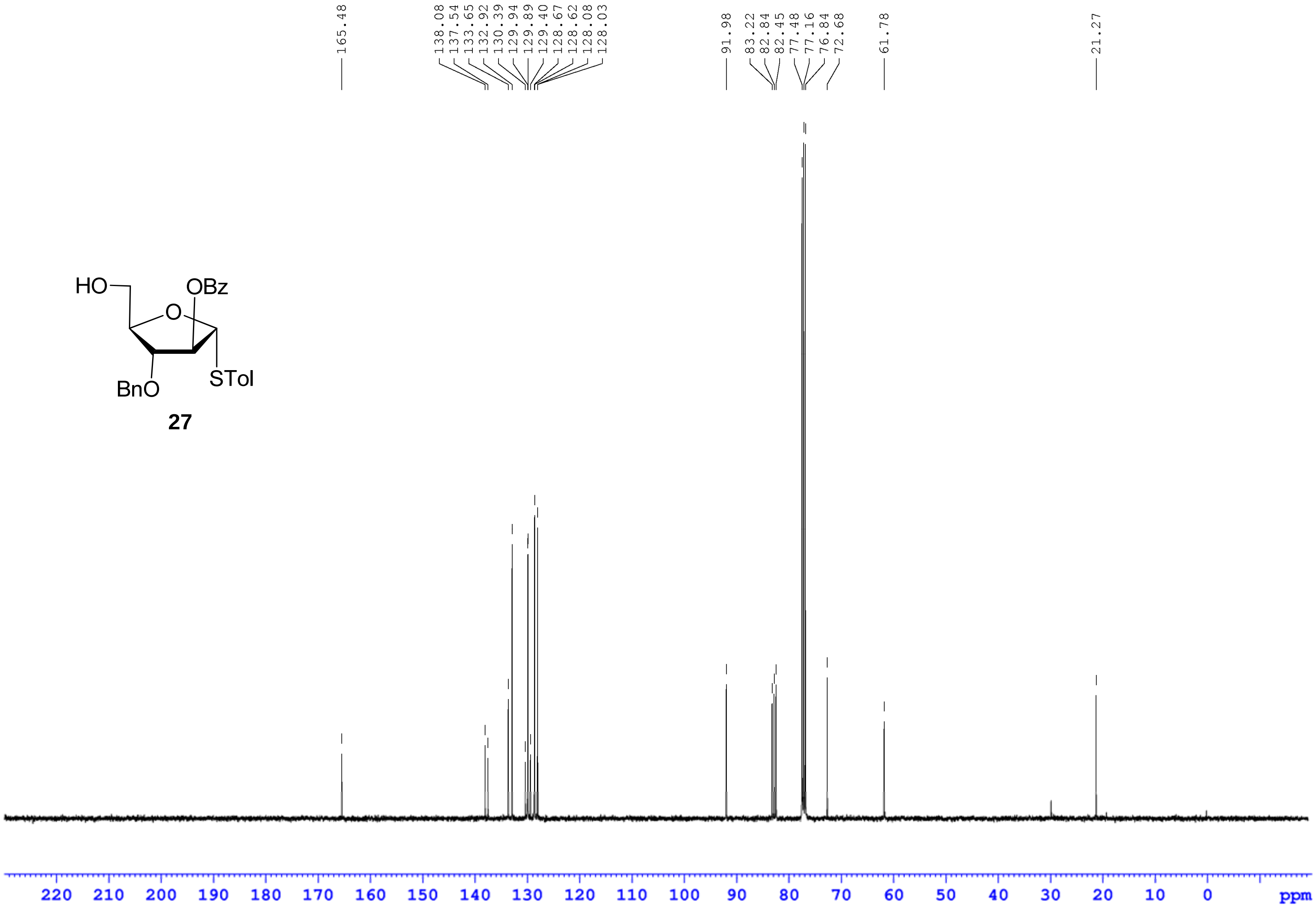

Supplementary Figure 77. ^{13}C NMR spectrum of compound 27

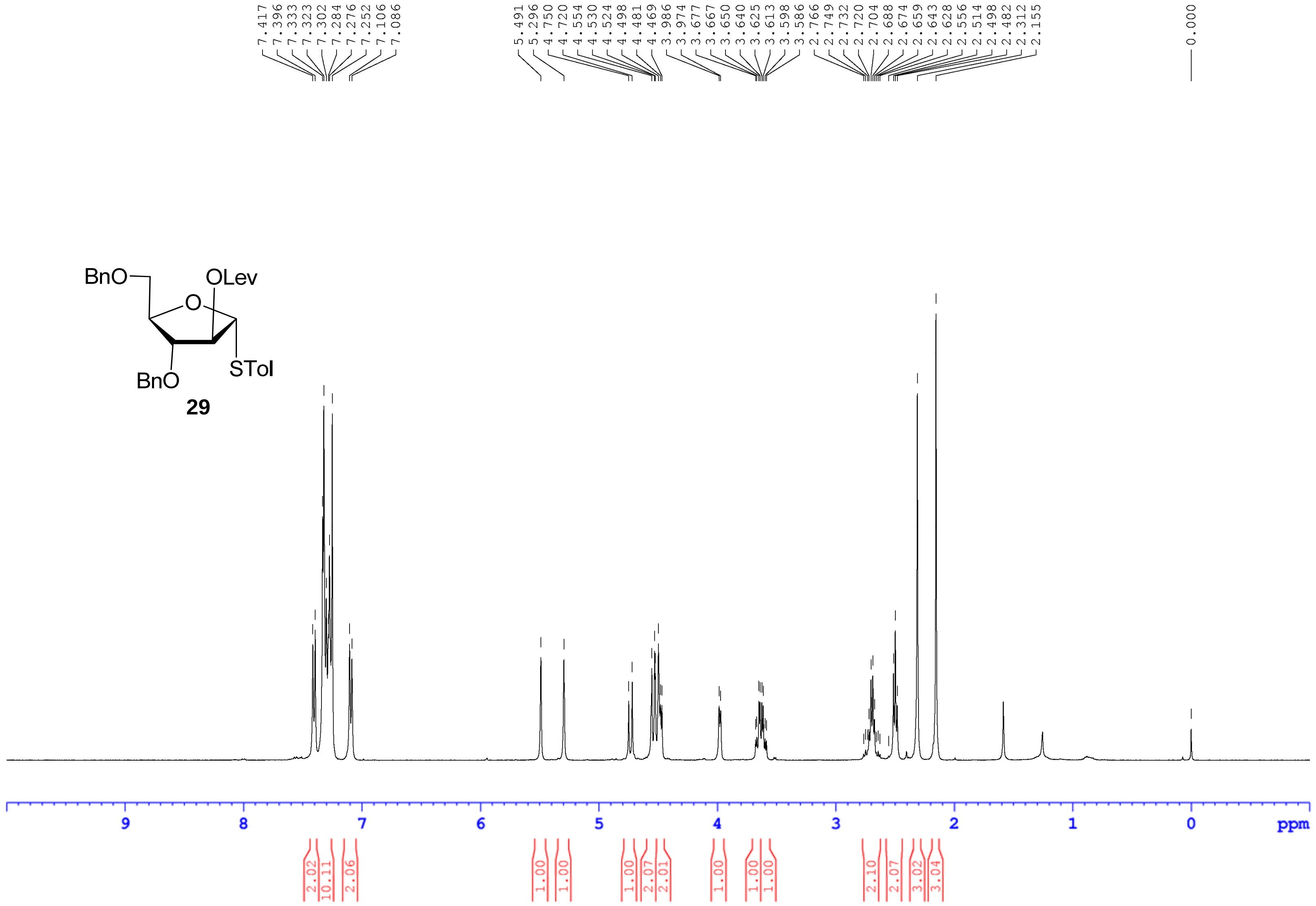

Supplementary Figure 78. ^{1}H NMR spectrum of compound 29

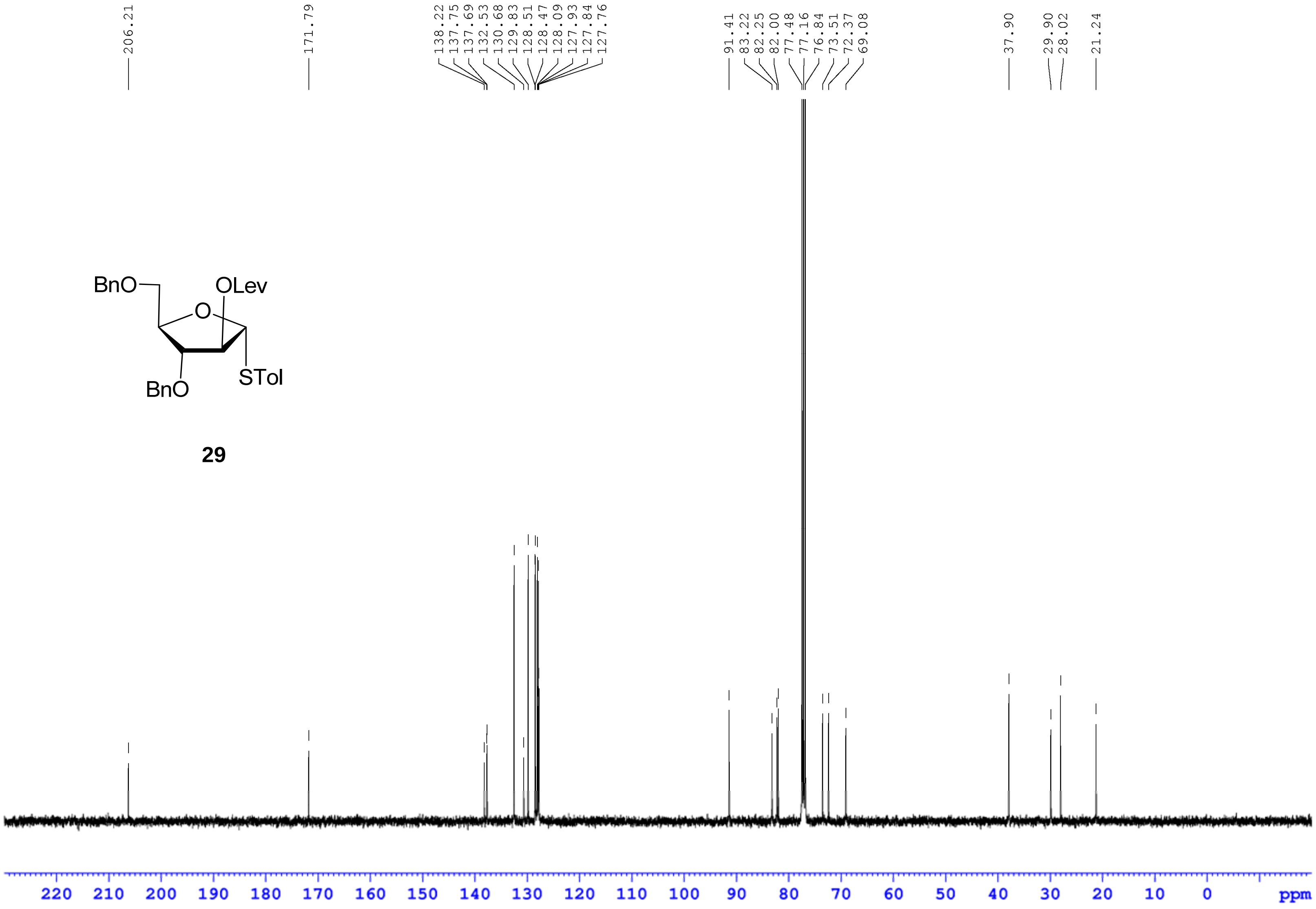

Supplementary Figure 79. ^{13}**C NMR spectrum of compound 29**

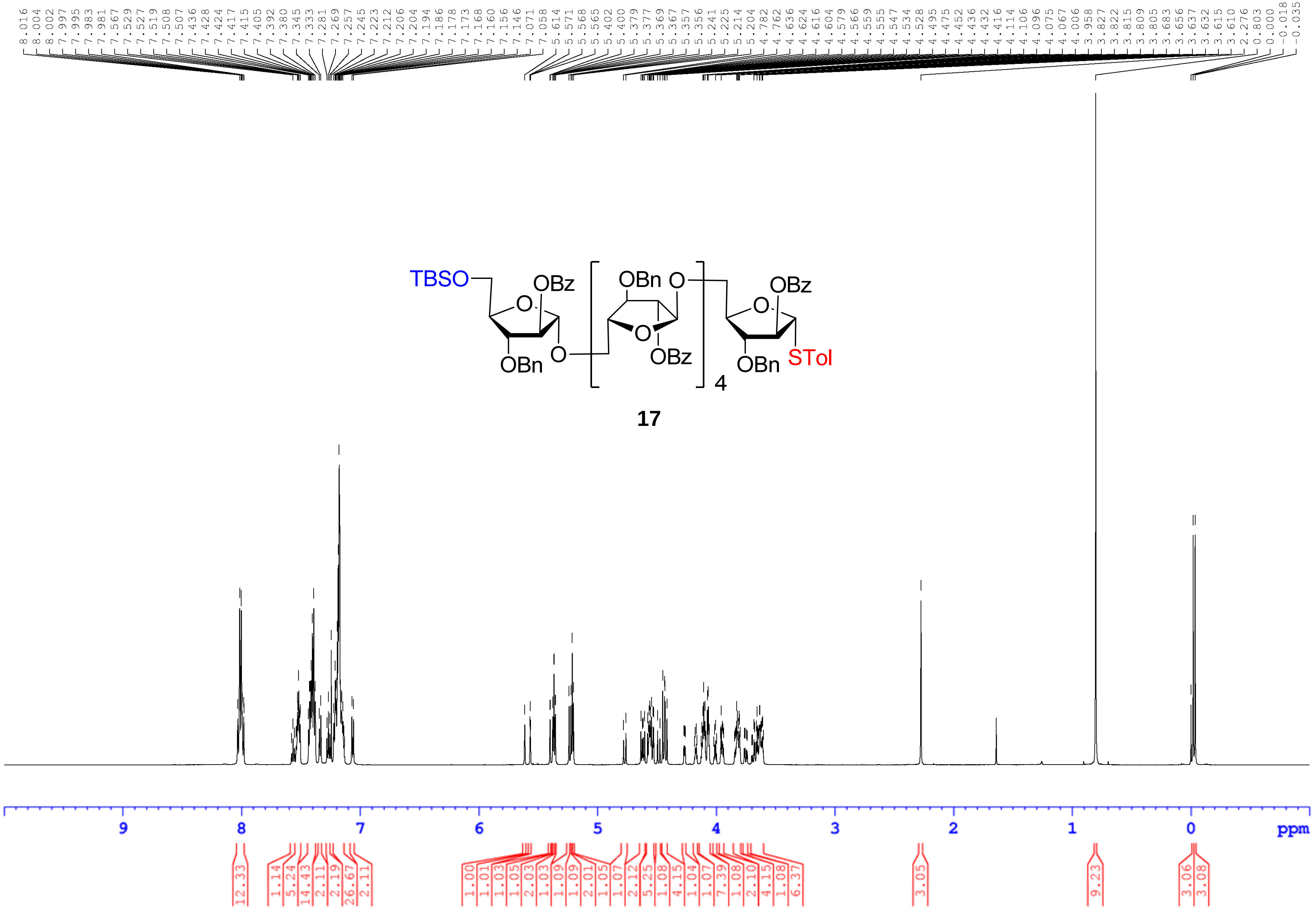

Supplementary Figure 80. ^{1}H NMR spectrum of compound 17

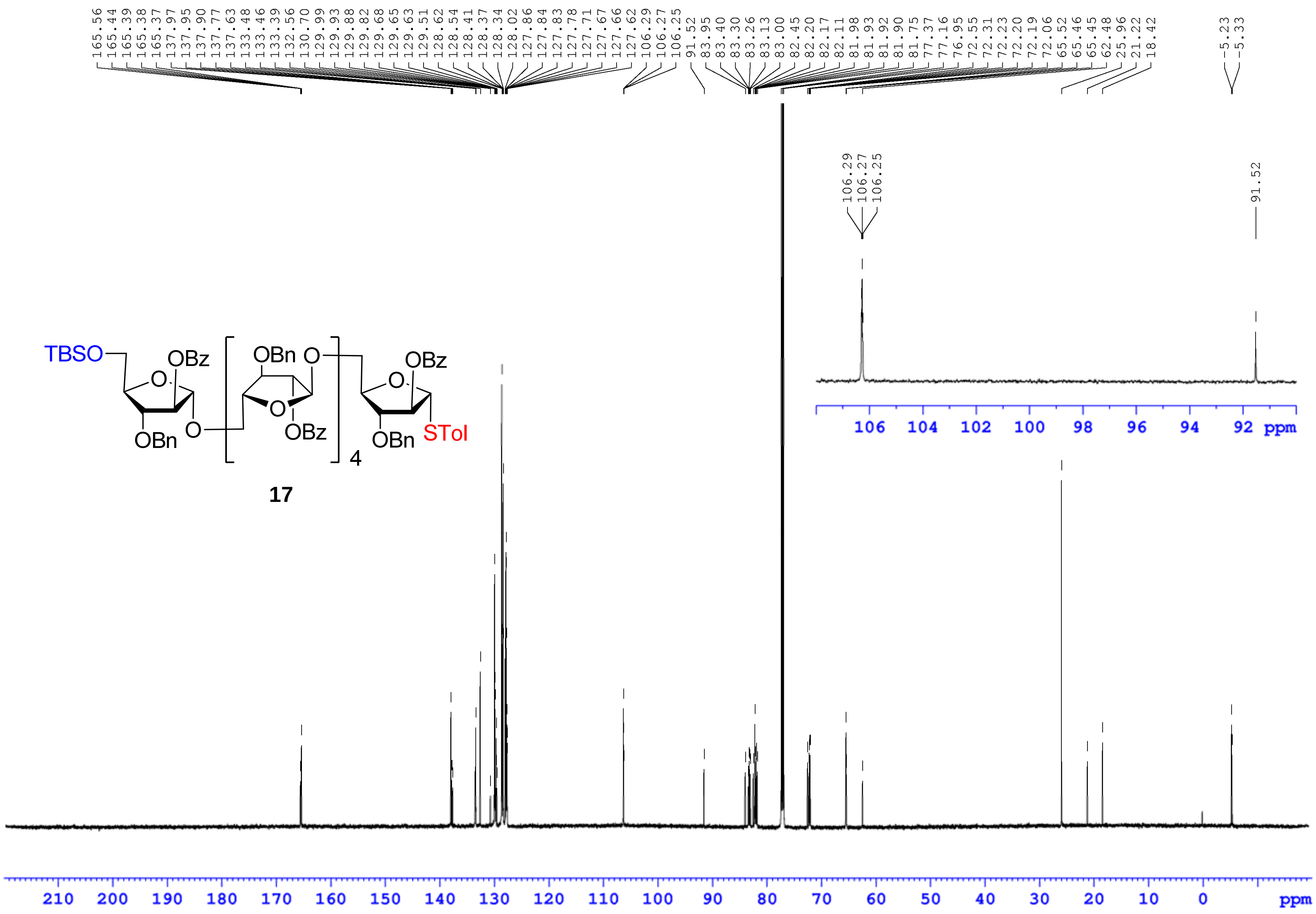

Supplementary Figure 81. ^{13}**C NMR spectrum of compound 17**

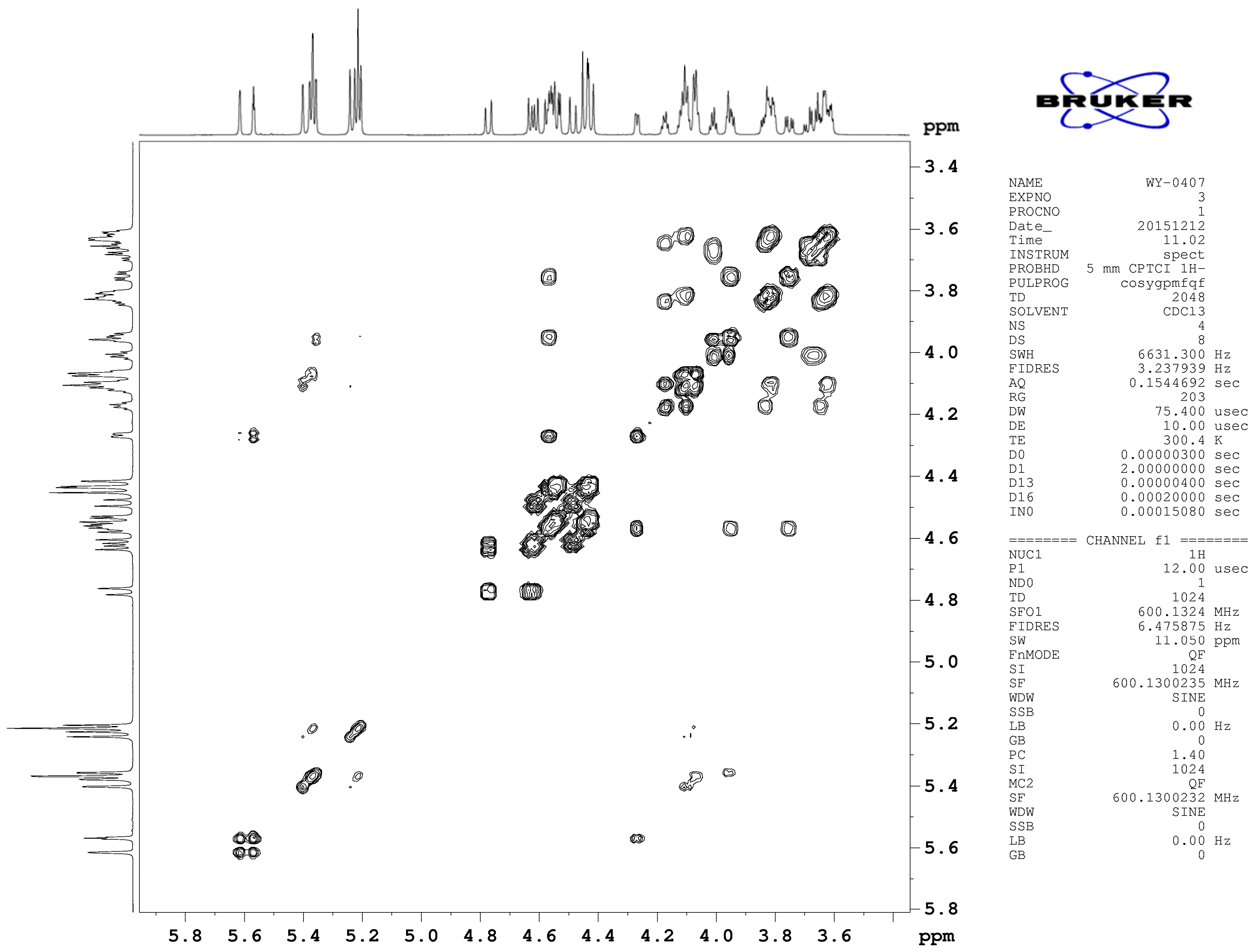

Supplementary Figure 82. COSY NMR spectrum of compound 17

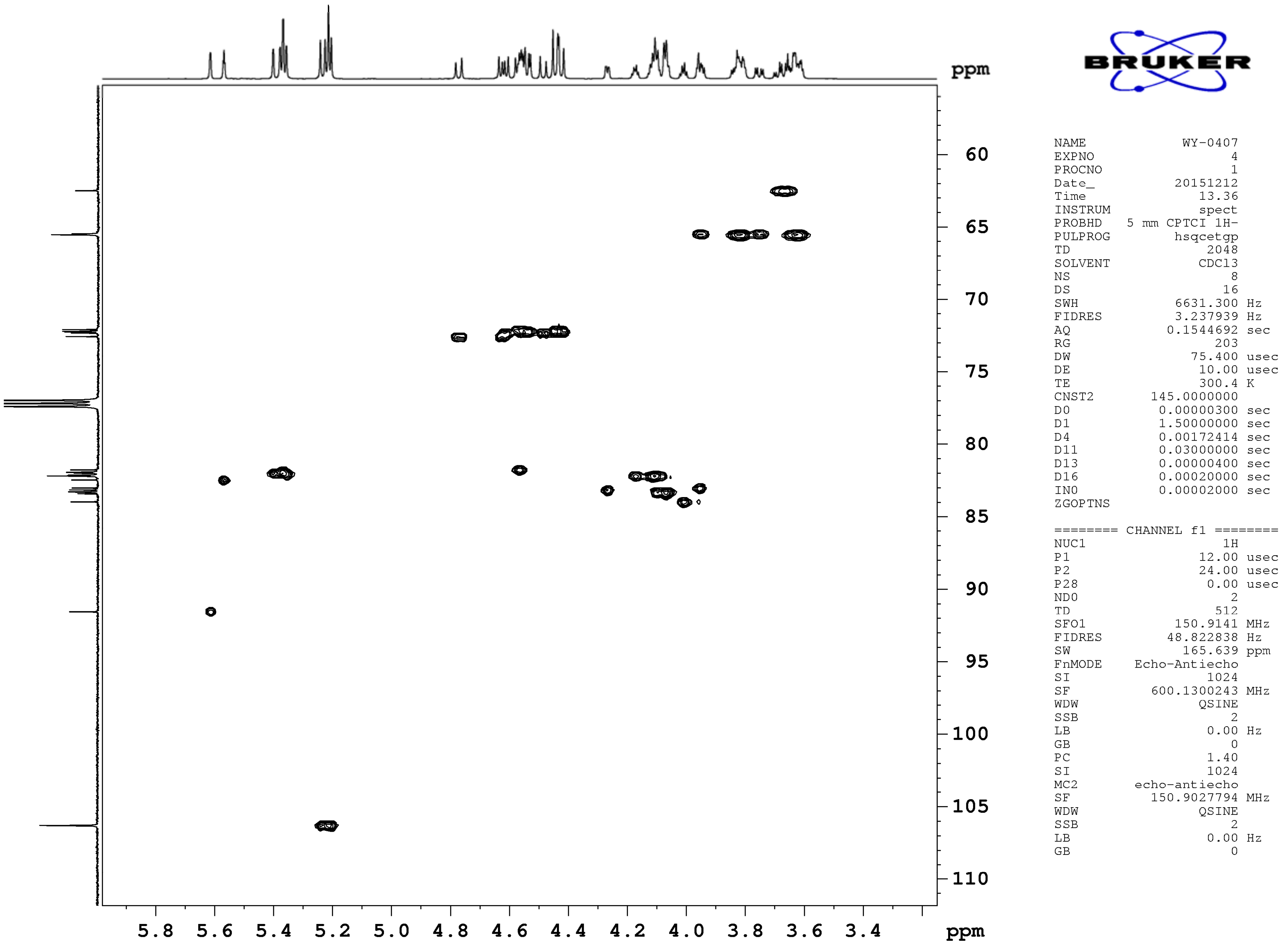

Supplementary Figure 83. HSQC NMR spectrum of compound 17

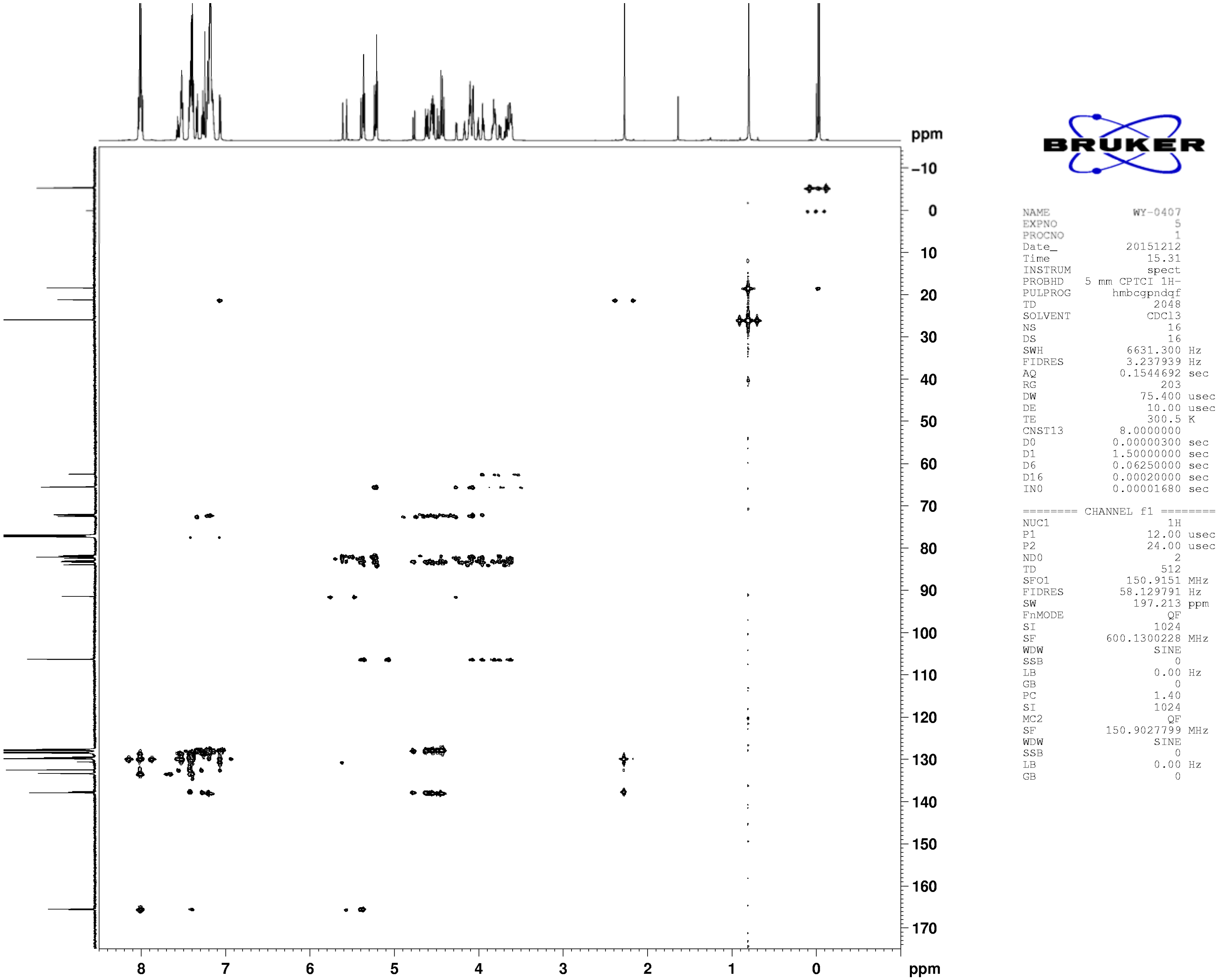

Supplementary Figure 84. HMBC NMR spectrum of compound 17

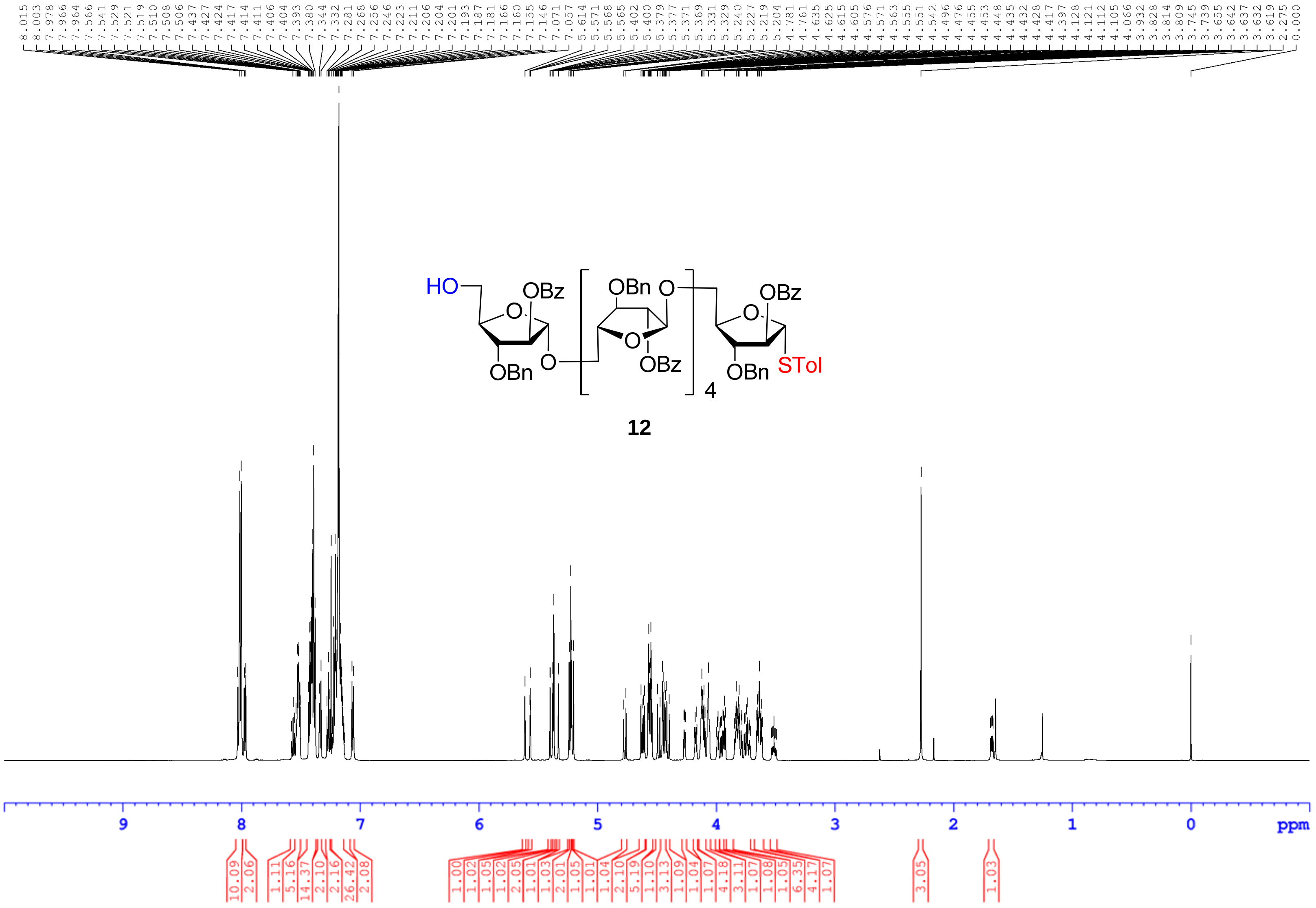

Supplementary Figure 85. ^{1}H NMR spectrum of compound 12

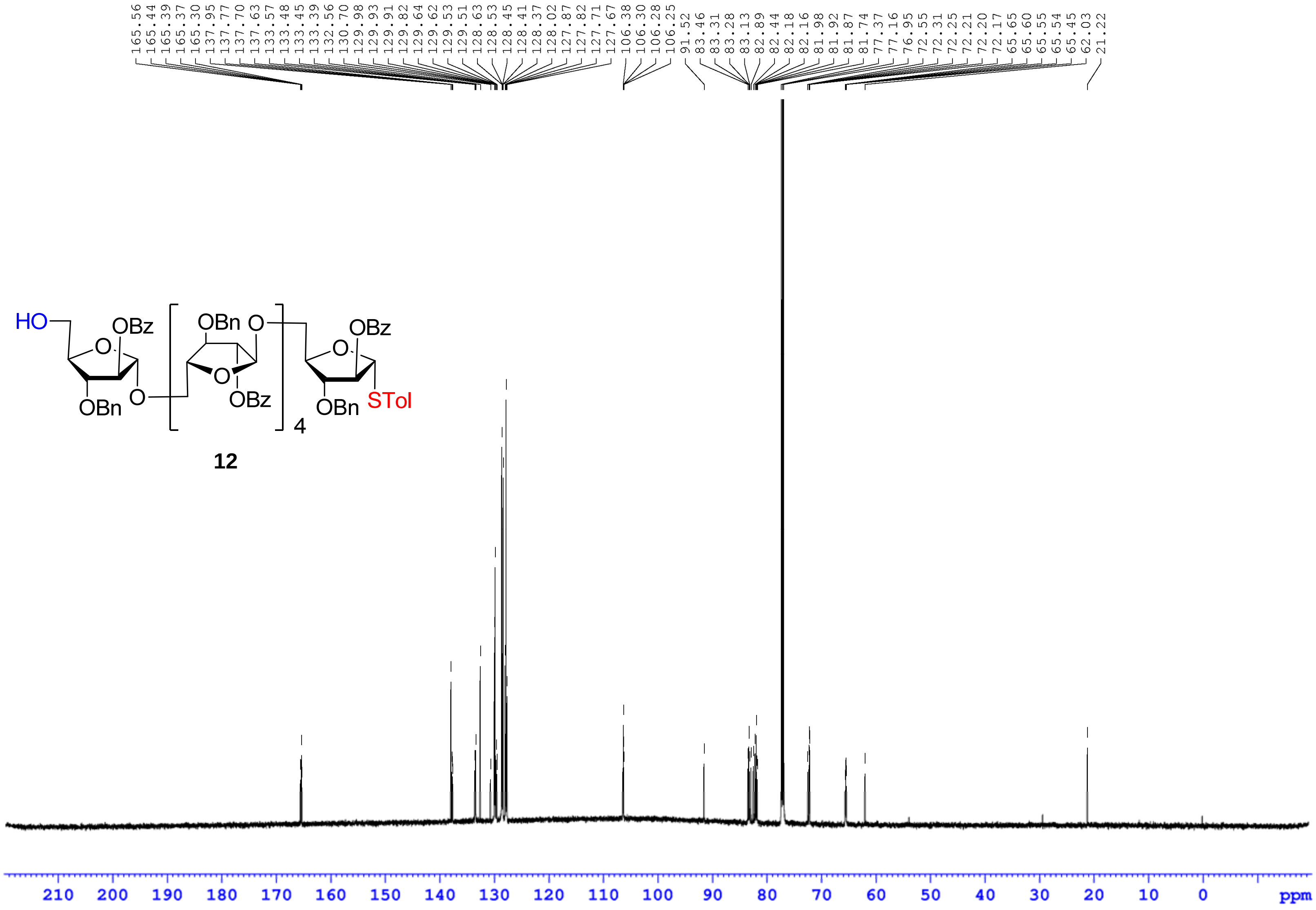

Supplementary Figure 86. ^{13}**C NMR spectrum of compound 12**

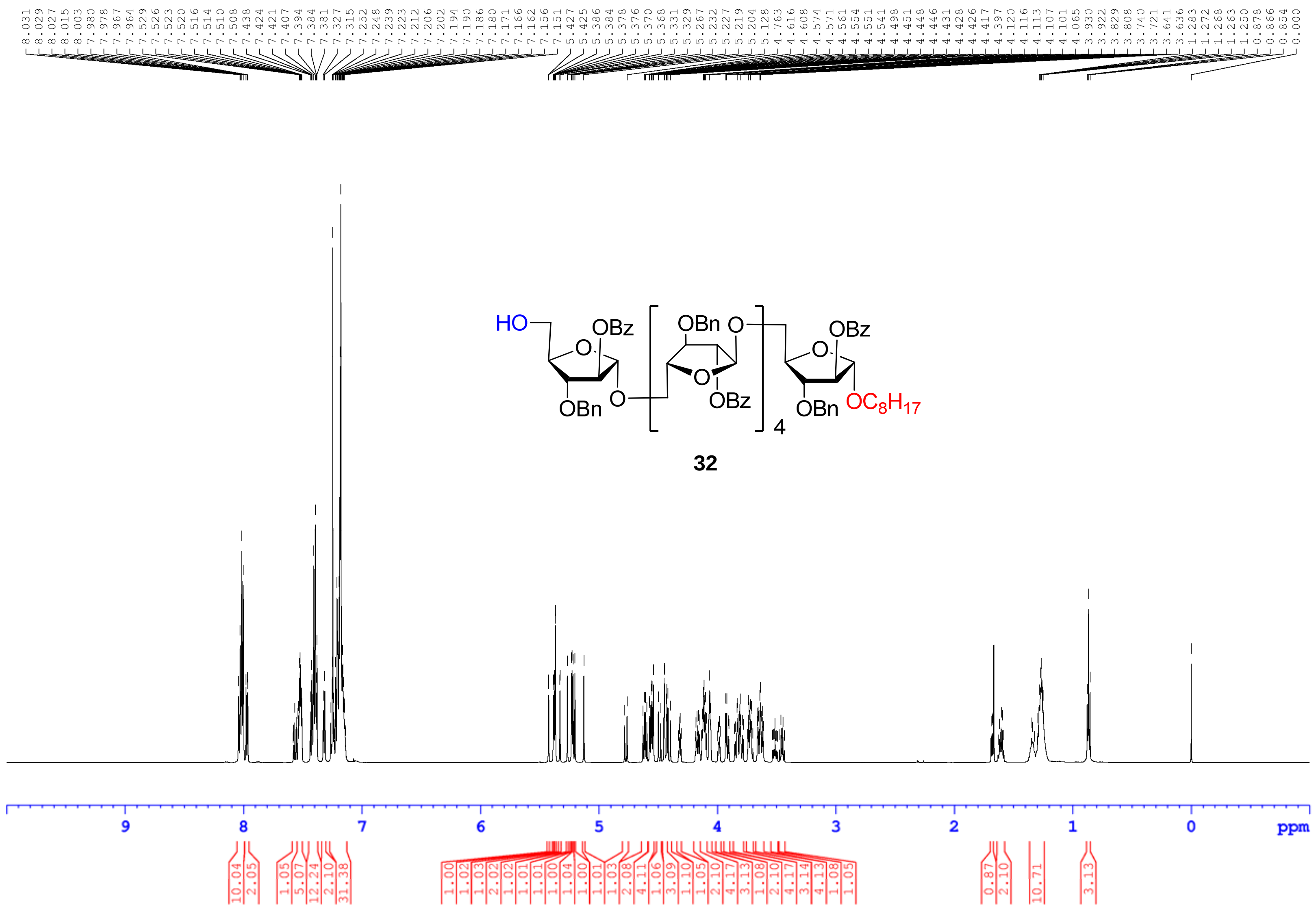

Supplementary Figure 87. ^{1}**H NMR spectrum of compound 32**

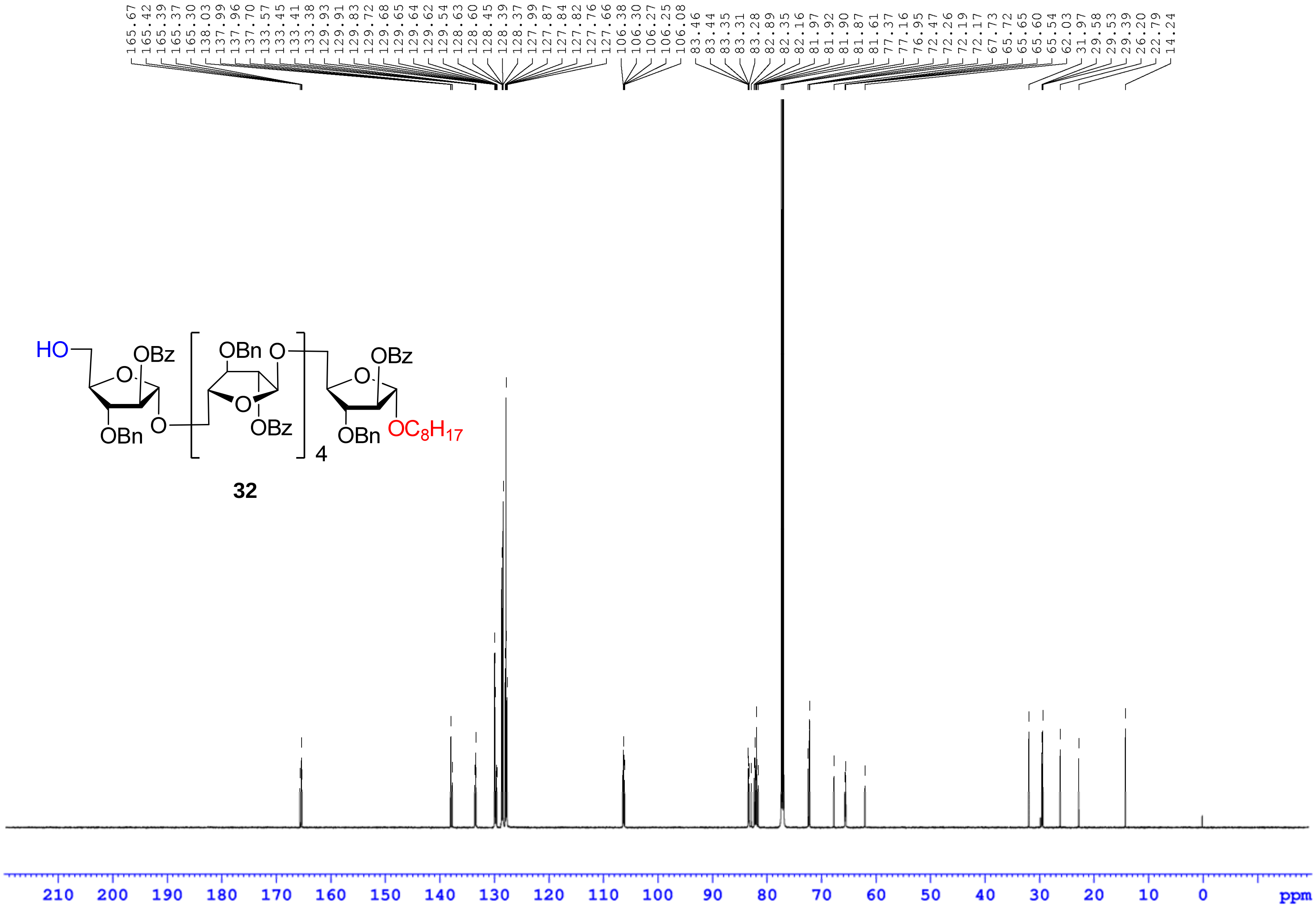

Supplementary Figure 88. ^{13}C NMR spectrum of compound 32

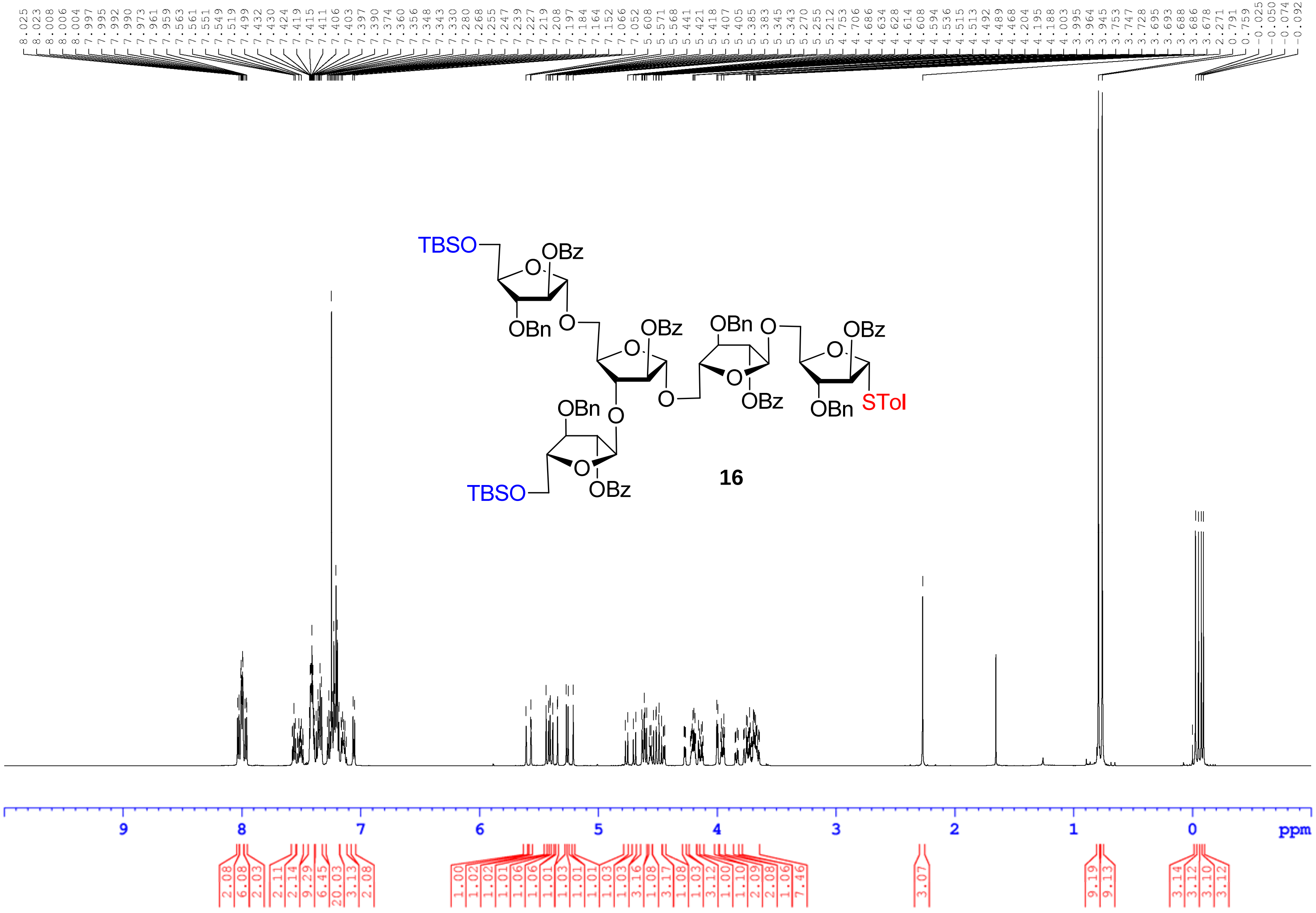

Supplementary Figure 89. ^{1}H NMR spectrum of compound 16

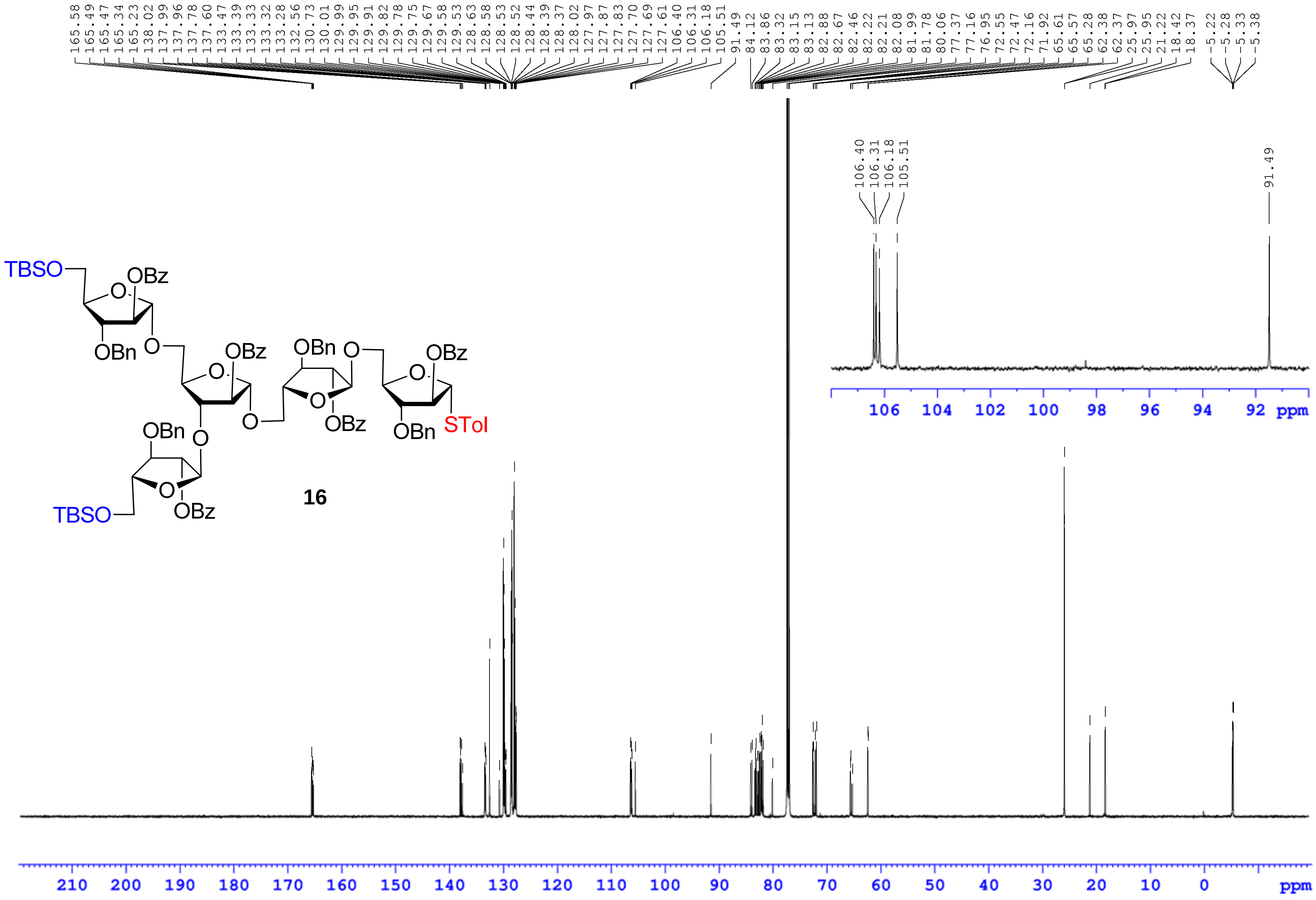

Supplementary Figure 90. ^{13}**C NMR spectrum of compound 16**

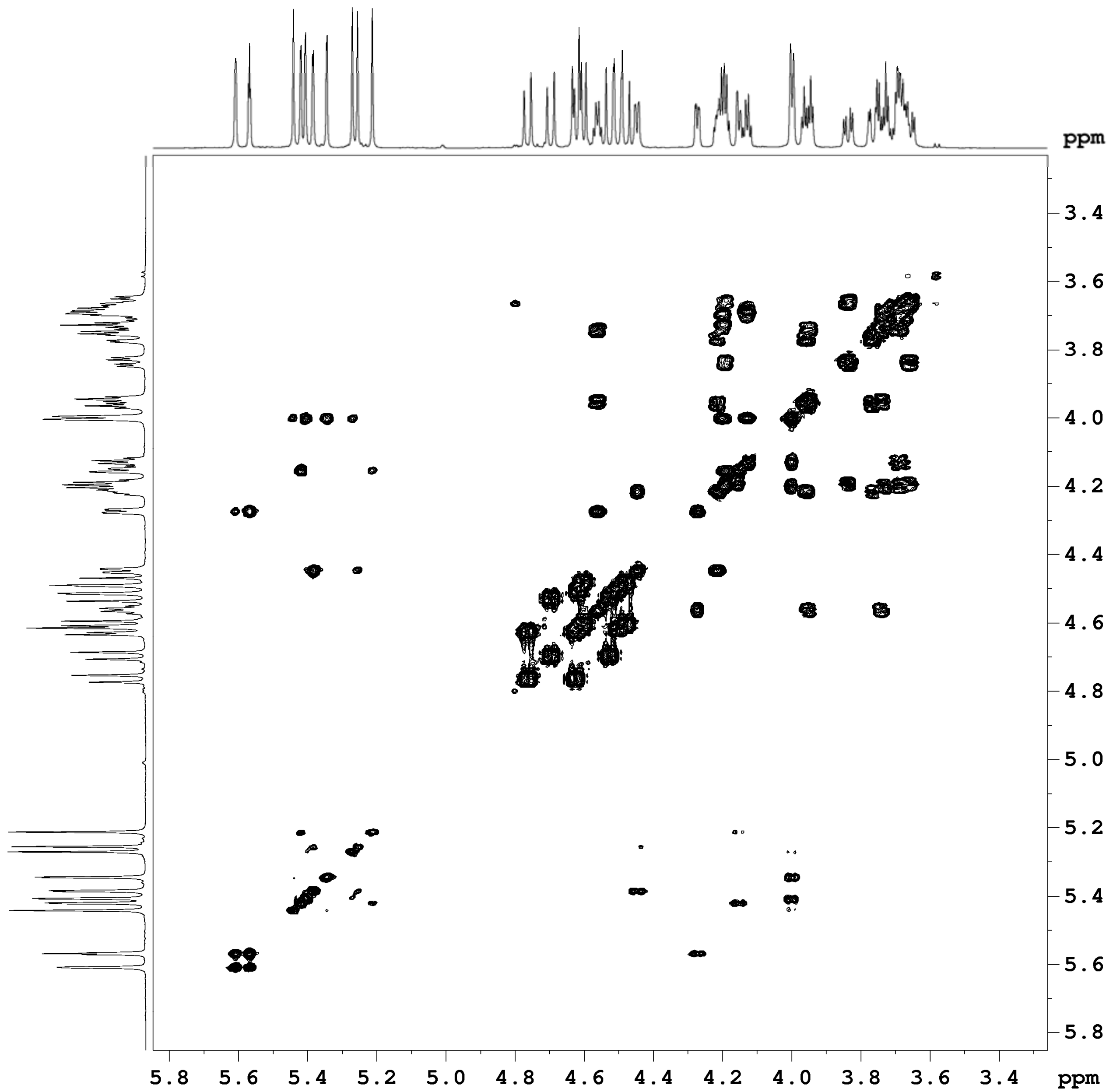


```
NAME              WY-0405
EXPNO                   3
PROCNO                  1
Date_            20151008
Time                10.49
INSTRUM             spect
PROBHD   5 mm CPTCI 1H-
PULPROG        cosygpmfqf
TD                   2048
SOLVENT             CDCl3
NS                      4
DS                      8
SWH             6720.430 Hz
FIDRES          3.281460 Hz
AQ             0.1524212 sec
RG                    203
DW                 74.400 usec
DE                  10.00 usec
TE                  300.4 K
D0            0.00000300 sec
D1            2.00000000 sec
D13           0.00000400 sec
D16           0.00020000 sec
IN0           0.00014880 sec

======== CHANNEL f1 ========
NUC1                   1H
P1                  12.00 usec
ND0                     1
TD                   1024
SFO1             600.1326 MHz
FIDRES           6.562906 Hz
SW                 11.198 ppm
FnMODE                 QF
SI                   2048
SF            600.1300230 MHz
WDW                  SINE
SSB                     0
LB                   0.00 Hz
GB                      0
PC                   1.40
SI                   2048
MC2                    QF
SF            600.1300225 MHz
WDW                  SINE
SSB                     0
LB                   0.00 Hz
GB                      0
```

Supplementary Figure 91. COSY NMR spectrum of compound 16

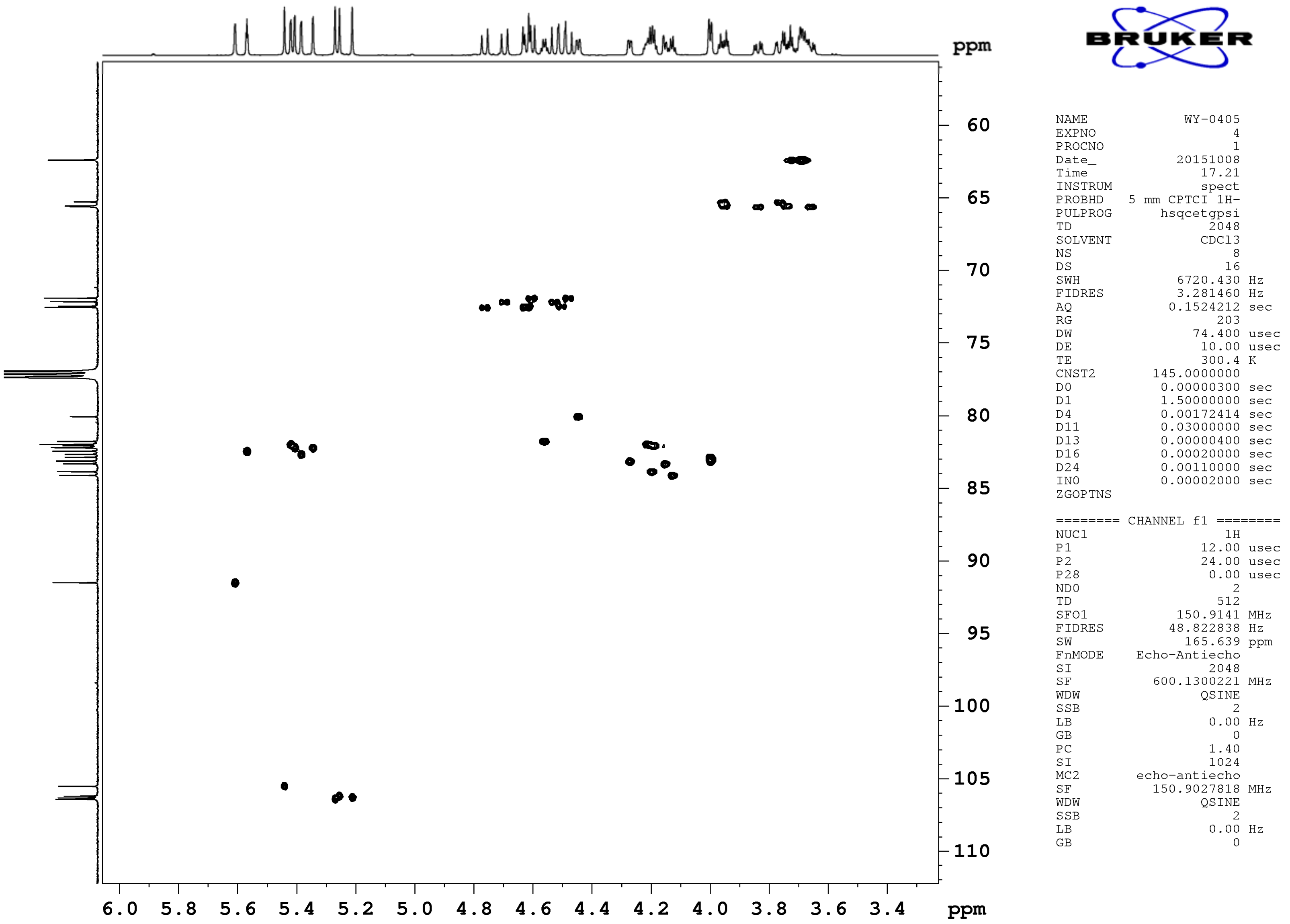

Supplementary Figure 92. HSQC NMR spectrum of compound 16

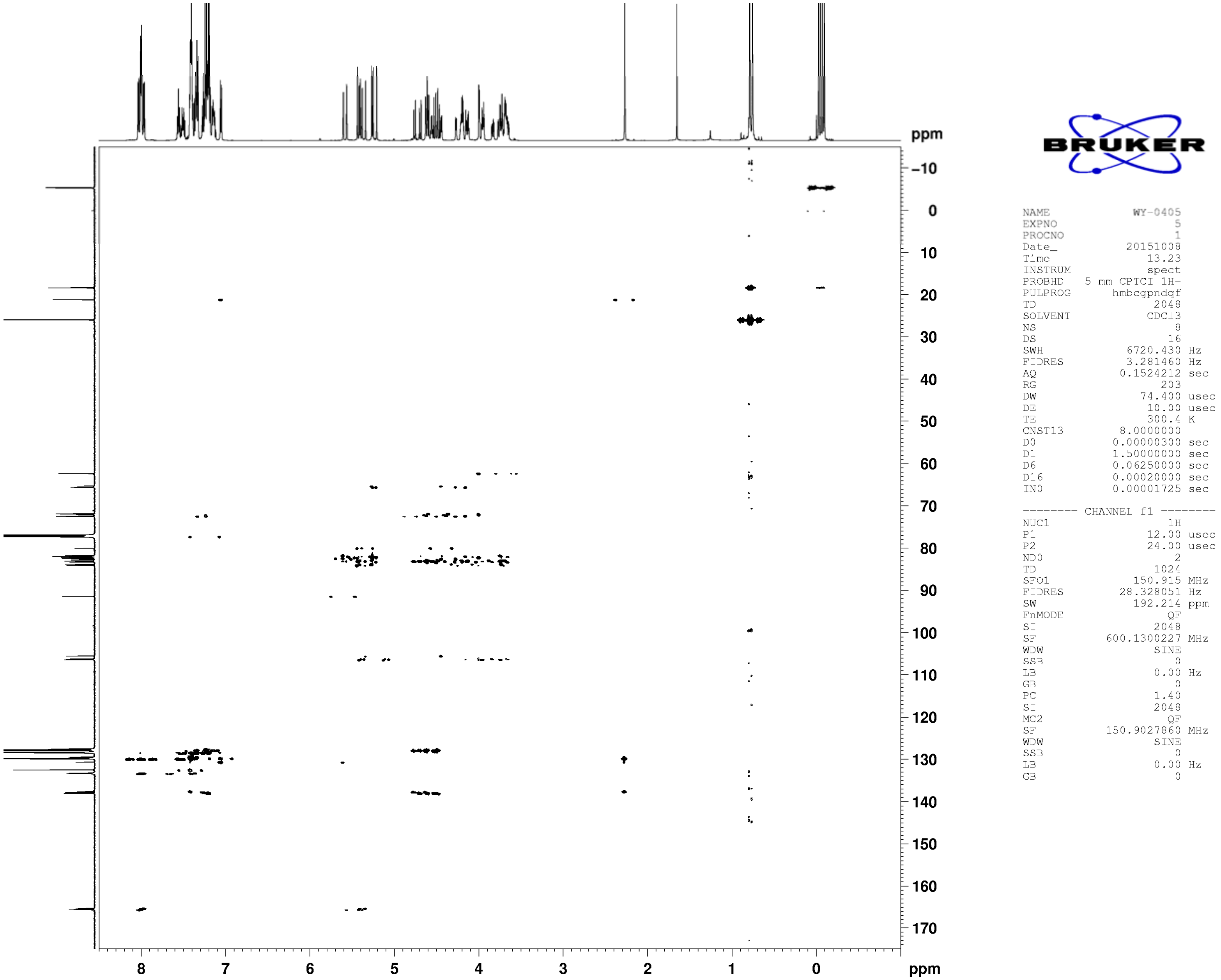

Supplementary Figure 93. HMBC NMR spectrum of compound 16

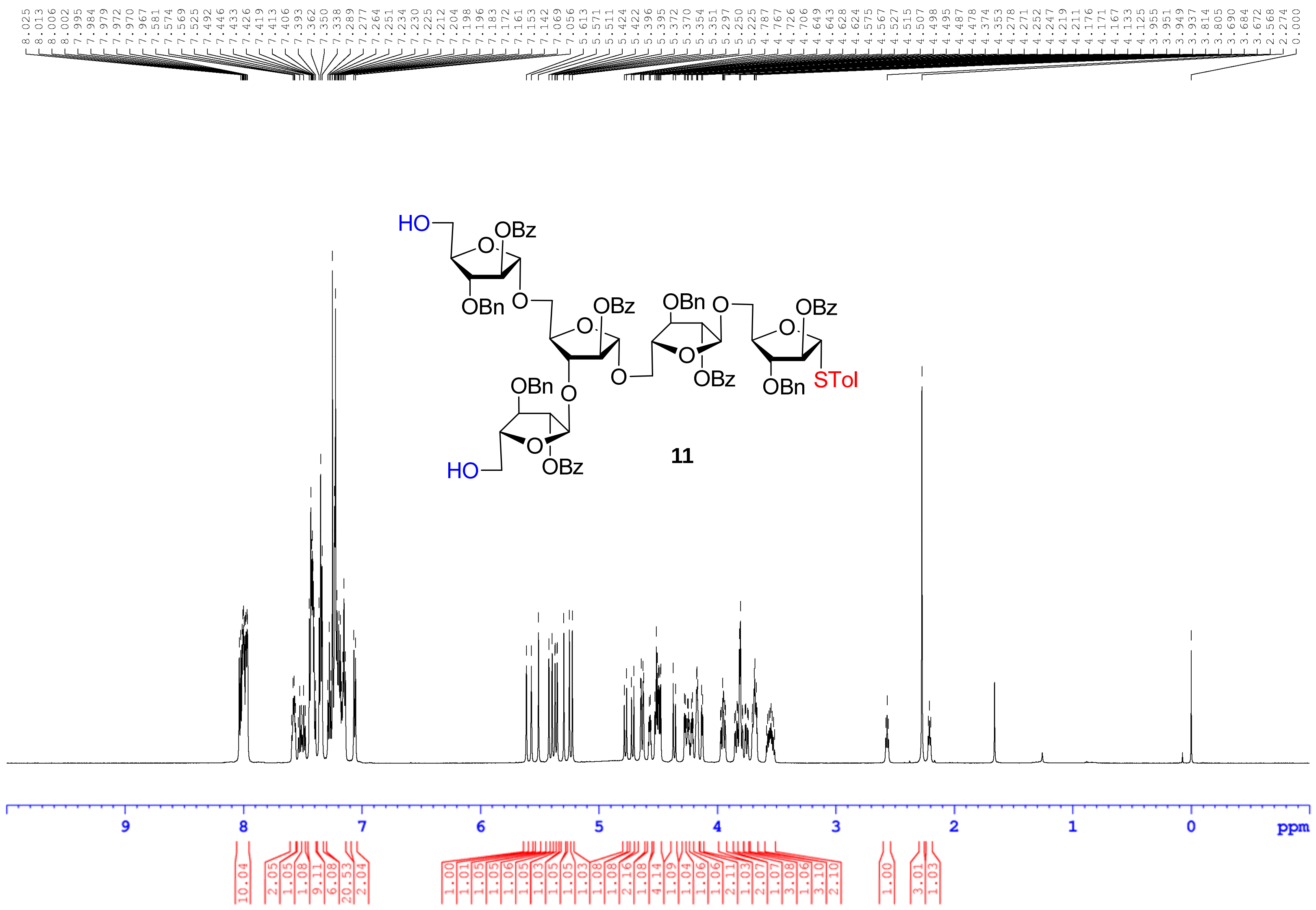

Supplementary Figure 94. ^{1}H NMR spectrum of compound 11

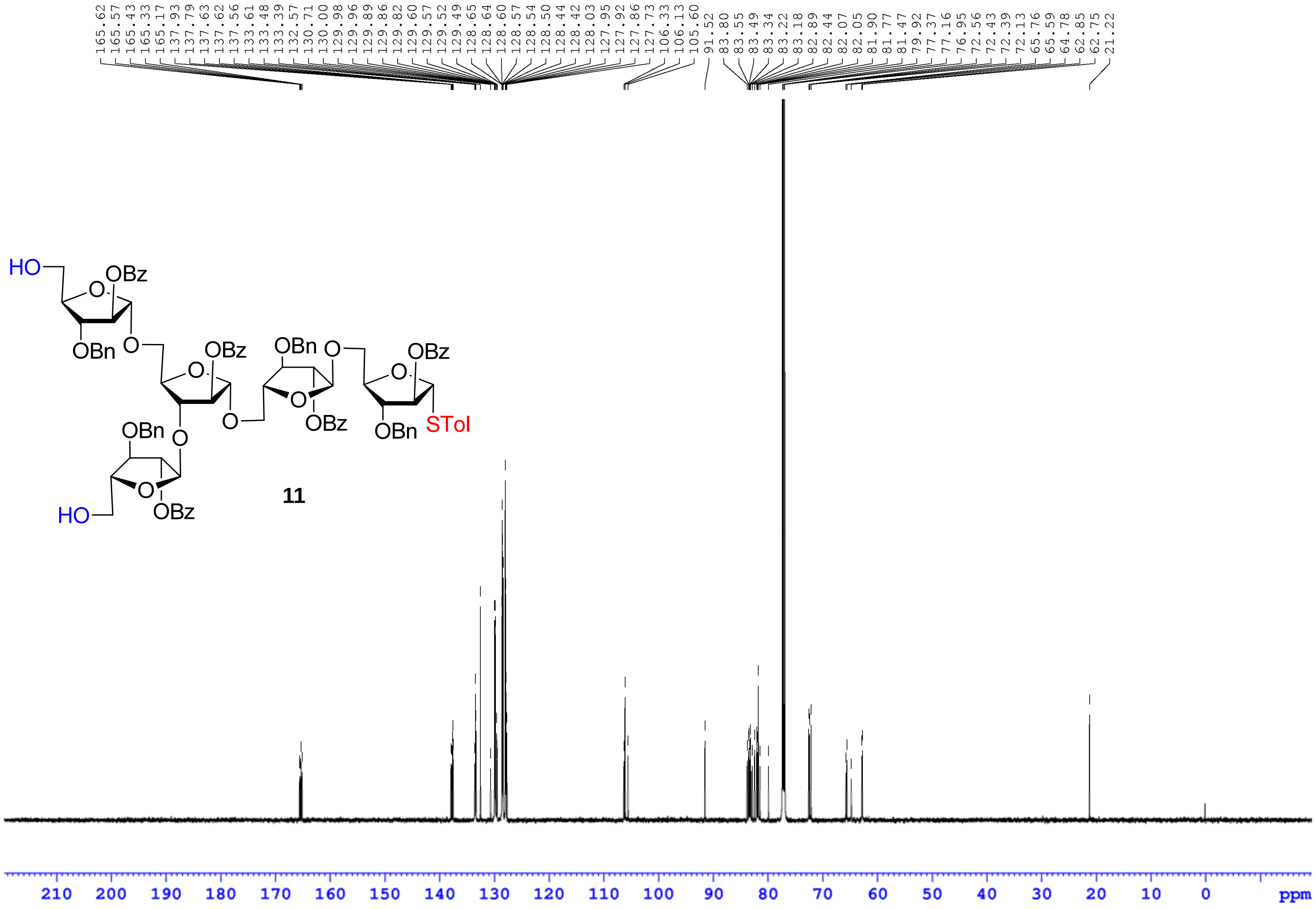

Supplementary Figure 95. ^{13}C NMR spectrum of compound 11

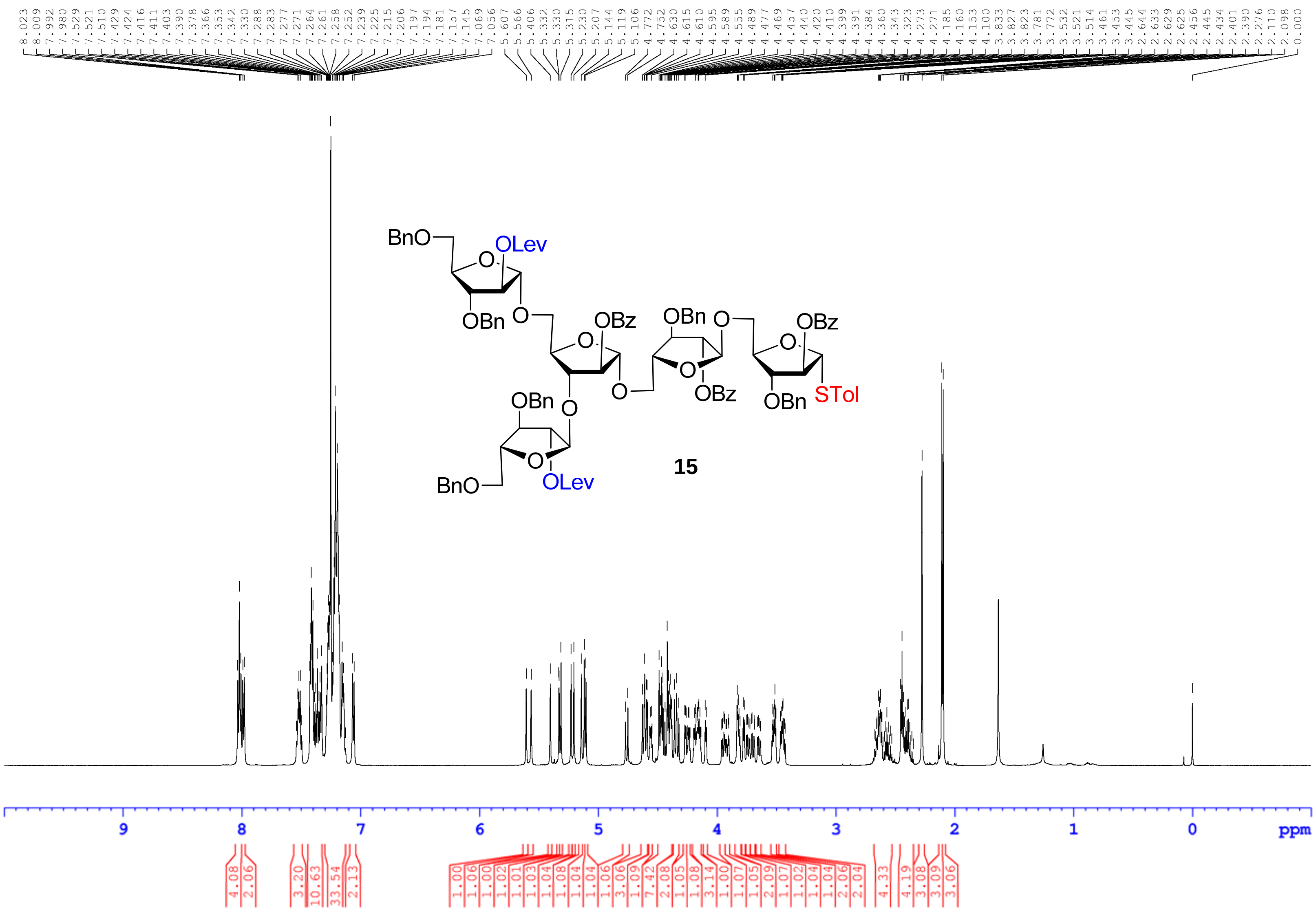

Supplementary Figure 96. ^{1}H NMR spectrum of compound 15

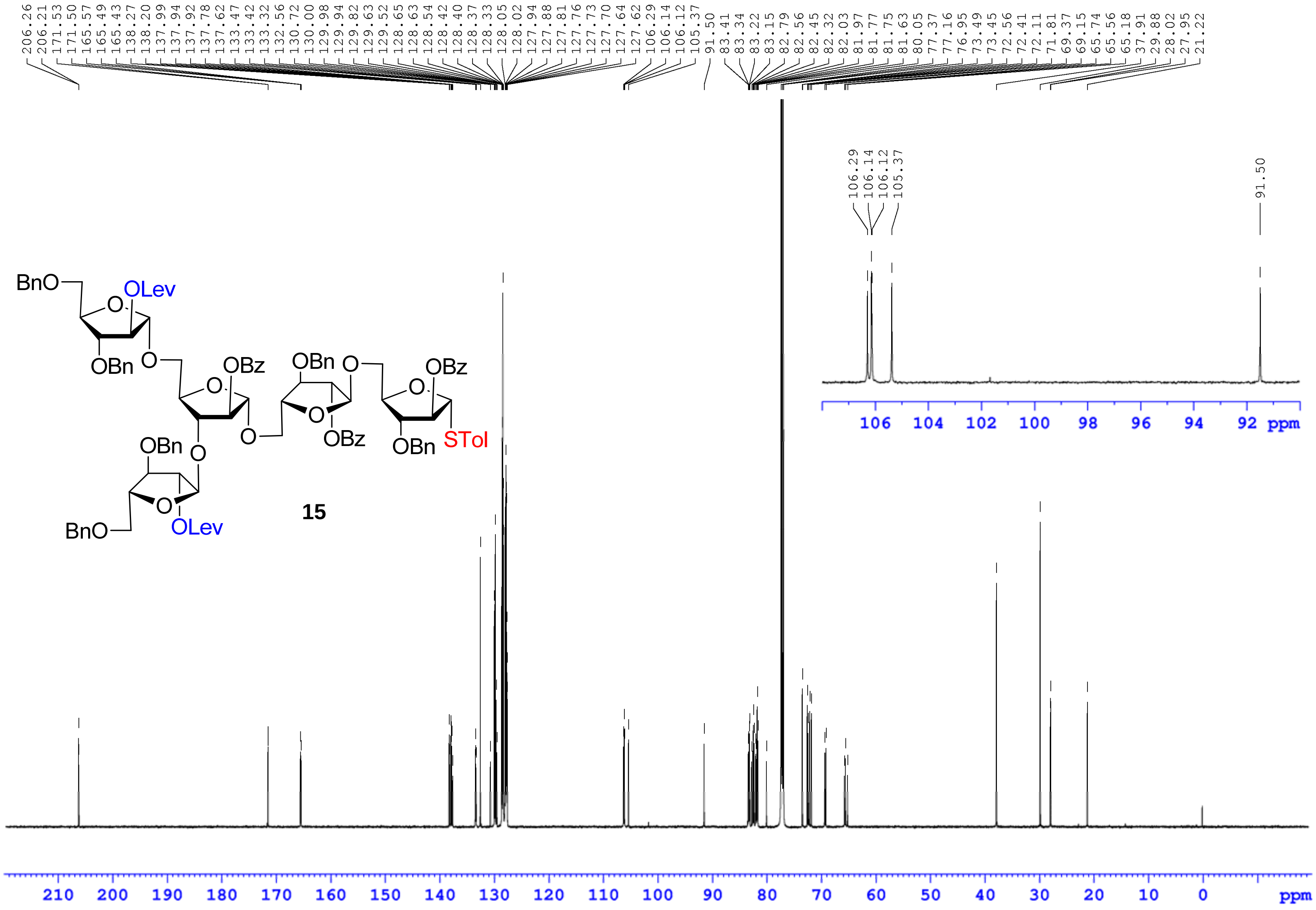

Supplementary Figure 97. ^{13}**C NMR spectrum of compound 15**

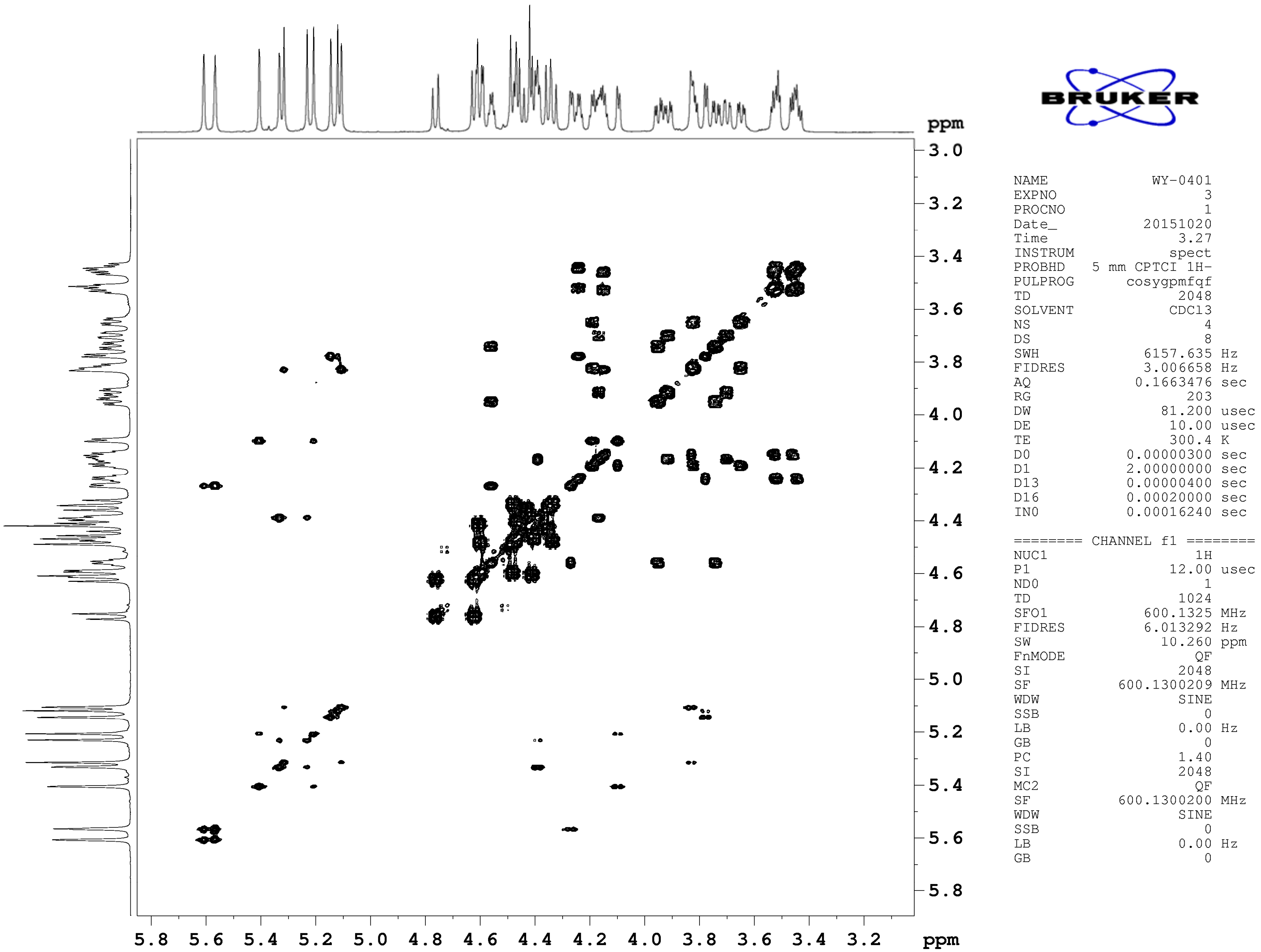

Supplementary Figure 98. COSY NMR spectrum of compound 15

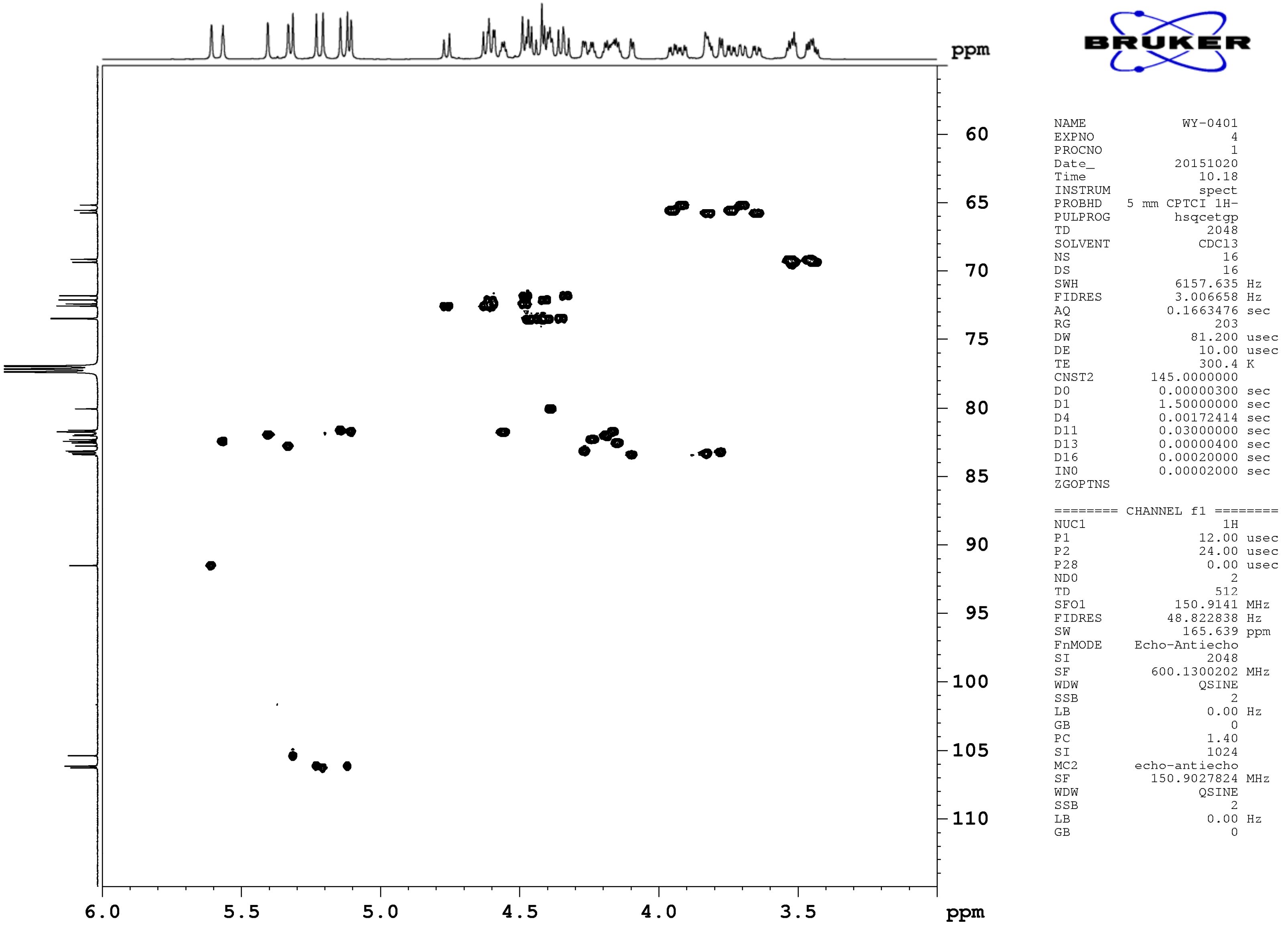

Supplementary Figure 99. HSQC NMR spectrum of compound 15

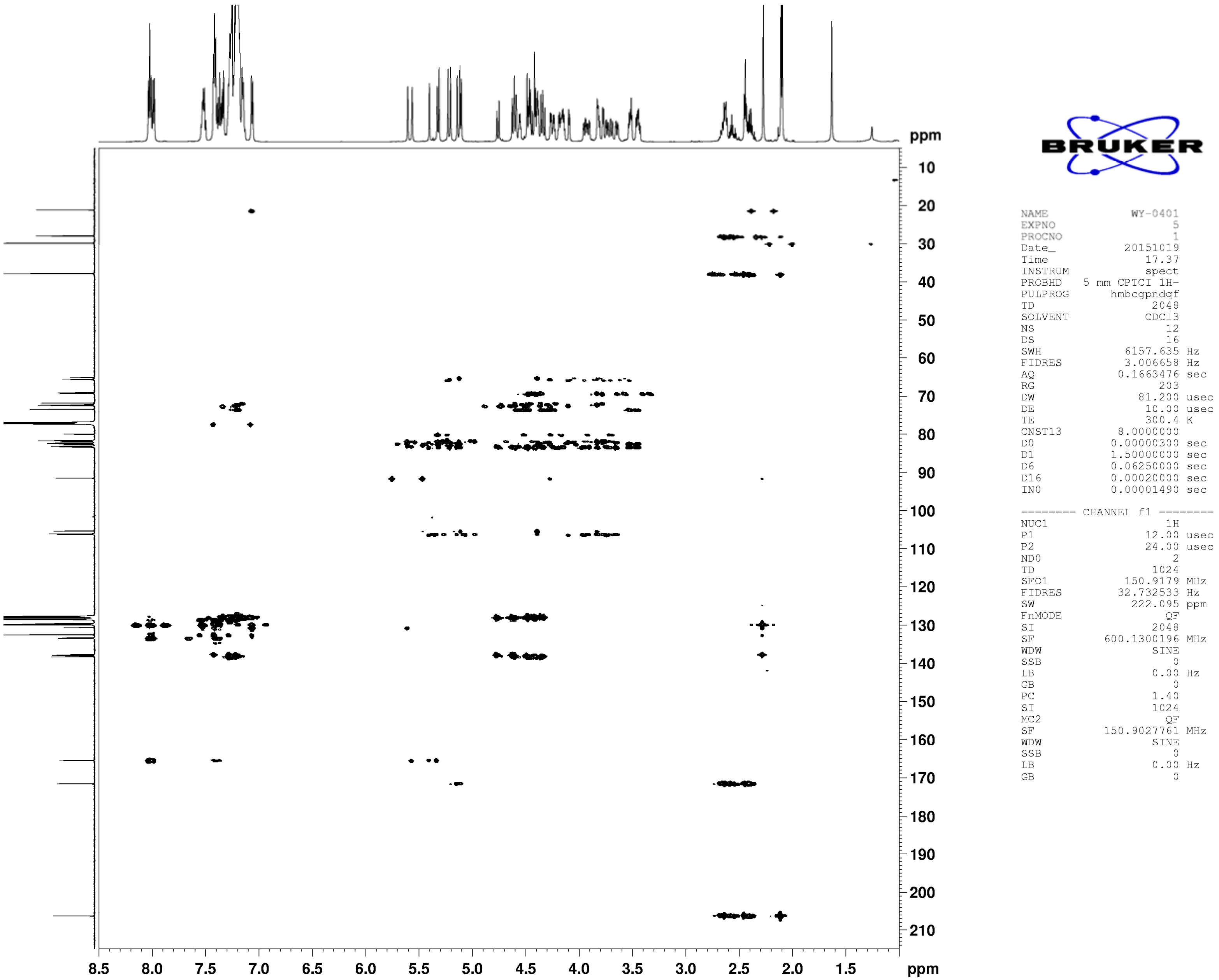

Supplementary Figure 100. HMBC NMR spectrum of compound 15

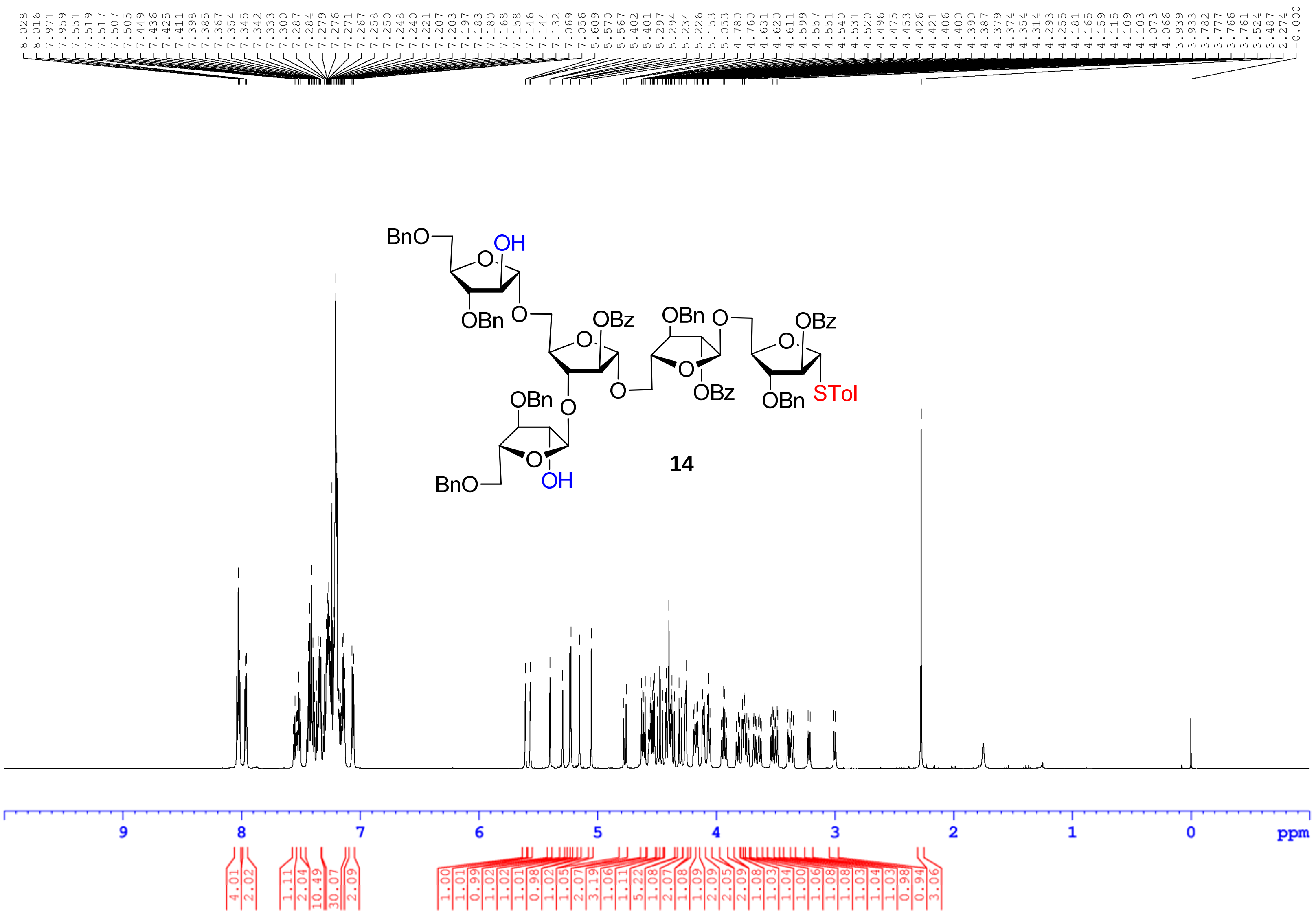

Supplementary Figure 101. ^{1}H NMR spectrum of compound 14

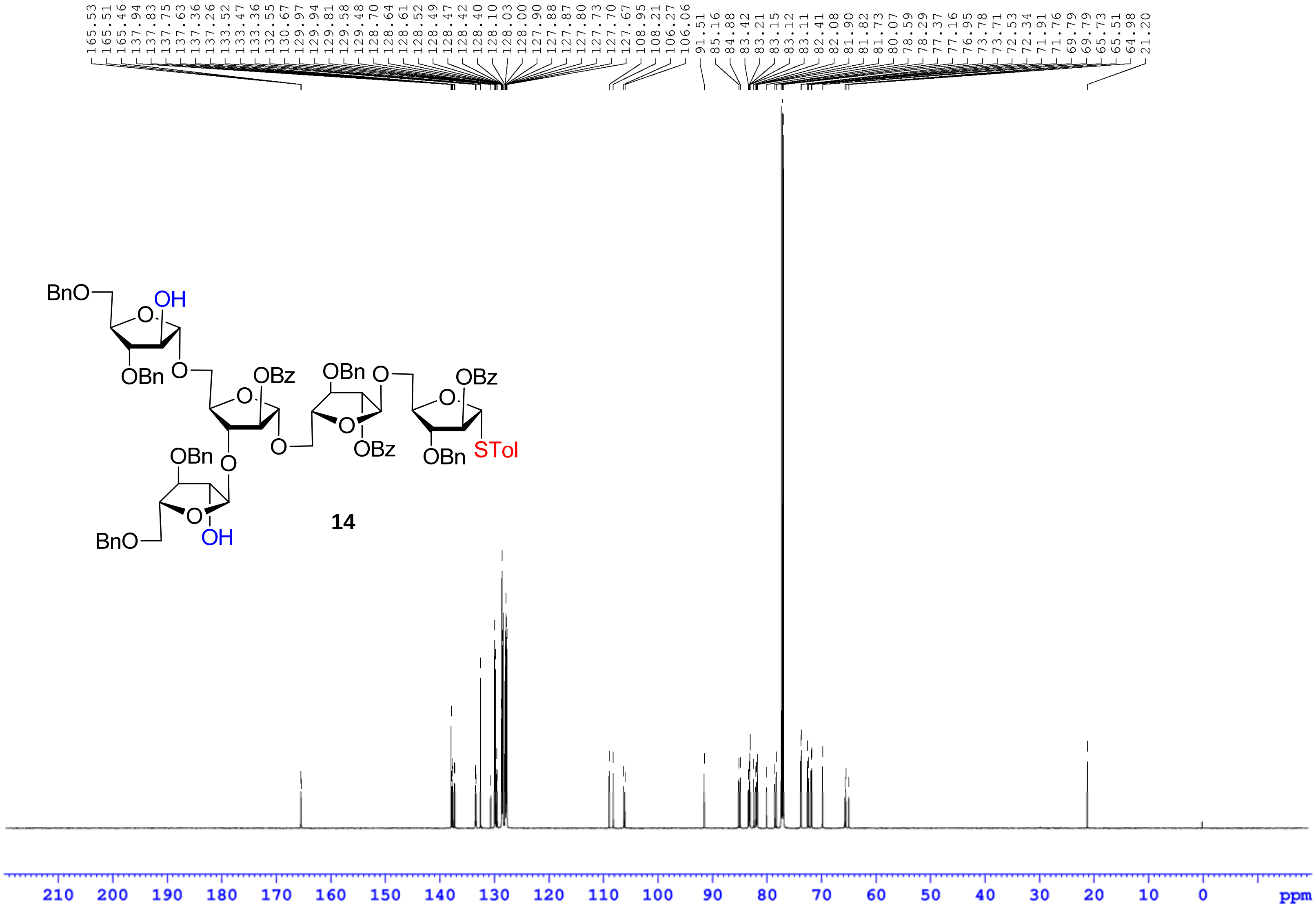

Supplementary Figure 102. ^{13}**C NMR spectrum of compound 14**

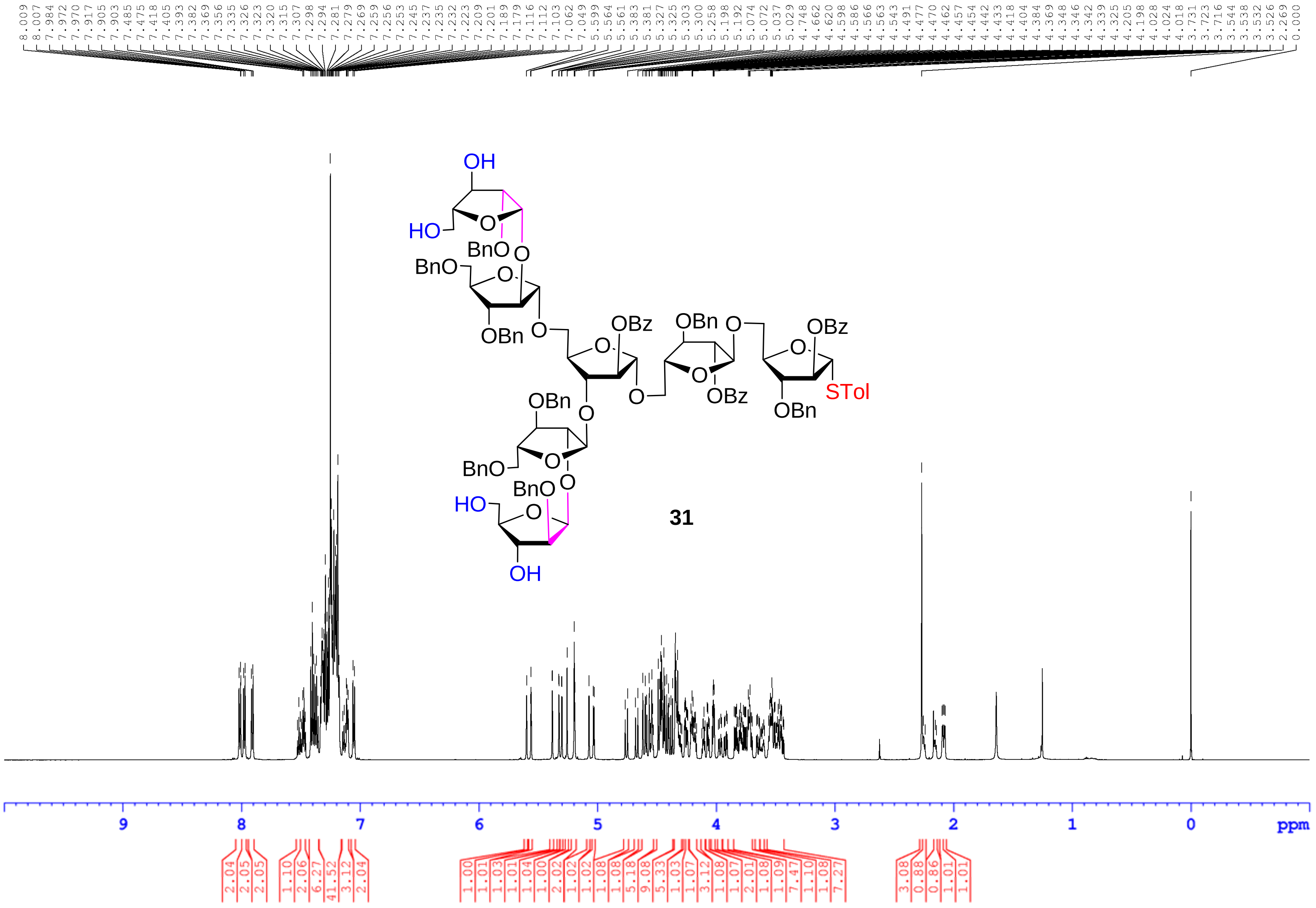

Supplementary Figure 103. ^{1}H NMR spectrum of compound 31

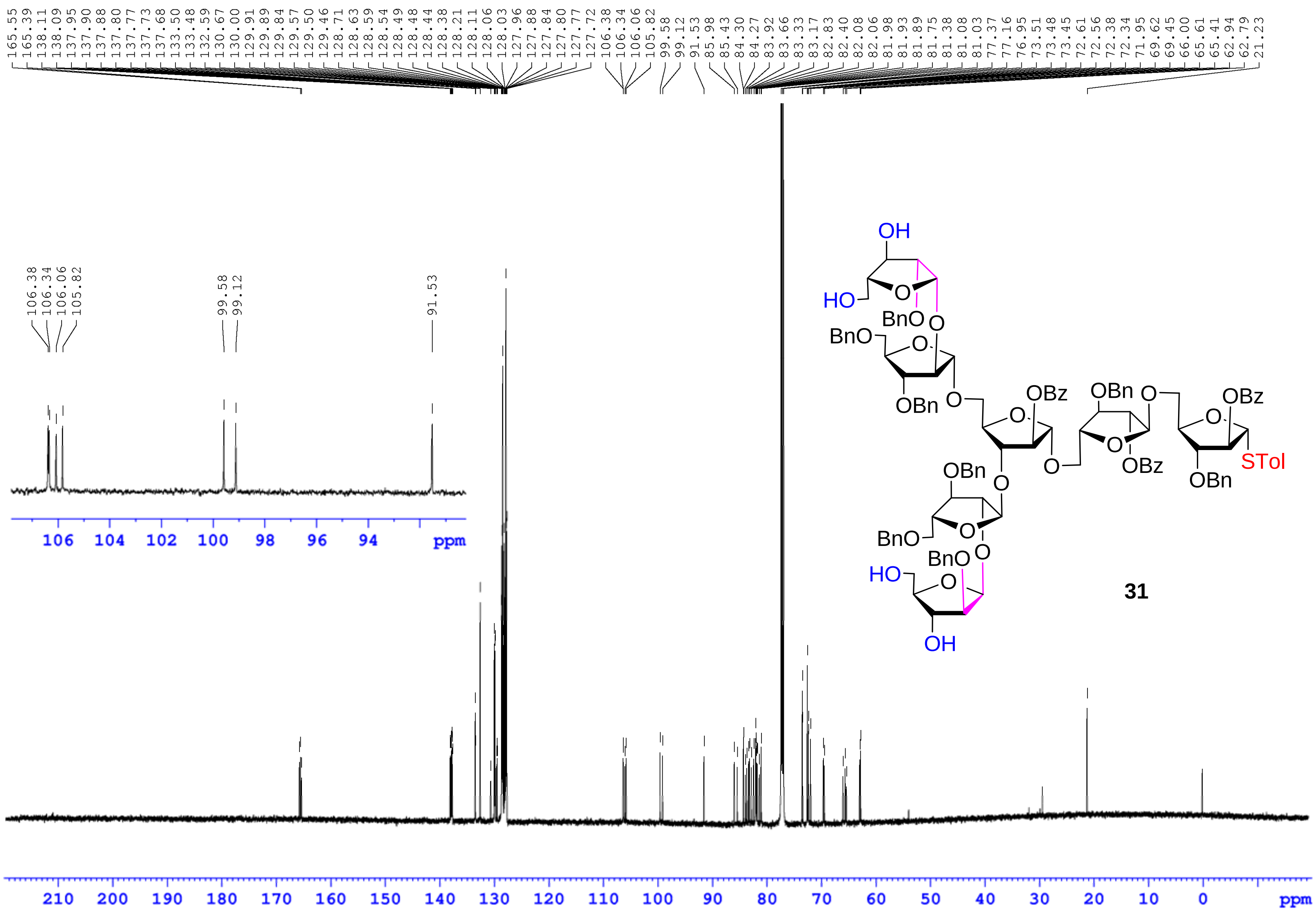

31

Supplementary Figure 104. ^{13}C NMR spectrum of compound 31

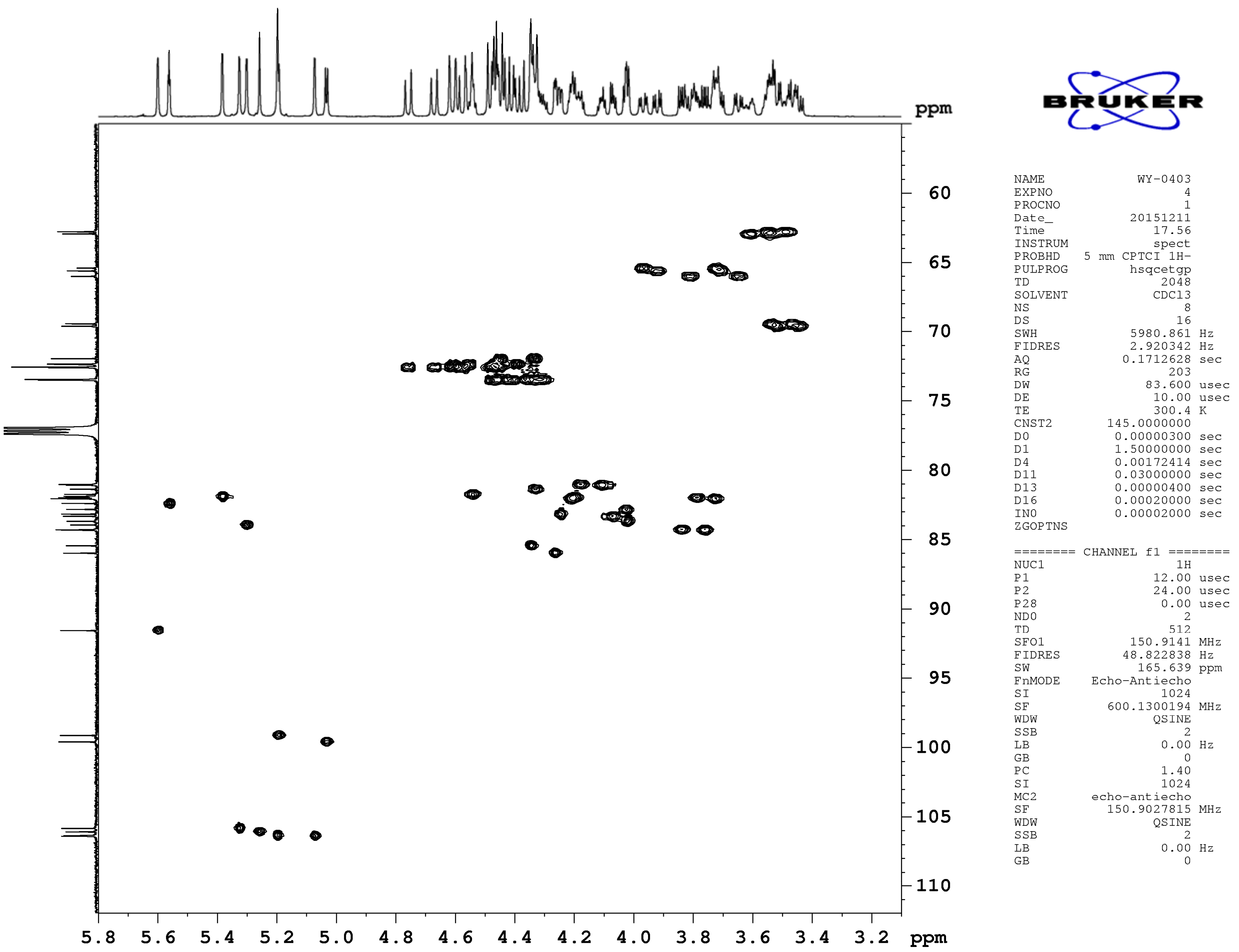

Supplementary Figure 105. HSQC NMR spectrum of compound 31

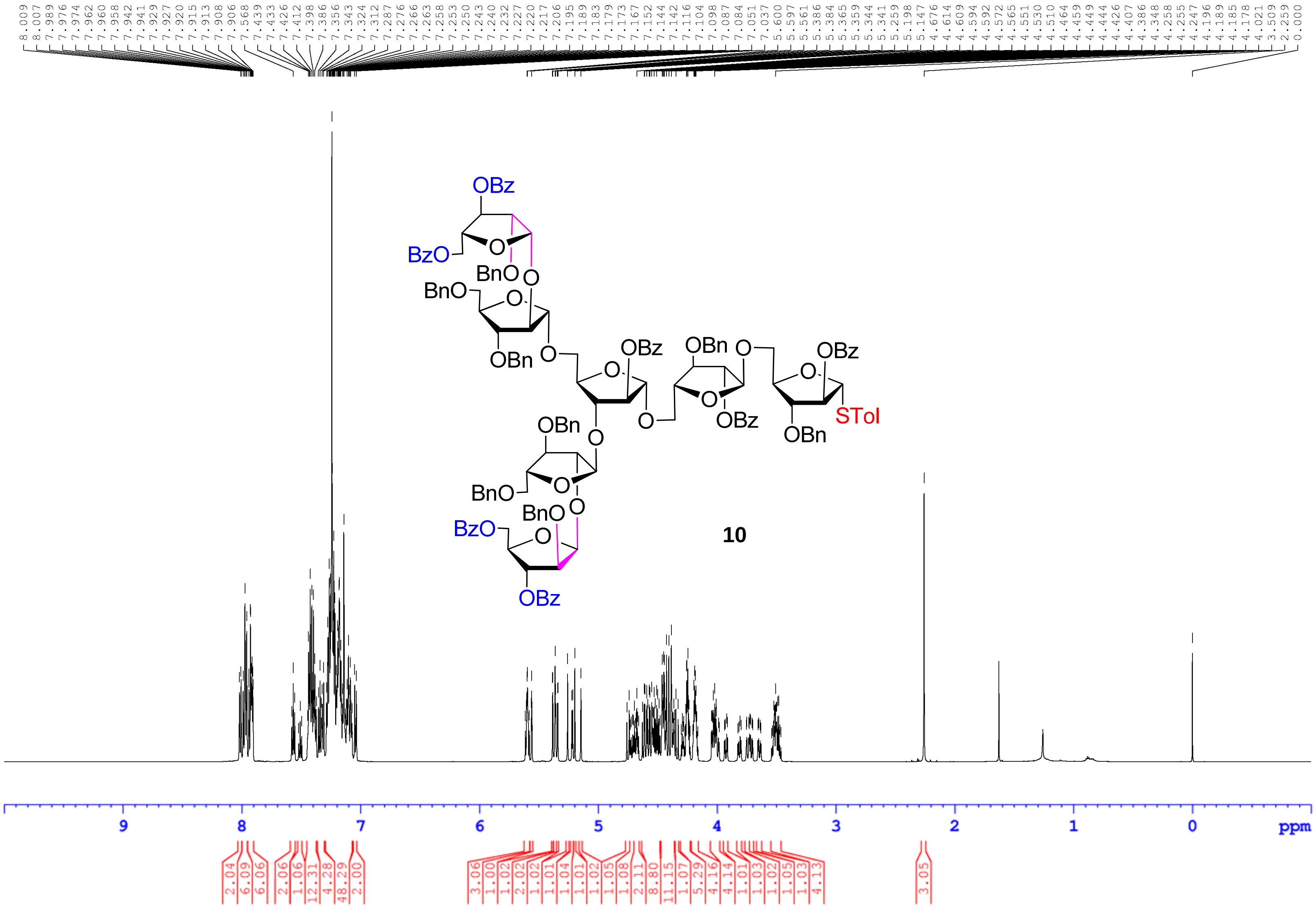

Supplementary Figure 106. ^{1}H NMR spectrum of compound 10

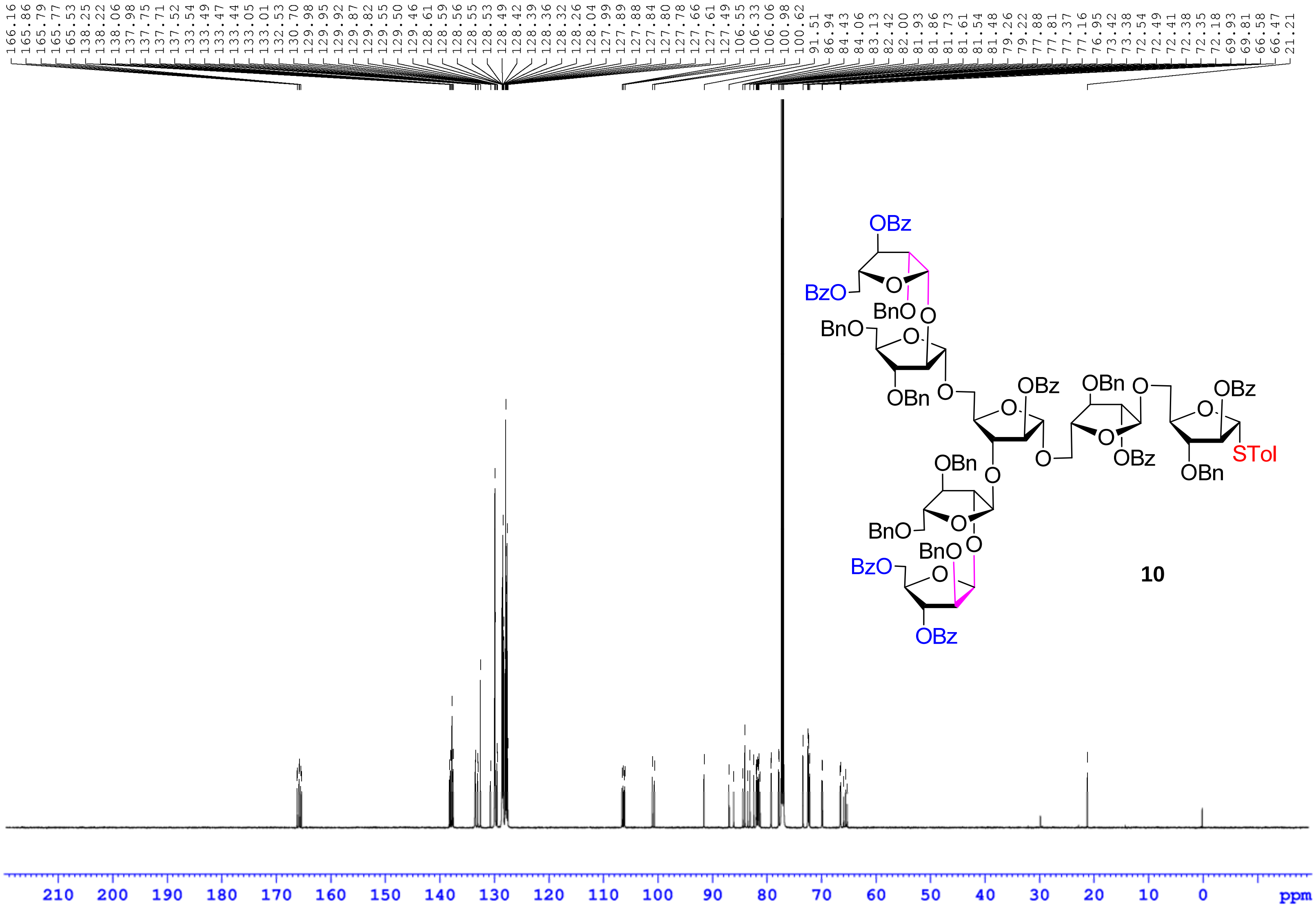

Supplementary Figure 107. ^{13}**C NMR spectrum of compound 10**

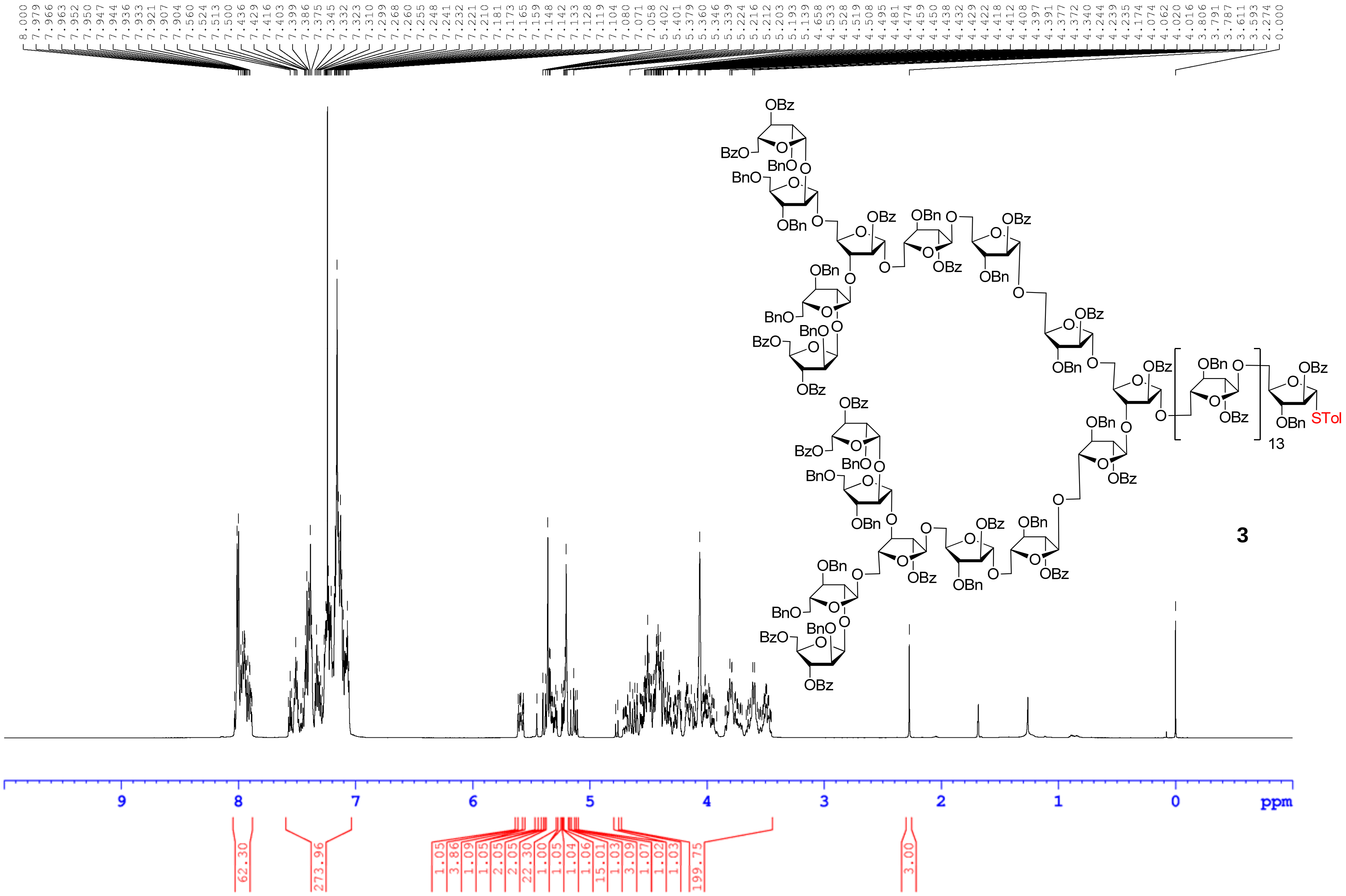

Supplementary Figure 108. ^{1}**H NMR spectrum of compound 3**

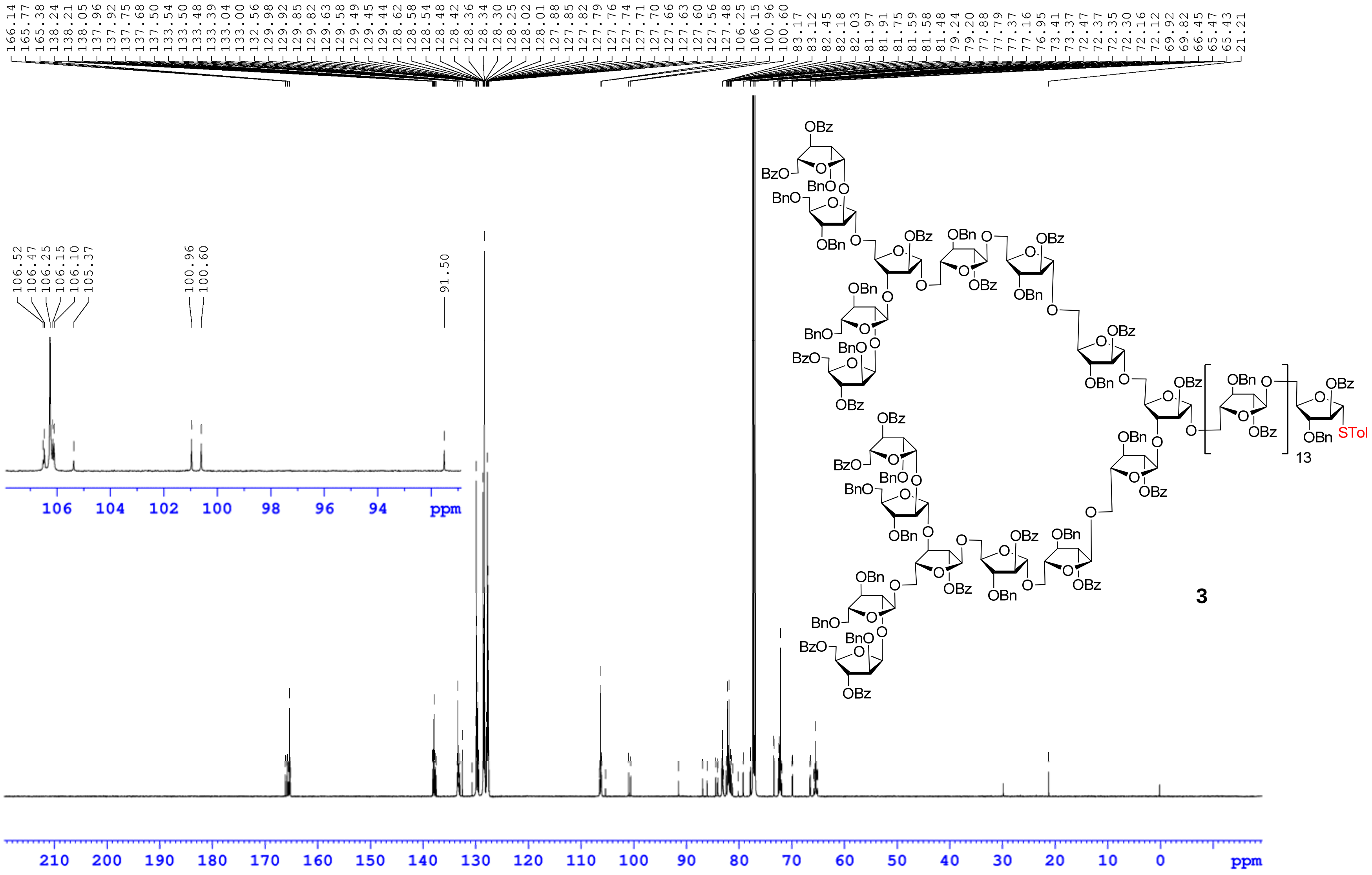

Supplementary Figure 109. ^{13}C NMR spectrum of compound 3

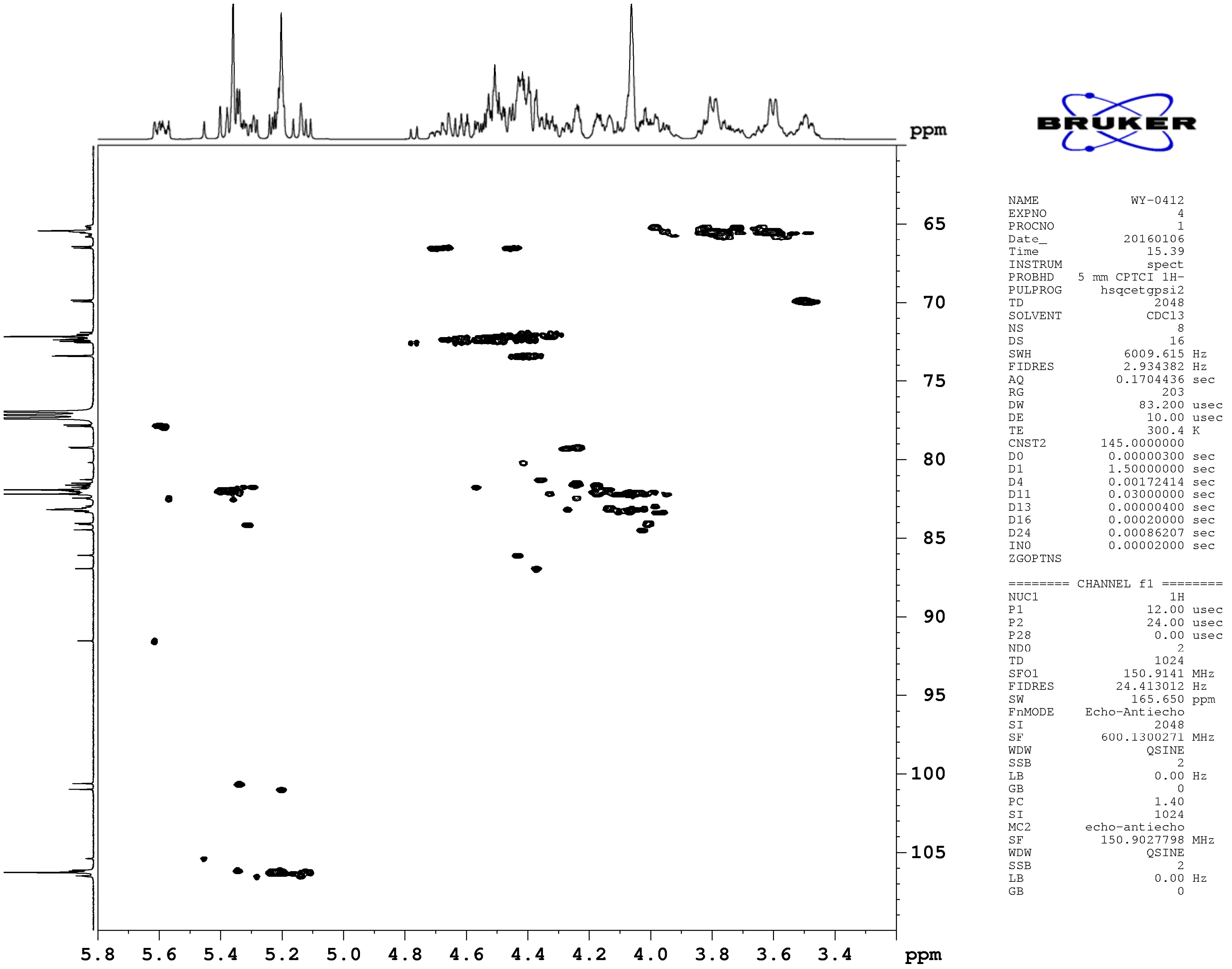

Supplementary Figure 110. HSQC NMR spectrum of compound 3

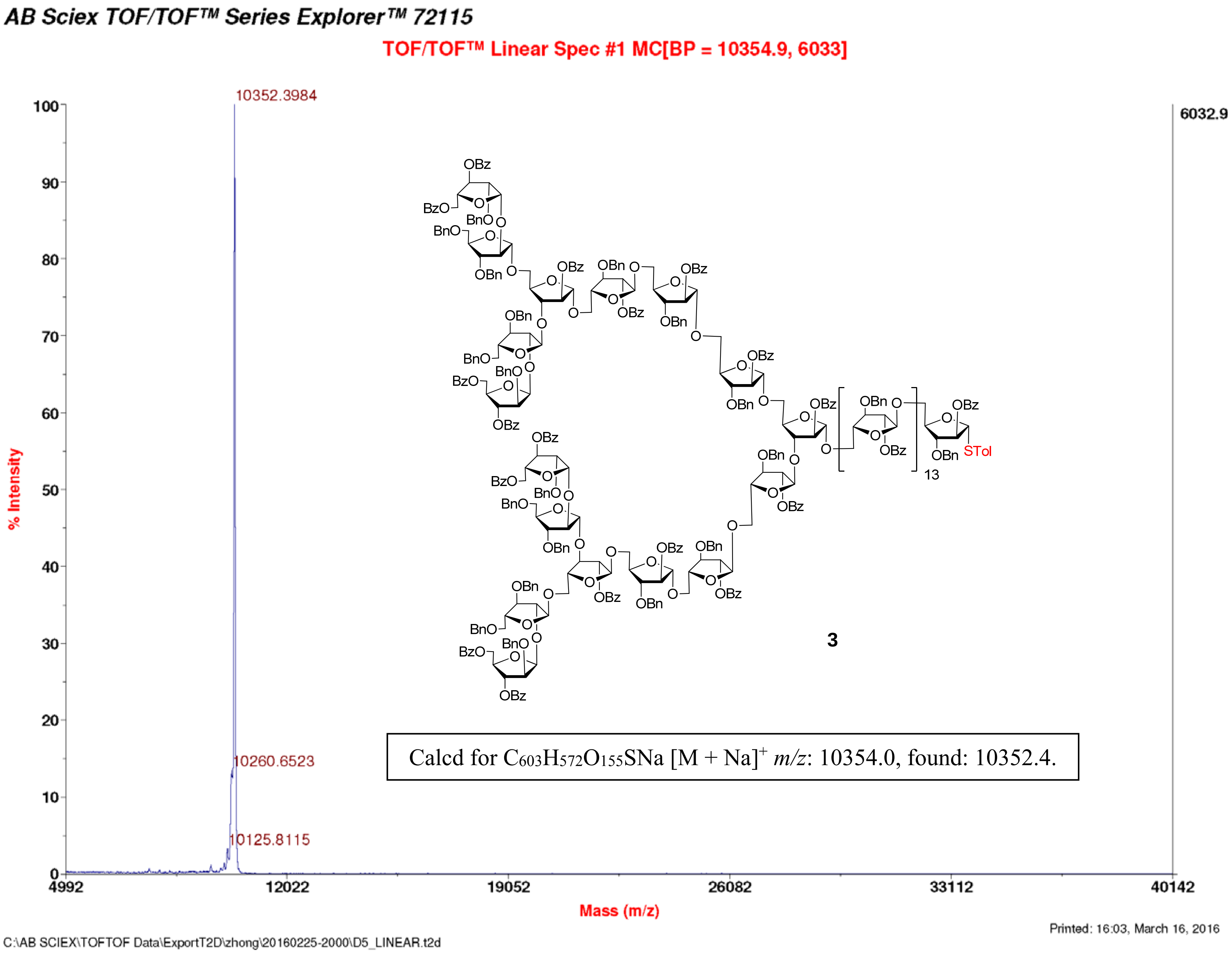

Supplementary Figure 111. MALDI-TOF MS spectrum of compound 3

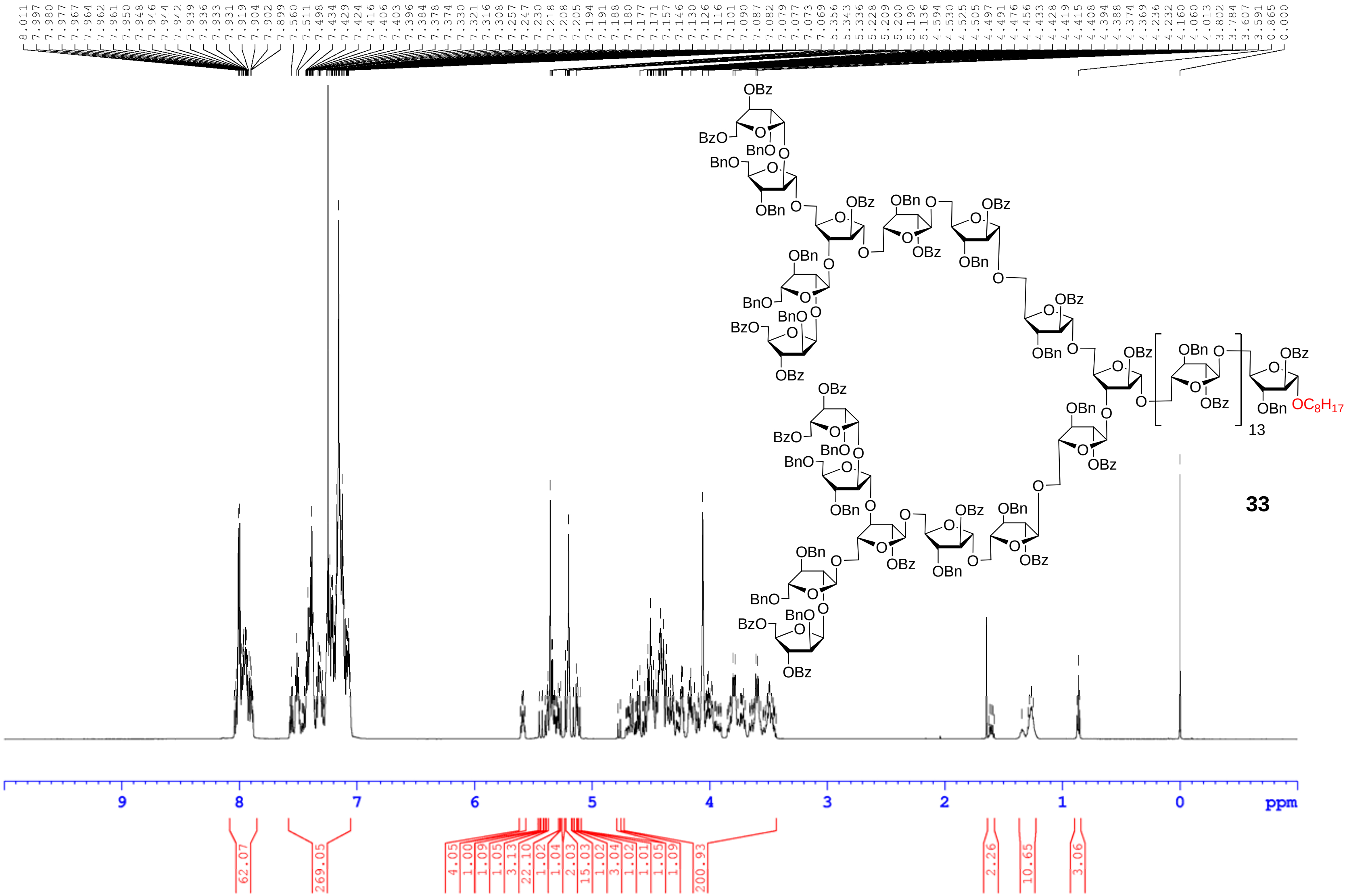

33

Supplementary Figure 112. ^1H NMR spectrum of compound 33

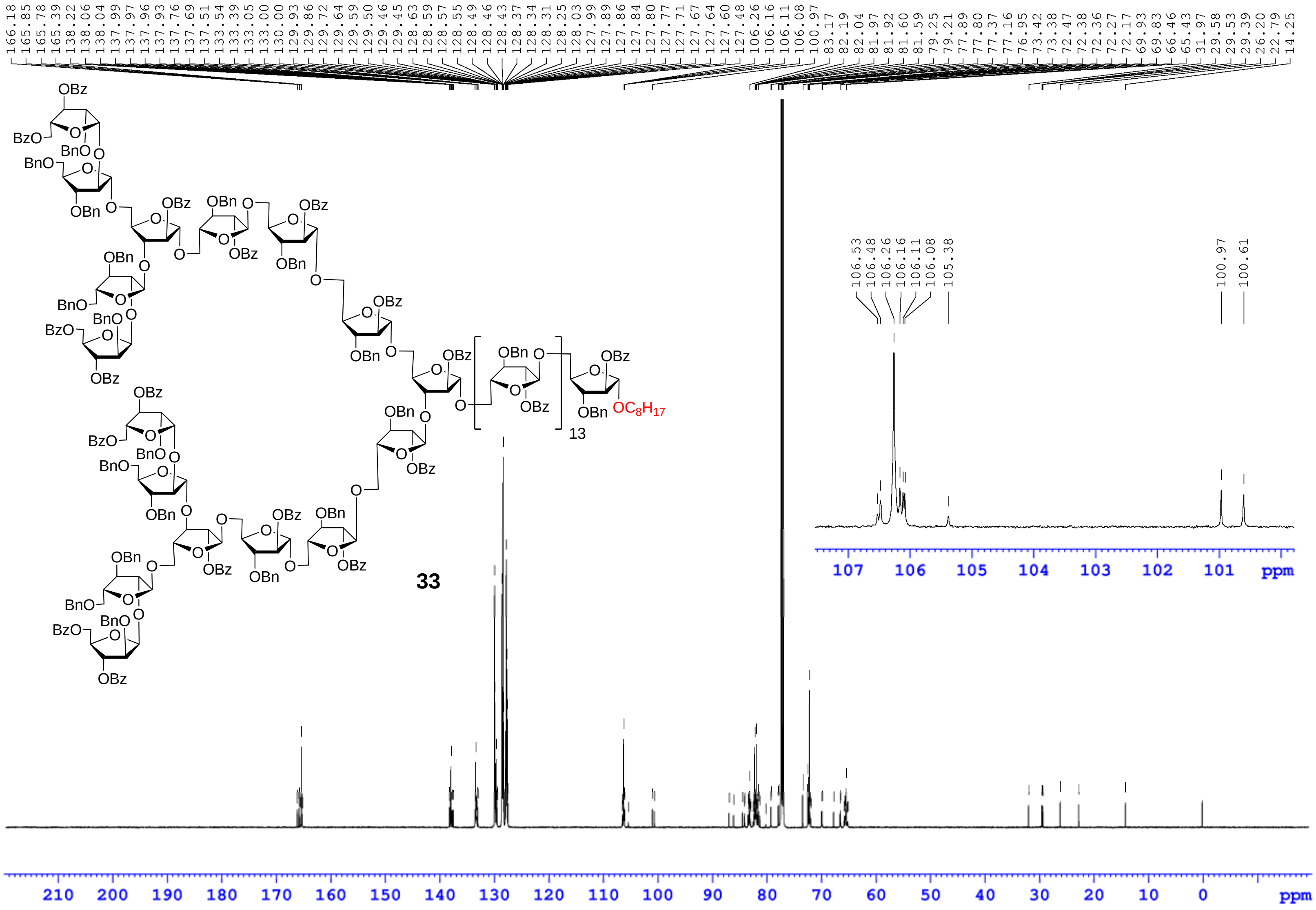

33

Supplementary Figure 113. ^{13}C NMR spectrum of compound 33

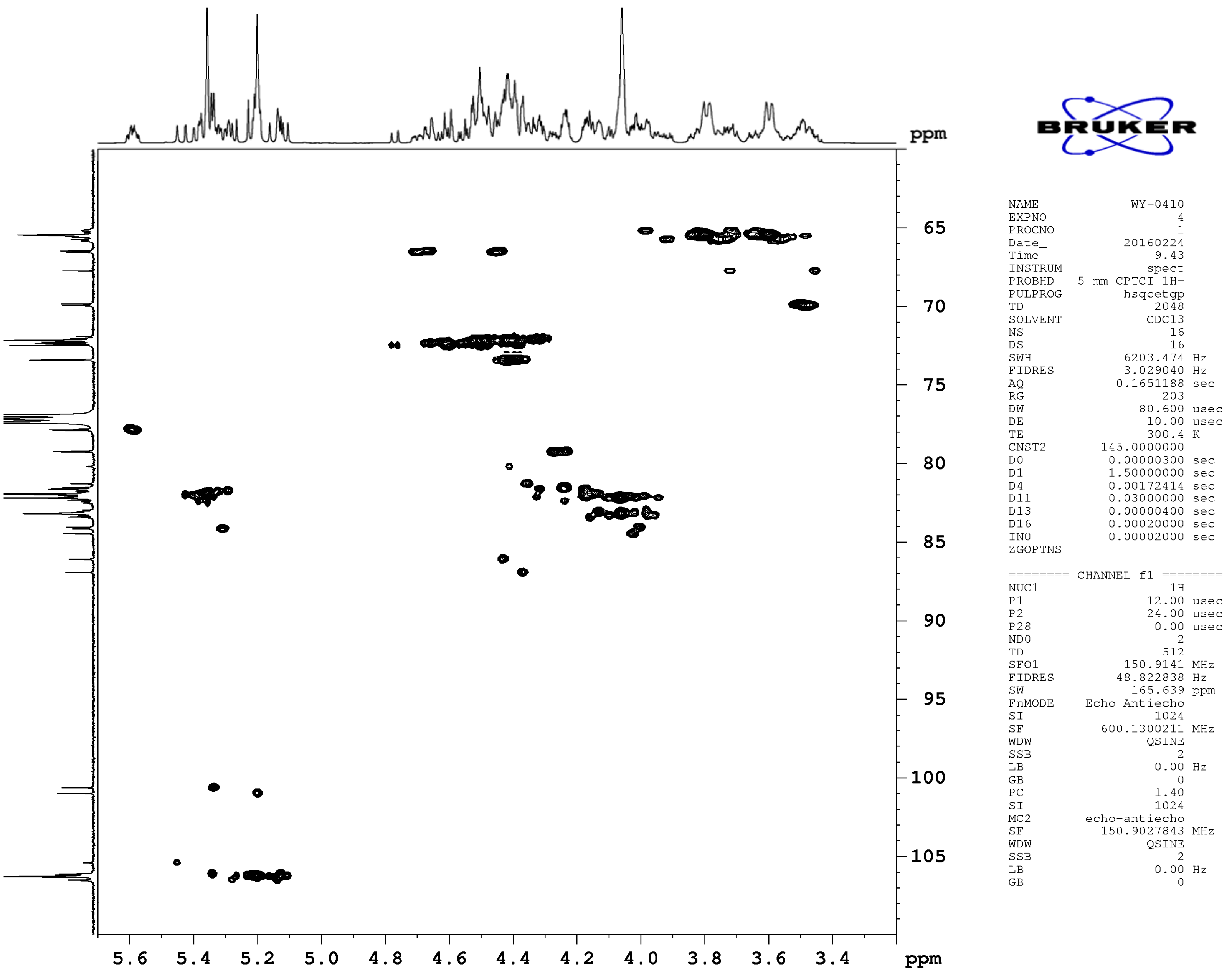

Supplementary Figure 114. HSQC NMR spectrum of compound 33

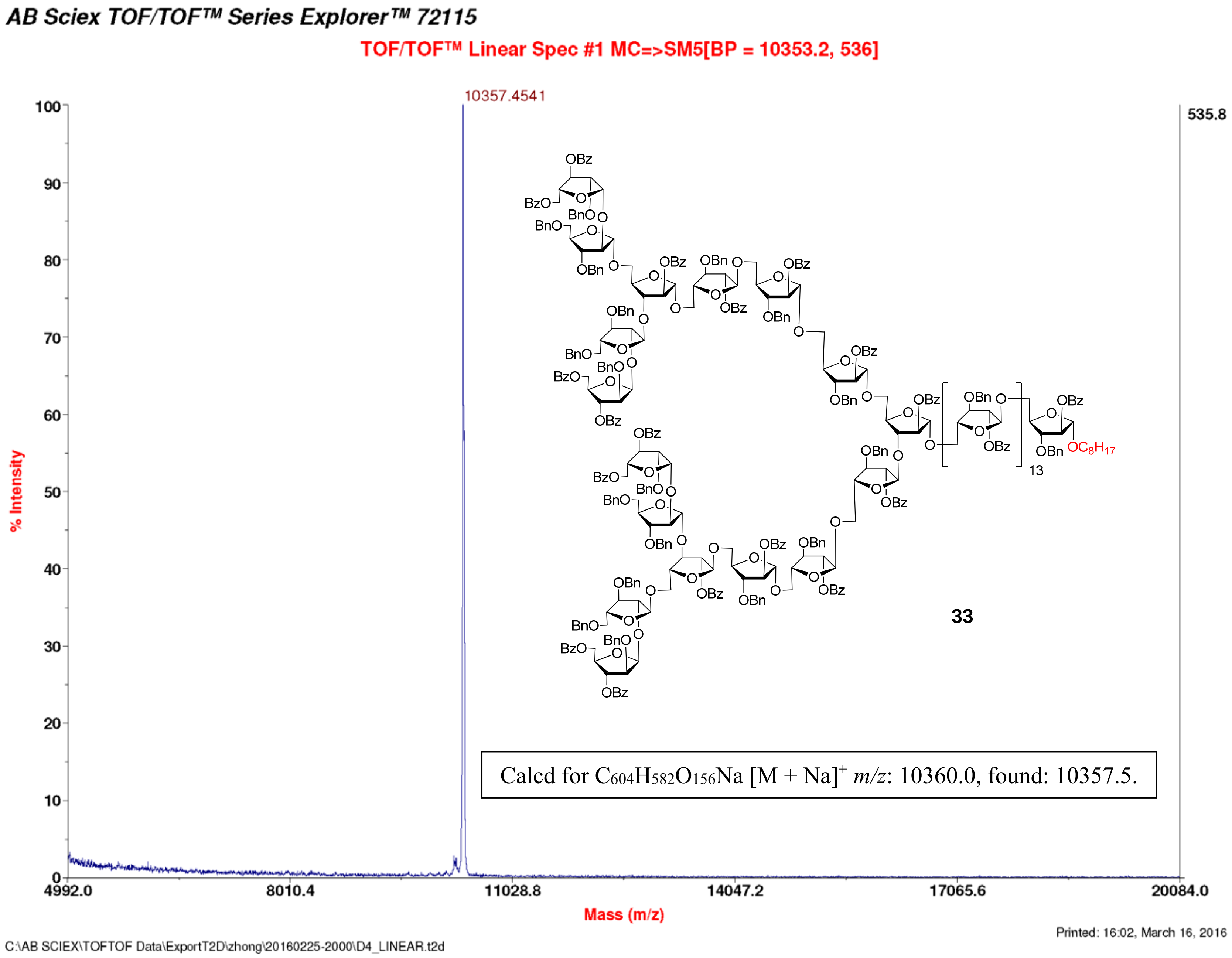

Supplementary Figure 115. MALDI-TOF MS spectrum of compound 33

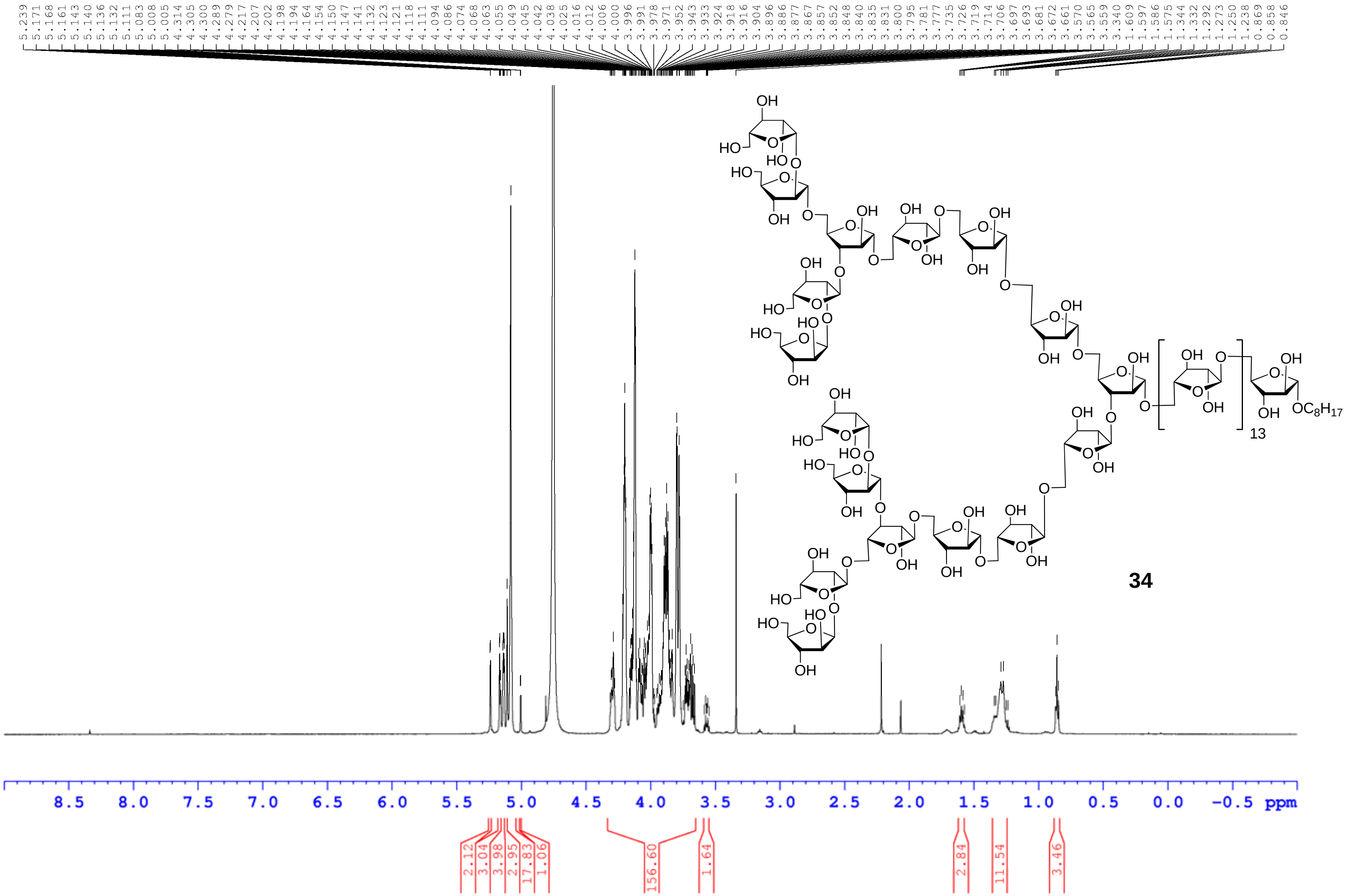

Supplementary Figure 116. ^{1}H NMR spectrum of compound 34

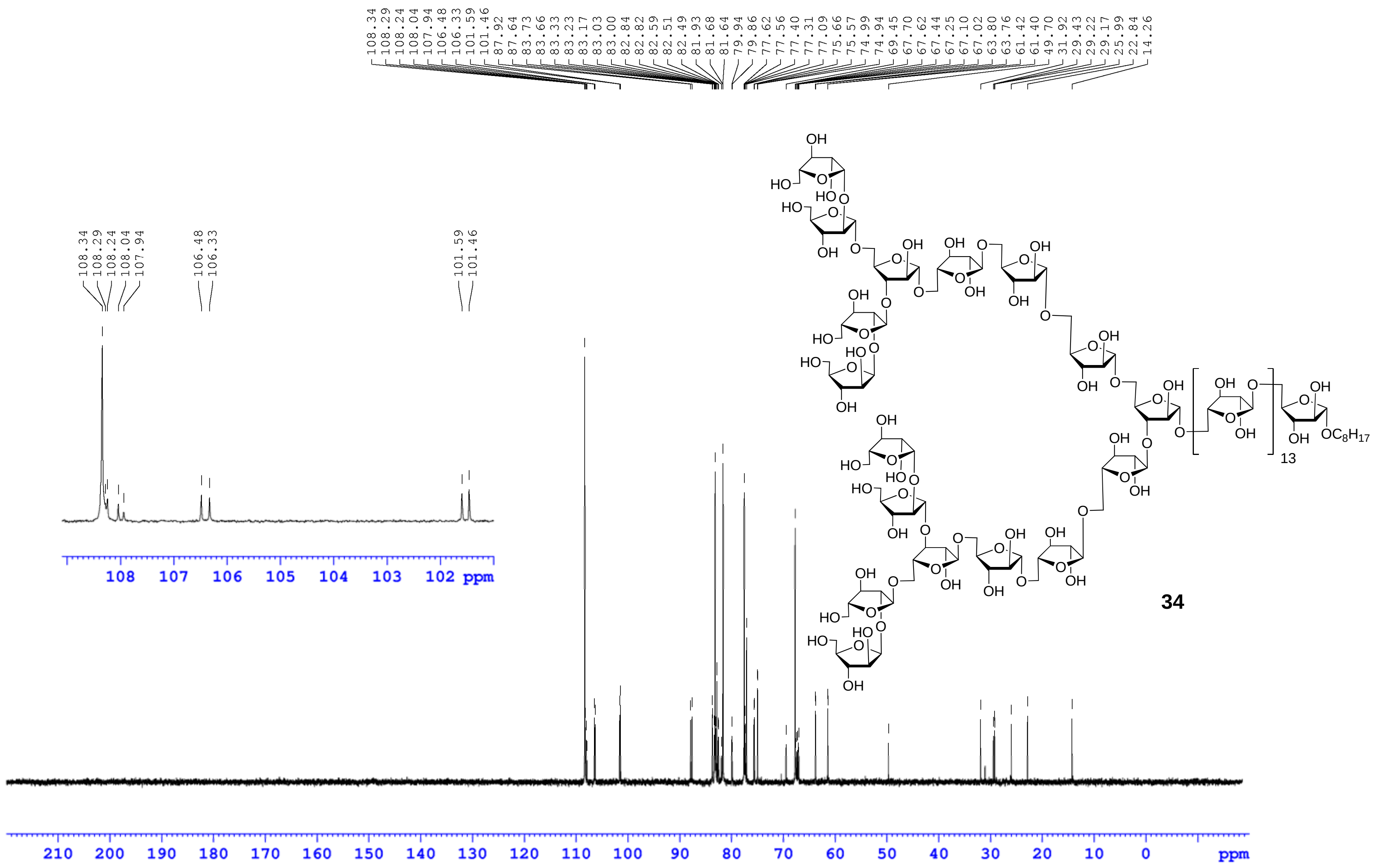

Supplementary Figure 117. ^{13}C NMR spectrum of compound 34

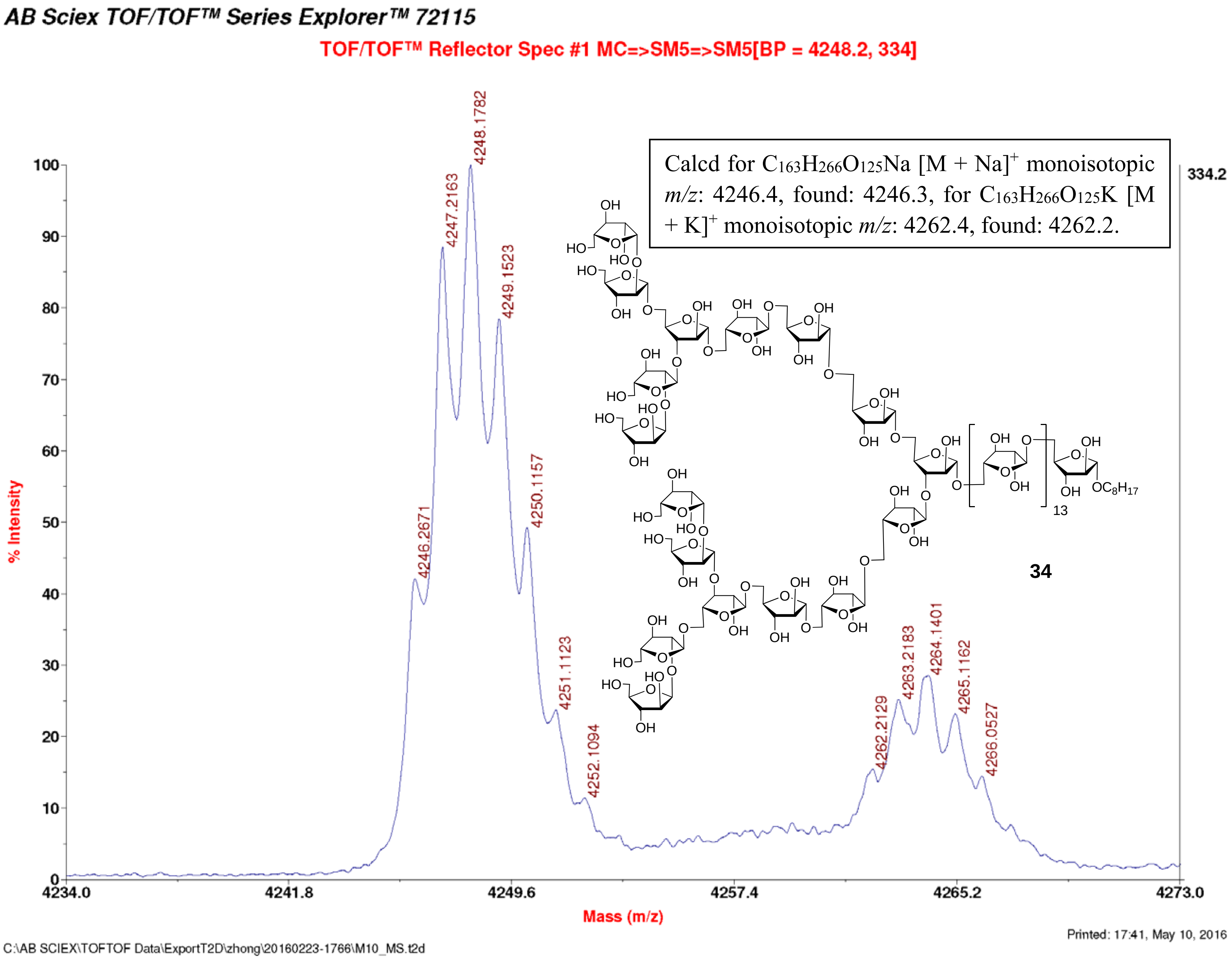

Supplementary Figure 118. MALDI-TOF MS spectrum of compound 34

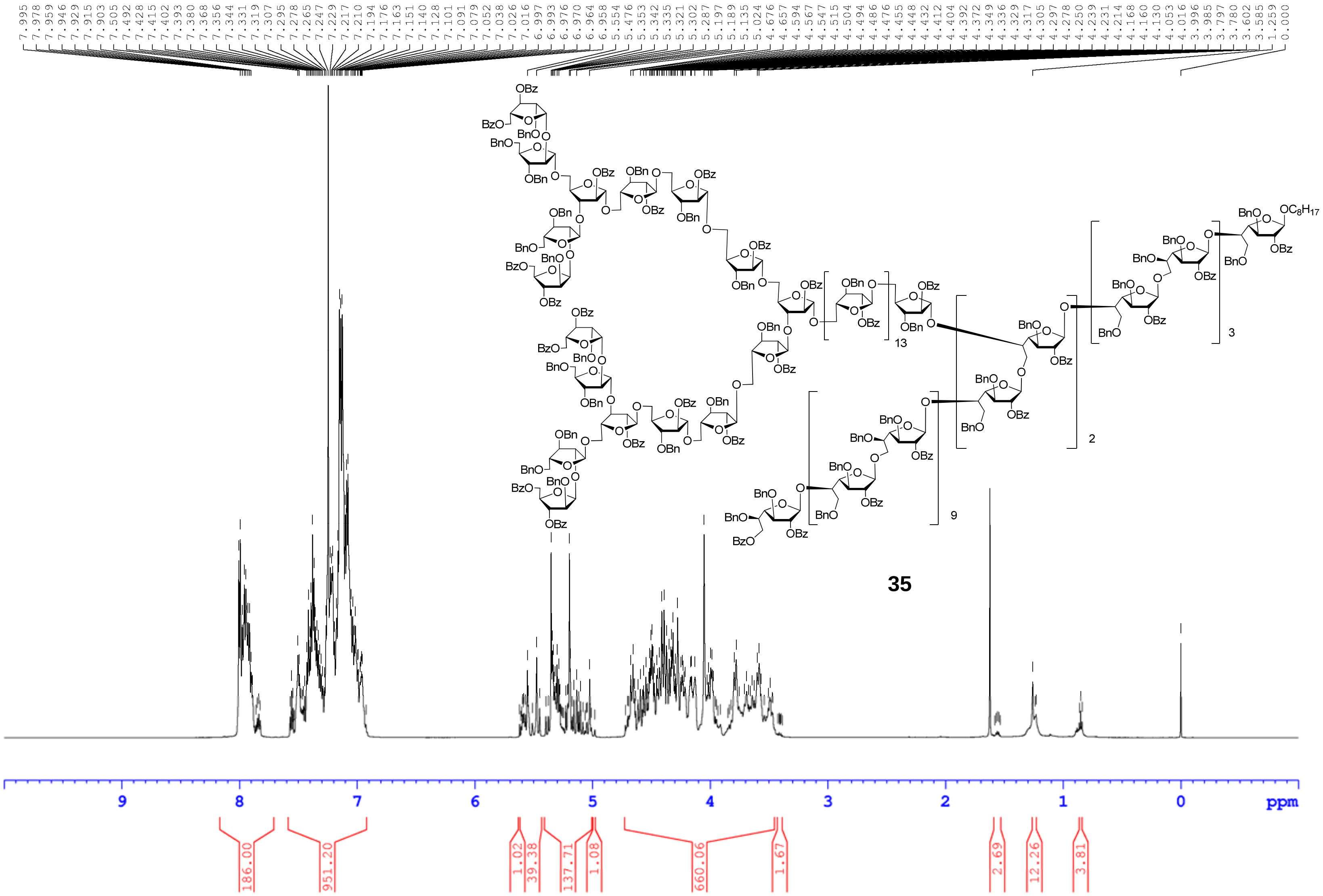

Supplementary Figure 119. ^{1}H NMR spectrum of compound 35

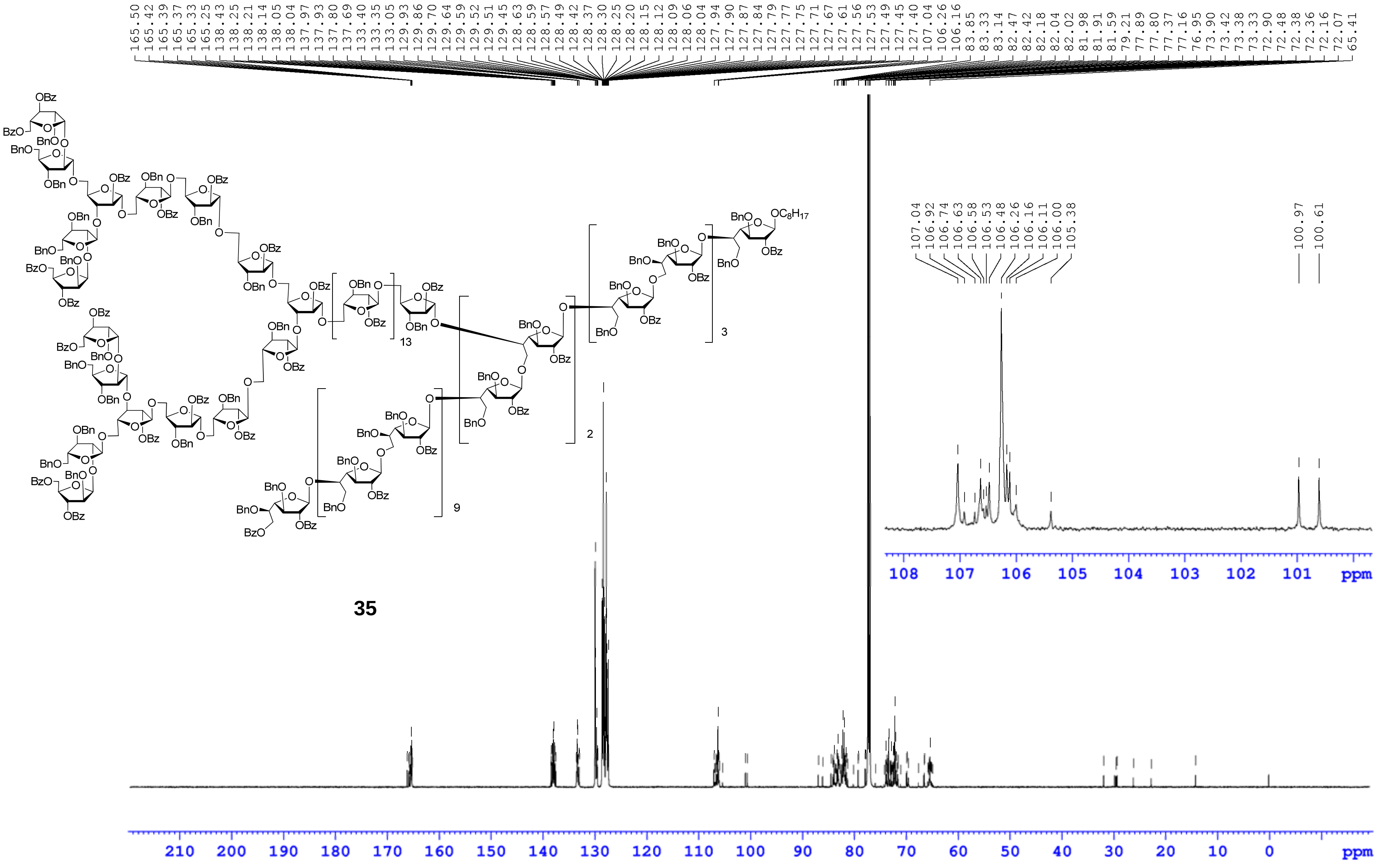

Supplementary Figure 120. ^{13}C **NMR spectrum of compound 35**

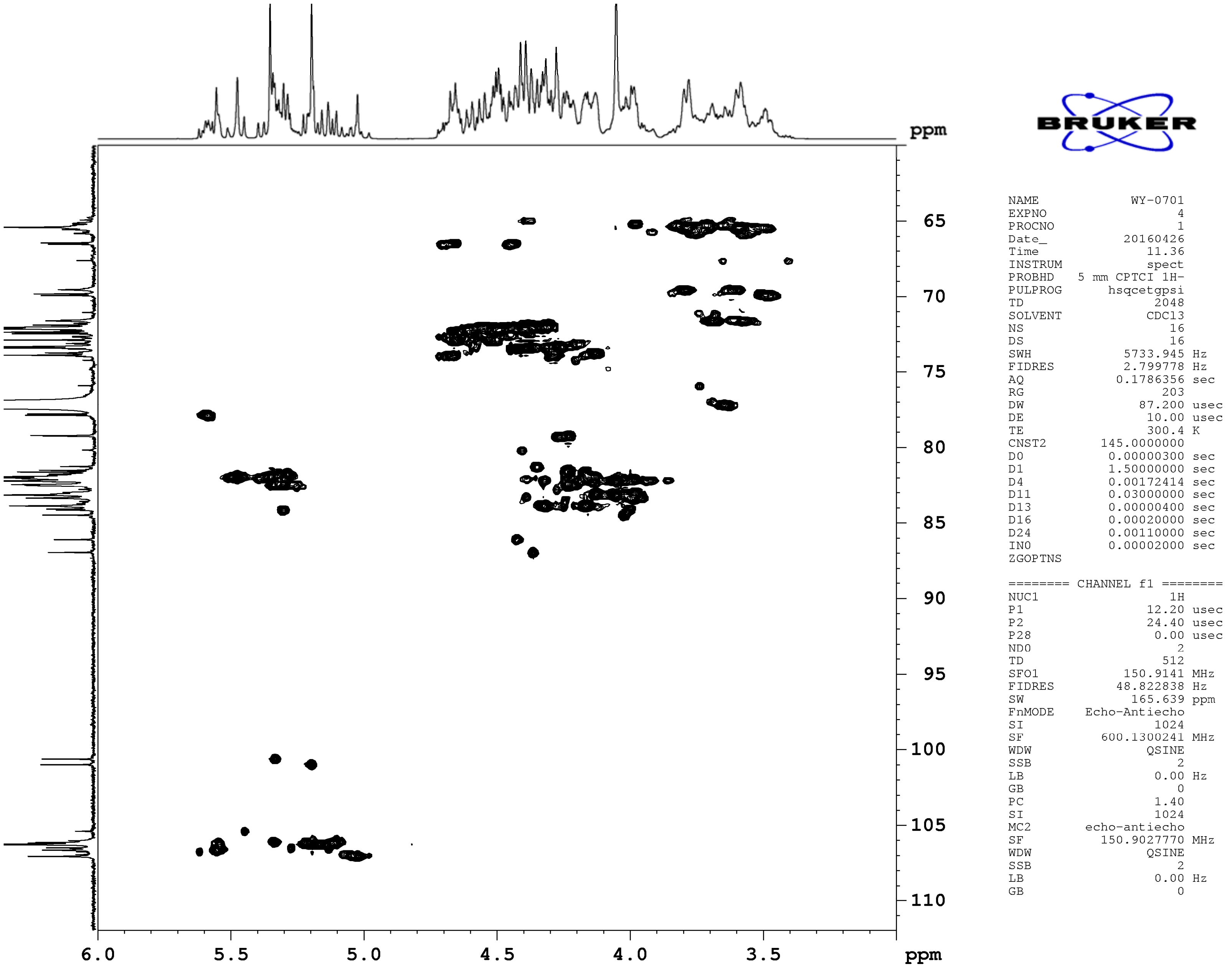

Supplementary Figure 121. HSQC NMR spectrum of compound 35

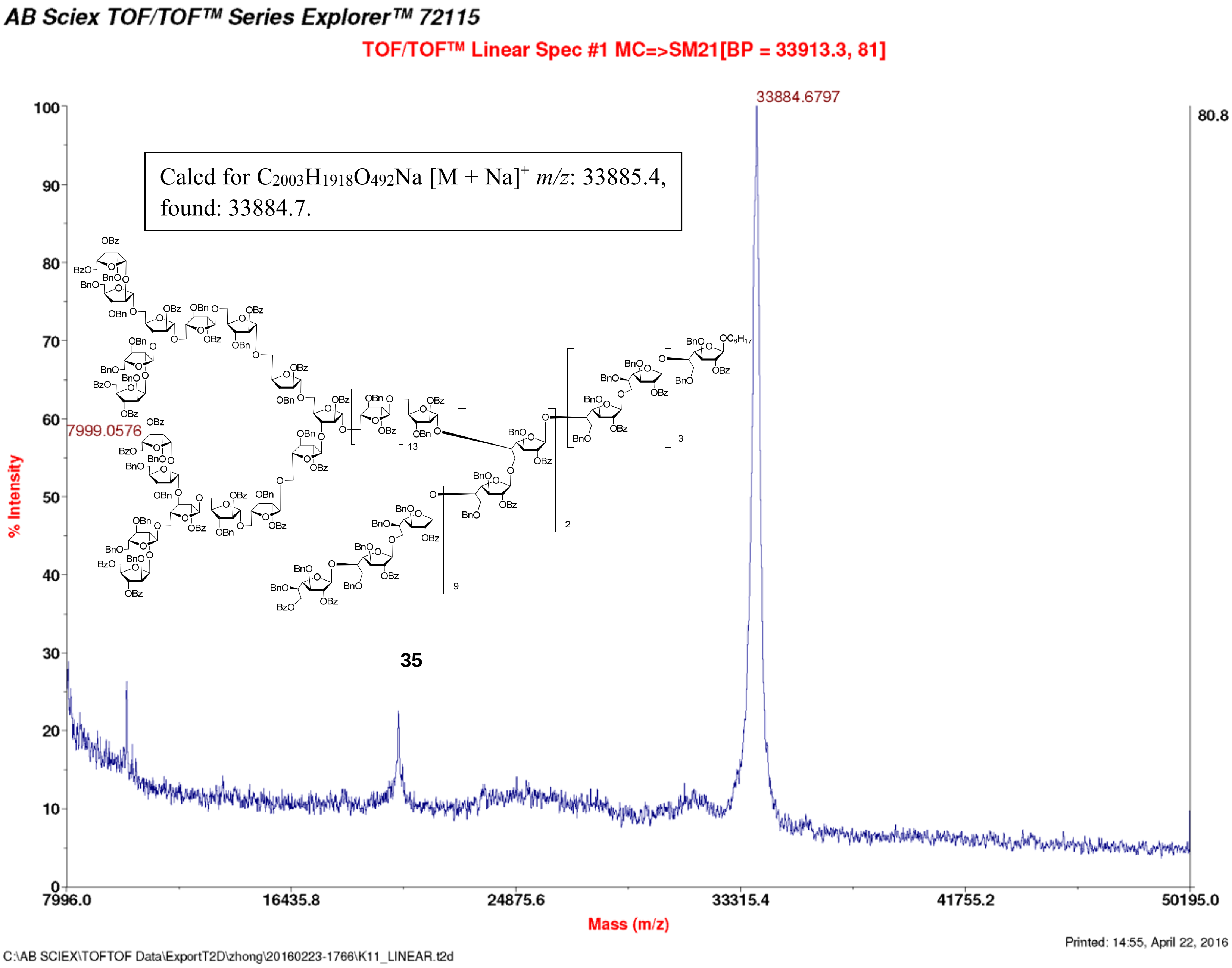

Calcd for $C_{2003}H_{1918}O_{492}Na$ $[M + Na]^+$ *m/z*: 33885.4, found: 33884.7.

Supplementary Figure 122. MALDI-TOF MS spectrum of compound 35

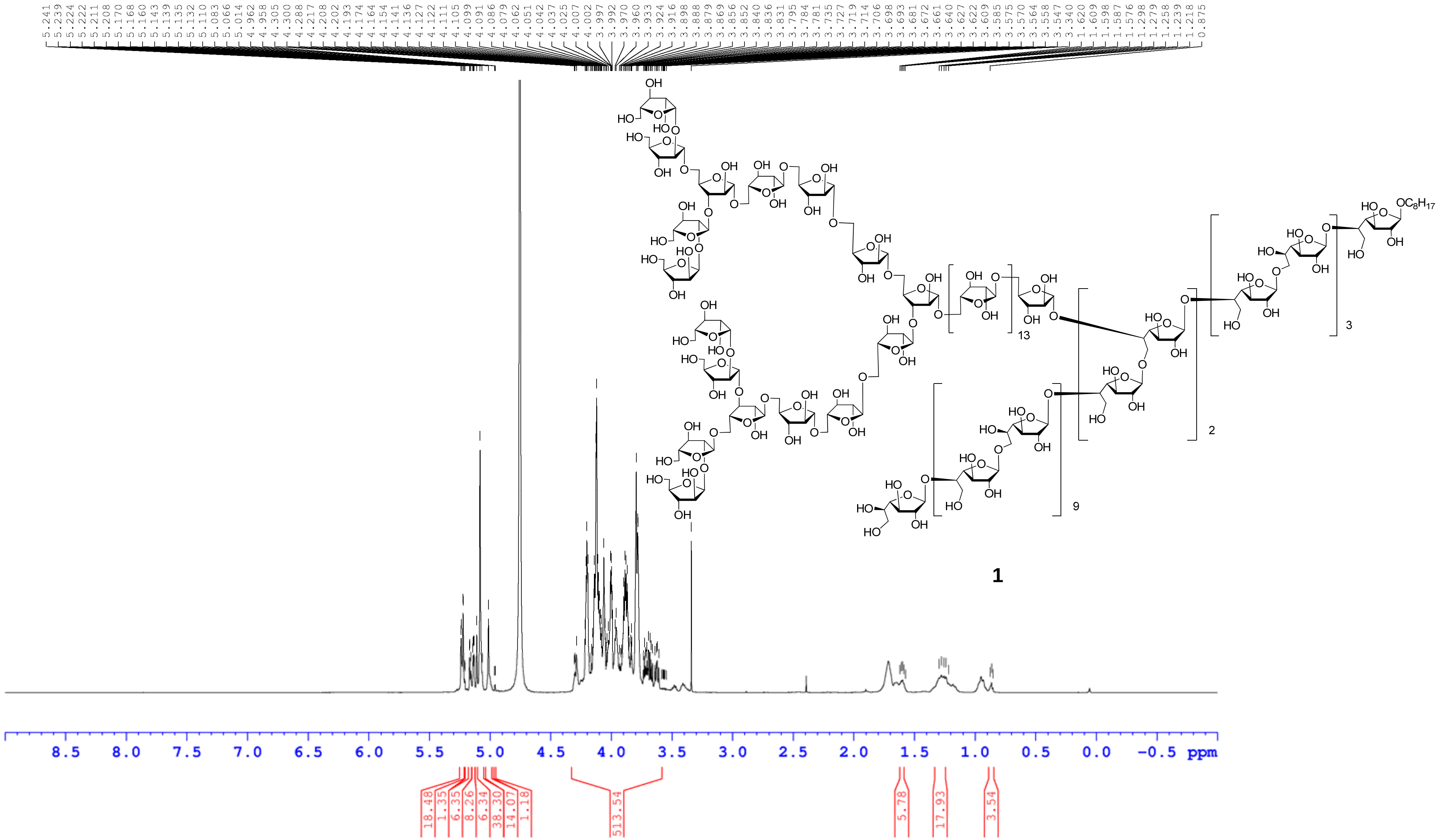

Supplementary Figure 123. **^{1}H NMR spectrum of compound 1**

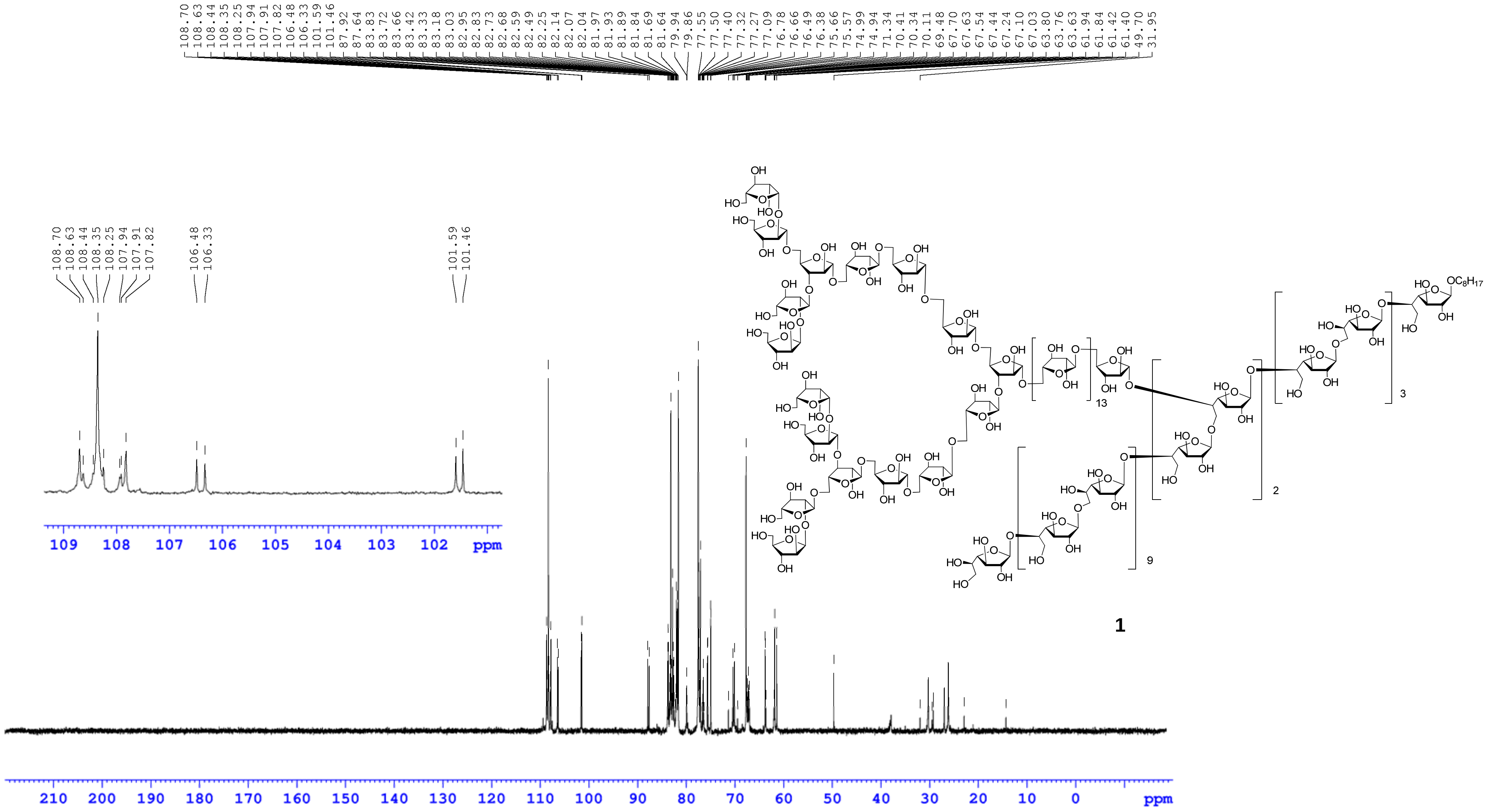

Supplementary Figure 124. ^{13}C NMR spectrum of compound 1

Spectrum Report

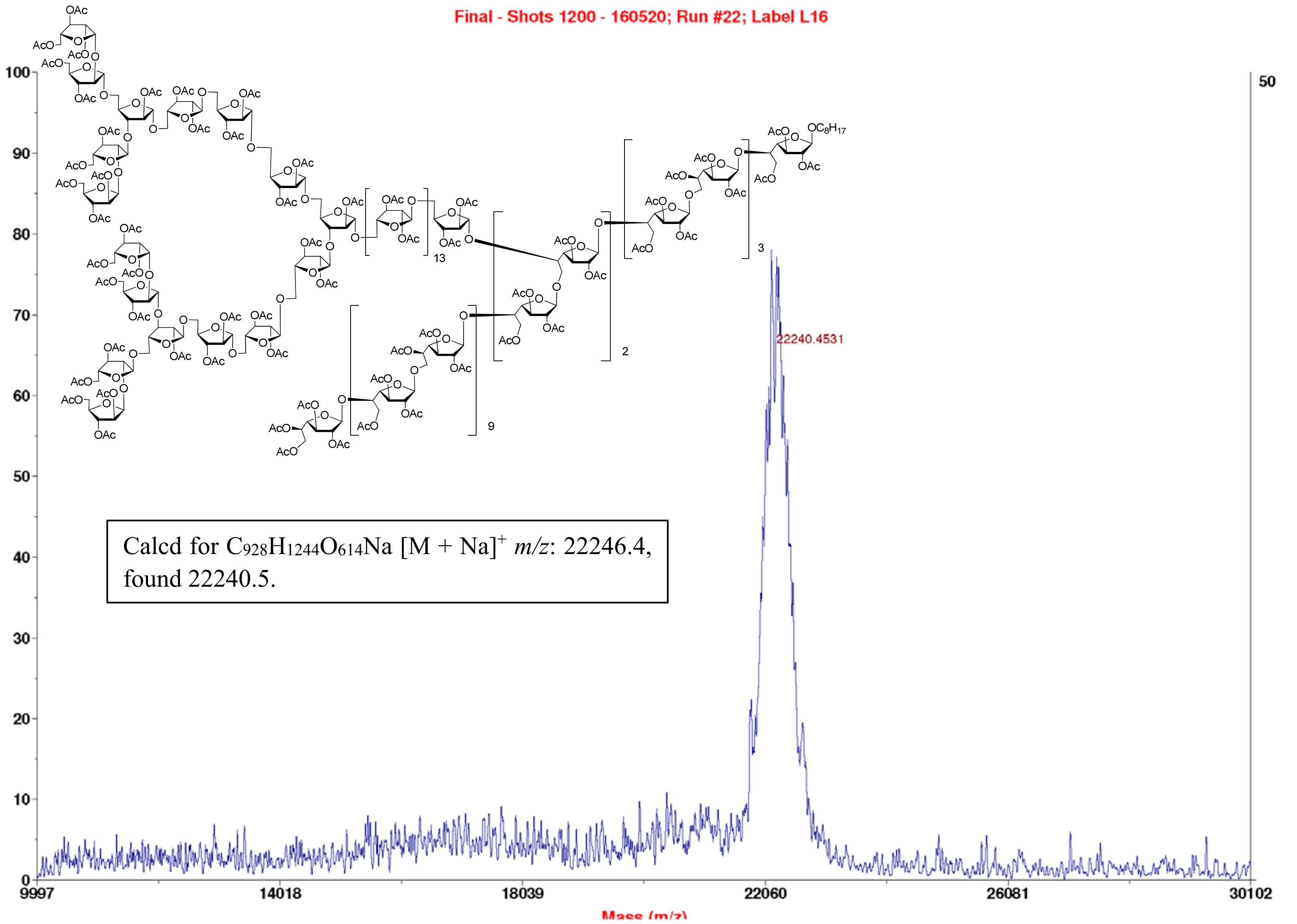

Phoenix 2016\160520 Label L16 Run # 22 Page 1 6/27/2016 11:13:36 AM

Supplementary Figure 125. MALDI-TOF MS spectrum of per-acetylated derivative of compound 1

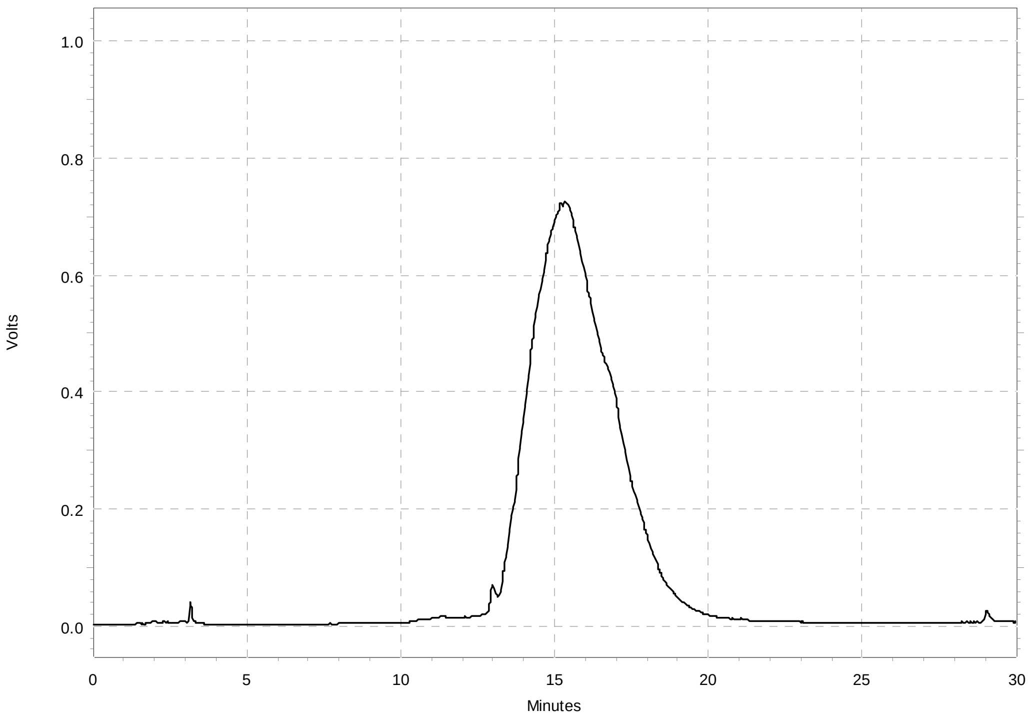

Supplementary Figure 126. HPLC trace of compound 1. Conditions: Waters XBridge® C18 5 µm, 4.6 × 250 mm column, 0-30 min linear gradient: 5-95% CH_3CN, H_2O, 1 mL/min flow.

Supplementary Table 1. One-pot assembly of pentasaccharide 15.

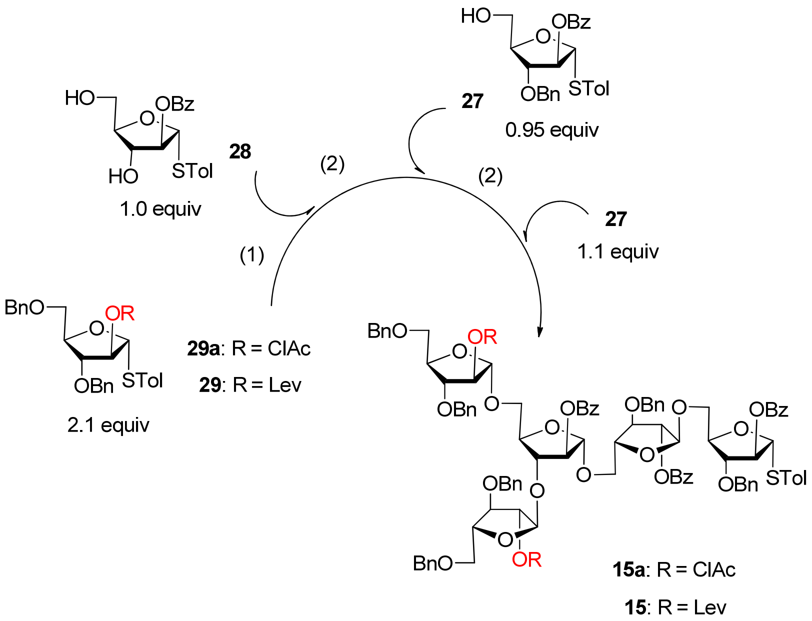

Reagents and conditions: (1) 4 A molecular sieves, CH_2Cl_2, *p*-TolSCl, AgOTf, then **28**, -78 °C to room temperature; (2) *p*-TolSCl, AgOTf, then **27**, -78 °C to room temperature.

Entry	Donor	TTBP (equiv.)	Product	Yield (%)[c]
1	**29a**	4.9[a]	**15a**	0
2	**29a**	4.9[b]	**15a**	43
3	**29a**	0	**15a**	35
4	**29**	4.9[a]	**15**	76

[a] TTBP was added along with donor **29a** or **29**; [b] TTBP was added along with acceptor **28**; [c] Isolated yield. TTBP, 2,4,6-tri-*tert*-butylpyrimidine.

Supplementary Table 2. *β*-Arabinofuranosylation by preactivation protocol.

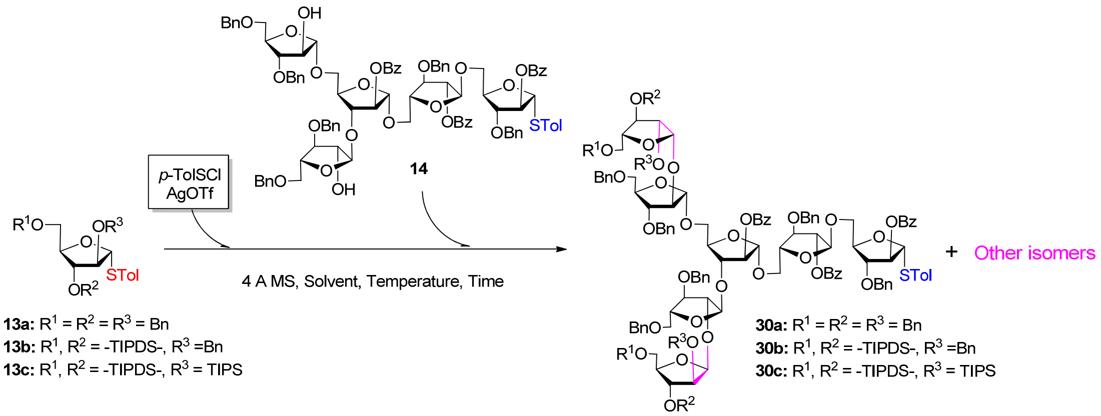

Entry	Donor (**13a-c**, equiv.)	Solvent	Temperature (°C)	Time (min)	Yield (%)[a]	*β,β*-isomer/other isomers[b]
1	**13a**, 4.0	CH_2Cl_2	-78 to r.t.	120	94	_[c]
2	**13b**, 4.0	CH_2Cl_2	-78 to r.t.	120	51	9:1
3	**13c**, 4.0	CH_2Cl_2	-78 to r.t.	120	22	<2:1
4	**13b**, 4.0	CH_2Cl_2	-78	10	88	9:1
5	**13b**, 3.0	CH_2Cl_2	-78	10	81	9:1
6	**13b**, 4.0	Et_2O	-78	10	92	1.5:1

[a] Isolated yield of all the anomeric isomers; [b] Determined by ^{1}H NMR; [c] Beyond calculation due to peak overlapping.

Supplementary Table 3. Assembly of fully protected arabinogalactan 35.

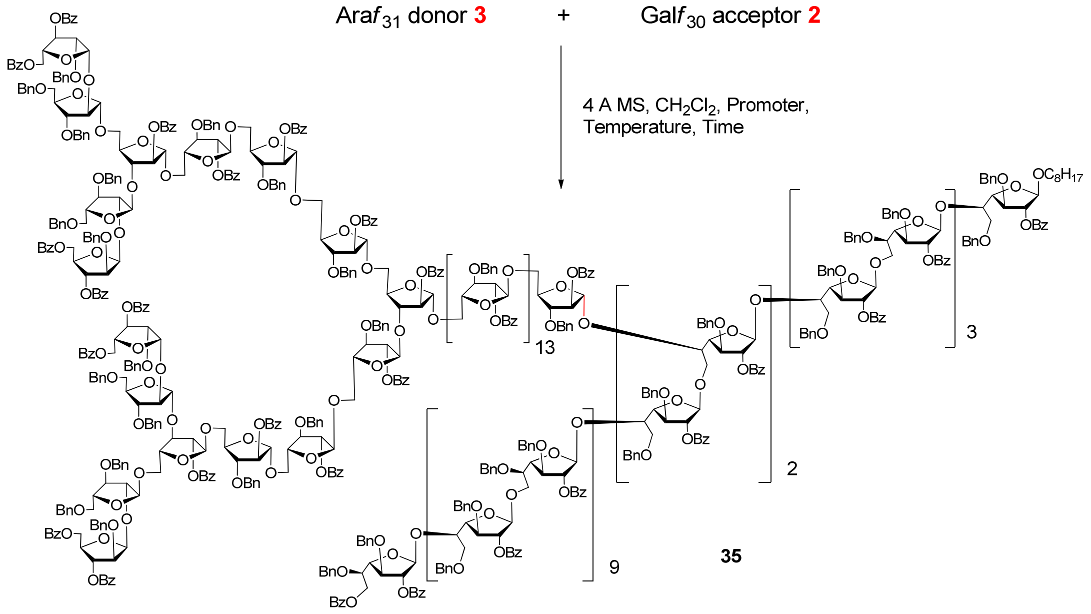

Entry[a]	Promoter system	Temperature (°C)	Time (h)	Yield (%)[e]
1[b]	*p*-TolSCl/AgOTf	-40	10	0
2	*p*-TolSCl/AgOTf	-40	10	0
3	NIS/AgOTf	-40	10	0
4	NIS/AgOTf	-20	10	0
5	NIS/AgOTf	0	10	0
6	NIS/TfOH	-40 to 0	2	0
7[c]	NIS/TfOH	-40 to 0	2	0
8	*N*-(*p*-methylphenylthio)-ε-caprolactam/Tf_2O	-40	4	0
9	*N*-(*p*-methylphenylthio)-ε-caprolactam/Tf_2O	r.t.	0.1	0
10[d]	TBPA	0	4	0
11	$Ph_3Bi(OTf)_2$	r.t.	10	<10
12	BSP/Tf_2O	-40	10	0
13	Ph_2SO/Tf_2O	-40	10	trace[f]
14	BSM/Tf_2O	-40	10	84

[a] Reaction conditions: **3** (5.0 equiv.), **2** (1.0 equiv.); [b] The donor was pre-activated in the absence of acceptor; [c] The reaction was conducted by inverse procedure[4]; [d] The solvent was CH_2Cl_2/CH_3CN (1:1); [e] Isolated yield. [f] Some monoglycosylation product was observed. TBPA, tris(4-bromophenyl)ammoniumyl hexachloroantimonate. BSP, 1-benzenesulfinyl piperidine. BSM, benzenesulfinyl morpholine.

Supplementary Methods

I. General methods

Reactions were carried out in oven-dried glassware. Substrates for glycosylation were dried by azeotoropic removal with toluene. All chemicals were purchased as reagent grade and used without further purification, unless otherwise noted. All solvents were purified before use. CH_2Cl_2, CH_3CN, and pyridine were distilled over CaH_2. Methanol was distilled from magnesium. DMF was stirred with CaH_2 and distilled under reduced pressure. Toluene was distilled over sodium. Ether was distilled with potassium and sodium. All reactions were carried out under anhydrous conditions with freshly distilled solvents under a positive pressure of argon, unless otherwise noted. Reactions were monitored by thin-layer chromatography (TLC) on silica gel-coated aluminum plates (60 F_{254}, E. Merck). Spots were visualized by UV light (254 nm) and charring with a solution of $(NH_4)_6Mo_7O_{24}\cdot 4H_2O$ (24.00 g, 19.4 mmol) and $Ce(NH_4)_2(NO_3)_6$ (0.50 g, 0.9 mmol) in sulfuric acid (5%, 500 mL). Column chromatography was performed on silica gel (200—300 mesh). Gel filtration was performed on Bio-Beads S-X1 or Sephadex LH-20 (Pharmacia). Optical rotations were obtained on a Hanon P850 Automatic Polarimeter. ^1H NMR spectra were recorded at room temperature for solutions in $CDCl_3$ or D_2O with the Avance III-400 or III-600 instruments (Bruker), and the chemical shifts were referenced to the peak for TMS (0 ppm, $CDCl_3$) or external CH_3OH (3.34 ppm, D_2O). ^{13}C NMR spectra were recorded using the same NMR spectrometers and the chemical shifts were reported relative to internal $CDCl_3$ (δ = 77.16 ppm) or external CH_3OH (49.70 ppm, D_2O). Assignments of resonances in ^1H and ^{13}C NMR spectra were done using ^1H-^1H COSY, HSQC and HMBC experiments (600 MHz for all the oligosaccharides synthesized in this study). The following standard abbreviations are used to indicate multiplicity: s = singlet, d = doublet, t = triplet, m = multiplet, dd = doublet of doublets, dt = doublet of triplets, td = triplet of doublets and br = broad. HRMS experiments were performed on a Waters Xevo G2 Q-TOF spectrometer or a Bruker APEX IV FTMS instrument. MALDI-TOF mass spectra were performed on an AB

SCIEX 5800 spectrometer with 2,5-dihydroxybenzoic acid as the matrix. Analytical HPLC was performed on a Shimadzu LC-10AT liquid chromatograph equipped with ELSD detector (ELSD 2000ES).

II. Synthetic methods and characterization data

Synthesis of Gal*f*$_{30}$ acceptor 2:

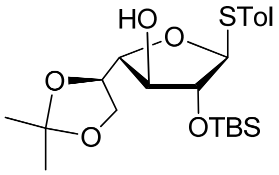

***p*-Tolyl 2-*O*-*tert*-butydimethylsilyl-5,6-*O*-isopropylidene-1-thio-*β*-D-galactofuranoside (18b):** To a solution of **18a**[1] (18.0 g, 55.1 mmol) and imidazole (11.3 g, 165.3 mmol) in anhydrous CH_2Cl_2 (150 mL) was added TBSCl (10.0 g, 66.1 mmol) at 0 °C. After stirring for 2 h at the same temperature, the solution was quenched with CH_3OH (5 mL) and stirred for another 30 min at room temperature. The reaction mixture was then concentrated under reduced pressure, and the residue was purified by column chromatography on silica gel (petroleum ether/ethyl acetate, 5:1) to afford **18b** (16.0 g, 66%) as colorless oil. R_f = 0.50 (petroleum ether/ethyl acetate, 3:1); $[\alpha]_D^{30}$ -123.5 (*c* 1.6, CH_2Cl_2); ^1H NMR (400 MHz, $CDCl_3$) δ 7.39 (d, *J* = 8.1 Hz, 2H, Ar), 7.12 (d, *J* = 7.9 Hz, 2H, Ar), 5.25 (d, *J* = 3.3 Hz, 1H, H-1), 4.33 (dd, *J* = 12.2, 6.5 Hz, 1H, H-5), 4.13 (t, *J* = 3.4 Hz, 1H, H-2), 4.10 (t, *J* = 5.7 Hz, 1H, H-4), 4.02 (dd, *J* = 8.5, 6.9 Hz, 1H, H-6a), 3.97 – 3.93 (m, 2H, H-3, H-6b), 2.33 (s, 3H, tolyl CH_3), 2.22 (d, *J* = 7.1 Hz, 1H, -OH), 1.44 (s, 3H, *i*-Pr), 1.36 (s, 3H, *i*-Pr), 0.89 (s, 9H, *t*-Bu), 0.10 (s, 3H, CH_3), 0.09 (s, 3H, CH_3); ^{13}C NMR (100 MHz, $CDCl_3$) δ 137.95 (Ar), 132.79 (Ar), 130.02 (Ar), 129.90 (Ar), 109.81 (*i*-Pr), 92.76 (C-1), 83.88 (C-4), 82.96 (C-2), 78.95 (C-3), 75.28 (C-5), 65.45 (C-6), 26.51 (*i*-Pr), 25.81 (*t*-Bu), 25.25 (*i*-Pr), 21.23 (tolyl CH_3), 18.06 (*t*-Bu), -4.52 (CH_3), -4.59 (CH_3); HRMS (ESI) Calcd for $C_{22}H_{40}NO_5SSi$ $[M + NH_4]^+$: 458.2391, found: 458.2386.

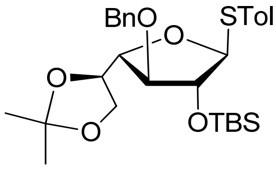

***p*-Tolyl 2-*O*-*tert*-butydimethylsilyl-3-*O*-benzyl-5,6-*O*-isopropylidene-1-thio-*β*-D-galactofuranoside (18c):** To a solution of **18b** (15.0 g, 34.0 mmol) and BnBr (4.8 mL, 40.8 mmol) in anhydrous DMF (50 mL) was slowly added NaH (1.5 g, 37.4 mmol, 60% in mineral oil) at 0 °C. The reaction mixture was slowly warmed to room temperature and stirred for 1 h, quenched with ice water, and extracted with CH_2Cl_2 (2 × 150 mL). The combined organic layer, after being washed with a saturated aqueous NH_4Cl solution (150 mL) and brine (150 mL), was dried over Na_2SO_4, filtered, and concentrated. The residue was purified by column chromatography on silica gel (petroleum ether/ethyl acetate, 25:1) to afford **18c** (14.6 g, 81%) as colorless syrup. R_f = 0.44 (petroleum ether/ethyl acetate, 8:1); $[\alpha]_D^{30}$ -159.6 (*c* 4.5, CH_2Cl_2); ^{1}H NMR (400 MHz, $CDCl_3$) δ 7.43 (d, *J* = 8.0 Hz, 2H, Ar), 7.39 – 7.30 (m, 5H, Ar), 7.12 (d, *J* = 7.9 Hz, 2H, Ar), 5.30 (d, *J* = 2.3 Hz, 1H, H-1), 4.72 (d, *J* = 11.8 Hz, 1H, $PhCH_2$), 4.58 (d, *J* = 11.8 Hz, 1H, $PhCH_2$), 4.37 (t, *J* = 2.4 Hz, 1H, H-2), 4.30 – 4.25 (m, 2H, H-4, H-5), 3.95 – 3.88 (m, 2H, H-6a, H-6b), 3.84 (dd, *J* = 4.3, 2.6 Hz, 1H, H-3), 2.34 (s, 3H, tolyl CH_3), 1.42 (s, 3H, *i*-Pr), 1.37 (s, 3H, *i*-Pr), 0.92 (s, 9H, *t*-Bu), 0.15 (s, 6H, CH_3); ^{13}C NMR (100 MHz, $CDCl_3$) δ 137.73 (Ar), 137.45 (Ar), 132.40 (Ar), 131.27 (Ar), 129.76 (Ar), 128.53 (Ar), 127.95 (Ar), 127.84 (Ar), 109.63 (*i*-Pr), 94.13 (C-1), 86.12 (C-3), 82.33 (C-4), 82.00 (C-2), 75.55 (C-5), 72.43 ($PhCH_2$), 65.59 (C-6), 26.47 (*i*-Pr), 25.80 (*t*-Bu), 25.38 (*i*-Pr), 21.17 (tolyl CH_3), 17.96 (*t*-Bu), -4.22 (CH_3), -4.77 (CH_3); HRMS (ESI) Calcd for $C_{29}H_{46}NO_5SSi$ [M + NH_4]$^+$: 548.2861, found: 548.2862.

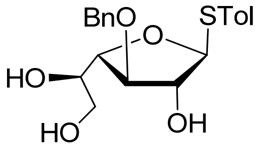

***p*-Tolyl 3-*O*-benzyl-1-thio-*β*-D-galactofuranoside (18d):** To a solution of **18c** (18.0 g, 33.9 mmol) in THF (50 mL) was added TBAF (37.3 mL, 37.3 mmol, 1 M in THF) at

room temperature. After stirring for 30 min, the reaction mixture was concentrated to give a crude residue, which was dissolved in 70% AcOH aqueous solution (100 mL). The resulting mixture was stirred overnight at 50 °C, and concentrated *in vacuo*. The residue was then purified by column chromatography on silica gel (petroleum ether/acetone, 2:1) to afford **18d** (12.1 g, 94% for two steps) as colorless syrup. R_f = 0.37 (petroleum ether/acetone, 1:1); $[\alpha]_D^{30}$ -233.1 (*c* 0.8, CH_2Cl_2); ^{1}H NMR (400 MHz, $CDCl_3$) δ 7.38 – 7.29 (m, 7H, Ar), 7.12 (d, *J* = 7.9 Hz, 2H, Ar), 5.39 (d, *J* = 2.0 Hz, 1H, H-1), 4.76 (d, *J* = 11.9 Hz, 1H, $PhCH_2$), 4.61 (d, *J* = 11.9 Hz, 1H, $PhCH_2$), 4.35 – 4.31 (m, 2H, H-2, H-4), 4.04 (dd, *J* = 4.2, 2.2 Hz, 1H, H-3), 3.84 – 3.79 (m, 2H, H-5, H-6a), 3.76 – 3.70 (m, 1H, H-6b), 3.31 (d, *J* = 8.4 Hz, 1H, -OH), 2.79 (d, *J* = 5.4 Hz, 1H, -OH), 2.33 (s, 3H, tolyl CH_3), 2.08 (dd, *J* = 8.2, 3.6 Hz, 1H, -OH); ^{13}C NMR (100 MHz, $CDCl_3$) δ 137.83 (Ar), 137.58 (Ar), 132.39 (Ar), 131.09 (Ar), 129.95 (Ar), 128.73 (Ar), 128.19 (Ar), 128.04 (Ar), 95.07 (C-1), 85.53 (C-3), 84.57 (C-4), 79.51 (C-2), 72.48 ($PhCH_2$), 70.95 (C-5), 64.61 (C-6), 21.24 (tolyl CH_3); HRMS (ESI) Calcd for $C_{20}H_{28}NO_5S$ $[M + NH_4]^+$: 394.1683, found: 394.1681.

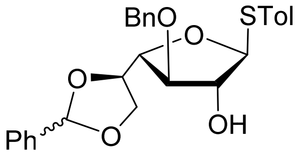

***p*-Tolyl 3-*O*-benzyl-5,6-*O*-(*R*/*S*)-benzylidene-1-thio-*β*-D-galactofuranoside (18e):** To a solution of **18d** (13.0 g, 34.5 mmol) in anhydrous DMF (60 mL) was added benzaldehyde dimethyl acetal (5.7 mL, 38.0 mmol) and a catalytic amount of PPTS (170 mg, 0.69 mmol). The reaction was conducted under reduced pressure at 60 °C for 1 h, quenched with Et_3N (1 mL) and concentrated. The residue was purified by column chromatography on silica gel (petroleum ether/acetone, 8:1) to afford **18e** (14.9 g, 93%) as colorless syrup. R_f = 0.40 (petroleum ether/acetone, 3:1); ^{1}H NMR (400 MHz, $CDCl_3$) δ 7.46 – 7.28 (m, 24H), 7.13 (m, 4H), 5.85 (s, 1H), 5.78 (s, 1H), 5.46 (d, *J* = 2.0 Hz, 1H), 5.39 (d, *J* = 2.0 Hz, 1H), 4.76 (d, *J* = 11.9 Hz, 1H), 4.72 (d, *J* = 11.9 Hz, 1H), 4.58 (d, *J* = 11.7 Hz, 1H), 4.55 (d, *J* = 11.8 Hz, 1H), 4.39 – 4.35 (m, 4H), 4.32 (dd, *J* = 3.7, 1.5 Hz, 1H), 4.28 – 4.21 (m, 3H), 4.12 – 4.04 (m, 3H), 4.01 (dd,

J = 3.2, 1.9 Hz, 1H), 3.46 (d, *J* = 8.5 Hz, 1H), 3.02 (d, *J* = 7.7 Hz, 1H), 2.33 (s, 3H), 2.33 (s, 3H); ^{13}C NMR (100 MHz, $CDCl_3$) δ 137.62, 137.56, 137.46, 136.98, 136.73, 132.21, 132.08, 131.51, 131.49, 129.90, 129.87, 129.84, 129.67, 128.68, 128.65, 128.60, 128.53, 128.13, 128.08, 127.99, 127.95, 126.85, 126.66, 104.94, 104.73, 95.02, 94.79, 85.75, 85.48, 82.74, 82.60, 79.50, 79.26, 76.56, 76.04, 72.38, 72.33, 67.23, 66.75, 21.22; HRMS (ESI) Calcd for $C_{27}H_{32}NO_5S$ $[M + NH_4]^+$: 482.1996, found: 482.1992.

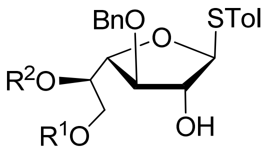

18f: R^1 = Bn; R^2 = H
18g: R^1 = H; R^2 = Bn

***p*-Tolyl 3,6-di-*O*-benzyl-1-thio-*β*-D-galactofuranoside (18f) and *p*-Tolyl 3,5-di-*O*-benzyl-1-thio-*β*-D-galactofuranoside (18g):** To a solution of **18e** (15.0 g, 32.3 mmol) in anhydrous CH_2Cl_2 (65 mL) charged with argon gas was added DIBAL-H (65 mL, 96.6 mmol, 1.5 M in toluene) at -40 °C. After stirring for 24 h at the same temperature, the reaction mixture was allowed to warm to room temperature, quenched with a saturated aqueous NH_4Cl solution, and filtered. Then the aqueous layer was extracted by CH_2Cl_2 (2 × 150 mL), the combined organic layer was dried over Na_2SO_4, filtered, and concentrated to give a crude residue, which was purified by column chromatography on silica gel (CH_2Cl_2/CH_3OH, 100:1) to afford **18f** (4.2 g, 28%) and **18g** (9.0 g, 60%) as colorless syrup.

For compound **18f**: R_f = 0.30 (CH_2Cl_2/CH_3OH, 30:1); $[\alpha]_D^{30}$ -188.9 (*c* 5.2, CH_2Cl_2); ^{1}H NMR (400 MHz, $CDCl_3$) δ 7.39 – 7.28 (m, 12H, Ar), 7.09 (d, *J* = 9.0 Hz, 2H, Ar), 5.44 (s, 1H, H-1), 4.74 (d, *J* = 12.0 Hz, 1H, $PhCH_2$), 4.59 – 4.51 (m, 3H, $PhCH_2$), 4.35 (dt, *J* = 9.8, 1.5 Hz, 1H, H-2), 4.30 (t, *J* = 1.4 Hz, 1H, H-3), 4.04 – 4.04 (m, 1H, H-4), 4.00 – 3.95 (m, 1H, H-5), 3.79 (d, *J* = 9.8 Hz, 1H, -OH), 3.65 – 3.57 (m, 2H, H-6a, H-6b), 2.80 (dd, *J* = 3.3, 1.2 Hz, 1H, -OH), 2.32 (s, 3H, tolyl CH_3); ^{13}C NMR (100 MHz, $CDCl_3$) δ 137.78 (Ar), 137.69 (Ar), 137.32 (Ar), 131.94 (Ar), 131.91 (Ar), 129.82 (Ar), 128.67 (Ar), 128.09 (Ar), 128.06 (Ar), 127.97 (Ar), 127.96 (Ar), 95.50 (C-1), 85.84 (C-4), 83.53 (C-3), 79.14 (C-2), 73.67 ($PhCH_2$), 72.28 ($PhCH_2$), 71.54

(C-6), 70.55 (C-5), 21.22 (tolyl CH_3); HRMS (ESI) Calcd for $C_{27}H_{34}NO_5S$ [M + $NH_4]^+$: 484.2152, found: 484.2148.

For compound **18g**: R_f = 0.35 (CH_2Cl_2/CH_3OH, 30:1); $[\alpha]_D^{30}$ -196.7 (*c* 1.9, CH_2Cl_2); ^{1}H NMR (400 MHz, $CDCl_3$) δ 7.38 – 7.26 (m, 12H, Ar), 7.09 (d, *J* = 7.9 Hz, 2H, Ar), 5.43 (s, 1H, H-1), 4.71 – 4.67 (m, 2H, PhCH_2), 4.51 – 4.47 (m, 3H, H-4, PhCH_2), 4.30 (d, *J* = 9.6 Hz, 1H, H-2), 3.93 (d, *J* = 3.2 Hz, 1H), 3.90 – 3.81 (m, 2H, H-6a, H-6b), 3.65 (d, *J* = 9.8 Hz, 1H, -OH), 3.60 (dd, *J* = 7.3, 4.1 Hz, 1H, H-5), 2.3 – 2.31 (m, 4H, -OH, tolyl CH_3); ^{13}C NMR (100 MHz, $CDCl_3$) δ 137.56 (Ar), 137.53 (Ar), 137.07 (Ar), 132.13 (Ar), 131.55 (Ar), 129.88 (Ar), 128.80 (Ar), 128.67 (Ar), 128.55 (Ar), 128.48 (Ar), 128.11 (Ar), 128.08 (Ar), 95.32 (C-1), 85.32 (C-3), 85.10 (C-4), 79.33 (C-2), 77.06 (C-5), 72.51 (PhCH_2), 72.25 (PhCH_2), 62.00 (C-6), 21.23 (tolyl CH_3); HRMS (ESI) Calcd for $C_{27}H_{34}NO_5S$ [M + $NH_4]^+$: 484.2152, found: 484.2144.

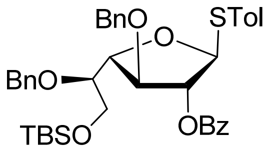

***p*-Tolyl 2-*O*-benzoyl-3,5-di-*O*-benzyl-6-*O*-*tert*-butydimethylsilyl-1-thio-*β*-D-galactofuranoside (18):** To a solution of **18g** (6.5 g, 13.9 mmol) and imidazole (2.8 g, 41.7 mmol) in anhydrous CH_2Cl_2 (30 mL) was added TBSCl (2.3 g, 15.3 mmol) at 0 °C, the mixture was stirred for 2 h at the same temperature. When TLC indicated the disappearance of **18g**, pyridine (11.2 mL, 139.3 mmol) and BzCl (1.9 mL, 16.7 mmol) was then added. The resulting solution was stirred overnight at room temperature, and quenched with CH_3OH (5 mL) and stirred for another 30 min. The solvent was removed under reduced pressure, and the residue was purified by column chromatography on silica gel (petroleum ether/acetone, 200:1) to afford **18** (8.3 g, 87% for two steps) as colorless syrup. R_f = 0.46 (petroleum ether/ethyl acetate, 8:1); $[\alpha]_D^{30}$ -107.7 (*c* 1.9, CH_2Cl_2); ^{1}H NMR (400 MHz, $CDCl_3$) δ 7.98 – 7.96 (m, 2H, Ar), 7.57 (t, *J* = 7.5 Hz, 1H, Ar), 7.42 – 7.38 (m, 4H, Ar), 7.32 – 7.28 (m, 4H, Ar), 7.25 – 7.14 (m, 6H, Ar), 7.07 (d, *J* = 8.0 Hz, 2H, Ar), 5.64 (s, 1H, H-1), 5.48 (t, *J* = 1.6 Hz, 1H, H-2), 4.72 (d, *J* = 12.0 Hz, 1H, PhCH_2), 4.68 (d, *J* = 11.5 Hz, 1H, PhCH_2), 4.51 (dd, *J* = 6.2,

2.5 Hz, 1H, H-4), 4.42 (d, J = 12.0 Hz, 1H, $PhCH_2$), 4.34 (d, J = 11.6 Hz, 1H, $PhCH_2$), 4.11 (d, J = 6.0 Hz, 1H, H-3), 3.82 (dd, J = 10.3, 6.7 Hz, 1H, H-6a), 3.77 (dd, J = 10.3, 5.8 Hz, 1H, H-6b), 3.64 (td, J = 6.1, 2.6 Hz, 1H, H-5), 2.31 (s, 3H, tolyl CH_3), 0.89 (s, 9H, *t*-Bu), 0.04 (s, 6H, Me); ^{13}C NMR (100 MHz, $CDCl_3$) δ 165.60 (C=O), 138.45 (Ar), 137.75 (Ar), 137.53 (Ar), 133.47 (Ar), 132.44 (Ar), 130.70 (Ar), 129.99 (Ar), 129.70 (Ar), 129.57 (Ar), 128.59 (Ar), 128.55 (Ar), 128.36 (Ar), 128.29 (Ar), 128.02 (Ar), 127.76 (Ar), 91.34 (C-1), 83.06 (C-3), 82.82 (C-2), 81.83 (C-4), 78.33 (C-5), 73.92 ($PhCH_2$), 72.45 ($PhCH_2$), 63.38 (C-6), 26.06 (*t*-Bu), 21.23 (tolyl CH_3), 18.40 (*t*-Bu), -5.25 (CH_3), -5.28 (CH_3); HRMS (ESI) Calcd for $C_{40}H_{52}NO_6SSi$ $[M + NH_4]^+$: 702.3279, found: 702.3279.

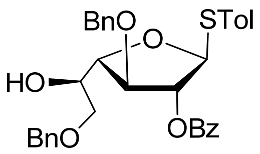

***p*-Tolyl 2-*O*-benzoyl-3,6-di-*O*-benzyl-1-thio-*β*-D-galactofuranoside (19):** To a solution of **18f** (6.9 g, 14.8 mmol) in anhydrous pyridine (50 mL) was added Bz_2O (4.0 g, 17.8 mmol) and a catalytic amount of DMAP (18 mg, 0.15 mmol). After stirring for 4 h at the room temperature, the solution was quenched with CH_3OH (5 mL) and stirred for another 30 min. The reaction mixture was then concentrated and the residue was purified by column chromatography on silica gel (petroleum ether/acetone, 12:1) to give a crude product, which was further purified by column chromatography on silica gel (CH_2Cl_2) to afford **19** (5.3 g, 63%) as colorless syrup. R_f = 0.39 (petroleum ether/ethyl acetate, 3:1); $[\alpha]_D^{30}$ -132.7 (*c* 2.2, CH_2Cl_2); ^1H NMR (400 MHz, $CDCl_3$) δ 8.02 – 8.00 (m, 2H, Ar), 7.58 (t, J = 7.4 Hz, 1H, Ar), 7.46 – 7.40 (m, 4H, Ar), 7.36 – 7.26 (m, 10H, Ar), 7.09 (d, J = 8.0 Hz, 2H, Ar), 5.60 (s, 1H, H-1), 5.53 (t, J = 1.6 Hz, 1H, H-2), 4.81 (d, J = 11.9 Hz, 1H, $PhCH_2$), 4.61 (d, J = 11.9 Hz, 1H, $PhCH_2$), 4.54 (d, J = 12.0 Hz, 1H, $PhCH_2$), 4.50 (d, J = 12.0 Hz, 1H, $PhCH_2$), 4.46 (dd, J = 5.8, 3.4 Hz, 1H, H-4), 4.25 (d, J = 5.8 Hz, 1H, H-3), 4.00 – 3.95 (m, 1H, H-5), 3.59 – 3.50 (m, 2H, H-6a, H-6b), 2.32 (s, 3H, tolyl CH_3), 2.30 (d, J = 6.5 Hz, 1H, -OH); ^{13}C NMR (100 MHz, $CDCl_3$) δ 165.51 (C=O), 138.01 (Ar), 137.96 (Ar),

137.50 (Ar), 133.63 (Ar), 132.99 (Ar), 130.31 (Ar), 129.94 (Ar), 129.85 (Ar), 129.40 (Ar), 128.63 (Ar), 128.58 (Ar), 128.14 (Ar), 128.07 (Ar), 127.90 (Ar), 127.85 (Ar), 91.78 (C-1), 83.35 (C-3), 82.53 (C-4), 82.15 (C-2), 73.62 ($PhCH_2$), 72.65 ($PhCH_2$), 71.68 (C-6), 69.75 (C-5), 21.26 (tolyl CH_3); HRMS (ESI) Calcd for $C_{34}H_{38}NO_6S$ [M + $NH_4]^+$: 588.2414, found: 588.2424.

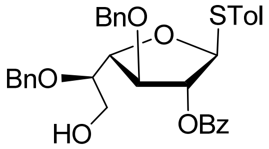

***p*-Tolyl 2-*O*-benzoyl-3,5-di-*O*-benzyl-1-thio-*β*-D-galactofuranoside (20):** To a solution of **18** (2.0 g, 2.92 mmol) in THF (10 mL) and H_2O (1 mL) was added 70% HF-pyridine (1 mL) at 0 °C, the resulting mixture was warmed to 35 °C and stirred for 4 h. Then the reaction solution was quenched with a saturated aqueous $NaHCO_3$ solution (40 mL), and extracted with EtOAc (2 × 60 mL). The combined organic layer, after being washed with 0.1 N HCl (2 × 40 mL), saturated aqueous $NaHCO_3$ solution (50 mL) and brine (40 mL), was dried over Na_2SO_4, filtered, and concentrated. The residue was purified by column chromatography on silica gel (petroleum ether/acetone, 6:1) to afford **20** (1.6 g, 96%) as colorless syrup. R_f = 0.41 (petroleum ether/ethyl acetate, 2:1); $[\alpha]_D^{30}$ -126.4 (*c* 2.2, CH_2Cl_2); ^1H NMR (400 MHz, $CDCl_3$) δ 8.01 (d, *J* = 8.2 Hz, 2H, Ar), 7.59 (t, *J* = 7.4 Hz, 1H, Ar), 7.45 – 7.26 (m, 9H, Ar), 7.23 – 7.21 (m, 5H, Ar), 7.10 (d, *J* = 8.1 Hz, 2H, Ar), 5.63 (s, 1H, H-1), 5.50 (d, *J* = 1.4 Hz, 1H, H-2), 4.79 (d, *J* = 11.8 Hz, 1H, $PhCH_2$), 4.61 – 4.58 (m, 2H, H-4, $PhCH_2$), 4.50 (d, *J* = 11.6 Hz, 2H, $PhCH_2$), 4.16 (d, *J* = 6.1 Hz, 1H, H-3), 3.80 – 3.69 (m, 3H, H-6a, H-6b, H-5), 2.31 (s, 3H, tolyl CH_3), 2.03 (br s, 1H, -OH); ^{13}C NMR (100 MHz, $CDCl_3$) δ 165.55 (C=O), 138.12 (Ar), 138.05 (Ar), 137.29 (Ar), 133.63 (Ar), 132.99 (Ar), 130.13 (Ar), 129.95 (Ar), 129.88 (Ar), 129.39 (Ar), 128.66 (Ar), 128.61 (Ar), 128.51 (Ar), 128.38 (Ar), 128.18 (Ar), 128.11 (Ar), 127.93 (Ar), 91.58 (C-1), 83.22 (C-3), 82.93 (C-4), 82.24 (C-2), 77.75 (C-5), 73.09 ($PhCH_2$), 72.58 ($PhCH_2$), 62.51 (C-6), 21.25 (tolyl CH_3); HRMS (ESI) Calcd for $C_{34}H_{38}NO_6S$ [M + $NH_4]^+$: 588.2414, found: 588.2408.

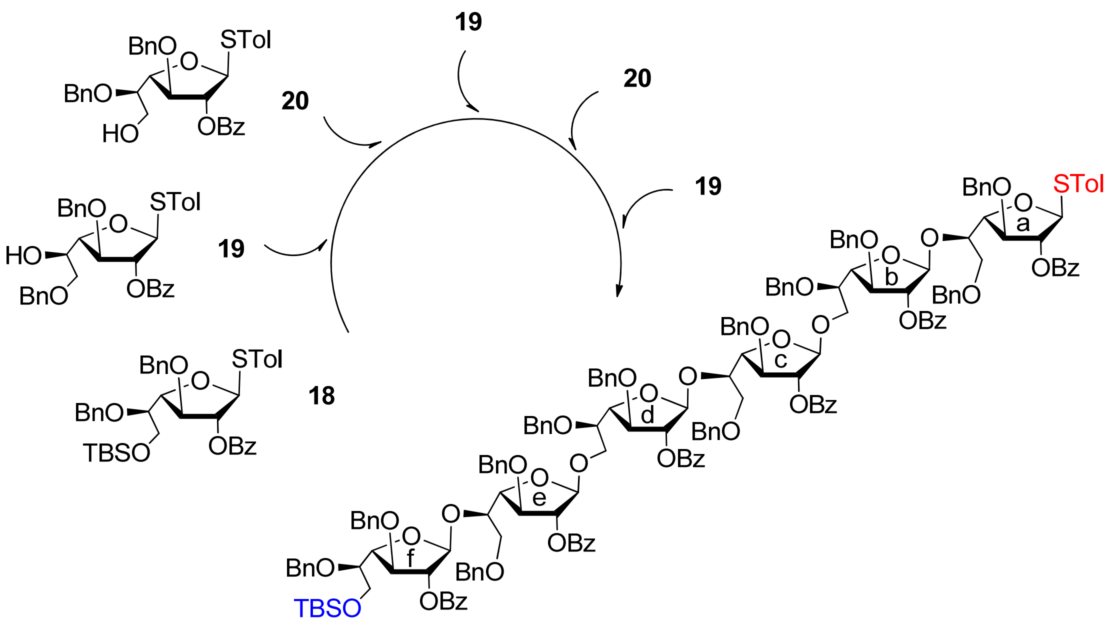

***p*-Tolyl 2-*O*-benzoyl-3,5-di-*O*-benzyl-6-*O*-*tert*-butydimethylsilyl-*β*-D-galactofuranosyl[f]-(1→5)-2-*O*-benzoyl-3,6-di-*O*-benzyl-*β*-D-galactofuranosyl[e]-(1→6)-2-*O*-benzoyl-3,5-di-*O*-benzyl-*β*-D-galactofuranosyl[d]-(1→5)-2-*O*-benzoyl-3,6-di-*O*-benzyl-*β*-D-galactofuranosyl[c]-(1→6)-2-*O*-benzoyl-3,5-di-*O*-benzyl-*β*-D-galactofuranosyl[b]-(1→5)-2-*O*-benzoyl-3,6-di-*O*-benzyl-1-thio-*β*-D-galactofuranoside[a] (8):** A mixture of **18** (550.0 mg, 802.97 μmol), TTBP (1.05 g, 4.22 mmol) and freshly activated 4 Å molecular sieves (7.0 g) in anhydrous CH_2Cl_2 (40 mL) under argon atmosphere was stirred for 20 min at room temperature, and cooled to -78 °C. 5 min later, stoichiometric *p*-TolSCl (110.8 μL, 802.97 μmol) was added, followed by dropwise addition of AgOTf (4.0 mL, 1.61 mmol, 0.4 mol/L in anhydrous toluene/CH_2Cl_2, 3:1). After another 5 min, a solution of **19** (436.4 mg, 764.73 μmol) in anhydrous CH_2Cl_2 (4 mL) was slowly added. The resulting mixture was slowly warmed to room temperature within 2 h and cooled back to -78 °C. 5 min later, stoichiometric *p*-TolSCl (105.5 μL, 764.73 μmol) was added, followed by dropwise addition of AgOTf (3.8 mL, 1.53 mmol, 0.4 mol/L in anhydrous toluene/CH_2Cl_2, 3:1). After another 5 min, a solution of **20** (414.6 mg, 726.49 μmol) in anhydrous CH_2Cl_2 (4 mL) was slowly added. The resulting mixture was stirred at -78 °C for 10 min, warmed to room temperature, and cooled back to -78 °C. 5 min later, stoichiometric *p*-TolSCl (100.2 μL, 726.49 μmol) was added, followed by dropwise addition of

AgOTf (3.6 mL, 1.45 mmol, 0.4 mol/L in anhydrous toluene/CH_2Cl_2, 3:1). After another 5 min, a solution of **19** (366.6 mg, 642.37 μmol) in anhydrous CH_2Cl_2 (4 mL) was slowly added. The resulting mixture was slowly warmed to room temperature within 2 h and cooled back to -78 °C. 5 min later, stoichiometric *p*-TolSCl (88.6 μL, 642.37 μmol) was added, followed by dropwise addition of AgOTf (3.2 mL, 1.28 mmol, 0.4 mol/L in anhydrous toluene/CH_2Cl_2, 3:1). After another 5 min, a solution of **20** (331.7 mg, 581.19 μmol) in anhydrous CH_2Cl_2 (4 mL) was slowly added. The resulting mixture was stirred at -78 °C for 10 min, warmed to room temperature, and cooled back to -78 °C. 5 min later, stoichiometric *p*-TolSCl (80.2 μL, 581.19 μmol) was added, followed by dropwise addition of AgOTf (2.9 mL, 1.16 mmol, 0.4 mol/L in anhydrous toluene/CH_2Cl_2, 3:1). After another 5 min, a solution of **19** (392.8 mg, 688.26 μmol) in anhydrous CH_2Cl_2 (0.6 mL) was slowly added. The resulting mixture was slowly warmed to room temperature within 2 h, quenched with Et_3N (1 mL) and filtered through Celite. After removal of the solvent, the desired product was purified by column chromatography on silica gel (petroleum ether/ethyl acetate, 5:1) to afford **8** (1.07 g, 63%) as white foam. R_f = 0.36 (petroleum ether/ethyl acetate, 3:1); $[\alpha]_D^{30}$ -72.2 (*c* 1.8, CH_2Cl_2); ^1H NMR (600 MHz, $CDCl_3$) δ 8.00 – 7.93 (m, 12H, Ar), 7.58 – 6.96 (m, 82H, Ar), 5.62 (s, 1H, H-1^f), 5.58 (br s, 2H, H-1^{a,d}), 5.51 (s, 1H, H-1^b), 5.48 (br s, 3H, H-2^{a,d,f}), 5.44 (s, 1H, H-2^b), 5.34 (s, 1H, H-2^e), 5.32 (d, *J* = 1.0 Hz, 1H, H-2^c), 5.04 (br s, 2H, H-1^{c,e}), 4.72 – 4.52 (m, 13H, $PhCH_2$, H-4^a), 4.43 (t, *J* = 11.8 Hz, 2H, $PhCH_2$), 4.37 – 4.22 (m, 17H, $PhCH_2$, H-3^{a,c,e}, H-4^{b,c,e,f}, H-5^{a,c,e}), 4.20 – 4.15 (m, 4H, H-4^d, H-5^{a,c,e}), 4.02 – 3.99 (m, 3H, H-3^{b,d,f}), 3.82 – 3.58 (m, 14H, H-5^{b,d}, H-6^{a,b,c,d,e,f}), 3.48 – 3.46 (m, 1H, H-5^f), 2.25 (s, 3H, tolyl CH_3), 0.79 (s, 9H, *t*-Bu), -0.09 (s, 3H, CH_3), -0.11 (s, 3H, CH_3); ^{13}C NMR (150 MHz, $CDCl_3$) δ 165.53 (C=O), 165.51 (C=O), 165.48 (C=O), 165.43 (C=O), 165.37 (C=O), 165.33 (C=O), 138.54 (Ar), 138.47 (Ar), 138.41 (Ar), 138.17 (Ar), 138.09 (Ar), 138.04 (Ar), 137.91 (Ar), 137.82 (Ar), 137.80 (Ar), 137.67 (Ar), 133.47 (Ar), 133.35 (Ar), 133.33 (Ar), 133.31 (Ar), 132.80 (Ar), 130.45 (Ar), 129.99 (Ar), 129.96 (Ar), 129.95 (Ar), 129.78 (Ar), 129.69 (Ar), 129.64 (Ar), 129.62 (Ar), 129.57 (Ar), 129.54 (Ar), 129.41 (Ar), 128.60 (Ar), 128.58 (Ar), 128.54 (Ar), 128.53 (Ar), 128.48 (Ar), 128.42 (Ar), 128.39 (Ar),

128.37 (Ar), 128.31 (Ar), 128.29 (Ar), 128.28 (Ar), 128.20 (Ar), 128.15 (Ar), 128.13 (Ar), 127.96 (Ar), 127.91 (Ar), 127.81 (Ar), 127.76 (Ar), 127.71 (Ar), 127.54 (Ar), 127.45 (Ar), 127.42 (Ar), 127.37 (Ar), 107.04 (C-1^{c}), 107.00 (C-1^{e}), 106.85 (C-1^{f}), 106.62 (C-1^{d}), 106.24 (C-1^{b}), 91.18 (C-1^{a}), 84.00 (C-3^{e}), 83.81 (C-3^{c}), 83.79 (C-4^{d}), 83.57 (C-3^{a}), 83.46 (×2, C-4^{b,f}), 83.31 (C-3), 83.26 (×2, C-3), 83.75 (C-4^{e}), 82.53 (C-4^{c}), 82.42 (C-2^{c}), 82.31 (C-2), 82.28 (C-2), 82.18 (C-2), 82.05 (×2, C-4^{a}, C-2), 81.98 (C-2), 78.91 (C-5^{f}), 77.08 (C-5^{d}), 76.76 (C-5^{b}), 74.20 (C-5^{a}), 74.12 (C-5^{e}), 74.11 ($PhCH_2$), 73.98 ($PhCH_2$), 73.92 ($PhCH_2$), 73.76 (C-5^{c}), 73.52 (×2, $PhCH_2$), 73.35 ($PhCH_2$), 72.98 ($PhCH_2$), 72.87 ($PhCH_2$), 72.68 ($PhCH_2$), 72.18 ($PhCH_2$), 72.08 (×2, $PhCH_2$), 71.62 (C-6), 71.60 (C-6), 70.73 (C-6^{a}), 69.87 (C-6^{b}), 69.51 (C-6^{d}), 64.77 (C-6^{f}), 25.94 (*t*-Bu), 21.21 (tolyl CH_3), 18.21 (*t*-Bu), -5.35 (×2, CH_3); HRMS (ESI) Calcd for $C_{175}H_{178}O_{36}SSiNa_2$ $[M + 2Na]^{2+}$: 1480.5686, found: 1480.5724.

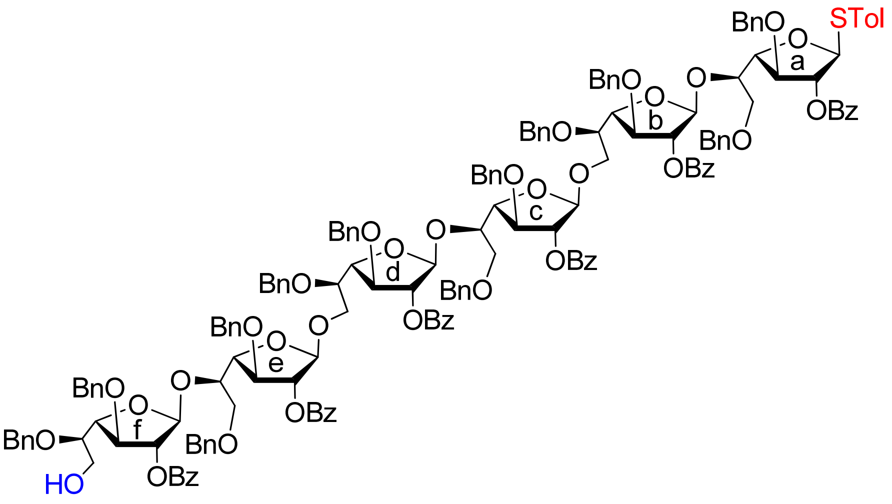

***p*-Tolyl 2-*O*-benzoyl-3,5-di-*O*-benzyl-*β*-D-galactofuranosyl[f]-(1→5)-2-*O*-benzoyl-3,6-di-*O*-benzyl-*β*-D-galactofuranosyl[e]-(1→6)-2-*O*-benzoyl-3,5-di-*O*-benzyl-*β*-D-galactofuranosyl[d]-(1→5)-2-*O*-benzoyl-3,6-di-*O*-benzyl-*β*-D-galactofuranosyl[c]-(1→6)-2-*O*-benzoyl-3,5-di-*O*-benzyl-*β*-D-galactofuranosyl[b]-(1→5)-2-*O*-benzoyl-3,6-di-*O*-benzyl-1-thio-*β*-D-galactofuranoside[a] (5):** To a solution of **8** (1.0 g, 0.343 mmol) in THF (10 mL) and H_2O (1 mL) was added 70% HF-pyridine (1 mL) at 0 °C, the resulting mixture was warmed to 35 °C and stirred for 4 h. Then the reaction solution was quenched with a saturated aqueous $NaHCO_3$ solution (30 mL), and extracted with EtOAc (2 × 50 mL). The combined organic layer, after being washed with 0.1 N HCl

(2 × 30 mL), saturated aqueous $NaHCO_3$ solution (40 mL) and brine (40 mL), was dried over Na_2SO_4, filtered, and concentrated. The residue was purified by column chromatography on silica gel (petroleum ether/acetone, 4:1) to afford **5** (0.83 g, 85%) as white foam. R_f = 0.29 (petroleum ether/ethyl acetate, 2:1); $[\alpha]_D^{30}$ -79.8 (*c* 1.6, CH_2Cl_2); ^{1}H NMR (600 MHz, $CDCl_3$) δ 8.00 – 7.93 (m, 12H, Ar), 7.59 – 6.98 (m, 82H, Ar), 5.58 (br s, 2H, H-1^a, H-1^d), 5.55 (s, 1H, H-1^f), 5.51 (s, 1H, H-1^b), 5.48 (br s, 3H, H-2^{a,d,f}), 5.44 (s, 1H, H-2^b), 5.33 (s, 1H, H-2^c), 5.30 (s, 1H, H-2^e), 5.05 (br s, 2H, H-1^{c,e}), 4.73 – 4.66 (m, 5H, $PhCH_2$), 4.62 – 4.53 (m, 7H, $PhCH_2$, H-4^a), 4.45 – 4.24 (m, 20H, $PhCH_2$, H-3^{a,c,e}, H-4^{b,c,e,f}), 4.19 – 4.15 (m, 4H, H-4^d, H-5^{a,c,e}), 4.01 (br s, 3H, H-3^{b,d,f}), 3.83 – 3.79 (m, 2H, H-6^{b,d}), 3.73 – 3.59 (m, 11H, H-5^{b,d}, H-6^{a,b,c,d,e,f}), 3.53 (dd, J = 11.0, 4.8 Hz, 1H, H-6^f), 3.433 – 3.427 (m, 1H, H-5^f), 2.25 (s, 3H, tolyl CH_3), 2.02 (br s, 1H. -OH); ^{13}C NMR (150 MHz, $CDCl_3$) δ 165.55 (C=O), 165.52 (C=O), 165.44 (C=O), 165.43 (C=O), 165.36 (C=O), 165.32 (C=O), 138.46 (Ar), 138.43 (Ar), 138.17 (Ar), 138.16 (Ar), 138.12 (Ar), 138.09 (Ar), 137.96 (Ar), 137.84 (Ar), 137.81 (Ar), 137.67 (Ar), 137.57 (Ar), 133.47 (Ar), 133.43 (Ar), 133.35 (Ar), 133.32 (Ar), 132.80 (Ar), 130.44 (Ar), 129.99 (Ar), 129.95 (Ar), 129.78 (Ar), 129.65 (Ar), 129.62 (Ar), 129.54 (Ar), 129.53 (Ar), 129.41 (Ar), 128.58 (Ar), 128.49 (Ar), 128.42 (Ar), 128.39 (Ar), 128.37 (Ar), 128.35 (Ar), 128.33 (Ar), 128.31 (Ar), 128.28 (Ar), 128.20 (Ar), 128.15 (Ar), 128.04 (Ar), 128.01 (Ar), 127.95 (Ar), 127.81 (Ar), 127.79 (Ar), 127.77 (Ar), 127.74 (Ar), 127.70 (Ar), 127.54 (Ar), 127.50 (Ar), 127.45 (Ar), 127.41 (Ar), 107.04 (C-1^c), 106.77 (C-1^f), 106.72 (C-1^e), 106.66 (C-1^d), 106.25 (C-1^b), 91.18 (C-1^a), 83.89 (C-3^c), 83.69, 83.66, 83.57, 83.43, 83.25 (×2, C-3), 82.94, 82.93, 82.84 (C-4), 82.60 (C-4^b), 82.40 (C-2^c), 82.28 (C-2), 82.13 (C-2), 82.06 (×2, C-4^a, C-2^b), 82.01 (C-2), 81.85 (C-2^e), 77.53 (C-5^f), 77.07 (C-5^d), 76.75 (C-5^b), 74.83 (C-5^e), 74.22 (C-5^a), 73.99 ($PhCH_2$), 73.95 ($PhCH_2$), 73.88 (C-5^c), 73.52 ($PhCH_2$), 73.39 ($PhCH_2$), 73.36 ($PhCH_2$), 72.92 ($PhCH_2$), 72.90 ($PhCH_2$), 72.67 ($PhCH_2$), 72.59 ($PhCH_2$), 72.21 ($PhCH_2$), 72.18 ($PhCH_2$), 72.07 ($PhCH_2$), 71.65 (C-6), 71.47 (C-6), 70.73 (C-6^a), 69.87 (C-6^b), 69.41 (C-6^d), 62.01 (C-6^f), 21.21 (tolyl CH_3); HRMS (ESI) Calcd for $C_{169}H_{164}O_{36}SNa_2$ $[M + 2Na]^{2+}$: 1423.5254, found: 1423.5251.

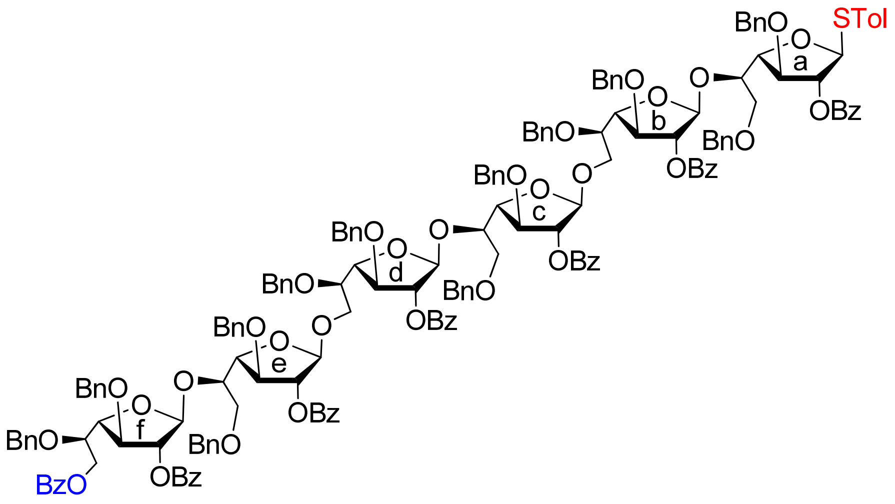

***p*-Tolyl 2,6-di-*O*-benzoyl-3,5-di-*O*-benzyl-*β*-D-galactofuranosyl[f]-(1→5)-2-*O*-benzoyl-3,6-di-*O*-benzyl-*β*-D-galactofuranosyl[e]-(1→6)-2-*O*-benzoyl-3,5-di-*O*-benzyl-*β*-D-galactofuranosyl[d]-(1→5)-2-*O*-benzoyl-3,6-di-*O*-benzyl-*β*-D-galactofuranosyl[c]-(1→6)-2-*O*-benzoyl-3,5-di-*O*-benzyl-*β*-D-galactofuranosyl[b]-(1→5)-2-*O*-benzoyl-3,6-di-*O*-benzyl-1-thio-*β*-D-galactofuranoside[a] (4):** To a solution of **5** (540 mg, 0.193 mmol) in anhydrous CH_2Cl_2 (10 mL) was added pyridine (310 μL, 3.853 mmol), Bz_2O (218 mg, 0.964 mmol) and a catalytic amount of DMAP (2.5 mg, 0.02 mmol), the reaction mixture was refluxed overnight. Then the reaction was quenched with CH_3OH (100 μL) and stirred for another 30 min. After removal of the solvent, the residue was dissolved in EtOAc (60 mL), washed with 0.1 N HCl (30 mL), saturated aqueous $NaHCO_3$ solution (30 mL) and brine (30 mL). The organic layer was dried over Na_2SO_4, filtered, and concentrated. The residue was purified by column chromatography on silica gel (petroleum ether/acetone, 4.5:1) to afford **4** (536 mg, 96%) as white foam. R_f = 0.39 (petroleum ether/ethyl acetate, 2:1); $[\alpha]_D^{30}$ -67.8 (*c* 1.2, CH_2Cl_2); ^1H NMR (600 MHz, $CDCl_3$) δ 8.00 – 7.93 (m, 12H, Ar), 7.86 (d, *J* = 7.3 Hz, 2H, Ar), 7.57 – 6.97 (m, 85H, Ar), 5.62 (s, 1H, H-1[f]), 5.58 (br s, 2H, H-1[a], H-1[d]), 5.513 – 5.509 (m, 2H, H-2[f], H-1[b]), 5.48 (br s, 2H, H-2[a], H-2[d]), 5.44 (s, 1H, H-2[b]), 5.32 (d, *J* = 1.0 Hz, 2H, H-2[c,e]), 5.052 (s, 1H, H-1[e]), 5.047 (s, 1H, H-1[c]), 4.72 – 4.67 (m, 5H, $PhCH_2$), 4.64 – 4.52 (m, 8H, $PhCH_2$, H-4[a]), 4.43 – 4.27 (m, 19H, $PhCH_2$, H-6[f], H-3[a,c,e], H-4[f], H-4[c]/H-4[e]), 4.24 – 4.15 (m, 6H, H-4[b,d], H-4[c]/H-4[e]), 4.07 (d, *J* = 5.6 Hz, 1H, H-3[f]), 4.02 – 4.00 (m, 2H, H-3[b], H-3[d]), 3.83 – 3.77 (m, 2H, H-6[b,d]), 3.75 – 3.58 (m, 11H, H-5[b,d,f], H-6[a,b,c,d,e,f]), 2.25 (s, 3H, tolyl CH_3); ^{13}C NMR (150 MHz, $CDCl_3$) δ

166.19 (C=O), 165.53 (C=O), 165.52 (C=O), 165.45 (C=O), 165.44 (C=O), 165.35 (C=O), 165.33 (C=O), 138.47 (Ar), 138.18 (Ar), 138.17 (Ar), 138.11 (Ar), 138.04 (Ar), 137.89 (Ar), 137.84 (Ar), 137.81 (Ar), 137.68 (Ar), 137.64 (Ar), 133.48 (Ar), 133.37 (Ar), 133.34 (Ar), 133.06 (Ar), 132.80 (Ar), 130.46 (Ar), 129.99 (Ar), 129.96 (Ar), 129.78 (Ar), 129.67 (Ar), 129.63 (Ar), 129.57 (Ar), 129.55 (Ar), 129.41 (Ar), 128.60 (Ar), 128.57 (Ar), 128.49 (Ar), 128.45 (Ar), 128.42 (Ar), 128.40 (Ar), 128.37 (Ar), 128.33 (Ar), 128.31 (Ar), 128.28 (Ar), 128.26 (Ar), 128.20 (Ar), 128.17 (Ar), 128.15 (Ar), 128.09 (Ar), 127.96 (Ar), 127.94 (Ar), 127.91 (Ar), 127.88 (Ar), 127.81 (Ar), 127.76 (Ar), 127.71 (Ar), 127.65 (Ar), 127.55 (Ar), 127.50 (Ar), 127.44 (Ar), 127.42 (Ar), 127.39 (Ar), 107.05 (C-1[c]), 106.93 (C-1[e]), 106.74 (C-1[f]), 106.63 (C-1[d]), 106.25 (C-1[b]), 91.18 (C-1[a]), 83.84, 83.79, 83.71, 83.58, 83.46, 83.36 (C-3), 83.26, 83.25, 82.97 (C-3[f]), 82.78, 82.56, 82.42 (C-2), 82.29 (C-2), 82.18 (C-2), 82.06 (×2, C-4[a], C-2), 82.01 (×2, C-2), 76.77 (C-5[b]), 75.91 (C-5[f]), 74.24 (C-5[e]), 74.21 (C-5[a]), 73.99 ($PhCH_2$), 73.94 ($PhCH_2$), 73.79 (C-5[c]), 73.52 ($PhCH_2$), 73.44 ($PhCH_2$), 73.40 ($PhCH_2$), 73.37 ($PhCH_2$), 72.89 ($PhCH_2$), 72.71 ($PhCH_2$), 72.68 ($PhCH_2$), 72.18 ($PhCH_2$), 72.11 ($PhCH_2$), 72.10 ($PhCH_2$), 71.64 (C-6), 71.57 (C-6), 70.74 (C-6[a]), 69.87 (C-6[b]), 69.50 (C-6[d]), 64.92 (C-6[f]), 21.22 (tolyl CH_3); HRMS (ESI) Calcd for $C_{176}H_{168}O_{37}SNa_2$ $[M + 2Na]^{2+}$: 1475.5385, found: 1475.5365.

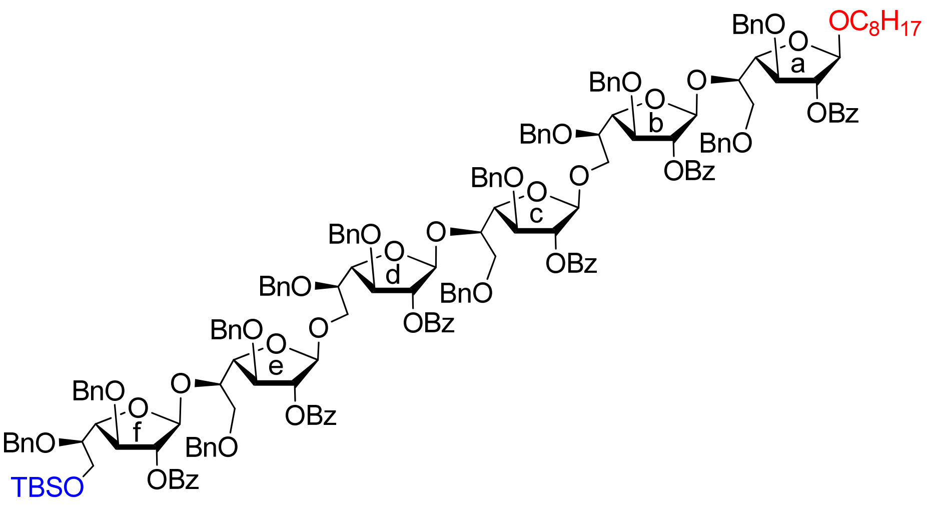

Octyl 2-*O*-benzoyl-3,5-di-*O*-benzyl-6-*O*-*tert*-butydimethylsilyl[f]-*β*-D-galactofuranosyl-(1→5)-2-*O*-benzoyl-3,6-di-*O*-benzyl-*β*-D-galactofuranosyl[e]-(1→6)-2-*O*-benzoyl-3,5-di-*O*-benzyl-*β*-D-galactofuranosyl[d]-(1→5)-2-*O*-benzoyl-3,6-di-

***O*-benzyl-*β*-D-galactofuranosyl[c]-(1→6)-2-*O*-benzoyl-3,5-di-*O*-benzyl-*β*-D-galactofuranosyl[b]-(1→5)-2-*O*-benzoyl-3,6-di-*O*-benzyl-*β*-D-galactofuranoside[a] (21):** A mixture of **8** (500 mg, 0.171 mmol), TTBP (51 mg, 0.205 mmol) and freshly activated 4 Å molecular sieves (2.0 g) in anhydrous CH_2Cl_2 (20 mL) under argon atmosphere was stirred for 20 min at room temperature, and cooled to -78 °C. 5 min later, stoichiometric *p*-TolSCl (23.6 μL, 0.171 mmol) was added, followed by dropwise addition of AgOTf (0.86 mL, 0.342 mmol, 0.4 mol/L in anhydrous toluene/CH_2Cl_2, 3:1). After another 5 min, 1-octanol (54 μL, 0.343 mmol) was added. The reaction mixture was warmed to room temperature in 10 min, quenched with Et_3N (0.5 mL) and filtered through Celite. After removal of the solvent, the desired product was purified by column chromatography on silica gel (petroleum ether/acetone, 6:1) to afford **21** (457 mg, 91%) as white foam. R_f = 0.44 (petroleum ether/ethyl acetate, 3:1); $[\alpha]_D^{30}$ -59.4 (*c* 0.9, CH_2Cl_2); ^1H NMR (600 MHz, $CDCl_3$) δ 8.02 – 7.92 (m, 12H, Ar), 7.58 – 7.48 (m, 4H, Ar), 7.44 – 6.96 (m, 74H, Ar), 5.62 (s, 1H, H-1[f]), 5.57 (s, 1H, H-1[d]), 5.55 (s, 1H, H-1[b]), 5.48 – 5.47 (m, 3H, H-2[b,d,f]), 5.33 – 5.33 (m, 3H, H-2[a,c,e]), 5.11 (s, 1H, H-1[a]), 5.06 (s, 1H, H-1[c]), 5.04 (s, 1H, H-1[e]), 4.72 – 4.65 (m, 6H, $PhCH_2$), 4.62 – 4.52 (m, 6H, $PhCH_2$), 4.47 (d, *J* = 12.0 Hz, 1H, $PhCH_2$), 4.44 (d, *J* = 12.0 Hz, 1H, $PhCH_2$), 4.37 – 4.23 (m, 18H, $PhCH_2$, H-3[a,c,e], H-4[a,b,c,e,f]), 4.19 – 4.14 (m, 4H, H-4[d], H-5[a,c,e]), 4.01 – 3.99 (m, 3H, H-3[b,d,f]), 3.85 – 3.60 (m, 15H, H-5[b,d], H-6[a,b,c,d,e,f], octyl OCH_2), 3.48 – 3.46 (m, 1H, H-5[f]), 3.41 (dt, *J* = 9.7, 6.5 Hz, 1H, octyl OCH_2), 1.57 – 1.54 (m, 2H, octyl CH_2), 1.30 – 1.23 (m, 10H, octyl CH_2), 0.85 (t, *J* = 6.9 Hz, 3H, octyl CH_3), 0.79 (s, 9H, *t*-Bu), -0.09 (s, 3H, CH_3), -0.11 (s, 3H, CH_3); ^{13}C NMR (150 MHz, $CDCl_3$) δ 165.68 (C=O), 165.51 (C=O), 165.48 (C=O), 165.42 (C=O), 165.38 (C=O), 165.34 (C=O), 138.54 (Ar), 138.47 (Ar), 138.42 (Ar), 138.22 (Ar), 138.17 (Ar), 138.07 (Ar), 138.05 (Ar), 137.92 (Ar), 137.87 (Ar), 137.82 (Ar), 133.37 (Ar), 133.34 (Ar), 133.32 (Ar), 130.01 (Ar), 130.00 (Ar), 129.97 (Ar), 129.95 (Ar), 129.70 (Ar), 129.66 (Ar), 129.65 (Ar), 129.62 (Ar), 129.58 (Ar), 129.55 (Ar), 128.59 (Ar), 128.58 (Ar), 128.55 (Ar), 128.53 (Ar), 128.42 (Ar), 128.39 (Ar), 128.37 (Ar), 128.33 (Ar), 128.30 (Ar), 128.26 (Ar), 128.21 (Ar), 128.17 (Ar), 128.14 (Ar), 127.98 (Ar), 127.95 (Ar), 127.92 (Ar), 127.81 (Ar), 127.77 (Ar), 127.71 (Ar), 127.53 (Ar),

127.47 (Ar), 127.45 (Ar), 127.43 (Ar), 127.40 (Ar), 127.38 (Ar), 107.04 (C-1[c]), 107.01 (C-1[e]), 106.85 (C-1[f]), 106.59 (C-1[d]), 106.25 (C-1[b]), 106.00 (C-1[a]), 84.01, 83.93, 83.82, 83.79, 83.52, 83.47, 83.31 (C-3), 83.30 (C-3), 83.28 (C-3), 82.76, 82.52, 82.48 (C-2), 82.42 (C-2), 82.32 (C-2), 82.19 (C-2), 82.08 (C-2), 82.00, 81.96, 78.92 (C-5[f]), 77.09 (C-5[d]), 74.13 (C-5[e]), 74.11 ($PhCH_2$), 74.00 (C-5[a]), 73.95 ($PhCH_2$), 73.94 ($PhCH_2$), 73.72 (C-5[c]), 73.50 ($PhCH_2$), 73.38 ($PhCH_2$), 73.35 ($PhCH_2$), 72.99 ($PhCH_2$), 72.88 ($PhCH_2$), 72.67 ($PhCH_2$), 72.13 ($PhCH_2$), 72.09 (×2, $PhCH_2$), 71.60 (C-6), 71.57 (C-6), 71.06 (C-6[a]), 69.85 (C-6[b]), 69.52 (C-6[d]), 67.64 (octyl OCH_2), 64.78 (C-6[f]), 31.99 (octyl CH_2), 29.59 (octyl CH_2), 29.56 (octyl $OCH_2\underline{C}H_2$), 29.39 (octyl CH_2), 26.24 (octyl CH_2), 25.95 (*t*-Bu), 22.80 (octyl CH_2), 18.22 (*t*-Bu), 14.25 (octyl CH_3), -5.35(×2, CH_3); HRMS (ESI) Calcd for $C_{176}H_{188}O_{37}SiNa_2$ $[M + 2Na]^{2+}$: 1483.6192, found: 1483.6171.

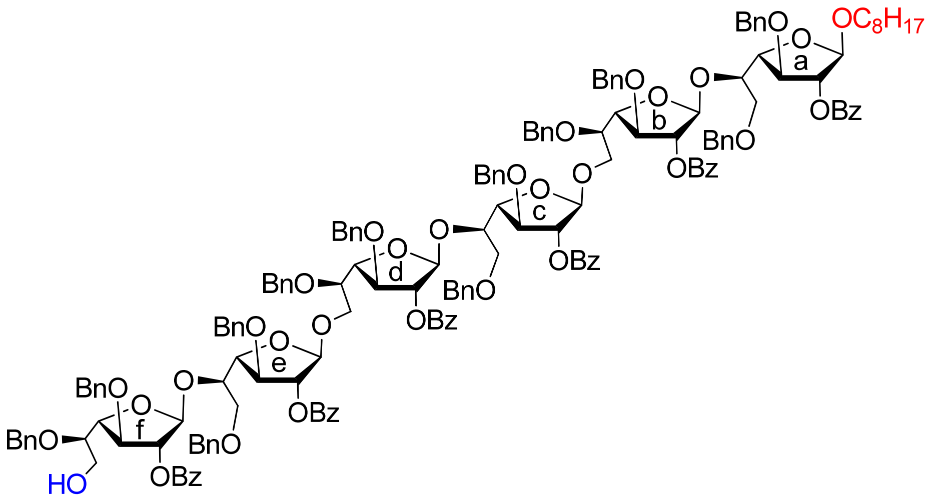

Octyl 2-*O*-benzoyl-3,5-di-*O*-benzyl-*β*-D-galactofuranosyl[f]-(1→5)-2-*O*-benzoyl-3,6-di-*O*-benzyl-*β*-D-galactofuranosyl[e]-(1→6)-2-*O*-benzoyl-3,5-di-*O*-benzyl-*β*-D-galactofuranosyl[d]-(1→5)-2-*O*-benzoyl-3,6-di-*O*-benzyl-*β*-D-galactofuranosyl[c]-(1→6)-2-*O*-benzoyl-3,5-di-*O*-benzyl-*β*-D-galactofuranosyl[b]-(1→5)-2-*O*-benzoyl-3,6-di-*O*-benzyl-*β*-D-galactofuranoside[a] (6): To a solution of **21** (440 mg, 0.151 mmol) in THF (10 mL) and H_2O (1 mL) was added 70% HF-pyridine (1 mL) at 0 °C, the resulting mixture was warmed to 35 °C and stirred for 4 h. Then the reaction solution was quenched with a saturated aqueous $NaHCO_3$ solution (30 mL), and extracted with EtOAc (2 × 40 mL). The combined organic layer, after being washed with 0.1 N HCl

(2 × 30 mL), saturated aqueous $NaHCO_3$ solution (30 mL) and brine (30 mL), was dried over Na_2SO_4, filtered, and concentrated. The residue was purified by column chromatography on silica gel (petroleum ether/acetone, 4.5:1) to afford **6** (374 mg, 87%) as white foam. R_f = 0.33 (petroleum ether/ethyl acetate, 2:1); $[\alpha]_D^{30}$ -61.7 (*c* 1.7, CH_2Cl_2); ^1H NMR (600 MHz, $CDCl_3$) δ 8.02 – 7.94 (m, 12H, Ar), 7.59 – 7.49 (m, 4H, Ar), 7.44 – 6.97 (m, 74H, Ar), 5.58 (s, 1H, H-1^d), 5.55 (br s, 2H, H-1^{b,f}), 5.49 (br s, 3H, H-2^{b,d,f}), 5.34 (s, 1H, H-2^c), 5.33 (s, 1H, H-2^a), 5.30 (s, 1H, H-2^e), 5.11 (s, 1H, H-1^a), 5.07 (s, 1H, H-1^c), 5.05 (s, 1H, H-1^e), 4.73 – 4.66 (m, 5H, $PhCH_2$), 4.62 – 4.53 (m, 6H, $PhCH_2$), 4.48 – 4.43 (m, 3H, $PhCH_2$), 4.38 – 4.24 (m, 18H, $PhCH_2$, H-3^{a,c,e}, H-4^{a,b,c,e,f}), 4.19 – 4.14 (m, 4H, H-4^d, H-5^{a,c,e}), 4.02 – 4.01 (m, 3H, H-3^{b,d,f}), 3.86 – 3.80 (m, 2H, H-6^{b,d}), 3.76 – 3.59 (m, 12H, H-5^{b,d}, H-6^{a,b,c,d,e,f}, octyl OCH_2), 3.54 – 3.51 (m, 1H, H-6^f), 3.44 – 3.39 (m, 2H, H-5^f, octyl OCH_2), 2.02 (br s, 1H, -OH), 1.59 – 1.54 (m, 2H, octyl CH_2), 1.31 – 1.23 (m, 10H, octyl CH_2), 0.86 (t, J = 6.9 Hz, 3H, octyl CH_3); ^{13}C NMR (150 MHz, $CDCl_3$) δ 165.67 (C=O), 165.55 (C=O), 165.45 (C=O), 165.40 (C=O), 165.36 (C=O), 165.32 (C=O), 138.47 (Ar), 138.43 (Ar), 138.22 (Ar), 138.17 (Ar), 138.15 (Ar), 138.12 (Ar), 138.09 (Ar), 138.07 (Ar), 137.96 (Ar), 137.87 (Ar), 137.83 (Ar), 137.58 (Ar), 133.43 (Ar), 133.36 (Ar), 133.35 (Ar), 133.33 (Ar), 130.01 (Ar), 129.96 (Ar), 129.93 (Ar), 129.66 (Ar), 129.62 (Ar), 129.55 (Ar), 129.53 (Ar), 128.58 (Ar), 128.42 (Ar), 128.37 (Ar), 128.32 (Ar), 128.26 (Ar), 128.16 (Ar), 128.15 (Ar), 128.05 (Ar), 128.01 (Ar), 127.94 (Ar), 127.79 (Ar), 127.77 (Ar), 127.74 (Ar), 127.70 (Ar), 127.52 (Ar), 127.50 (Ar), 127.46 (Ar), 127.42 (Ar), 107.03 (C-1^c), 106.77 (C-1^f), 106.72 (C-1^e), 106.64 (C-1^d), 106.26 (C-1^b), 105.99 (C-1^a), 83.92, 83.90, 83.69, 83.67, 83.50, 83.31 (C-3), 83.24 (C-3), 82.94, 82.93, 82.84, 82.58, 82.47 (C-2), 82.40 (C-2), 82.13 (C-2), 82.08 (C-2), 82.02 (C-2), 81.96, 81.85 (C-2^e), 77.53 (C-5^f), 77.07 (C-5^d), 76.89 (C-5^b), 74.83 (C-5^e), 74.00 (C-5^a), 73.95 (×2, $PhCH_2$), 73.83 (C-5^c), 73.49 ($PhCH_2$), 73.39 ($PhCH_2$), 73.38 ($PhCH_2$), 72.93 ($PhCH_2$), 72.90 ($PhCH_2$), 72.66 ($PhCH_2$), 72.60 ($PhCH_2$), 72.21 ($PhCH_2$), 72.11 ($PhCH_2$), 72.07 ($PhCH_2$), 71.60 (C-6), 71.47 (C-6), 71.06 (C-6^a), 69.85 (C-6^b), 69.42 (C-6^d), 67.62 (octyl OCH_2), 62.01 (C-6^f), 31.97 (octyl CH_2), 29.58 (octyl CH_2), 29.55 (octyl $OCH_2\underline{C}H_2$), 29.38 (octyl CH_2), 26.23 (octyl CH_2), 22.79 (octyl CH_2), 14.25

(octyl CH_3); HRMS (ESI) Calcd for $C_{170}H_{174}O_{37}Na_2$ $[M + 2Na]^{2+}$: 1426.5759, found: 1426.5749.

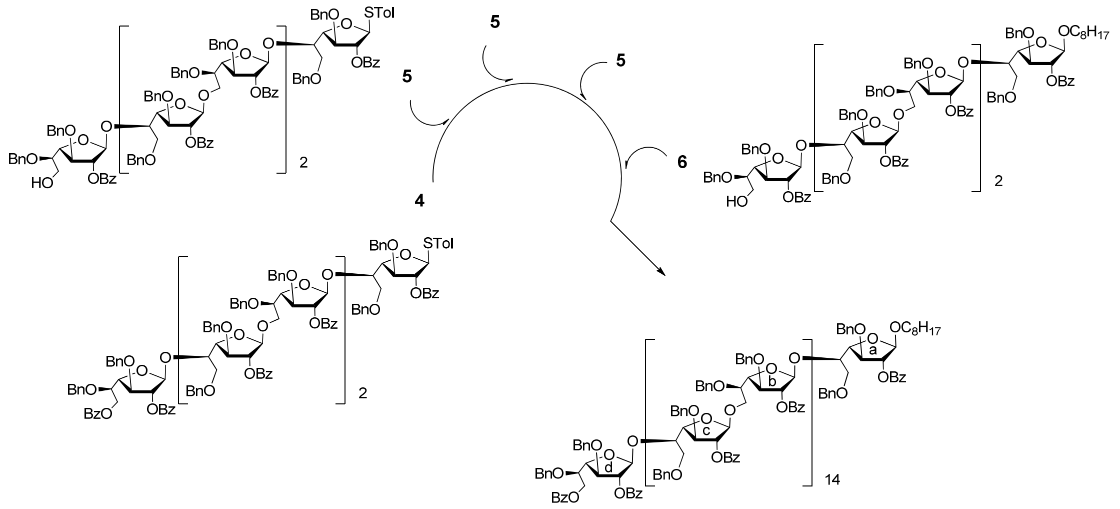

Octyl 2,6-di-*O*-benzoyl-3,5-di-*O*-benzyl-*β*-D-galactofuranosyl^{d}-(1→5)-2-*O*-benzoyl-3,6-di-*O*-benzyl-*β*-D-galactofuranosyl^{c14}-(1→6)-2-*O*-benzoyl-3,5-di-*O*-benzyl-*β*-D-galactofuranosyl^{b14}-(1→5)-2-*O*-benzoyl-3,6-di-*O*-benzyl-*β*-D-galactofuranosyl^{c13}-(1→6)-2-*O*-benzoyl-3,5-di-*O*-benzyl-*β*-D-galactofuranosyl^{b13}-(1→5)-2-*O*-benzoyl-3,6-di-*O*-benzyl-*β*-D-galactofuranosyl^{c12}-(1→6)-2-*O*-benzoyl-3,5-di-*O*-benzyl-*β*-D-galactofuranosyl^{b12}-(1→5)-2-*O*-benzoyl-3,6-di-*O*-benzyl-*β*-D-galactofuranosyl^{c11}-(1→6)-2-*O*-benzoyl-3,5-di-*O*-benzyl-*β*-D-galactofuranosyl^{b11}-(1→5)-2-*O*-benzoyl-3,6-di-*O*-benzyl-*β*-D-galactofuranosyl^{c10}-(1→6)-2-*O*-benzoyl-3,5-di-*O*-benzyl-*β*-D-galactofuranosyl^{b10}-(1→5)-2-*O*-benzoyl-3,6-di-*O*-benzyl-*β*-D-galactofuranosyl^{c9}-(1→6)-2-*O*-benzoyl-3,5-di-*O*-benzyl-*β*-D-galactofuranosyl^{b9}-(1→5)-2-*O*-benzoyl-3,6-di-*O*-benzyl-*β*-D-galactofuranosyl^{c8}-(1→6)-2-*O*-benzoyl-3,5-di-*O*-benzyl-*β*-D-galactofuranosyl^{b8}-(1→5)-2-*O*-benzoyl-3,6-di-*O*-benzyl-*β*-D-galactofuranosyl^{c7}-(1→6)-2-*O*-benzoyl-3,5-di-*O*-benzyl-*β*-D-galactofuranosyl^{b7}-(1→5)-2-*O*-benzoyl-3,6-di-*O*-benzyl-*β*-D-galactofuranosyl^{c6}-(1→6)-2-*O*-benzoyl-3,5-di-*O*-benzyl-*β*-D-galactofuranosyl^{b6}-(1→5)-2-*O*-benzoyl-3,6-di-*O*-benzyl-*β*-D-galactofuranosyl^{c5}-(1→6)-2-*O*-benzoyl-3,5-di-*O*-benzyl-*β*-D-galactofuranosyl^{b5}-(1→5)-2-*O*-benzoyl-3,6-di-*O*-benzyl-*β*-D-galactofuranosyl^{c4}-(1→6)-2-*O*-benzoyl-3,5-di-*O*-benzyl-*β*-D-galactofuranosyl^{b4}-(1→5)-2-*O*-benzoyl-3,6-di-*O*-benzyl-*β*-D-galactofuranosyl^{c3}-(1→6)-2-

***O*-benzoyl-3,5-di-*O*-benzyl-*β*-D-galactofuranosyl[b3]-(1→5)-2-*O*-benzoyl-3,6-di-*O*-benzyl-*β*-D-galactofuranosyl[c2]-(1→6)-2-*O*-benzoyl-3,5-di-*O*-benzyl-*β*-D-galactofuranosyl[b2]-(1→5)-2-*O*-benzoyl-3,6-di-*O*-benzyl-*β*-D-galactofuranosyl[c1]-(1→6)-2-*O*-benzoyl-3,5-di-*O*-benzyl-*β*-D-galactofuranosyl[b1]-(1→5)-2-*O*-benzoyl-3,6-di-*O*-benzyl-*β*-D-galactofuranoside[a] (22):** A mixture of **4** (25.00 mg, 8.60 µmol), TTBP (8.8 mg, 35.3 µmol) and freshly activated 4 Å molecular sieves (400 mg) in anhydrous CH_2Cl_2 (1.5 mL) under argon atmosphere was stirred for 20 min at room temperature, and cooled to -78 °C. 5 min later, stoichiometric *p*-TolSCl (29.7 µL, 8.60 µmol, 4% in anhydrous toluene) was added, followed by dropwise addition of AgOTf (65 µL, 25.80 µmol, 0.4 mol/L in anhydrous toluene/CH_2Cl_2, 3:1). After another 5 min, a solution of **5** (21.70 mg, 7.74 µmol) in anhydrous CH_2Cl_2 (0.6 mL) was slowly added. The resulting mixture was slowly warmed to room temperature within 2 h and cooled back to -78 °C. 5 min later, stoichiometric *p*-TolSCl (26.7 µL, 7.74 µmol, 4% in anhydrous toluene) was added, followed by dropwise addition of AgOTf (39 µL, 15.5 µmol, 0.4 mol/L in anhydrous toluene/CH_2Cl_2, 3:1). After another 5 min, a solution of **5** (19.29 mg, 6.88 µmol) in anhydrous CH_2Cl_2 (0.6 mL) was slowly added. The resulting mixture was slowly warmed to room temperature within 2 h and cooled back to -78 °C. 5 min later, stoichiometric *p*-TolSCl (23.7 µL, 6.88 µmol, 4% in anhydrous toluene) was added, followed by dropwise addition of AgOTf (35 µL, 13.8 µmol, 0.4 mol/L in anhydrous toluene/CH_2Cl_2, 3:1). After another 5 min, a solution of **5** (16.87 mg, 6.02 µmol) in anhydrous CH_2Cl_2 (0.6 mL) was slowly added. The resulting mixture was slowly warmed to room temperature within 2 h and cooled back to -78 °C. 5 min later, stoichiometric *p*-TolSCl (20.8 µL, 6.02 µmol, 4% in anhydrous toluene) was added, followed by dropwise addition of AgOTf (30 µL, 12.0 µmol, 0.4 mol/L in anhydrous toluene/CH_2Cl_2, 3:1). After another 5 min, a solution of **6** (19.33 mg, 6.88 µmol) in anhydrous CH_2Cl_2 (0.6 mL) was slowly added. The resulting mixture was slowly warmed to room temperature within 2 h, quenched with Et_3N (50 µL) and filtered through Celite. After removal of the solvent, the desired product was purified by column chromatography on silica gel (petroleum ether/acetone, 1.9:1) to give a crude product, which was further purified by size exclusion chromatography

(Bio-Beads S-X1, toluene/ethyl acetate, 1:1) to afford **22** (56.0 mg, 68%) as white foam. R_f = 0.40 (petroleum ether/acetone, 1.5:1); $[\alpha]_D^{30}$ -60.2 (*c* 1.2,CH_2Cl_2); ^{1}H NMR (600 MHz, $CDCl_3$) δ 8.02 – 7.92 (m, 60H, Ar), 7.86 – 7.85 (m, 2H, Ar), 7.57 – 6.95 (m, 393H, Ar), 5.62 (s, 1H, H-1^{d}), 5.57 – 5.55 (m, 14H, H-1^{b1-b14}), 5.52 (s, 1H, H-2^{d}), 5.48 (br s, 14H, H-2^{b1-b14}), 5.33 – 5.31 (m, 15H, H-2$^{a,c1-c14}$), 5.11 (s, 1H, H-1^{a}), 5.06 – 5.03 (m, 14H, H-1^{c1-c14}), 4.73 – 4.13 (m, 182H, $PhCH_2$, H-3$^{a,c1-c14}$, H-4$^{a,b1-b14,c1-c14,d}$, H-5$^{a,c1-c14}$, H-6^{d}), 4.07 (d, J = 5.8 Hz, 1H, H-3^{d}), 4.02 – 3.99 (m, 14H, H-3^{b1-b14}), 3.86 – 3.57 (m, 74H, H-5$^{b1-b14,d}$, H-6$^{a,b1-b14,c1-c14}$, octyl OCH_2), 3.41 (dt, J = 9.6, 6.6 Hz, 1H, octyl OCH_2), 1.59 – 1.54 (m, 2H, octyl CH_2), 1.26 – 1.23 (m, 10H, octyl CH_2), 0.85 (t, J = 7.0 Hz, 3H, octyl CH_3); ^{13}C NMR (150 MHz, $CDCl_3$) δ 166.19 (C=O), 165.67 (C=O), 165.50 (×13, C=O), 165.45 (C=O), 165.42 (C=O), 165.34 (×13, C=O), 138.46 (Ar), 138.42 (Ar), 138.21 (Ar), 138.12 (Ar), 138.05 (Ar), 138.02 (Ar), 137.87 (Ar), 137.85 (Ar), 137.78 (Ar), 138.62 (Ar), 133.35 (Ar), 133.06 (Ar), 129.97 (Ar), 129.93 (Ar), 129.66 (Ar), 129.60 (Ar), 129.55 (Ar), 129.50 (Ar), 128.59 (Ar), 128.56 (Ar), 128.45 (Ar), 128.41 (Ar), 128.36 (Ar), 128.33 (Ar), 128.29 (Ar), 128.25 (Ar), 128.14 (Ar), 128.12 (Ar), 128.08 (Ar), 128.01 (Ar), 127.94 (Ar), 127.90 (Ar), 127.77 (Ar), 127.71 (Ar), 127.66 (Ar), 127.57 (Ar), 127.52 (Ar), 127.50 (Ar), 127.45 (Ar), 127.40 (Ar), 127.37 (Ar), 107.02 (×13, C-1^{c1-c13}), 106.91 (C-1^{c14}), 106.73 (C-1^{d}), 106.62 (×13, C-1^{b2-b14}), 106.24 (C-1^{b1}), 105.99 (C-1^{a}), 83.91, 83.84, 83.70, 83.52, 83.31, 83.24, 82.95, 82.76, 82.47, 82.40, 82.17, 82.07, 81.97, 75.90 (C-5^{d}), 74.24, 73.98, 73.92, 73.89, 73.75, 73.49, 73.44, 73.38, 73.36, 73.32, 72.89, 72.71, 72.66, 72.12, 72.07, 71.58, 71.55, 71.05, 69.81, 69.53, 69.46, 67.63 (octyl OCH_2), 64.93 (C-6^{d}), 31.97 (octyl CH_2), 29.57 (octyl CH_2), 29.54 (octyl $OCH_2\underline{C}H_2$), 29.38 (octyl CH_2), 26.22 (octyl CH_2), 22.79 (octyl CH_2), 14.24 (octyl CH_3); MALDI-TOF MS Calcd for $C_{825}H_{802}O_{182}Na$ $[M + Na]^+$ *m/z*: 13652.1, found: 13650.8.

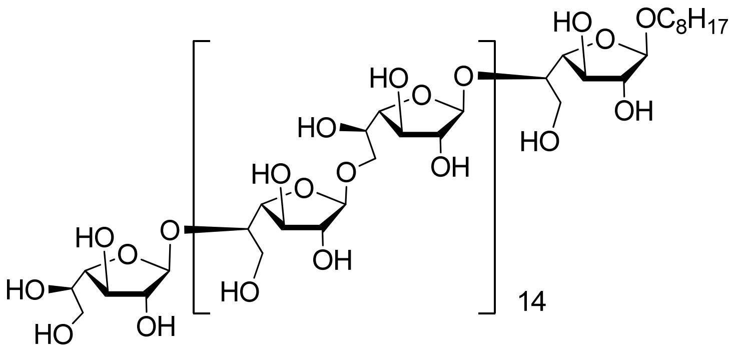

Octyl *β*-D-galactofuranosyl-(1→5)-*β*-D-galactofuranosyl-(1→6)-*β*-D-galactofuranosyl-(1→5)-*β*-D-galactofuranosyl-(1→6)-*β*-D-galactofuranosyl-(1→5)-*β*-D-galactofuranosyl-(1→6)-*β*-D-galactofuranosyl-(1→5)-*β*-D-galactofuranosyl-(1→6)-*β*-D-galactofuranosyl-(1→5)-*β*-D-galactofuranosyl-(1→6)-*β*-D-galactofuranosyl-(1→5)-*β*-D-galactofuranosyl-(1→6)-*β*-D-galactofuranosyl-(1→5)-*β*-D-galactofuranosyl-(1→6)-*β*-D-galactofuranosyl-(1→5)-*β*-D-galactofuranosyl-(1→6)-*β*-D-galactofuranosyl-(1→5)-*β*-D-galactofuranosyl-(1→6)-*β*-D-galactofuranosyl-(1→5)-*β*-D-galactofuranosyl-(1→6)-*β*-D-galactofuranosyl-(1→5)-*β*-D-galactofuranosyl-(1→6)-*β*-D-galactofuranosyl-(1→5)-*β*-D-galactofuranosyl-(1→6)-*β*-D-galactofuranosyl-(1→5)-*β*-D-galactofuranosyl-(1→6)-*β*-D-galactofuranosyl-(1→5)-*β*-D-galactofuranosyl-(1→6)-*β*-D-galactofuranosyl-(1→5)-*β*-D-galactofuranoside (23): To a solution of **22** (20.0 mg, 1.47 µmol) in CH_2Cl_2 (2 mL) and CH_3OH (4 mL) was added CH_3ONa (5.0 M in CH_3OH) to adjust pH to ~10, the reaction mixture was stirred for 24 h at room temperature, and neutralized by the addition of Dowex®50WX4 ion-exchange resin. The solution was filtered and concentrated. Purification by size exclusion chromatography (Bio-Beads S-X1, ethyl acetate as eluent) gave a residue, which was dissolved in a mixture of EtOAc/THF/1-PrOH/H_2O (6 mL, 2:1:1:1) with Pd/C (100 mg, 10% Pd content). The resulting solution was stirred under an atmosphere of hydrogen (0.4 MPa) for 48 h at room temperature, filtered through Celite, and concentrated to give a crude product, which was purified by gel filtration (Sephadex LH-20, CH_3OH/H_2O, 1:1) to afford **23** (5.7 mg, 78% for two steps) as glassy solid. ^1H NMR (600 MHz, D_2O) δ 5.22 (d, J = 1.7 Hz, 14H, H-1), 5.21 (d, J = 2.0 Hz, 1H, H-1), 5.01 (br s, 14H, H-1), 4.96 (d, J = 2.2 Hz, 1H, H-1), 4.14 – 3.61 (m, 181H), 3.57 (dt, J = 9.9, 6.6 Hz, 1H, octyl OCH_2), 1.62 – 1.57 (m, 2H, octyl CH_2),

1.35 – 1.24 (m, 10H, octyl CH_2), 0.86 (t, *J* = 6.8 Hz, 3H, octyl CH_3), ^{13}C NMR (150 MHz, D_2O) δ 108.70 (C-1), 108.63 (C-1), 107.91 (C-1), 107.82 (C-1), 83.82, 83.42, 82.73, 82.67, 82.24, 82.08, 82.04, 81.89, 81.84, 77.54, 77.49, 77.45, 77.32, 77.29, 76.79, 76.66, 76.49, 71.35, 70.41, 70.34, 70.12, 69.50, 63.63, 61.92, 61.83, 31.93 (octyl CH_2), 29.47 (octyl CH_2), 29.24 (octyl CH_2), 29.21 (octyl CH_2), 26.02 (octyl CH_2), 22.86 (octyl CH_2), 14.27 (octyl CH_3); MALDI-TOF MS Calcd for $C_{188}H_{318}O_{151}Na$ $[M + Na]^+$ *m/z*: 5017.4, found: 5018.1.

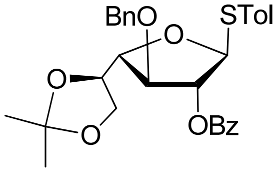

***p*-Tolyl 2-*O*-benzoyl-3-*O*-benzyl-5,6-*O*-isopropylidene-1-thio-*β*-D-galactofuranoside (24c):** To a solution of **18c** (9.0 g, 16.96 mmol) in THF (30 mL) was added TBAF (25.5 mL, 25.5 mmol, 1 M in THF) at room temperature. After stirring for 30 min, the reaction mixture was concentrated to give a crude residue, which was dissolved in pyridine (50 mL), the resulting mixture was added a catalytic amount of DMAP (208 mg, 1.70 mmol) and BzCl (4.9 mL, 42.40 mmol) at 0 °C. After stirring for 36 h at room temperature, the solution was quenched with CH_3OH (5 mL) and stirred for another 30 min. The solvent was evaporated *in vacuo* and the crude residue was purified by column chromatography on silica gel (petroleum ether/acetone, 35:1) to afford **24c** (8.33 g, 94% for two steps) as colorless syrup. R_f = 0.49 (petroleum ether/ethyl acetate, 4:1); $[\alpha]_D^{30}$ -136.5 (*c* 0.3, CH_2Cl_2); ^{1}H NMR (400 MHz, $CDCl_3$) δ 8.03 (d, *J* = 7.5 Hz, 2H, Ar), 7.59 (t, *J* = 7.4 Hz, 1H, Ar), 7.47 – 7.43 (m, 4H, Ar), 7.38 – 7.28 (m, 5H, Ar), 7.11 (d, *J* = 7.9 Hz, 2H, Ar), 5.62 (s, 1H, H-1), 5.56 (s, 1H, H-2), 4.85 (d, *J* = 11.9 Hz, 1H, $PhCH_2$), 4.62 (d, *J* = 11.9 Hz, 1H, $PhCH_2$), 4.42 (t, *J* = 5.4 Hz, 1H, H-4), 4.27 (dd, *J* = 12.5, 6.5 Hz, 1H, H-5), 4.01 (d, *J* = 5.2 Hz, 1H, H-3), 3.91 – 3.87 (m, 1H, H-6a), 3.85 – 3.81 (m, 1H, H-6b), 2.32 (s, 3H, tolyl CH_3), 1.40 (s, 1H, *i*-Pr), 1.34 (s, 1H, *i*-Pr); ^{13}C NMR (100 MHz, $CDCl_3$) δ 165.48 (C=O), 137.99 (Ar), 137.38 (Ar), 133.66 (Ar), 133.00 (Ar), 130.39 (Ar), 129.95 (Ar),

129.89 (Ar), 129.49 (Ar), 128.66 (Ar), 128.60 (Ar), 128.29 (Ar), 128.12 (Ar), 109.94 (*i*-Pr), 91.82 (C-1), 83.71 (C-3), 82.96 (C-4), 82.05 (C-2), 75.52 (C-5), 72.54 ($PhCH_2$), 65.60 (C-6), 26.52 (*i*-Pr), 25.49 (*i*-Pr), 21.25 (tolyl CH_3); HRMS (ESI) Calcd for $C_{30}H_{32}O_6SNa$ $[M + Na]^+$: 543.1812, found: 543.1819.

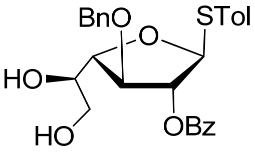

***p*-Tolyl 2-*O*-benzoyl-3-*O*-benzyl-1-thio-*β*-D-galactofuranoside (24d):** A solution of **24c** (3.4 g, 6.53 mmol) in 70% AcOH aqueous solution (50 mL) was stirred overnight at 50 °C. When TLC indicated the disappearance of starting material, the solution was concentrated *in vacuo*. The residue was then purified by column chromatography on silica gel (petroleum ether/acetone, 4:1) to yield **24d** (2.93 g, 93%) as white foam. R_f = 0.28 (petroleum ether/ethyl acetate, 1:1); $[\alpha]_D^{30}$ -147.8 (*c* 0.4, CH_2Cl_2); ^1H NMR (400 MHz, $CDCl_3$) δ 8.02 – 8.00 (m, 2H, Ar), 7.60 – 7.56 (m, 1H, Ar), 7.46 – 7.40 (m, 4H, Ar), 7.36 – 7.25 (m, 5H, Ar), 7.12 (d, *J* = 8.0 Hz, 2H, Ar), 5.57 (s, 1H, H-1), 5.52 (t, *J* = 1.7 Hz, 1H, H-2), 4.83 (d, *J* = 11.8 Hz, 1H, $PhCH_2$), 4.61 (d, *J* = 11.9 Hz, 1H, $PhCH_2$), 4.44 (dd, *J* = 6.0, 3.4 Hz, 1H, H-4), 4.24 (dd, *J* = 6.0, 1.1 Hz, 1H, H-3), 3.81 (br s, 1H, H-5), 3.72 – 3.68 (m, 2H, H-6a, H-6b), 2.52 (d, *J* = 7.5 Hz, 1H, -OH), 2.32 (s, 3H, tolyl CH_3), 2.16 (br s, 1H, -OH); ^{13}C NMR (100 MHz, $CDCl_3$) δ 165.48 (C=O), 138.36 (Ar), 137.26 (Ar), 133.70 (Ar), 133.24 (Ar), 129.96 (Ar), 129.90 (Ar), 129.86 (Ar), 129.27 (Ar), 128.66 (Ar), 128.63 (Ar), 128.18 (Ar), 128.16 (Ar), 91.87 (C-1), 83.45 (C-3), 83.40 (C-4), 81.96 (C-2), 72.76 ($PhCH_2$), 70.67 (C-5), 64.75 (C-6), 21.25 (tolyl CH_3); HRMS (ESI) Calcd for $C_{27}H_{28}O_6SNa$ $[M + Na]^+$: 503.1499, found: 503.1511.

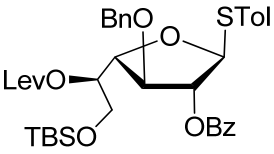

***p*-Tolyl 2-*O*-benzoyl-3-*O*-benzyl-5-*O*-levulinoyl-6-*O*-*tert*-butydimethylsilyl-1-thio-**

***β*-D-galactofuranoside (24e):** To a solution of **24d** (5.24 g, 10.9 mmol) and imidazole (1.86 g, 27.3 mmol) in anhydrous CH_2Cl_2 (25 mL) was added TBSCl (1.81 g, 12.0 mmol) at 0 °C, the mixture was stirred for 5 h at the same temperature. When TLC indicated the disappearance of **24d**, the reaction was quenched with CH_3OH (5 mL) and stirred for another 30 min. The solvent was removed under reduced pressure, and the residue was purified by column chromatography on silica gel (petroleum ether/acetone, 30:1) to afford a colorless syrup, which was dissolved in anhydrous CH_2Cl_2 (20 mL). To the resulting mixture was added EDC·HCl (3.13 g, 17.35 mmol) and DMAP (133 mg, 1.09 mmol), followed by the addition of Levulinoyl acid (1.34 mL, 13.1 mmol) at 0 °C. After stirring for 36 h at room temperature, the reaction mixture was diluted with CH_2Cl_2 (50 mL) and washed with a saturated aqueous $NaHCO_3$ solution (30 mL) and brine (30 mL). The organic layer extract was dried over Na_2SO_4, filtered, and concentrated. The residue was purified by column chromatography on silica gel (petroleum ether/acetone, 20:1) to yield **24e** (6.05 g, 80% for two steps) as colorless syrup. R_f = 0.31 (petroleum ether/ethyl acetate, 4:1); $[\alpha]_D^{30}$ -81.1 (*c* 0.5, CH_2Cl_2); ^{1}H NMR (400 MHz, $CDCl_3$) δ 8.07 – 8.05 (m, 2H, Ar), 7.58 (t, *J* = 7.4 Hz, 1H, Ar), 7.46 – 7.42 (m, 4H, Ar), 7.36 – 7.22 (m, 5H, Ar), 7.10 (d, *J* = 8.0 Hz, 2H, Ar), 5.58 (s, 1H, H-1), 5.50 (t, *J* = 1.8 Hz, 1H, H-2), 5.24 – 5.20 (m, 1H, H-5), 4.78 (d, *J* = 11.8 Hz, 1H, $PhCH_2$), 4.63 – 4.59 (m, 2H, $PhCH_2$, H-4), 4.09 (dd, *J* = 6.2, 1.7 Hz, 1H, H-3), 3.77 – 3.69 (m, 2H, H-6a, H-6b), 2.69 – 2.56 (m, 2H, Lev CH_2), 2.54 – 2.41 (m, 2H, Lev CH_2), 2.32 (s, 3H, tolyl CH_3), 2.09 (s, 3H, Lev CH_3), 0.87 (s, 9H, *t*-Bu), 0.04 (s, 3H, CH_3), 0.04 (s, 3H, CH_3); ^{13}C NMR (100 MHz, $CDCl_3$) δ 206.14 (C=O), 172.27 (C=O), 165.50 (C=O), 137.87 (Ar), 137.51 (Ar), 133.60 (Ar), 132.91 (Ar), 130.33 (Ar), 129.98 (Ar), 129.74 (Ar), 129.47 (Ar), 128.60 (Ar), 128.53 (Ar), 128.18 (Ar), 127.95 (Ar), 91.20 (C-1), 82.99 (C-3), 82.38 (C-2), 80.22 (C-4), 72.79 ($PhCH_2$), 72.48 (C-5), 61.56 (C-6), 38.04 (Lev CH_2), 29.83 (Lev CH_3), 28.11 (Lev CH_2), 25.91 (*t*-Bu), 21.25 (tolyl CH_3), 18.32 (*t*-Bu), -5.31 (CH_3); HRMS (ESI) Calcd for $C_{38}H_{48}O_8SSiNa$ $[M + Na]^+$: 715.2731, found: 715.2743.

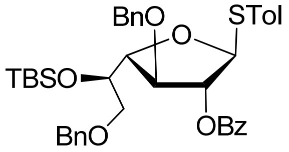

***p*-Tolyl 2-*O*-benzoyl-3,6-di-*O*-benzyl-5-*O*-*tert*-butydimethylsilyl-1-thio-*β*-D-galactofuranoside (24f):** To a solution of **19** (1.83 g, 3.21 mmol) and imidazole (0.87 g, 12.84 mmol) in anhydrous CH_2Cl_2 (10 mL) was added TBSCl (0.97 g, 6.42 mmol) at 0 °C, the reaction mixture was refluxed for 5 h, then the reaction was quenched with CH_3OH (0.5 mL) and stirred for another 30 min at room temperature. The solvent was removed under reduced pressure, and the residue was purified by column chromatography on silica gel (petroleum ether/acetone, 100:1) to give **24f** (2.11 g, 96%) as colorless syrup. R_f = 0.55 (petroleum ether/ethyl acetate, 8:1); $[\alpha]_D^{30}$ -105.7 (*c* 0.7, CH_2Cl_2); ^{1}H NMR (400 MHz, $CDCl_3$) δ 8.03 – 8.00 (m, 2H, Ar), 7.58 (t, *J* = 7.4 Hz, 1H, Ar), 7.45 – 7.41 (m, 4H, Ar), 7.35 – 7.25 (m, 10H, Ar), 7.06 (d, *J* = 8.0 Hz, 2H, Ar), 5.60 (s, 1H, H-1), 5.52 (t, *J* = 1.8 Hz, 1H, H-2), 4.80 (d, *J* = 11.9 Hz, 1H, $PhCH_2$), 4.59 (d, *J* = 11.9 Hz, 1H, $PhCH_2$), 4.51 (dd, *J* = 5.7, 3.8 Hz, 1H, H-4), 4.46 (t, *J* = 12.4 Hz, 2H, $PhCH_2$), 4.24 (d, *J* = 5.1 Hz, 1H, H-3), 4.08 – 4.04 (m, 1H, H-5), 3.57 (dd, *J* = 9.6, 5.5 Hz, 1H, H-6a), 3.49 (dd, *J* = 9.5, 6.3 Hz, 1H, H-6b), 2.31 (s, 3H, tolyl CH_3), 0.81 (s, 9H, *t*-Bu), 0.02 (s, 3H, CH_3), -0.05 (s, 3H, CH_3); ^{13}C NMR (100 MHz, $CDCl_3$) δ 165.54 (C=O), 138.30 (Ar), 137.78 (Ar), 137.74 (Ar), 133.53 (Ar), 133.00 (Ar), 130.51 (Ar), 129.98 (Ar), 129.73 (Ar), 129.59 (Ar), 128.56 (Ar), 128.45 (Ar), 128.11 (Ar), 127.95 (Ar), 127.70 (Ar), 127.66 (Ar), 91.25 (C-1), 83.53 (C-4), 82.93 (C-3), 82.51 (C-2), 73.54 ($PhCH_2$), 72.35 ($PhCH_2$), 72.30 (C-6), 71.04 (C-5), 26.02 (*t*-Bu), 21.25 (tolyl CH_3), 18.33 (*t*-Bu), -4.14 (CH_3), -4.85 (CH_3); HRMS (ESI) Calcd for $C_{40}H_{48}O_6SSiNa$ $[M + Na]^+$: 707.2833, found: 707.2823.

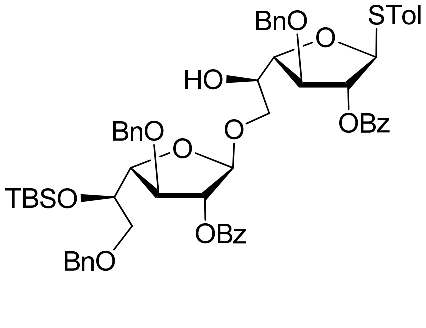

***p*-Tolyl 2-*O*-benzoyl-3,6-di-*O*-benzyl-5-*O*-*tert*-butydimethylsilyl-*β*-D-galactofuranosyl-(1→6)-2-*O*-benzoyl-3-*O*-benzyl-1-thio-*β*-D-galactofuranoside (24g):** A mixture of **24f** (68.5 mg, 0.10 mmol), TTBP (29.8 mg, 0.12 mmol), and freshly activated 4 Å molecular sieves (2.5 g) in CH_2Cl_2 (18 mL) under argon atmosphere was stirred for 20 min at room temperature, and cooled to -78 °C using acetone-anhydrous ice bath. 5 min later, stoichiometric *p*-TolSCl (13.8 μL, 0.10 mmol) was added without touching the wall of flask, followed by dropwise addition of AgOTf (0.5 mL, 0.20 mmol, 0.4 mol/L in anhydrous toluene/CH_2Cl_2, 3:1). After another 5 min, the acceptor **24d** (48.1 mg, 0.10 mmol) in anhydrous CH_2Cl_2 (1 mL) was added dropwise. 5 min later, the reaction solution was quenched with Et_3N (0.1 mL), warmed to room temperature, and filtered through Celite. The filtrate was concentrated to give a crude residue, which was purified by column chromatography on silica gel (petroleum ether/acetone, 10:1) to afford **24g** (57.0 mg, 55%) as white foam. R_f = 0.46 (petroleum ether/ethyl acetate, 3:1); $[\alpha]_D^{30}$ -91.3 (*c* 0.3, CH_2Cl_2); ^1H NMR (400 MHz, $CDCl_3$) δ 8.03 – 8.00 (m, 4H, Ar), 7.61 – 7.54 (m, 2H, Ar), 7.46 – 7.20 (m, 21H, Ar), 7.09 (d, *J* = 8.0 Hz, 2H, Ar), 5.59 (s, 1H, H-1), 5.53 (t, *J* = 1.7 Hz, 1H, H-2), 5.37 (d, *J* = 1.4 Hz, 1H, H-2′), 5.18 (s, 1H, H-1′), 4.78 (t, *J* = 12.2 Hz, 2H, $PhCH_2$), 4.61 (d, *J* = 11.9 Hz, 1H, $PhCH_2$), 4.57 (d, *J* = 11.9 Hz, 1H, $PhCH_2$), 4.43 – 4.40 (m, 3H, $PhCH_2$, H-4), 4.32 (d, *J* = 5.6 Hz, 1H, H-3), 4.24 (dd, *J* = 6.1, 3.8 Hz, 1H, H-4′), 4.18 (d, *J* = 5.9 Hz, 1H, H-3′), 4.09 – 4.00 (m, 2H, H-5, H-5′), 3.81 (dd, *J* = 10.6, 4.0 Hz, 1H, H-6a), 3.63 – 3.54 (m, 2H, H-6b, H-6′a), 3.48 (dd, *J* = 9.6, 6.5 Hz, 1H, H-6′b), 2.62 (d, *J* = 5.8 Hz, 1H, -OH), 2.29 (s, 3H, tolyl CH_3), 0.82 (s, 9H, *t*-Bu), 0.04 (s, 3H, CH_3), -0.02 (s, 3H, CH_3); ^{13}C NMR (100 MHz, $CDCl_3$) δ 165.75 (C=O), 165.56 (C=O), 138.26 (Ar), 137.84 (Ar), 137.82 (Ar), 137.66 (Ar), 133.54 (Ar), 132.80 (Ar), 130.58 (Ar), 130.01 (Ar), 129.98 (Ar), 129.87 (Ar), 129.61 (Ar), 129.56 (Ar), 128.63 (Ar), 128.58 (Ar), 128.57 (Ar), 128.54 (Ar), 128.46 (Ar), 128.13 (Ar), 128.09 (Ar), 128.00 (Ar), 127.91 (Ar), 127.72 (Ar), 127.70 (Ar), 106.81 (C-1′), 91.85 (C-1), 83.73 (C-4′), 83.41 (C-3), 82.82 (C-3′), 82.70 (C-2′), 82.45 (C-4), 82.20 (C-2), 73.53 ($PhCH_2$), 72.73 ($PhCH_2$), 72.43, 72.40, 71.00 (C-5′), 69.60 (C-6), 69.54 (C-5), 26.06 (*t*-Bu), 21.24 (tolyl CH_3), 18.39 (*t*-Bu), -4.11 (CH_3), -4.76 (CH_3); HRMS (ESI)

Calcd for $C_{60}H_{72}O_{12}NSSi$ $[M + NH_4]^+$: 1058.4539, found: 1058.4551.

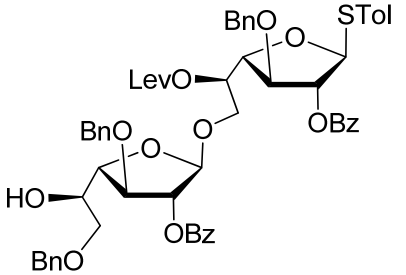

***p*-Tolyl 2-*O*-benzoyl-3,6-di-*O*-benzyl-*β*-D-galactofuranosyl-(1→6)-2-*O*-benzoyl-3-*O*-benzyl-5-*O*-levulinoyl-1-thio-*β*-D-galactofuranoside (24b):** To a solution of **24g** (210 mg, 0.202 mmol) in anhydrous CH_2Cl_2 (5 mL) was added EDC·HCl (96.8 g, 0.505 mmol) and DMAP (2.4 mg, 0.02 mmol), followed by the addition of Levulinoyl acid (41.5 mL, 0.404 mmol) at 0 °C. After stirring for 24 h at 35 °C, the reaction mixture was diluted with CH_2Cl_2 (15 mL) and washed with a saturated aqueous $NaHCO_3$ solution (10 mL) and brine (10 mL). The organic layer extract was dried over Na_2SO_4, filtered, and concentrated. The residue was purified by column chromatography on silica gel (petroleum ether/acetone, 10:1) to give a white foam, which was dissolved in THF (5 mL) and H_2O (0.5 mL). To the resulting solution was added 70% HF-pyridine (1.0 mL) at 0 °C, the reaction solution was warmed to 35 °C and stirred overnight. Then the reaction mixture was quenched with a saturated aqueous $NaHCO_3$ solution (40 mL), and extracted with EtOAc (2 × 40 mL). The combined organic layer, after being washed with 0.1 N HCl (2 × 30 mL), saturated aqueous $NaHCO_3$ solution (30 mL) and brine (30 mL), was dried over Na_2SO_4, filtered, and concentrated. The residue was purified by column chromatography on silica gel (petroleum ether/ethyl acetate, 2:1) to afford **24b** (153 mg, 74% for two steps) as white foam. R_f = 0.59 (petroleum ether/ethyl acetate, 1:1); $[\alpha]_D^{30}$ -80.2 (*c* 0.9, CH_2Cl_2); ^1H NMR (400 MHz, $CDCl_3$) δ 8.07 – 8.05 (m, 2H, Ar), 8.00 – 7.98 (m, 2H, Ar), 7.60 – 7.54 (m, 2H, Ar), 7.46 – 7.40 (m, 6H, Ar), 7.36 – 7.20 (m, 15H, Ar), 7.08 (d, *J* = 8.0 Hz, 2H, Ar), 5.58 (s, 1H, H-1), 5.50 (t, *J* = 1.9 Hz, 1H, H-2), 5.48 – 5.44 (m, 1H, H-5), 5.33 (d, *J* = 1.3 Hz, 1H, H-2′), 5.13 (s, 1H, H-1′), 4.79 – 4.74 (m, 2H, $PhCH_2$), 4.61 – 4.57 (m, 2H, $PhCH_2$, H-3), 4.54 (d, *J* = 12.0 Hz, 1H, $PhCH_2$), 4.50 (d,

J = 11.9 Hz, 1H, PhCH$_2$), 4.46 (d, J = 11.9 Hz, 1H, PhCH$_2$), 4.18 (dd, J = 5.8, 3.7 Hz, 1H, H-4′), 4.15 – 4.11 (m, 2H, H-3′, H-4), 3.94 – 3.87 (m, 2H, H-5′, H-6a), 3.67 (dd, J = 10.8, 7.4 Hz, 1H, H-6b), 3.55 – 3.47 (m, 2H, H-6′a, H-6′b), 2.67 – 2.42 (m, 4H, Lev CH$_2$CH$_2$), 2.38 (d, J = 6.1 Hz, 1H, -OH), 2.29 (s, 3H, tolyl CH$_3$), 2.05 (s, 3H, Lev CH$_3$); ^{13}C NMR (100 MHz, CDCl$_3$) δ 206.23 (C=O), 172.35 (C=O), 165.50 (C=O), 138.06 (Ar), 137.88 (Ar), 137.71 (Ar), 137.54 (Ar), 133.60 (Ar), 133.57 (Ar), 132.81 (Ar), 130.40 (Ar), 130.02 (Ar), 129.92 (Ar), 129.86 (Ar), 129.55 (Ar), 129.50 (Ar), 128.63 (Ar), 128.58 (Ar), 128.53 (Ar), 128.26 (Ar), 128.12 (Ar), 127.96 (Ar), 127.93 (Ar), 127.89 (Ar), 127.86 (Ar), 106.91 (C-1′), 91.43 (C-1), 83.51 (C-3′), 83.19 (C-4), 82.91 (C-4′), 82.27 (C-2), 81.78 (C-2′), 80.79 (C-3), 73.56 (PhCH$_2$), 72.93 (PhCH$_2$), 72.51 (PhCH$_2$), 71.85 (C-6′), 70.76 (C-5), 70.21 (C-5′), 66.50 (C-6), 38.09 (Lev CH$_2$), 29.77 (Lev CH$_3$), 28.18 (Lev CH$_2$), 21.25 (tolyl CH$_3$); HRMS (ESI) Calcd for $C_{59}H_{64}O_{14}NS$ [M + NH$_4$]$^+$: 1042.4042, found: 1042.4052.

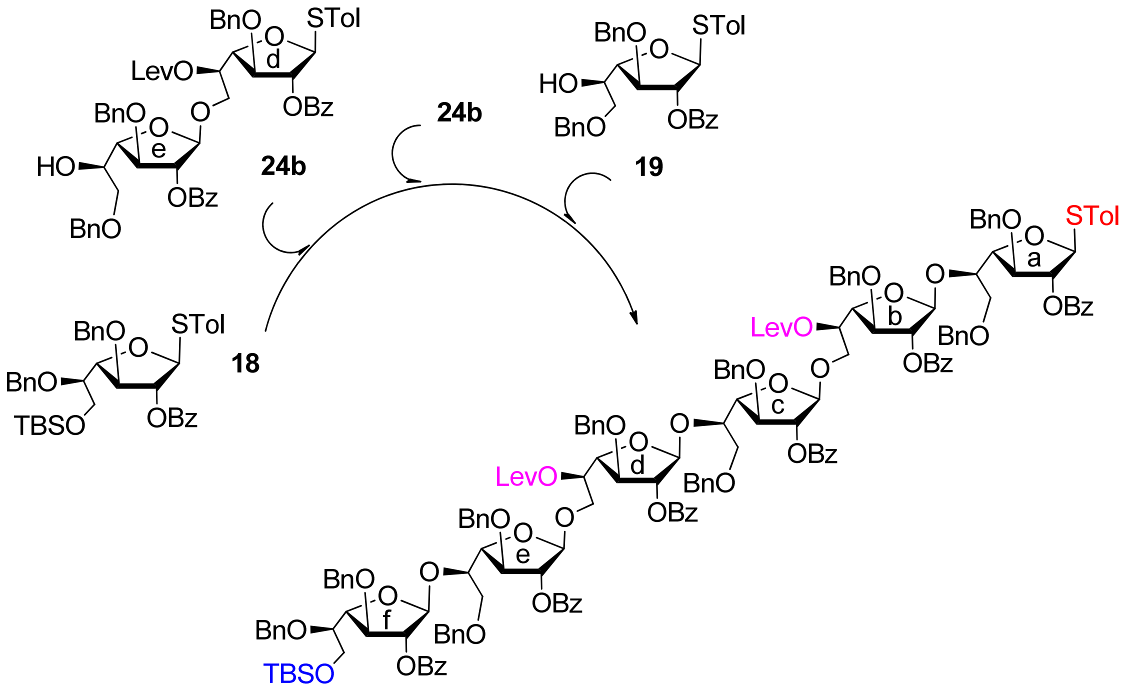

***p*-Tolyl 2-*O*-benzoyl-3,5-di-*O*-benzyl-6-*O*-*tert*-butydimethylsilyl-*β*-D-galactofuranosyl^{f}-(1→5)-2-*O*-benzoyl-3,6-di-*O*-benzyl-*β*-D-galactofuranosyl^{e}-(1→6)-2-*O*-benzoyl-3-*O*-benzyl-5-*O*-levulinoyl-*β*-D-galactofuranosyl^{d}-(1→5)-2-*O*-benz**

oyl-3,6-di-*O*-benzyl-*β*-D-galactofuranosyl[c]-(1→6)-2-*O*-benzoyl-3-*O*-benzyl-5-*O*-levulinoyl-*β*-D-galactofuranosyl[b]-(1→5)-2-*O*-benzoyl-3,6-di-*O*-benzyl-1-thio-*β*-D-galactofuranoside[a] (9): A mixture of **18** (150.2 mg, 219.3 μmol), TTBP (184 mg, 739.4 μmol) and freshly activated 4 Å molecular sieves (2.5 g) in anhydrous CH_2Cl_2 (15 mL) under argon atmosphere was stirred for 20 min at room temperature, and cooled to -78 °C. 5 min later, stoichiometric *p*-TolSCl (30.3 μL, 219.3 μmol) was added, followed by dropwise addition of AgOTf (1.1 mL, 438.6 μmol, 0.4 mol/L in anhydrous toluene/CH_2Cl_2, 3:1). After another 5 min, a solution of **24b** (213.6 mg, 208.3 μmol) in anhydrous CH_2Cl_2 (2 mL) was slowly added. The resulting mixture was slowly warmed to room temperature within 2 h and cooled back to -78 °C. 5 min later, stoichiometric *p*-TolSCl (28.7 μL, 208.3 μmol) was added, followed by dropwise addition of AgOTf (1 mL, 416.7 μmol, 0.4 mol/L in anhydrous toluene/CH_2Cl_2, 3:1). After another 5 min, a solution of **24b** (193.3 mg, 188.6 μmol) in anhydrous CH_2Cl_2 (2 mL) was slowly added. The resulting mixture was slowly warmed to room temperature within 2 h and cooled back to -78 °C. 5 min later, stoichiometric *p*-TolSCl (26.0 μL, 188.6 μmol) was added, followed by dropwise addition of AgOTf (0.9 mL, 377.2 μmol, 0.4 mol/L in anhydrous toluene/CH_2Cl_2, 3:1). After another 5 min, a solution of **19** (125.1 mg, 219.3 μmol) in anhydrous CH_2Cl_2 (2 mL) was slowly added. The resulting mixture was slowly warmed to room temperature within 2 h, quenched with Et_3N (300 μL) and filtered through Celite. After removal of the solvent, the desired product was purified by column chromatography on silica gel (petroleum ether/acetone, 3.5:1) to afford **9** (437.4 mg, 79%) as white foam. R_f = 0.30 (petroleum ether/ethyl acetate, 2:1); $[\alpha]_D^{30}$ -63.4 (*c* 0.4, CH_2Cl_2); ^1H NMR (600 MHz, $CDCl_3$) δ 8.03 – 7.91 (m, 12H, Ar), 7.58 – 7.52 (m, 3H, Ar), 7.49 – 7.46 (m, 1H, Ar), 7.44 – 7.37 (m, 10H, Ar), 7.32 – 7.05 (m, 56H, Ar), 7.02 (d, *J* = 8.0 Hz, 2H, Ar), 5.63 (s, 1H, H-1[f]), 5.57 (br s, 2H, H-1[a,d]), 5.491 – 5.485 (m, 4H, H-2[a,d,f], H-1[b]), 5.44 (d, *J* = 1.7 Hz, 1H, H-2[b]), 5.30 – 5.26 (m, 2H, H-5[b,d]), 5.22 (d, *J* = 1.7 Hz, 1H, H-2[e]), 5.21 (d, *J* = 2.0 Hz, 1H, H-2[c]), 4.96 (s, 1H, H-1[e]), 4.95 (s, 1H, H-1[c]), 4.73 (d, *J* = 11.5 Hz, 1H, PhCH2), 4.68 – 4.62 (m, 6H, PhCH2), 4.56 (d, *J* = 11.6 Hz, 1H, PhCH2), 4.53 – 4.47 (m, 5H, PhCH2), 4.44 (t, *J* = 12.3 Hz, 2H, PhCH2),

4.38 – 4.23 (m, 12H, $PhCH_2$, H-3[a,c,e], H-4[b,d,f]), 4.20 – 4.13 (m, 5H, H-4[c,e], H-5[a,c,e]), 4.00 – 3.99 (m, 3H, H-3[b,d,f]), 3.78 – 3.61 (m, 10H, H-6[a,b,c,d,e,f]), 3.55 – 3.51 (m, 2H, H-6[b,d]), 3.49 – 3.46 (m, 1H, H-5[f]), 2.58 – 2.31 (m, 8H, Lev CH_2), 2.25 (s, 3H, tolyl CH_3), 1.96 (s, 3H, Lev CH_3), 1.93 (s, 3H, Lev CH_3), 0.79 (s, 9H, *t*-Bu), -0.09 (s, 3H, CH_3), -0.11 (s, 3H, CH_3); ^{13}C NMR (150 MHz, $CDCl_3$) δ 206.23 (Lev C=O), 206.15 (Lev C=O), 172.32 (Lev C=O), 172.22 (Lev C=O), 165.51 (C=O), 165.50 (C=O), 165.49 (C=O), 165.39 (C=O), 165.37 (C=O), 165.28 (C=O), 138.54 (Ar), 138.21 (Ar), 138.20 (Ar), 138.17 (Ar), 138.02 (Ar), 138.00 (Ar), 137.93 (Ar), 137.82 (Ar), 137.73 (Ar), 133.52 (Ar), 133.44 (Ar), 133.42 (Ar), 133.38 (Ar), 133.34 (Ar), 133.31 (Ar), 132.88 (Ar), 130.38 (Ar), 129.98 (Ar), 129.95 (Ar), 129.87 (Ar), 129.83 (Ar), 129.70 (Ar), 129.62 (Ar), 129.61 (Ar), 129.55 (Ar), 129.46 (Ar), 129.36 (Ar), 128.62 (Ar), 128.58 (Ar), 128.55 (Ar), 128.52 (Ar), 128.44 (Ar), 128.37 (Ar), 128.32 (Ar), 128.30 (Ar), 128.20 (Ar), 128.13 (Ar), 128.01 (Ar), 127.93 (Ar), 127.90 (Ar), 127.79 (Ar), 127.76 (Ar), 127.73 (Ar), 127.69 (Ar), 127.57 (Ar), 127.55 (Ar), 127.54 (Ar), 127.46 (Ar), 127.44 (Ar), 127.38 (Ar), 107.17 (C-1[c]), 107.07 (C-1[e]), 106.82 (C-1[f]), 106.55 (C-1[d]), 106.24 (C-1[b]), 91.26 (C-1[a]), 83.83, 83.69, 83.62, 83.58, 83.44, 83.31, 82.46, 82.42, 82.24, 82.19, 82.03, 82.00, 81.93, 81.88, 81.50, 78.92 (C-5[f]), 74.65 (C-5[a]), 74.10 ($PhCH_2$), 73.99 (C-5[e]), 73.91 (C-5[c]), 73.54 ($PhCH_2$), 73.35 (×2, $PhCH_2$), 72.86 ($PhCH_2$), 72.82 ($PhCH_2$), 72.74 ($PhCH_2$), 72.69 ($PhCH_2$), 72.64 ($PhCH_2$), 72.09 ($PhCH_2$), 71.85 (C-6), 71.72 (C-6), 71.33 (C-5[d]), 71.10 (C-5[b]), 70.84 (C-6[a]), 67.26 (C-6[b]), 66.93 (C-6[d]), 64.80 (C-6[f]), 38.08 (Lev CH_2), 38.04 (Lev CH_2), 29.68 (Lev CH_3), 29.63 (Lev CH_3), 28.15 (Lev CH_2), 28.12 (Lev CH_2), 25.94 (*t*-Bu), 21.21 (tolyl CH_3), 18.21 (*t*-Bu), -5.36 (×2, CH_3); HRMS (ESI) Calcd for $C_{171}H_{186}O_{40}N_2SSi$ $[M + 2NH_4]^{2+}$: 1483.6030, found: 1483.6063.

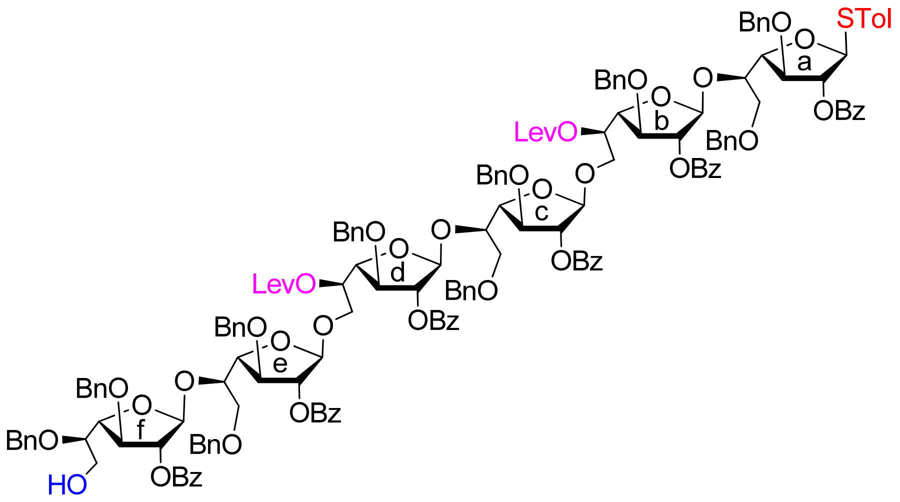

***p*-Tolyl 2-*O*-benzoyl-3,5-di-*O*-benzyl-*β*-D-galactofuranosyl[f]-(1→5)-2-*O*-benzoyl-3,6-di-*O*-benzyl-*β*-D-galactofuranosyl[e]-(1→6)-2-*O*-benzoyl-3-*O*-benzyl-5-*O*-levulinoyl-*β*-D-galactofuranosyl[d]-(1→5)-2-*O*-benzoyl-3,6-di-*O*-benzyl-*β*-D-galactofuranosyl[c]-(1→6)-2-*O*-benzoyl-3-*O*-benzyl-5-*O*-levulinoyl-*β*-D-galactofuranosyl[b]-(1→5)-2-*O*-benzoyl-3,6-di-*O*-benzyl-1-thio-*β*-D-galactofuranoside[a] (7):** To a solution of **9** (480 mg, 0.164 mmol) in THF (5 mL) and H_2O (0.5 mL) was added 70% HF-pyridine (0.5 mL) at 0 °C, the resulting mixture was warmed to 35 °C and stirred for 4 h. Then the reaction solution was quenched with a saturated aqueous $NaHCO_3$ solution (20 mL), and extracted with EtOAc (2 × 30 mL). The combined organic layer, after being washed with 0.1 N HCl (2 × 20 mL), saturated aqueous $NaHCO_3$ solution (30 mL) and brine (30 mL), was dried over Na_2SO_4, filtered, and concentrated. The residue was purified by column chromatography on silica gel (petroleum ether/ethyl acetate, 2:1) to afford **7** (417 mg, 90%) as white foam. R_f = 0.46 (petroleum ether/acetone, 1.5:1); $[\alpha]_D^{30}$ -76.7 (*c* 0.4, CH_2Cl_2); ^{1}H NMR (600 MHz, $CDCl_3$) δ 8.03 – 7.92 (m, 12H, Ar), 7.58 – 7.56 (m, 1H, Ar), 7.55 – 7.49 (m, 3H, Ar), 7.44 – 7.38 (m, 10H, Ar), 7.35 – 7.06 (m, 56H, Ar), 7.02 (d, *J* = 8.0 Hz, 2H, Ar), 5.574 (br s, 2H, H-1^{a,d}), 5.566 (s, 1H, H-1^{f}), 5.49 – 5.48 (m, 4H, H-2^{a,d,f}, H-1^{b}), 5.44 (d, *J* = 1.7 Hz, 1H, H-2^{b}), 5.30 – 5.27 (m, 2H, H-5^{b,d}), 5.21 (d, *J* = 1.9 Hz, 1H, H-2^{c}), 5.20 (s, 1H, H-2^{e}), 4.964 (s, 1H, H-1^{e}), 4.956 (s, 1H, H-1^{c}), 4.73 (d, *J* = 11.6 Hz, 1H, $PhCH_2$), 4.68 – 4.47 (m, 12H, $PhCH_2$, H-4^{a}), 4.44 (t, *J* = 12.9 Hz, 1H, $PhCH_2$), 4.39 – 4.28 (m, 10H, $PhCH_2$, H-3^{a,c}, H-4^{b,f}), 4.25 – 4.14 (m, 7H, H-3^{e}, H-4^{d,c,e}, H-5^{a,c,e}), 4.02 – 3.99 (m, 3H, H-3^{b,d,f}), 3.74 – 3.61 (m, 9H, H-6^{a,b,c,d,e,f}), 3.58 – 3.53 (m, 3H, H-6^{b,d,f}), 3.47 – 3.45 (m, 1H, H-5^{f}),

2.57 – 2.31 (m, 8H, Lev CH_2), 2.25 (s, 3H, tolyl CH_3), 2.20 (t, *J* = 6.3 Hz, 1H, -OH), 1.96 (s, 3H, Lev CH_3), 1.93 (s, 3H, Lev CH_3); ^{13}C NMR (150 MHz, $CDCl_3$) δ 206.22 (Lev C=O), 206.17 (Lev C=O), 172.40 (Lev C=O), 172.32 (Lev C=O), 165.52 (×2, C=O), 165.42 (C=O), 165.38 (C=O), 165.37 (C=O), 165.28 (C=O), 138.19 (Ar), 138.16 (Ar), 138.13 (Ar), 138.05 (Ar), 137.97 (Ar), 137.82 (Ar), 137.73 (Ar), 137.72 (Ar), 137.61 (Ar), 133.52 (Ar), 133.41 (Ar), 132.88 (Ar), 130.37 (Ar), 129.97 (Ar), 129.95 (Ar), 129.94 (Ar), 129.90 (Ar), 129.89 (Ar), 129.82 (Ar), 129.63 (Ar), 129.61 (Ar), 129.53 (Ar), 129.45 (Ar), 129.36 (Ar), 128.62 (Ar), 128.57 (Ar), 128.51 (Ar), 128.43 (Ar), 128.41 (Ar), 128.38 (Ar), 128.32 (Ar), 128.20 (Ar), 128.16 (Ar), 128.03 (Ar), 128.00 (Ar), 127.97 (Ar), 127.93 (Ar), 127.90 (Ar), 127.88 (Ar), 127.77 (Ar), 127.76 (Ar), 127.74 (Ar), 127.72 (Ar), 127.69 (Ar), 127.56 (Ar), 127.49 (Ar), 127.46 (Ar), 127.44 (Ar), 127.42 (Ar), 107.17 (C-1^c), 106.81 (C-1^e), 106.72 (C-1^f), 106.59 (C-1^d), 106.24 (C-1^b), 91.25 (C-1^a), 83.68, 83.62, 83.61, 83.58, 83.41, 83.00, 82.90, 82.57, 82.41, 82.26, 82.24, 82.18, 82.02, 82.00, 81.98, 81.93, 81.82, 81.48, 77.60 (C-5^f), 74.65 (C-5^a), 74.61 (C-5^e), 74.03 (C-5^c), 73.53 ($PhCH_2$), 73.39 ($PhCH_2$), 73.36 ($PhCH_2$), 72.92 ($PhCH_2$), 72.82 ($PhCH_2$), 72.73 ($PhCH_2$), 72.68 ($PhCH_2$), 72.66 ($PhCH_2$), 72.41 ($PhCH_2$), 72.22 ($PhCH_2$), 71.86 ($PhCH_2$), 71.58 ($PhCH_2$), 71.37 (C-5^d), 71.09 (C-5^b), 70.84 (C-6^a), 67.25 (C-6^b), 66.94 (C-6^d), 62.01 (C-6^f), 38.07 (Lev CH_2), 38.01 (Lev CH_2), 29.67 (Lev CH_3), 29.62 (Lev CH_3), 28.15 (Lev CH_2), 28.12 (Lev CH_2), 21.20 (tolyl CH_3); HRMS (ESI) Calcd for $C_{165}H_{172}O_{40}N_2S$ $[M + 2NH_4]^{2+}$: 1426.5598, found: 1426.5626.

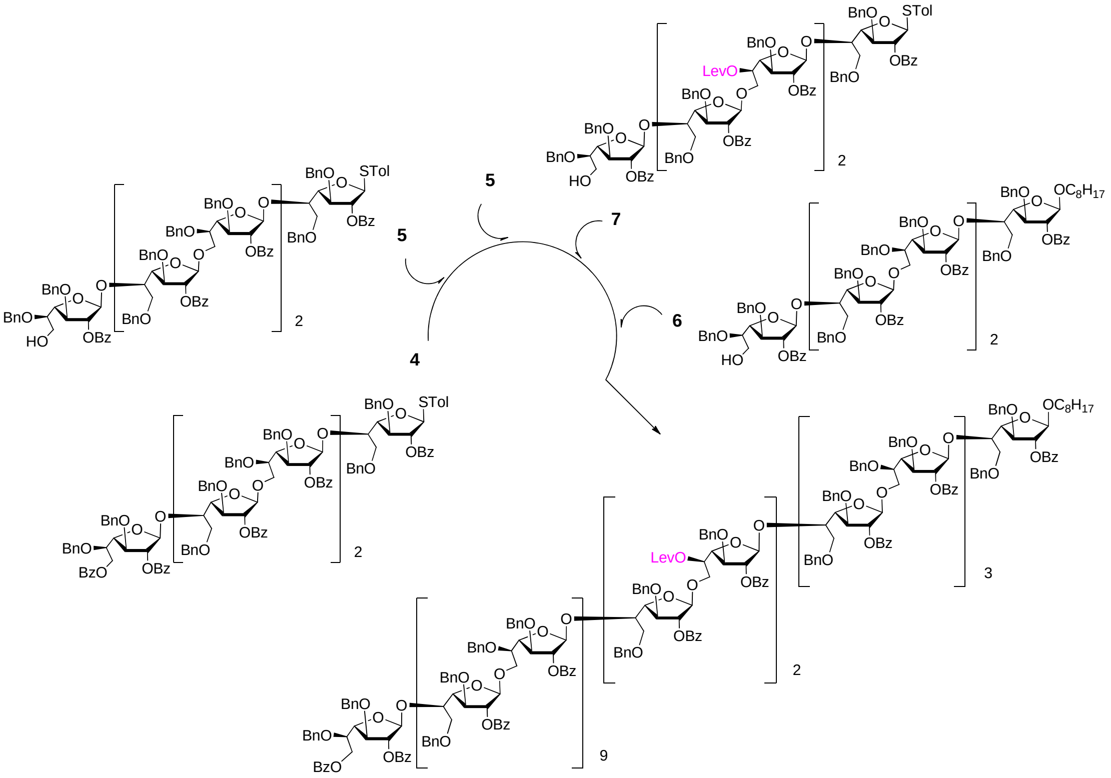

Octyl 2,6-di-*O*-benzoyl-3,5-di-*O*-benzyl-*β*-D-galactofuranosyl[d]-(1→5)-2-*O*-benzoyl-3,6-di-*O*-benzyl-*β*-D-galactofuranosyl[c14]-(1→6)-2-*O*-benzoyl-3,5-di-*O*-benzyl-*β*-D-galactofuranosyl[b14]-(1→5)-2-*O*-benzoyl-3,6-di-*O*-benzyl-*β*-D-galactofuranosyl[c13]-(1→6)-2-*O*-benzoyl-3,5-di-*O*-benzyl-*β*-D-galactofuranosyl[b13]-(1→5)-2-*O*-benzoyl-3,6-di-*O*-benzyl-*β*-D-galactofuranosyl[c12]-(1→6)-2-*O*-benzoyl-3,5-di-*O*-benzyl-*β*-D-galactofuranosyl[b12]-(1→5)-2-*O*-benzoyl-3,6-di-*O*-benzyl-*β*-D-galactofuranosyl[c11]-(1→6)-2-*O*-benzoyl-3,5-di-*O*-benzyl-*β*-D-galactofuranosyl[b11]-(1→5)-2-*O*-benzoyl-3,6-di-*O*-benzyl-*β*-D-galactofuranosyl[c10]-(1→6)-2-*O*-benzoyl-3,5-di-*O*-benzyl-*β*-D-galactofuranosyl[b10]-(1→5)-2-*O*-benzoyl-3,6-di-*O*-benzyl-*β*-D-galactofuranosyl[c9]-(1→6)-2-*O*-benzoyl-3,5-di-*O*-benzyl-*β*-D-galactofuranosyl[b9]-(1→5)-2-*O*-benzoyl-3,6-di-*O*-benzyl-*β*-D-galactofuranosyl[c8]-(1→6)-2-*O*-benzoyl-3,5-di-*O*-benzyl-*β*-D-galactofuranosyl[b8]-(1→5)-2-*O*-benzoyl-3,6-di-*O*-benzyl-*β*-D-galactofuranosyl[c7]-(1→6)-2-*O*-benzoyl-3,5-di-*O*-benzyl-*β*-D-galactofuranosyl[b7]-(1→5)-2-*O*-benzoyl-3,6-di-*O*-benzyl-*β*-D-galactofuranosyl[c6]-(1→6)-2-*O*-benzoyl-3,5-di-*O*-benzyl-*β*-D-galactofuranosyl[b6]-(1→5)-2-*O*-benzoyl-3,6-di-*O*-benzyl-*β*-D-galactofuranosyl[c5]-(1→6)-2-*O*-benzoyl-3-*O*-benzyl-5-*O*-levulinoyl-*β*-D-galactofuranosyl[b5]-(1→5)-2-*O*-benzoyl-3,6-di-*O*-benzyl-*β*-D-galactofuranosyl[c4]-(1→6)-2-*O*-benzoyl-3-*O*-benzyl-5-*O*-le

vulinoyl-*β*-D-galactofuranosyl[b4]-(1→5)-2-*O*-benzoyl-3,6-di-*O*-benzyl-*β*-D-galactofuranosyl[c3]-(1→6)-2-*O*-benzoyl-3,5-di-*O*-benzyl-*β*-D-galactofuranosyl[b3]-(1→5)-2-*O*-benzoyl-3,6-di-*O*-benzyl-*β*-D-galactofuranosyl[c2]-(1→6)-2-*O*-benzoyl-3,5-di-*O*-benzyl-*β*-D-galactofuranosyl[b2]-(1→5)-2-*O*-benzoyl-3,6-di-*O*-benzyl-*β*-D-galactofuranosyl[c1]-(1→6)-2-*O*-benzoyl-3,5-di-*O*-benzyl-*β*-D-galactofuranosyl[b1]-(1→5)-2-*O*-benzoyl-3,6-di-*O*-benzyl-*β*-D-galactofuranoside[a] (25): A mixture of **4** (25.00 mg, 8.60 μmol), TTBP (8.8 mg, 35.3 μmol) and freshly activated 4 Å molecular sieves (400 mg) in anhydrous CH_2Cl_2 (1.5 mL) under argon atmosphere was stirred for 20 min at room temperature, and cooled to -78 °C. 5 min later, stoichiometric *p*-TolSCl (29.7 μL, 8.60 μmol, 4% in anhydrous toluene) was added, followed by dropwise addition of AgOTf (65 μL, 25.80 μmol, 0.4 mol/L in anhydrous toluene/CH_2Cl_2, 3:1). After another 5 min, a solution of **5** (21.70 mg, 7.74 μmol) in anhydrous CH_2Cl_2 (0.6 mL) was slowly added. The resulting mixture was slowly warmed to room temperature within 2 h and cooled back to -78 °C. 5 min later, stoichiometric *p*-TolSCl (26.7 μL, 7.74 μmol, 4% in anhydrous toluene) was added, followed by dropwise addition of AgOTf (39 μL, 15.5 μmol, 0.4 mol/L in anhydrous toluene/CH_2Cl_2, 3:1). After another 5 min, a solution of **5** (19.29 mg, 6.88 μmol) in anhydrous CH_2Cl_2 (0.6 mL) was slowly added. The resulting mixture was slowly warmed to room temperature within 2 h and cooled back to -78 °C. 5 min later, stoichiometric *p*-TolSCl (23.7 μL, 6.88 μmol, 4% in anhydrous toluene) was added, followed by dropwise addition of AgOTf (35 μL, 13.8 μmol, 0.4 mol/L in anhydrous toluene/CH_2Cl_2, 3:1). After another 5 min, a solution of **7** (16.97 mg, 6.02 μmol) in anhydrous CH_2Cl_2 (0.6 mL) was slowly added. The resulting mixture was slowly warmed to room temperature within 2 h and cooled back to -78 °C. 5 min later, stoichiometric *p*-TolSCl (20.8 μL, 6.02 μmol, 4% in anhydrous toluene) was added, followed by dropwise addition of AgOTf (30 μL, 12.0 μmol, 0.4 mol/L in anhydrous toluene/CH_2Cl_2, 3:1). After another 5 min, a solution of **6** (24.16 mg, 8.60 μmol) in anhydrous CH_2Cl_2 (0.6 mL) was slowly added. The resulting mixture was slowly warmed to room temperature within 2 h, quenched with Et_3N (50 μL) and filtered through Celite. After removal of the solvent, the desired product was purified by

column chromatography on silica gel (petroleum ether/acetone, 1.8:1) to give a crude product, which was further purified by size exclusion chromatography (Bio-Beads S-X1, toluene/ethyl acetate, 1:1) to afford **25** (52.3 mg, 64%) as white foam. R_f = 0.45 (petroleum ether/acetone, 1.3:1); $[\alpha]_D^{30}$ -58.5 (*c* 0.5, CH_2Cl_2); ^1H NMR (600 MHz, $CDCl_3$) δ 8.02 – 7.90 (m, 60H, Ar), 7.86 – 7.85 (m, 2H, Ar), 7.57 – 6.95 (m, 383H, Ar), 5.62 (s, 1H, H-1^{d}), 5.57 – 5.55 (m, 14H, H-1^{b1-b14}), 5.51 (d, *J* = 1.3 Hz, 1H, H-2^{d}), 5.48 (br s, 14H, H-2^{b1-b14}), 5.33 – 5.29 (m, 13H, H-2$^{a,c1-c3,c6-c14}$), 5.28 – 5.24 (m, 2H, H-5^{b4,b5}), 5.20 (d, *J* = 1.8 Hz, 1H, H-2^{c4}/H-2^{c5}), 5.19 (d, *J* = 1.6 Hz, 1H, H-2^{c4}/H-2^{c5}), 5.11 (s, 1H, H-1^{a}), 5.06 – 5.03 (m, 11H, H-1$^{c1-c3,c6-c14}$), 4.93 (br s, 2H, H-1^{c4,c5}), 4.73 – 4.13 (m, 178H, $PhCH_2$, H-3$^{a,c1-c14}$, H-4$^{a,b1-b14,c1-c14,d}$, H-5$^{a,c1-c14}$, H-6^{d}), 4.07 (d, *J* = 5.9 Hz, 1H, H-3^{d}), 4.02 – 3.99 (m, 14H, H-3^{b1-b14}), 3.85 – 3.56 (m, 70H, H-5$^{b1-b3,b6-b14,d}$, H-6$^{a,b1-b14,c1-c14}$, octyl OCH_2), 3.52 – 3.48 (m, 2H, H-6^{b4,b5}), 3.41 (dt, *J* = 9.7, 6.6 Hz, 1H, octyl OCH_2), 2.47 – 2.31 (m, 8H, Lev CH_2), 1.914 (s, 3H, Lev CH_3), 1.906 (s, 3H, Lev CH_3), 1.59 – 1.54 (m, 2H, octyl CH_2), 1.29 – 1.23 (m, 10H, octyl CH_2), 0.85 (t, *J* = 7.0 Hz, 3H, octyl CH_3); ^{13}C NMR (150 MHz, $CDCl_3$) δ 206.16 (×2, Lev C=O), 172.23 (×2, Lev C=O), 166.19 (C=O), 165.67 (C=O), 165.50 (×13, C=O), 165.45 (C=O), 165.41 (C=O), 165.33 (×13, C=O), 165.29 (C=O), 138.47 (Ar), 138.43 (Ar), 138.23 (Ar), 138.18 (Ar), 138.14 (Ar), 138.07 (Ar), 138.03 (Ar), 138.00 (Ar), 137.98 (Ar), 137.88 (Ar), 137.87 (Ar), 137.82 (Ar), 137.80 (Ar), 137.72 (Ar), 137.71 (Ar), 137.63 (Ar), 133.35 (Ar), 133.06 (Ar), 129.98 (Ar), 129.94 (Ar), 129.88 (Ar), 129.66 (Ar), 129.62 (Ar), 129.56 (Ar), 129.54 (Ar), 129.52 (Ar), 129.49 (Ar), 129.45 (Ar), 129.43 (Ar), 128.59 (Ar), 128.56 (Ar), 128.45 (Ar), 128.42 (Ar), 128.36 (Ar), 128.33 (Ar), 128.30 (Ar), 128.26 (Ar), 128.15 (Ar), 128.12 (Ar), 128.08 (Ar), 127.94 (Ar), 127.90 (Ar), 127.86 (Ar), 127.77 (Ar), 127.71 (Ar), 127.65 (Ar), 127.53 (Ar), 127.50 (Ar), 127.45 (Ar), 127.43 (Ar), 127.40 (Ar), 127.37 (Ar), 107.14 (C-1^{c4}/C-1^{c5}), 107.09 (C-1^{c4}/C-1^{c5}), 107.04 (×11, C-1$^{c1-c3,c3-c13}$), 106.92 (C-1^{c14}), 106.74 (C-1^{d}), 106.63 (×11, C-1$^{b2,b3,b6-b14}$), 106.59 (C-1^{b4}/C-1^{b5}), 106.56 (C-1^{b4}/C-1^{b5}), 106.24 (C-1^{b1}), 106.00 (C-1^{a}), 83.91, 83.85, 83.79, 83.70, 83.61, 83.54, 83.32, 83.25, 82.97, 82.77, 82.53, 82.48, 82.41, 82.34, 82.18, 82.14, 82.08, 81.98, 81.90, 75.91 (C-5^{d}), 74.23, 74.11, 73.99, 73.93, 73.89, 73.74, 74.57, 73.49, 73.44, 73.39, 73.37, 73.33, 72.90, 72.77,

72.71, 72.66, 72.63, 72.12, 72.07, 71.82, 71.73, 71.60, 71.56, 71.37, 71.34, 71.06, 69.84, 69.54, 69.47, 67.63 (octyl OCH_2), 67.00 (C-6^{b4}/C-6^{b5}), 66.95 (C-6^{b4}/C-6^{b5}), 64.93 (C-6^{d}), 38.03 (×2, Lev CH_2), 31.98 (octyl CH_2), 29.63 (×2, Lev CH_3), 29.58 (octyl CH_2), 29.55 (octyl $OCH_2\underline{C}H_2$), 29.39 (octyl CH_2), 28.11 (×2, Lev CH_2), 26.23 (octyl CH_2), 22.80 (octyl CH_2), 14.25 (octyl CH_3); MALDI-TOF MS Calcd for $C_{821}H_{802}O_{186}Na$ $[M + Na]^+$ *m/z*: 13668.0, found: 13668.5.

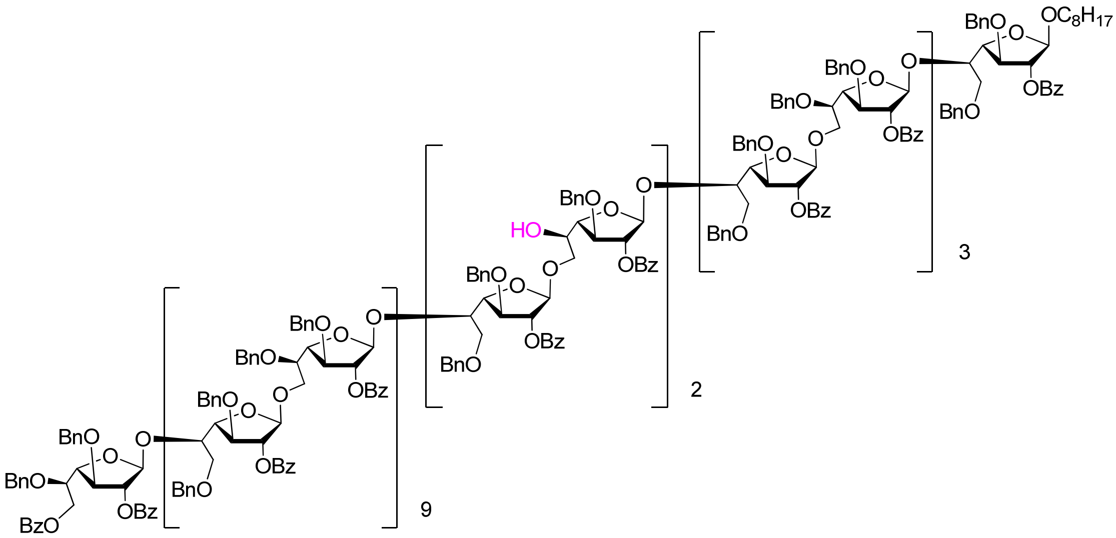

Octyl 2,6-di-*O*-benzoyl-3,5-di-*O*-benzyl-*β*-D-galactofuranosyl^{d}-(1→5)-2-*O*-benzoyl-3,6-di-*O*-benzyl-*β*-D-galactofuranosyl^{c14}-(1→6)-2-*O*-benzoyl-3,5-di-*O*-benzyl-*β*-D-galactofuranosyl^{b14}-(1→5)-2-*O*-benzoyl-3,6-di-*O*-benzyl-*β*-D-galactofuranosyl^{c13}-(1→6)-2-*O*-benzoyl-3,5-di-*O*-benzyl-*β*-D-galactofuranosyl^{b13}-(1→5)-2-*O*-benzoyl-3,6-di-*O*-benzyl-*β*-D-galactofuranosyl^{c12}-(1→6)-2-*O*-benzoyl-3,5-di-*O*-benzyl-*β*-D-galactofuranosyl^{b12}-(1→5)-2-*O*-benzoyl-3,6-di-*O*-benzyl-*β*-D-galactofuranosyl^{c11}-(1→6)-2-*O*-benzoyl-3,5-di-*O*-benzyl-*β*-D-galactofuranosyl^{b11}-(1→5)-2-*O*-benzoyl-3,6-di-*O*-benzyl-*β*-D-galactofuranosyl^{c10}-(1→6)-2-*O*-benzoyl-3,5-di-*O*-benzyl-*β*-D-galactofuranosyl^{b10}-(1→5)-2-*O*-benzoyl-3,6-di-*O*-benzyl-*β*-D-galactofuranosyl^{c9}-(1→6)-2-*O*-benzoyl-3,5-di-*O*-benzyl-*β*-D-galactofuranosyl^{b9}-(1→5)-2-*O*-benzoyl-3,6-di-*O*-benzyl-*β*-D-galactofuranosyl^{c8}-(1→6)-2-*O*-benzoyl-3,5-di-*O*-benzyl-*β*-D-galactofuranosyl^{b8}-(1→5)-2-*O*-benzoyl-3,6-di-*O*-benzyl-*β*-D-galactofuranosyl^{c7}-(1→6)-2-*O*-benzoyl-3,5-di-*O*-benzyl-*β*-D-galactofuranosyl^{b7}-(1→5)-2-*O*-benzoyl-3,6-di-*O*-benzyl-*β*-D-galactofuranosyl^{c6}-(1→6)-2-*O*-benzoyl-3,5-di-*O*-benzyl-*β*-D-gal

actofuranosyl^{b6}-(1→5)-2-*O*-benzoyl-3,6-di-*O*-benzyl-*β*-D-galactofuranosyl^{c5}-(1→6)-2-*O*-benzoyl-3-*O*-benzyl-*β*-D-galactofuranosyl^{b5}-(1→5)-2-*O*-benzoyl-3,6-di-*O*-benzyl-*β*-D-galactofuranosyl^{c4}-(1→6)-2-*O*-benzoyl-3-*O*-benzyl-*β*-D-galactofuranosyl^{b4}-(1→5)-2-*O*-benzoyl-3,6-di-*O*-benzyl-*β*-D-galactofuranosyl^{c3}-(1→6)-2-*O*-benzoyl-3,5-di-*O*-benzyl-*β*-D-galactofuranosyl^{b3}-(1→5)-2-*O*-benzoyl-3,6-di-*O*-benzyl-*β*-D-galactofuranosyl^{c2}-(1→6)-2-*O*-benzoyl-3,5-di-*O*-benzyl-*β*-D-galactofuranosyl^{b2}-(1→5)-2-*O*-benzoyl-3,6-di-*O*-benzyl-*β*-D-galactofuranosyl^{c1}-(1→6)-2-*O*-benzoyl-3,5-di-*O*-benzyl-*β*-D-galactofuranosyl^{b1}-(1→5)-2-*O*-benzoyl-3,6-di-*O*-benzyl-*β*-D-galactofuranoside^{a} (2): To a solution of **25** (260 mg, 19.06 μmol) in THF (2 mL) and CH_3OH (0.2 mL) was added hydrazine acetate (35.1 mg, 381.1 μmol), the resulting mixture was stirred at room temperature for 20 h, quenched with acetone (0.5 mL) and stirred for another 30 min. After removal of the solvent, the residue was dissolved in EtOAc (30 mL), washed with a saturated aqueous $NaHCO_3$ solution (10 mL) and brine (10 mL), dried over Na_2SO_4, filtered, and concentrated. The desired product was purified by column chromatography on silica gel (petroleum ether/acetone, 1.8:1) to afford **2** (218.4 mg, 85%) as white foam. R_f = 0.47 (petroleum ether/acetone, 1.3:1); $[\alpha]_D^{30}$ -64.7 (*c* 0.4, CH_2Cl_2); ^{1}H NMR (600 MHz, $CDCl_3$) δ 8.02 – 7.92 (m, 60H, Ar), 7.86 – 7.85 (m, 2H, Ar), 7.57 – 6.95 (m, 383H, Ar), 5.62 (s, 1H, H-1^{d}), 5.57 – 5.53 (m, 14H, H-1$^{b1\text{-}b14}$), 5.51 (br s, 2H, H-2^{d}, H-2^{b4}/H-2^{b5}), 5.50 (s, 1H, H-2^{b4}/H-2^{b5}), 5.48 – 5.47 (m, 12H, H-2$^{b1\text{-}b3,b6\text{-}b14}$), 5.33 – 5.30 (m, 13H, H-2$^{a,c1\text{-}c13,c6\text{-}c14}$), 5.24 (br s, 2H, H-2^{c4,c5}), 5.11 (s, 1H, H-1^{a}), 5.06 – 5.03 (m, 12H, H-1$^{c1\text{-}c3,c6\text{-}c14}$), 4.98 (s, 1H, H-1^{c4}/H-1^{c5}), 4.97 (s, 1H, H-1^{c4}/H-1^{c5}), 4.73 – 4.09 (m, 180H, $PhCH_2$, H-3$^{a,b4,b5,c1\text{-}c14}$, H-4$^{a,b1\text{-}b14,c1\text{-}c14,d}$, H-5$^{a,c1\text{-}c14}$, H-6^{d}), 4.07 (d, J = 5.9 Hz, 1H, H-3^{d}), 4.02 – 3.99 (m, 12H, H-3$^{b1\text{-}b3,b6\text{-}b14}$), 3.85 – 3.57 (m, 72H, H-5$^{b1\text{-}b14,d}$, H-6$^{a,b1\text{-}b14,c1\text{-}c14}$, octyl OCH_2), 3.43 – 3.37 (m, 3H, H-6^{b4,b5}, octyl OCH_2), 2.75 (d, J = 3.7 Hz, 1H, -OH), 2.73 (d, J = 3.7 Hz, 1H, -OH), 1.58 – 1.54 (m, 2H, octyl CH_2), 1.28 – 1.23 (m, 10H, octyl CH_2), 0.85 (t, J = 6.8 Hz, 3H, octyl CH_3); ^{13}C NMR (150 MHz, $CDCl_3$) δ 166.18 (C=O), 165.82 (C=O), 165.78 (C=O), 165.67 (C=O), 165.50 (C=O), 165.47 (C=O), 165.45 (C=O), 165.41 (C=O), 165.37 (C=O), 165.3 (C=O), 138.47 (Ar), 138.43 (Ar), 138.22 (Ar), 138.16 (Ar), 138.14 (Ar), 138.07 (Ar), 138.03 (Ar), 137.87 (Ar), 137.81 (Ar), 137.79

(Ar), 137.74 (Ar), 137.72 (Ar), 137.63 (Ar), 133.47 (Ar), 133.35 (Ar), 133.06 (Ar), 129.97 (Ar), 129.94 (Ar), 129.66 (Ar), 129.61 (Ar), 129.58 (Ar), 129.56 (Ar), 129.53 (Ar), 129.51 (Ar), 129.37 (Ar), 128.58 (Ar), 128.56 (Ar), 128.45 (Ar), 128.41 (Ar), 128.36 (Ar), 128.33 (Ar), 128.30 (Ar), 128.25 (Ar), 128.15 (Ar), 128.12 (Ar), 128.08 (Ar), 128.01 (Ar), 127.98 (Ar), 127.96 (Ar), 127.94 (Ar), 127.90 (Ar), 127.79 (Ar), 127.76 (Ar), 127.71 (Ar), 127.65 (Ar), 127.52 (Ar), 127.50 (Ar), 127.45 (Ar), 127.43 (Ar), 127.40 (Ar), 127.37 (Ar), 107.25 (C-1), 107.23 (C-1), 107.03 (C-1), 106.92 (C-1^{c14}), 106.80 (C-1), 106.73 (C-1^{d}), 106.62 (C-1), 106.59 (C-1), 106.49 (C-1), 106.24 (C-1^{b1}), 106.00 (C-1^{a}), 83.92, 83.85, 83.79, 83.70, 83.61, 83.56, 83.53, 83.42, 83.32, 83.25, 83.18, 82.96, 82.94, 82.85, 82.77, 82.53, 82.48, 82.41, 82.33, 82.18, 82.07, 81.98, 81.94, 81.89, 75.91 (C-5^{d}), 74.23, 74.07, 73.98, 73.93, 73.89, 73.73, 73.49, 73.44, 73.36 73.33, 73.09, 72.89, 72.84, 72.71, 72.66, 72.37, 72.34, 72.11, 72.07, 71.73, 71.59, 71.55, 71.27, 71.06, 70.31, 70.28, 69.89, 69.86, 69.83, 69.54, 69.46, 67.63 (octyl OCH_2), 64.93 (C-6^{d}), 31.97 (octyl CH_2), 29.58 (octyl CH_2), 29.54 (octyl $OCH_2\underline{C}H_2$), 29.38 (octyl CH_2), 26.22 (octyl CH_2), 22.80 (octyl CH_2), 14.24 (octyl CH_3); MALDI-TOF MS Calcd for $C_{811}H_{790}O_{182}Na$ $[M + Na]^+$ *m/z*: 13471.8, found: 13473.8.

Synthesis of Ara*f*31 donor 3:

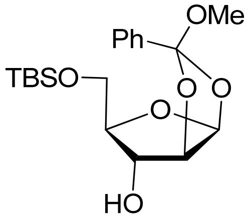

1,2-*O*-(*α*-Methoxybenzylidene)-5-*O*-*tert*-butydimethylsilyl-*β*-D-arabinofuranose (26b): To a stirred solution of **26a**[2] (20.0 g, 42 mmol) in anhydrous CH_2Cl_2 (50 mL) was added AcBr (9.3 mL, 126 mmol) and anhydrous CH_3OH (4.6 mL, 113 mmol) at 0 °C. The reaction mixture was stirred for 3 h at room temperature, and then 2,6-lutidine (24.5 mL, 210 mmol) and anhydrous CH_3OH (17 mL, 420 mmol) was added dropwise at 0 °C. After stirring for 24 h at room temperature, the solvent was evaporated *in vacuo*. The residue was purified by silica gel column chromatography (petroleum ether/acetone, 15:1, containing 0.2% Et_3N) to give a light yellow syrup, which was dissolved in CH_3OH (150 mL). Then CH_3ONa (5.0 M in CH_3OH) was added to adjust pH to ~9, the reaction mixture was stirred overnight, and neutralized by the addition of Dowex®50WX4 ion-exchange resin. The solution was filtered and the filtrate was evaporated *in vacuo*, the residue was purified by column chromatography on silica gel (petroleum ether/acetone, 2.5:1, containing 0.2% Et_3N) to afford a light yellow syrup (7.5 g, 28.0 mmol), which was dissolved in pyridine (50 mL), followed by the addition of Et_3N (9.8 mL, 70 mmol) and a catalytic amount of DMAP (34.2 mg, 0.28 mmol), the resulting mixture was then added TBSCl (4.64 g, 30.8 mmol) at 0 °C. 30 min later, the reaction solution was warmed to room temperature and stirred for 5 h. Then the mixture was added CH_3OH (1 mL) and stirred for another 30 min. The solvent was evaporated *in vacuo* and the residue was purified by column chromatography on silica gel (petroleum ether/ ethyl acetate, 6:1, containing 0.2% Et_3N) to yield **26b** (9.16 g, 57% for four steps) as light yellow syrup. R_f = 0.26 (petroleum ether/ethyl acetate, 3:1); $[\alpha]_D^{30}$ -17.6 (*c* 2.1, CH_2Cl_2); ^1H NMR (400 MHz, $CDCl_3$) δ 7.59 – 7.57 (m, 2H, Ar), 7.37 – 7.36 (m, 3H, Ar), 6.20 (d, *J* = 4.2 Hz, 1H, H-1), 4.84 (d, *J* = 4.2 Hz, 1H, H-2), 4.32 (d, *J* = 3.7 Hz, 1H, H-3), 4.05 (dd, *J* = 9.6, 5.5 Hz, 1H, H-4), 3.48 (dd, *J* = 10.0, 5.5 Hz, 1H, H-5a), 3.25 (t, *J* = 9.8 Hz, 1H,

H-5b), 3.15 (s, 3H, $-OCH_3$), 1.98 (br s, 1H, -OH), 0.76 (s, 9H, *t*-Bu), -0.16 (s, 3H, CH_3), -0.18 (s, 3H, CH_3); ^{13}C NMR (100 MHz, $CDCl_3$) δ 136.31 (Ar), 129.47 (Ar), 128.28 (Ar), 126.44 (Ar), 123.15 (PhC), 106.24 (C-1), 89.05 (C-4), 87.23 (C-2), 76.29 (C-3), 63.12 (C-5), 50.92 ($-OCH_3$), 25.94 (*t*-Bu), 18.32 (*t*-Bu), -5.45 (CH_3), -5.52 (CH_3); HRMS (ESI) Calcd for $C_{19}H_{30}O_6SiNa$ $[M + Na]^+$: 405.1704, found: 405.1698.

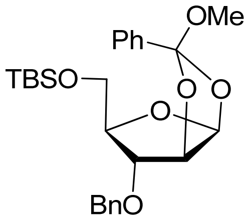

1,2-*O*-(*α*-Methoxybenzylidene)-3-*O*-benzyl-5-*O*-*tert*-butydimethylsilyl-*β*-D-arabinofuranose (26c): To a solution of **26b** (7.6 g, 19.9 mmol) and BnBr (2.8 mL, 23.8 mmol) in anhydrous DMF (40 mL) was slowly added NaH (0.87 g, 21.9 mmol, 60% in mineral oil) at 0 °C. The reaction mixture was slowly warmed to room temperature and stirred for 1 h, quenched with ice water, and extracted with CH_2Cl_2 (2 × 150 mL). The combined organic layer, after being washed with a saturated aqueous NH_4Cl solution (150 mL) and brine (150 mL), was dried over Na_2SO_4, filtered, and concentrated. The residue was purified by column chromatography on silica gel (petroleum ether/ethyl acetate, 30:1) to afford **26c** (8.1 g, 86%) as light yellow syrup. R_f = 0.43 (petroleum ether/ethyl acetate, 7:1); $[\alpha]_D^{30}$ -35.4 (*c* 2.3, CH_2Cl_2); ^1H NMR (400 MHz, $CDCl_3$) δ 7.58 – 7.55 (m, 2H, Ar), 7.37 – 7.27 (m, 8H, Ar), 6.21 (d, *J* = 4.3 Hz, 1H, H-1), 4.94 (d, *J* = 4.3 Hz, 1H, H-2), 4.59 (d, *J* = 11.9 Hz, 1H, $PhCH_2$), 4.55 (d, *J* = 11.9 Hz, 1H, $PhCH_2$), 4.05 (dd, *J* = 9.6, 5.5 Hz, 1H, H-4), 4.06 (s, 1H, H-3), 3.46 (dd, *J* = 10.0, 5.4 Hz, 1H, H-5a), 3.20 (t, *J* = 9.9 Hz, 1H, H-5b), 3.15 (s, 3H, $-OCH_3$), 0.75 (s, 9H, *t*-Bu), -0.18 (s, 3H, CH_3), -0.21 (s, 3H, CH_3); ^{13}C NMR (100 MHz, $CDCl_3$) δ 137.46 (Ar), 136.36 (Ar), 129.44 (Ar), 128.64 (Ar), 128.27 (Ar), 128.03 (Ar), 127.91 (Ar), 126.41 (Ar), 123.06 (PhC), 106.56 (C-1), 86.79 (C-4), 85.51 (C-2), 82.74 (C-3), 71.56 ($PhCH_2$), 62.91 (C-5), 50.94 ($-OCH_3$), 25.93 (*t*-Bu), 18.27 (*t*-Bu), -5.51 (CH_3), -5.52 (CH_3); HRMS (ESI) Calcd for $C_{26}H_{36}O_6SiNa$ $[M + Na]^+$: 495.2173, found: 495.2179.

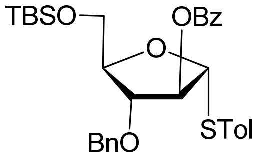

***p*-Tolyl 2-*O*-benzoyl-3-*O*-benzyl-5-*O*-*tert*-butydimethylsilyl-1-thio-*α*-D-arabinofuranoside (26):** To a mixture of **26c** (8.1 g, 17.1 mmol), *p*-thiocresol (2.55 g, 20.5 mmol) and freshly activated 4 Å molecular sieves (2.5 g) in CH_2Cl_2 (25 mL) under argon atmosphere was added a catalytic amount of $SnCl_4$ (1.7 mL, 1,7 mmol, 1.0 M in CH_2Cl_2) at 0 °C. After stirring for 30 min at the same temperature, the reaction was quenched by the addition of Et_3N (2 mL), filtered and concentrated. The crude residue was purified by column chromatography on silica gel (petroleum ether/acetone, 100:1) to yield **26** (6.0 g, 62%) as colorless syrup. R_f = 0.57 (petroleum ether/ethyl acetate, 7:1); $[\alpha]_D^{30}$ +108.6 (*c* 1.0, CH_2Cl_2); ^1H NMR (400 MHz, $CDCl_3$) δ 8.03 – 8.01 (m, 2H, Ar), 7.59 (t, *J* = 7.4 Hz, 1H, Ar), 7.47 – 7.43 (m, 4H, Ar), 7.39 – 7.25 (m, 5H, Ar), 7.12 (d, *J* = 8.0 Hz, 2H, Ar), 5.62 (s, 1H, H-1), 5.58 (t, *J* = 1.6 Hz, 1H, H-2), 4.82 (d, *J* = 12.1 Hz, 1H, $PhCH_2$), 4.65 (d, *J* = 12.0 Hz, 1H, $PhCH_2$), 4.45 (dd, *J* = 9.5, 4.7 Hz, 1H, H-4), 4.16 – 4.15 (m, 1H, H-3), 3.81 (d, *J* = 4.5 Hz, 2H, H-5a, H-5b), 2.33 (s, 3H, tolyl CH_3), 0.85 (s, 9H, *t*-Bu), 0.03 (s, 3H, CH_3), 0.02 (s, 3H, CH_3); ^{13}C NMR (100 MHz, $CDCl_3$) δ 165.51 (C=O), 137.75 (Ar), 137.68 (Ar), 133.54 (Ar), 132.63 (Ar), 130.83 (Ar), 129.94 (Ar), 129.80 (Ar), 129.56 (Ar), 128.57 (Ar), 128.54 (Ar), 128.03 (Ar), 127.92 (Ar), 91.61 (C-1), 83.63 (C-4), 83.02 (C-3), 82.63 (C-2), 72.38 ($PhCH_2$), 62.51 (C-5), 25.98 (*t*-Bu), 21.925 (tolyl CH_3), 18.43 (*t*-Bu), -5.21 (CH_3), -5.29 (CH_3); HRMS (ESI) Calcd for $C_{32}H_{40}O_5SSiNa$ $[M + Na]^+$: 587.2256, found: 587.2266.

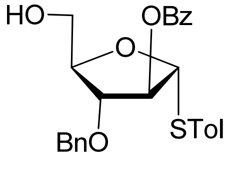

***p*-Tolyl 2-*O*-benzoyl-3-*O*-benzyl-1-thio-*α*-D-arabinofuranoside (27):** To solution of **26** (3.3 g, 5.84 mmol) in THF (20 mL) was added AcOH (670 μL, 11.7 mmol) and

TBAF (11.7 mL, 11.7 mmol, 1.0 M in THF), the resulting mixture was stirred overnight at 35 °C, and the solvent was evaporated *in vacuo* to give a residue, which was dissolved in EtOAc (100 mL). After being washed with a saturated aqueous $NaHCO_3$ solution (50 mL) and brine (50 mL), the organic layer was dried over Na_2SO_4, filtered, and concentrated. The residue was purified by column chromatography on silica gel (petroleum ether/acetone, 6:1) to afford **27** (2.42 g, 92%) as colorless syrup. R_f = 0.24 (petroleum ether/ethyl acetate, 3:1); $[\alpha]_D^{30}$ +147.3 (*c* 1.0, CH_2Cl_2); ^1H NMR (400 MHz, $CDCl_3$) δ 8.01 – 8.00 (m, 2H, Ar), 7.60 – 7.56 (m, 1H, Ar), 7.46 – 7.42 (m, 4H, Ar), 7.38 – 7.27 (m, 5H, Ar), 7.12 (d, *J* = 7.9 Hz, 2H, Ar), 5.61 (s, 1H, H-1), 5.55 (t, *J* = 1.5 Hz, 1H, H-2), 4.84 (d, *J* = 12.0 Hz, 1H, $PhCH_2$), 4.63 (d, *J* = 12.0 Hz, 1H, $PhCH_2$), 4.51 – 4.48 (m, 1H, H-4), 4.17 (dd, *J* = 5.5, 0.7 Hz, 1H, H-3), 3.90 (d, *J* = 12.1 Hz, 1H, H-5a), 3.73 – 3.67 (m, 1H, H-5b), 2.32 (s, 3H, tolyl CH_3), 1.81 (d, *J* = 3.3 Hz, 1H, -OH); ^{13}C NMR (100 MHz, $CDCl_3$) δ 165.48 (C=O), 138.08 (Ar), 137.54 (Ar), 133.65 (Ar), 132.92 (Ar), 130.39 (Ar), 129.94 (Ar), 129.89 (Ar), 129.40 (Ar), 128.67 (Ar), 128.62 (Ar), 128.08 (Ar), 128.03 (Ar), 91.98 (C-1), 83.22 (C-4), 82.84 (C-3), 82.45 (C-2), 72.68 ($PhCH_2$), 61.78 (C-5), 21.27 (tolyl CH_3); HRMS (ESI) Calcd for $C_{26}H_{26}O_5SNa$ $[M + Na]^+$: 473.1393, found: 473.1396.

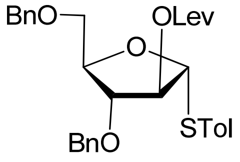

***p*-Tolyl 2-*O*-levulinoyl-3,5-di-*O*-benzyl-1-thio-*α*-D-arabinofuranoside (29):** To a solution of **29a**[3] (7.5 g, 15.7 mmol) in CH_3OH (30 mL) was added CH_3ONa (5.0 M in CH_3OH) to adjust pH to ~9, the reaction mixture was stirred for 2 h at room temperature, and neutralized by the addition of Dowex®50WX4 ion-exchange resin. The solution was filtered and concentrated to give a residue, which was dissolved in anhydrous CH_2Cl_2 (30 mL). To the resulting mixture was added EDC·HCl (4.5 g, 23.6 mmol) and DMAP (195 mg, 1.6 mmol), followed by the addition of Levulinoyl acid (1.9 mL, 18.8 mmol) at 0 °C. After stirring for 24 h at room temperature, the reaction mixture was diluted with CH_2Cl_2 (30 mL) and washed with a saturated aqueous

NaHCO3 solution (30 mL) and brine (30 mL). The organic layer extract was dried over Na_2SO_4, filtered, and concentrated. The residue was purified by column chromatography on silica gel (petroleum ether/acetone, 10:1) to afford **29** (7.2 g, 86% for two steps) as colorless syrup. R_f = 0.27 (petroleum ether/ethyl acetate, 3:1); $[\alpha]_D^{30}$ -142.4 (*c* 0.5, CH_2Cl_2); ^{1}H NMR (400 MHz, $CDCl_3$) δ 7.41 (d, *J* = 8.0 Hz, 2H, Ar), 7.33 – 7.25 (m, 10H, Ar), 7.10 (d, *J* = 7.9 Hz, 2H, Ar), 5.49 (s, 1H, H-1), 5.30 (s, 1H, H-2), 4.73 (d, *J* = 12.0 Hz, 1H, $PhCH_2$), 4.55 – 4.52 (m, 2H, , $PhCH_2$), 4.50 – 4.47 (m, 2H, $PhCH_2$, H-4), 3.98 (d, *J* = 4.8 Hz, 1H, H-3), 3.66 (dd, *J* = 10.9, 3.9 Hz, 1H, H-5a), 3.61 (dd, *J* = 10.8, 4.6 Hz, 1H, H-5b), 2.77 – 2.63 (m, 2H, Lev CH_2), 2.56 – 2.48 (m, 2H, Lev CH_2), 2.31 (s, 3H, tolyl CH_3), 2.15 (s, 3H, Lev CH_3); ^{13}C NMR (100 MHz, $CDCl_3$) δ 206.21 (C=O), 171.79 (C=O), 138.22 (Ar), 137.75 (Ar), 137.69 (Ar), 132.53 (Ar), 130.68 (Ar), 129.83 (Ar), 128.51 (Ar), 128.47 (Ar), 128.09 (Ar), 127.93 (Ar), 127.84 (Ar), 127.76 (Ar), 91.41 (C-1), 83.22 (C-3), 82.25 (C-2), 82.00 (C-4), 73.51 ($PhCH_2$), 72.37 ($PhCH_2$), 69.08 (C-5), 37.90 (Lev CH_2), 29.90 (Lev CH_3), 28.02 (Lev CH_2), 21.24 (tolyl CH_3); HRMS (ESI) Calcd for $C_{31}H_{34}O_6SNa$ $[M + Na]^+$: 557.1968, found: 557.1967.

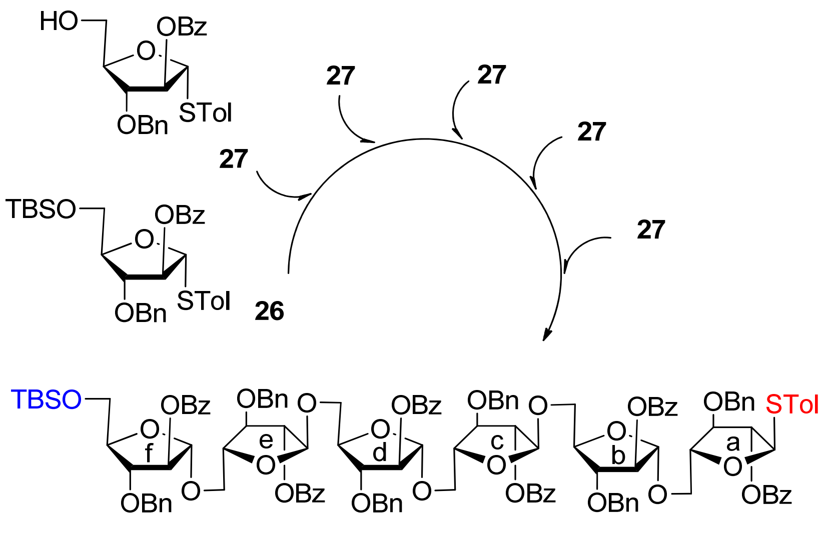

***p*-Tolyl 2-*O*-benzoyl-3-*O*-benzyl-5-*O*-*tert*-butydimethylsilyl-*α*-D-arabinofuranosyl[f]-(1→5)-2-*O*-benzoyl-3-*O*-benzyl-*α*-D-arabinofuranosyl[e]-(1→5)-2-*O*-benzoyl-3-*O*-benzyl-*α*-D-arabinofuranosyl[d]-(1→5)-2-*O*-benzoyl-3-*O*-benzyl-*α*-D-arabinofuranosyl[c]-(1→5)-2-*O*-benzoyl-3-*O*-benzyl-*α*-D-arabinofuranosyl[b]-(1→**

5)-2-*O*-benzoyl-3-*O*-benzyl-1-thio-*α*-D-arabinofuranoside[a] (17): A mixture of **26** (526.5 mg, 0.932 mmol), TTBP (1.25 g, 5.05 mmol) and freshly activated 4 Å molecular sieves (7.0 g) in anhydrous CH_2Cl_2 (36 mL) under argon atmosphere was stirred for 20 min at room temperature, and cooled to -78 °C. 5 min later, stoichiometric *p*-TolSCl (128.6 μL, 0.932 mmol) was added, followed by dropwise addition of AgOTf (4.7 mL, 1.864 mmol, 0.4 mol/L in anhydrous toluene/CH_2Cl_2, 3:1). After another 5 min, a solution of **27** (400.0 mg, 0.888 mmol) in anhydrous CH_2Cl_2 (3 mL) was slowly added. The resulting mixture was stirred at -78 °C for 10 min, warmed to room temperature, and cooled back to -78 °C. 5 min later, stoichiometric *p*-TolSCl (122.5 μL, 0.888 mmol) was added, followed by dropwise addition of AgOTf (4.4 mL, 1.776 mmol, 0.4 mol/L in anhydrous toluene/CH_2Cl_2, 3:1). After another 5 min, a solution of **27** (380.0 mg, 0.843 mmol) in anhydrous CH_2Cl_2 (3 mL) was slowly added. The resulting mixture was stirred at -78 °C for 10 min, warmed to room temperature, and cooled back to -78 °C. 5 min later, stoichiometric *p*-TolSCl (116.3 μL, 0.843 mmol) was added, followed by dropwise addition of AgOTf (4.2 mL, 1.686 mmol, 0.4 mol/L in anhydrous toluene/CH_2Cl_2, 3:1). After another 5 min, a solution of **27** (360.0 mg, 0.799 mmol) in anhydrous CH_2Cl_2 (3 mL) was slowly added. The resulting mixture was stirred at -78 °C for 10 min, warmed to room temperature, and cooled back to -78 °C. 5 min later, stoichiometric *p*-TolSCl (110.2 μL, 0.799 mmol) was added, followed by dropwise addition of AgOTf (4.0 mL, 1.598 mmol, 0.4 mol/L in anhydrous toluene/CH_2Cl_2, 3:1). After another 5 min, a solution of **27** (336.0 mg, 0.746 mmol) in anhydrous CH_2Cl_2 (3 mL) was slowly added. The resulting mixture was stirred at -78 °C for 10 min, warmed to room temperature, and cooled back to -78 °C. 5 min later, stoichiometric *p*-TolSCl (102.9 μL, 0.746 mmol) was added, followed by dropwise addition of AgOTf (3.7 mL, 1.492 mmol, 0.4 mol/L in anhydrous toluene/CH_2Cl_2, 3:1). After another 5 min, a solution of **27** (400.0 mg, 0.888 mmol) in anhydrous CH_2Cl_2 (3 mL) was slowly added. The resulting mixture was stirred at -78 °C for 10 min, warmed to room temperature, and quenched with Et_3N (1 mL) and filtered through Celite. After removal of the solvent, the desired product was purified by

column chromatography on silica gel (petroleum ether/acetone, 6:1) to afford **17** (1.20 g, 73%) as white foam. R_f = 0.34 (petroleum ether/ethyl acetate, 3:1); $[\alpha]_D^{30}$ +103.9 (*c* 0.6, CH_2Cl_2); ^1H NMR (600 MHz, $CDCl_3$) δ 8.03 – 7.98 (m, 12H, Ar), 7.57 (t, *J* = 7.4 Hz, 1H, Ar), 7.54 – 7.51 (m, 5H, Ar), 7.44 – 7.38 (m, 14H, Ar), 7.34 (d, *J* = 7.2 Hz, 2H, Ar), 7.27 (t, *J* = 7.3 Hz, 1H, Ar), 7.22 – 7.13 (m, 26H, Ar), 7.06 (d, *J* = 8.0 Hz, 2H, Ar), 5.61 (s, 1H, H-1[a]), 5.57 (t, *J* = 1.8 Hz, 1H, H-2[a]), 5.40 (d, *J* = 1.1 Hz, 1H, H-2), 5.38 (d, *J* = 1.1 Hz, 1H, H-2), 5.37 (d, *J* = 1.1 Hz, 2H, H-2), 5.36 (d, *J* = 1.0 Hz, 1H, H-2[f]), 5.24 (s, 1H, H-1), 5.23 (s, 1H, H-1), 5.21 (br s, 2H, H-1), 5.20 (s, 1H, H-1), 4.77 (d, *J* = 12.0 Hz, 1H, $PhCH_2$), 4.64 – 4.60 (m, 2H, $PhCH_2$), 4.58 – 4.53 (m, 5H, H-4[a], $PhCH_2$), 4.49 (d, *J* = 12.2 Hz, 1H, $PhCH_2$), 4.45 – 4.42 (m, 4H, $PhCH_2$), 4.27 (dd, *J* = 5.5, 1.2 Hz, 1H, H-3[a]), 4.17 (dd, *J* = 9.1, 3.8 Hz, 1H, H-4), 4.13 – 4.06 (m, 7H, H-3 ×4, H-4 ×4), 4.01 (dd, *J* = 9.3, 4.3 Hz, 1H, H-4[f]), 3.96 – 3.94 (m, 2H, H-3[f], H-5[a]), 3.85 – 3.80 (m, 4H, H-5 ×4), 3.75 (dd, *J* = 11.3, 3.9 Hz, 1H, H-5[a]), 3.70 – 3.60 (m, 6H, H-5 ×6), 2.28 (s, 3H, tolyl CH_3), 0.80 (s, 9H, *t*-Bu), -0.02 (s, 3H, CH_3), -0.04 (s, 3H, CH_3); ^{13}C NMR (150 MHz, $CDCl_3$) δ 165.56 (C=O), 165.44 (C=O), 165.39 (C=O), 165.38 (C=O), 165.37 (×2, C=O), 137.97 (Ar), 137.95 (Ar), 137.90 (Ar), 137.77 (Ar), 137.63 (Ar), 133.48 (Ar), 133.46 (Ar), 133.39 (Ar), 132.56 (Ar), 130.70 (Ar), 129.99 (Ar), 129.93 (Ar), 129.88 (Ar), 129.82 (Ar), 129.68 (Ar), 129.65 (Ar), 129.63 (Ar), 129.51 (Ar), 128.62 (Ar), 128.54 (Ar), 128.41 (Ar), 128.37 (Ar), 128.34 (Ar), 128.02 (Ar), 127.86 (Ar), 127.84 (Ar), 127.83 (Ar), 127.78 (Ar), 127.71 (Ar), 127.67 (Ar), 127.66 (Ar), 127.62 (Ar), 106.29 (C-1), 106.27 (×2, C-1), 106.25 (C-1), 91.52 (C-1[a]), 83.95, 83.40, 83.30, 83.26, 83.13 (C-3[a]), 83.00, 82.45 (C-2[a]), 82.20, 82.17, 82.11, 81.98, 81.93, 81.92, 81.90, 81.75 (C-4[a]), 72.55 ($PhCH_2$), 72.31 ($PhCH_2$), 72.23 ($PhCH_2$), 72.20 ($PhCH_2$), 72.19 ($PhCH_2$), 72.06 ($PhCH_2$), 65.52 (×2, C-5), 65.46 (C-5), 65.45 (C-5), 62.48 (C-5[f]), 25.96 (*t*-Bu), 21.22 (tolyl CH_3), 18.42 (*t*-Bu), -5.23 (CH_3), -5.33 (CH_3); HRMS (ESI) Calcd for $C_{127}H_{130}O_{30}SSiNa$ $[M + Na]^+$: 2217.8029, found: 2217.8101.

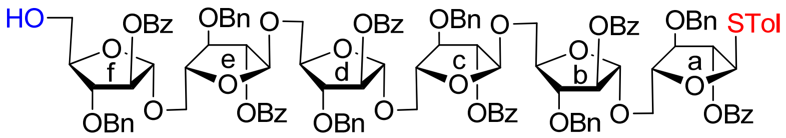

***p*-Tolyl 2-*O*-benzoyl-3-*O*-benzyl-*α*-D-arabinofuranosyl^{f}-(1→5)-2-*O*-benzoyl-3-*O*-benzyl-*α*-D-arabinofuranosyl^{e}-(1→5)-2-*O*-benzoyl-3-*O*-benzyl-*α*-D-arabinofuranosyl^{d}-(1→5)-2-*O*-benzoyl-3-*O*-benzyl-*α*-D-arabinofuranosyl^{c}-(1→5)-2-*O*-benzoyl-3-*O*-benzyl-*α*-D-arabinofuranosyl^{b}-(1→5)-2-*O*-benzoyl-3-*O*-benzyl-1-thio-*α*-D-arabinofuranoside^{a} (12):** To a solution of **17** (1.0 g, 0.455 mmol) in THF (10 mL) was added AcOH (104 μL, 1.82 mmol) and TBAF (1.8 mL, 1.8 mmol, 1.0 M in THF), the resulting mixture was stirred at 35 °C for 24 h, and the solvent was evaporated *in vacuo* to give a residue, which was dissolved in EtOAc (80 mL). After being washed with a saturated aqueous $NaHCO_3$ solution (40 mL) and brine (40 mL), the organic layer was dried over Na_2SO_4, filtered, and concentrated. The residue was purified by column chromatography on silica gel (petroleum ether/acetone, 3:1) to afford **12** (872 mg, 92%) as white foam. R_f = 0.47 (petroleum ether/ethyl acetate, 1.5:1); $[\alpha]_D^{30}$ +107.9 (*c* 0.5, CH_2Cl_2); ^{1}H NMR (600 MHz, $CDCl_3$) δ 8.03 – 8.00 (m, 10H, Ar), 7.98 – 7.96 (m, 2H, Ar), 7.57 (t, *J* = 7.4 Hz, 1H, Ar), 7.54 – 7.51 (m, 5H, Ar), 7.44 – 7.38 (m, 14H, Ar), 7.34 (d, *J* = 7.2 Hz, 2H, Ar), 7.27 (t, *J* = 7.4 Hz, 1H, Ar), 7.24 – 7.13 (m, 26H, Ar), 7.06 (d, *J* = 8.0 Hz, 2H, Ar), 5.61 (s, 1H, H-1^{a}), 5.57 (t, *J* = 1.8 Hz, 1H, H-2^{a}), 5.40 (d, *J* = 1.3 Hz, 1H, H-2^{b}), 5.38 (d, *J* = 1.2 Hz, 1H, H-2), 5.37 (d, *J* = 1.2 Hz, 2H, H-2), 5.33 (d, *J* = 1.2 Hz, 1H, H-2^{f}), 5.24 (s, 1H, H-1^{b}), 5.23 (br s, 2H, H-1), 5.22 (s, 1H, H-1), 5.20 (s, 1H, H-1^{f}), 4.77 (d, *J* = 12.1 Hz, 1H, $PhCH_2$), 4.64 – 4.60 (m, 2H, $PhCH_2$), 4.58 – 4.54 (m, 5H, H-4^{a}, $PhCH_2$), 4.49 (d, *J* = 12.1 Hz, 1H, $PhCH_2$), 4.45 – 4.43 (m, 3H, $PhCH_2$), 4.41 (d, *J* = 12.2 Hz, 1H, $PhCH_2$), 4.27 (dd, *J* = 5.5, 1.3 Hz, 1H, H-3^{a}), 4.17 (dd, *J* = 9.1, 3.8 Hz, 1H, H-4^{b}), 4.13 – 4.10 (m, 4H, H-3^{b}, H-4^{c,d,e}), 4.07 (br s, 3H, H-3^{c,d,e}), 4.00 – 3.98 (m, 1H, H-4^{f}), 3.95 (dd, *J* = 11.4, 4.0 Hz, 1H, H-5^{a}), 3.93 (d, *J* = 5.5 Hz, 1H, H-3^{f}), 3.85 – 3.71 (m, 6H, H-5^{a,b,c,d,e,f}), 3.66 – 3.61 (m, 4H, H-5^{b,c,d,e}), 3.53 – 3.49 (m, 1H, H-5^{f}), 2.27 (s, 3H, tolyl CH_3), 1.68 (dd, *J* = 8.4, 4.3 Hz, 1H, -OH); ^{13}C NMR (150 MHz, $CDCl_3$) δ 165.56 (C=O), 165.44 (C=O), 165.39 (C=O), 165.37 (×2, C=O), 165.30 (C=O), 137.95 (Ar), 137.77 (Ar), 137.70 (Ar),

137.63 (Ar), 133.57 (Ar), 133.48 (Ar), 133.45 (Ar), 133.39 (Ar), 132.56 (Ar), 130.70 (Ar), 129.98 (Ar), 129.93 (Ar), 129.91 (Ar), 129.82 (Ar), 129.64 (Ar), 129.62 (Ar), 129.53 (Ar), 129.51 (Ar), 128.63 (Ar), 128.53 (Ar), 128.45 (Ar), 128.41 (Ar), 128.37 (Ar), 128.02 (Ar), 127.87 (Ar), 127.82 (Ar), 127.71 (Ar), 127.67 (Ar), 106.38 (C-1), 106.30 (C-1), 106.28 (×2, C-1), 106.25 (C-1), 91.52 (C-1[a]), 83.46, 83.31, 83.28, 83.13 (C-3[a]), 82.89 (C-3[f]), 82.44 (C-2[b]), 82.18, 82.16, 81.98, 81.92, 81.87, 81.74 (C-4[a]), 72.55 ($PhCH_2$), 72.31 ($PhCH_2$), 72.25 ($PhCH_2$), 72.21 ($PhCH_2$), 72.20 ($PhCH_2$), 72.17 ($PhCH_2$), 65.65 (C-5), 65.60 (C-5), 65.55 (C-5), 65.54 (C-5), 65.45 (C-5), 62.03 (C-5[f]), 21.22 (tolyl CH_3); HRMS (ESI) Calcd for $C_{121}H_{116}O_{30}SNa$ $[M + Na]^+$: 2103.7164, found: 2103.7183.

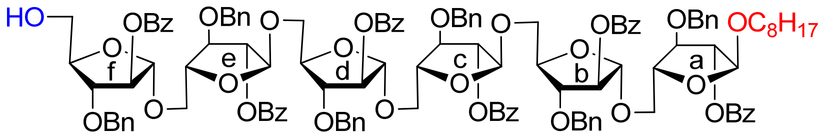

Octyl 2-*O*-benzoyl-3-*O*-benzyl-*α*-D-arabinofuranosyl[f]-(1→5)-2-*O*-benzoyl-3-*O*-benzyl-*α*-D-arabinofuranosyl[e]-(1→5)-2-*O*-benzoyl-3-*O*-benzyl-*α*-D-arabinofuranosyl[d]-(1→5)-2-*O*-benzoyl-3-*O*-benzyl-*α*-D-arabinofuranosyl[c]-(1→5)-2-*O*-benzoyl-3-*O*-benzyl-*α*-D-arabinofuranosyl[b]-(1→5)-2-*O*-benzoyl-3-*O*-benzyl-*α*-D-arabinofuranoside[a] (32): A mixture of **17** (500 mg, 0.228 mmol), TTBP (68 mg, 0.274 mmol) and freshly activated 4 Å molecular sieves (2.0 g) in anhydrous CH_2Cl_2 (20 mL) under argon atmosphere was stirred for 20 min at room temperature, and cooled to -78 °C. 5 min later, stoichiometric *p*-TolSCl (31.5 μL, 0.228 mmol) was added, followed by dropwise addition of AgOTf (1.1 mL, 0.455 mmol, 0.4 mol/L in anhydrous toluene/CH_2Cl_2, 3:1). After another 5 min, 1-octanol (72 μL, 0.455 mmol) was added. The reaction mixture was warmed to room temperature in 10 min, quenched with Et_3N (0.5 mL) and filtered through Celite. After removal of the solvent, the residue was purified by column chromatography on silica gel (petroleum ether/ethyl acetate, 5:1) to give a crude product, which was dissolved in THF (5 mL), followed by the addition of AcOH (52 μL, 0.91 mmol) and TBAF (0.9 mL, 0.9 mmol, 1.0 M in THF). The resulting mixture was stirred at 35 °C for 24 h, and the solvent

was evaporated *in vacuo* to give a residue, which was dissolved in EtOAc (50 mL). After being washed with a saturated aqueous $NaHCO_3$ solution (20 mL) and brine (20 mL), the organic layer was dried over Na_2SO_4, filtered, and concentrated. The residue was purified by column chromatography on silica gel (toluene/acetonitrile, 16:1) to afford **32** (409 mg, 86% for two steps) as white foam. R_f = 0.53 (petroleum ether/ethyl acetate, 1.5:1); $[\alpha]_D^{30}$ -84.2 (*c* 0.6, CH_2Cl_2); ^1H NMR (600 MHz, $CDCl_3$) δ 8.04 – 8.00 (m, 10H, Ar), 7.98 – 7.96 (m, 2H, Ar), 7.58 – 7.55 (m, 1H, Ar), 7.54 – 7.51 (m, 5H, Ar), 7.44 – 7.38 (m, 12H, Ar), 7.32 (d, *J* = 7.3 Hz, 2H, Ar), 7.26 – 7.14 (m, 28H, Ar), 5.43 (d, *J* = 1.3 Hz, 1H, H-2[b]), 5.39 (d, *J* = 1.5 Hz, 1H, H-2[a]), 5.38 (d, *J* = 1.3 Hz, 1H, H-2), 5.37 (d, *J* = 1.3 Hz, 2H, H-2), 5.33 (d, *J* = 1.3 Hz, 1H, H-2[b]), 5.27 (s, 1H, H-1[b]), 5.23 (s, 1H, H-1), 5.23 (s, 1H, H-1), 5.22 (s, 1H, H-1), 5.20 (s, 1H, H-1[f]), 5.13 (s, 1H, H-1[a]), 4.77 (d, *J* = 12.0 Hz, 1H, $PhCH_2$), 4.63 – 4.60 (m, 2H, $PhCH_2$), 4.57 – 4.54 (m, 4H, $PhCH_2$), 4.49 (d, *J* = 12.2 Hz, 1H, $PhCH_2$), 4.45 – 4.43 (m, 3H, $PhCH_2$), 4.41 (d, *J* = 12.2 Hz, 1H, $PhCH_2$), 4.33 – 4.31 (m, 1H, H-4[a]), 4.19 – 4.15 (m, 2H, H-4[b], H-3[a]), 4.14 – 4.09 (m, 4H, H-4[c,d,e], H-3[b]), 4.07 – 4.06 (m, 3H, H-3[c,d,e]), 4.00 – 3.98 (m, 1H, H-4[f]), 3.93 – 3.90 (m, 2H, H-4[f], H-5[a]), 3.85 – 3.78 (m, 4H, H-5[b,c,d,e]), 3.75 – 3.70 (m, 3H, H-5[a,f], octyl OCH_2), 3.66 – 3.61 (m, 4H, H-5[b,c,d,e]), 3.53 – 3.49 (m, 1H, H-5[f]), 3.45 (dt, *J* = 9.7, 6.6 Hz, 1H, octyl OCH_2), 1.68 (t, *J* = 4.4 Hz, 1H, -OH), 1.63 – 1.58 (m, 2H, octyl CH_2), 1.35 – 1.25 (m, 10H octyl CH_2), 0.87 (t, *J* = 7.0 Hz, 3H octyl OCH_3); ^{13}C NMR (150 MHz, $CDCl_3$) δ 165.67 (C=O), 165.42 (C=O), 165.39 (C=O), 165.37 (×2, C=O), 165.30 (C=O), 138.03 (Ar), 137.99 (Ar), 137.96 (Ar), 137.70 (Ar), 133.57 (Ar), 133.45 (Ar), 133.41 (Ar), 133.38 (Ar), 129.93 (Ar), 129.91 (Ar), 129.83 (Ar), 129.72 (Ar), 129.68 (Ar), 129.65 (Ar), 129.64 (Ar), 129.62 (Ar), 129.54 (Ar), 128.63 (Ar), 128.60 (Ar), 128.45 (Ar), 128.39 (Ar), 128.37 (Ar), 127.99 (Ar), 127.87 (Ar), 127.84 (Ar), 127.82 (Ar), 127.76 (Ar), 127.66 (Ar), 106.38 (C-1[f]), 106.30 (C-1), 106.27 (×2, C-1), 106.25 (C-1), 106.08 (C-1[a]), 83.46, 83.44, 83.35, 83.31, 83.28, 82.89 (C-3[f]), 82.35 (C-2[a]), 82.16, 81.97, 81.92, 81.90, 81.87, 81.61 (C-4[a]), 72.47 ($PhCH_2$), 72.26 (×2, $PhCH_2$), 72.19 (×2, $PhCH_2$), 72.17 ($PhCH_2$), 67.73 (octyl OCH_2), 65.72 (C-5[a]), 65.65 (C-5), 65.60 (C-5), 65.54 (×2, C-5), 62.03 (C-5[f]), 31.97 (octyl CH_2), 29.58 (octyl CH_2), 29.53 (octyl $OCH_2\underline{C}H_2$), 29.39 (octyl CH_2), 26.20 (octyl

CH_2), 22.79 (octyl CH_2), 14.24 (octyl CH_3); HRMS (ESI) Calcd for $C_{122}H_{130}O_{31}N$ [M + $NH_4]^+$: 2104.8621, found: 2104.8641.

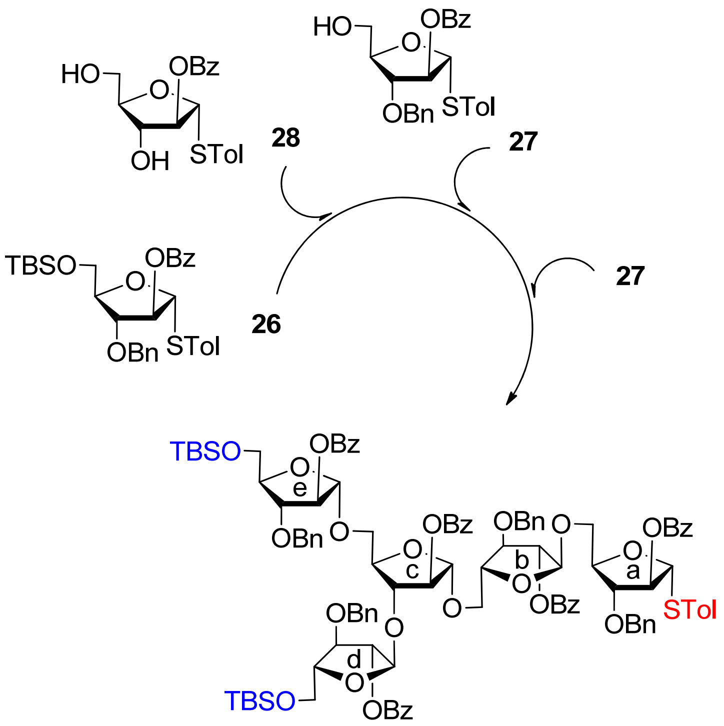

***p*-Tolyl 2-*O*-benzoyl-3-*O*-benzyl-5-*O*-*tert*-butydimethylsilyl-*α*-D-arabinofuranosyl[e]-(1→5)-(2-*O*-benzoyl-3-*O*-benzyl-5-*O*-*tert*-butydimethylsilyl-*α*-D-arabinofuranosyl[d])-(1→3)-2-*O*-benzoyl-*α*-D-arabinofuranosyl[c]-(1→5)-2-*O*-benzoyl-3-*O*-benzyl-*α*-D-arabinofuranosyl[b]-(1→5)-2-*O*-benzoyl-3-*O*-benzyl-1-thio-*α*-D-arabinofuranoside[a] (16):** A mixture of **26** (1.15 g, 2.039 mmol), TTBP (1.17 g, 4.72 mmol) and freshly activated 4 Å molecular sieves (7.0 g) in anhydrous CH_2Cl_2 (40 mL) under argon atmosphere was stirred for 20 min at room temperature, and cooled to -78 °C. 5 min later, stoichiometric *p*-TolSCl (281.3 μL, 2.039 mmol) was added, followed by dropwise addition of AgOTf (10.2 mL, 4.078 mmol, 0.4 mol/L in anhydrous toluene/CH_2Cl_2, 3:1). After another 5 min, a solution of **28** (350.0 mg, 0.971 mmol) in anhydrous CH_2Cl_2 (4 mL) was slowly added. The resulting mixture was slowly warmed to room temperature within 2 h and cooled back to -78 °C. 5 min later, stoichiometric *p*-TolSCl (134.0 μL, 0.971 mmol) was added, followed by dropwise addition of AgOTf (4.9 mL, 1.942 mmol, 0.4 mol/L in anhydrous toluene/CH_2Cl_2, 3:1). After another 5 min, a solution of **27** (415.6 mg, 0.922 mmol) in

anhydrous CH_2Cl_2 (4 mL) was slowly added. The resulting mixture was stirred at -78 °C for 10 min, warmed to room temperature, and cooled back to -78 °C. 5 min later, stoichiometric *p*-TolSCl (127.2 μL, 0.922 mmol) was added, followed by dropwise addition of AgOTf (4.6 mL, 1.844 mmol, 0.4 mol/L in anhydrous toluene/CH_2Cl_2, 3:1). After another 5 min, a solution of **27** (481.2 mg, 1.068 mmol) in anhydrous CH_2Cl_2 (4 mL) was slowly added. The resulting mixture was stirred at -78 °C for 10 min, warmed to room temperature, and quenched with Et_3N (1 mL) and filtered through Celite. After removal of the solvent, the desired product was purified by column chromatography on silica gel (petroleum ether/acetone, 7:1) to afford **16** (1.36 g, 78%) as white foam. R_f = 0.48 (petroleum ether/ethyl acetate, 3:1); $[\alpha]_D^{30}$ +78.2 (*c* 0.4, CH_2Cl_2); ^1H NMR (600 MHz, $CDCl_3$) δ 8.04 – 8.02 (m, 2H, Ar), 8.01 – 7.99 (m, 6H, Ar), 7.97 – 7.96 (m, 2H, Ar), 7.56 (td, *J* = 7.6, 1.1 Hz, 2H, Ar), 7.53 – 7.48 (m, 2H, Ar), 7.43 – 7.939 (m, 9H, Ar), 7.37 – 7.33 (m, 6H, Ar), 7.28 – 7.18 (m, 15H, Ar), 7.16 – 7.12 (m, 3H, Ar), 7.06 (d, *J* = 8.0 Hz, 2H, Ar), 5.61 (s, 1H, H-1^a), 5.57 (t, *J* = 1.9 Hz, 1H, H-2^a), 5.44 (s, 1H, H-1^d), 5.42 (d, *J* = 1.4 Hz, 1H, H-2^b), 5.41 (d, *J* = 1.1 Hz, 1H, H-2^e), 5.38 (d, *J* = 1.6 Hz, 1H, H-2^c), 5.34 (d, *J* = 1.2 Hz, 1H, H-2^d), 5.27 (s, 1H, H-1^e), 5.26 (s, 1H, H-1^c), 5.21 (s, 1H, H-1^b), 4.76 (d, *J* = 12.0 Hz, 1H, $PhCH_2$), 4.70 (d, *J* = 12.1 Hz, 1H, $PhCH_2$), 4.63 – 4.59 (m, 3H, $PhCH_2$), 4.56 (dd, *J* = 9.1, 4.0 Hz, 1H, H-4^a), 4.54 – 4.47 (m, 3H, $PhCH_2$), 4.45 (dd, *J* = 6.1, 1.1 Hz, 1H, H-3^c), 4.27 (dd, *J* = 5.5, 1.3 Hz, 1H, H-3^a), 4.22 – 4.18 (m, 3H, H-4^{b,c,e}), 4.16 – 4.15 (m, 1H, H-3^b), 4.13 (dd, *J* = 9.3, 4.4 Hz, 1H, H-4^d), 4.00 (d, *J* = 5.1 Hz, 2H, H-3^{d,e}), 3.97 – 3.94 (m, 2H, H-5^{a,c}), 3.84 (dd, *J* = 11.4, 4.1 Hz, 1H, H-5^b), 3.78 – 3.65 (m, 7H, H-5^{a,b,c,d,e}), 2.27 (s, 3H, tolyl CH_3), 0.79 (s, 9H, *t*-Bu), 0.76 (s, 9H, *t*-Bu), -0.02 (s, 3H, CH_3), -0.05 (s, 3H, CH_3), -0.07 (s, 3H, CH_3), -0.09 (s, 3H, CH_3); ^{13}C NMR (150 MHz, $CDCl_3$) δ 165.58 (C=O), 165.49 (C=O), 165.47 (C=O), 165.34 (C=O), 165.23 (C=O), 138.02 (Ar), 137.99 (Ar), 137.96 (Ar), 137.78 (Ar), 137.60 (Ar), 133.47 (Ar), 133.39 (Ar), 133.33 (Ar), 133.32 (Ar), 133.28 (Ar), 132.56 (Ar), 130.73 (Ar), 130.01 (Ar), 129.99 (Ar), 129.95 (Ar), 129.91 (Ar), 129.82 (Ar), 129.78 (Ar), 129.75 (Ar), 129.67 (Ar), 129.58 (Ar), 129.53 (Ar), 128.63 (Ar), 128.58 (Ar), 128.53 (Ar), 128.52 (Ar), 128.44 (Ar), 128.39 (Ar), 128.37 (Ar), 128.02 (Ar), 127.97 (Ar), 127.87 (Ar), 127.83

(Ar), 127.70 (Ar), 127.69 (Ar), 127.61 (Ar), 106.40 (C-1[e]), 106.31 (C-1[b]), 106.18 (C-1[c]), 105.51 (C-1[d]), 91.49 (C-1[a]), 84.12 (C-4[d]), 83.86, 83.32 (C-3[b]), 83.15 (C-3), 83.13 (C-3), 82.88 (C-3), 82.67 (C-2[c]), 82.46 (C-2[a]), 82.22, 82.21, 82.08, 81.99, 81.78 (C-4[a]), 80.06 (C-3[c]), 72.55 ($PhCH_2$), 72.47 ($PhCH_2$), 72.16 ($PhCH_2$), 71.92 ($PhCH_2$), 65.61 (C-5[b]), 65.57 (C-5[a]), 65.28 (C-5[c]), 62.38 (C-5), 62.37 (C-5), 25.97 (*t*-Bu), 25.95 (*t*-Bu), 21.22 (tolyl CH_3), 18.42 (*t*-Bu), 18.37 (*t*-Bu), -5.22 (CH_3), -5.28 (CH_3), -5.33 (CH_3), -5.38 (CH_3); HRMS (ESI) Calcd for $C_{107}H_{120}O_{25}SSi_2Na$ $[M + Na]^+$: 1915.7270, found: 1915.7243.

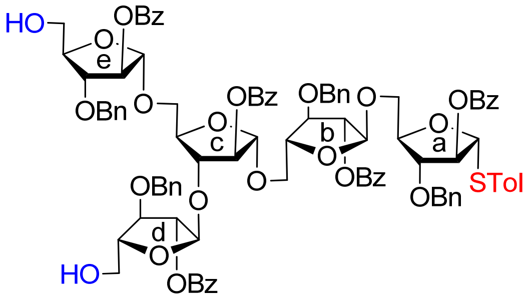

***p*-Tolyl 2-*O*-benzoyl-3-*O*-benzyl-*α*-D-arabinofuranosyl[e]-(1→5)-(2-*O*-benzoyl-3-*O*-benzyl-*α*-D-arabinofuranosyl[d])-(1→3)-2-*O*-benzoyl-*α*-D-arabinofuranosyl[c]-(1→5)-2-*O*-benzoyl-3-*O*-benzyl-*α*-D-arabinofuranosyl[b]-(1→5)-2-*O*-benzoyl-3-*O*-benzyl-1-thio-*α*-D-arabinofuranoside[a] (11):** To solution of **16** (2.17 g, 1.146 mmol) in THF (20 mL) was added AcOH (262 μL, 4.58 mmol) and TBAF (4.6 mL, 4.6 mmol, 1.0 M in THF), the resulting mixture was stirred at 35 °C for 24 h, and the solvent was evaporated *in vacuo* to give a residue, which was dissolved in EtOAc (100 mL). After being washed with a saturated aqueous $NaHCO_3$ solution (50 mL) and brine (50 mL), the organic layer was dried over Na_2SO_4, filtered, and concentrated. The residue was purified by column chromatography on silica gel (petroleum ether/acetone, 3:1) to afford **11** (1.82 g, 95%) as white foam. R_f = 0.28 (petroleum ether/ethyl acetate, 1:1); $[\alpha]_D^{30}$ +100.1 (*c* 0.6, CH_2Cl_2); ^1H NMR (600 MHz, $CDCl_3$) δ 8.04 – 7.97 (m, 10H, Ar), 7.59 – 7.56 (m, 2H, Ar), 7.53 (t, *J* = 7.4 Hz, 1H, Ar), 7.49 (t, *J* = 7.4 Hz, 1H, Ar), 7.45 – 7.39 (m, 9H, Ar), 7.35 (t, *J* = 7.5 Hz, 1H, Ar), 7.29 – 7.14 (m, 18H, Ar), 7.06 (d, *J* = 8.0 Hz, 2H, Ar), 5.61 (s, 1H, H-1[a]), 5.57 (s, 1H, H-2[a]), 5.51 (s, 1H, H-1[d]), 5.42 (d,

J = 0.7 Hz, 1H, H-2[b]), 5.40 (d, J = 0.7 Hz, 1H, H-2[e]), 5.37 (d, J = 1.3 Hz, 1H, H-2[c]), 5.35 (d, J = 1.3 Hz, 1H, H-2[d]), 5.30 (s, 1H, H-1[c]), 5.25 (s, 1H, H-1[e]), 5.23 (s, 1H, H-1[b]), 4.78 (d, J = 12.1 Hz, 1H, $PhCH_2$), 4.72 (d, J = 12.0 Hz, 1H, $PhCH_2$), 4.65 – 4.62 (m, 2H, $PhCH_2$), 4.57 (dd, J = 9.1, 4.1 Hz, 1H, H-4[a]), 4.53 – 4.48 (m, 4H, $PhCH_2$, H-3[c]), 4.36 (d, J = 12.2 Hz, 1H, $PhCH_2$), 4.27 (d, J = 5.3 Hz, 1H, H-3[a]), 4.26 – 4.24 (m, 1H, H-4[d]/H-4[e]), 4.22 (dd, J = 9.1, 4.1 Hz, 1H, H-4[b]), 4.19 – 4.16 (m, 2H, H-4[c], H-4[d]/H-4[e]), 4.13 (d, J = 5.2 Hz, 1H, H-3[b]), 3.97 – 3.93 (m, 2H, H-5[a,c]), 3.84 (dd, J = 11.4, 4.2 Hz, 1H, H-5[b]), 3.81 – 3.79 (m, 3H, H-3[d,e], H-5[c]), 3.75 (dd, J = 11.3, 4.0 Hz, 1H, H-5[a]), 3.71 – 3.67 (m, 3H, H-5[b,d,e]), 3.59 – 3.52 (m, 2H, H-5[d,e]), 2.57 (t, J = 6.5 Hz, 1H, -OH), 2.27 (s, 3H, tolyl CH_3), 2.21 (t, J = 6.3 Hz, 1H, -OH); ^{13}C NMR (150 MHz, $CDCl_3$) δ 165.62 (C=O), 165.57 (C=O), 165.43 (C=O), 165.33 (C=O), 165.17 (C=O), 137.93 (Ar), 137.79 (Ar), 137.63 (Ar), 137.62 (Ar), 137.56 (Ar), 133.61 (Ar), 133.48 (Ar), 133.39 (Ar), 132.57 (Ar), 130.71 (Ar), 130.00 (Ar), 129.98 (Ar), 129.96 (Ar), 129.89 (Ar), 129.86 (Ar), 129.82 (Ar), 129.60 (Ar), 129.57 (Ar), 129.52 (Ar), 129.49 (Ar), 128.65 (Ar), 128.64 (Ar), 128.60 (Ar), 128.57 (Ar), 128.54 (Ar), 128.50 (Ar), 128.44 (Ar), 128.42 (Ar), 128.03 (Ar), 127.95 (Ar), 127.92 (Ar), 127.86 (Ar), 127.73 (Ar), 106.33 (C-1[b]), 106.13 (×2, C-1[c,e]), 105.60 (H-1[d]), 91.52 (H-1[a]), 83.80, 83.55, 83.49, 83.34, 83.33, 83.18, 82.89 (C-2[c]), 82.44 (C-2[a]), 82.07, 82.05, 81.90 (C-2[b]), 81.77 (×2, C-2[e], C-4[a]), 81.47, 79.92 (C-3[c]), 72.56 ($PhCH_2$), 72.43 ($PhCH_2$), 72.39 ($PhCH_2$), 72.13 ($PhCH_2$), 65.76 (C-5[b]), 65.59 (C-5[a]), 64.78 (C-5[c]), 62.85 (C-5), 62.75 (C-5), 21.22 (tolyl CH_3); HRMS (ESI) Calcd for $C_{95}H_{92}O_{25}SNa$ $[M + Na]^+$: 1687.5541, found: 1687.5589.

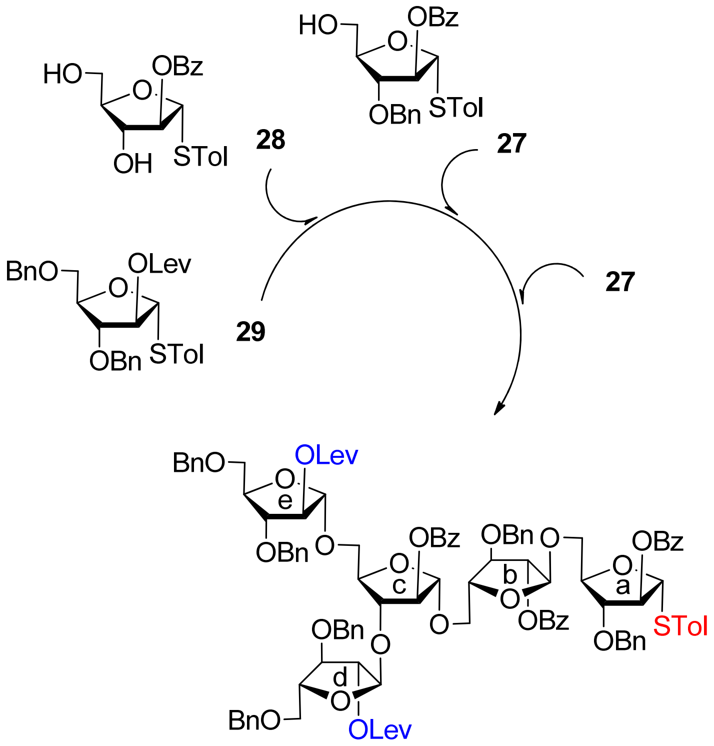

***p*-Tolyl 2-*O*-levulinoyl-3,5-di-*O*-benzyl-*α*-D-arabinofuranosyl[e]-(1→5)-(2-*O*-levulinoyl-3,5-di-*O*-benzyl-*α*-D-arabinofuranosyl[d])-(1→3)-2-*O*-benzoyl-*α*-D-arabinofuranosyl[c]-(1→5)-2-*O*-benzoyl-3-*O*-benzyl-*α*-D-arabinofuranosyl[b]-(1→5)-2-*O*-benzoyl-3-*O*-benzyl-1-thio-*α*-D-arabinofuranoside[a] (15):** A mixture of **29** (1.09 g, 2.039 mmol), TTBP (1.17 g, 4.72 mmol) and freshly activated 4 Å molecular sieves (7.0 g) in anhydrous CH_2Cl_2 (40 mL) under argon atmosphere was stirred for 20 min at room temperature, and cooled to -78 °C. 5 min later, stoichiometric *p*-TolSCl (281.3 μL, 2.039 mmol) was added, followed by dropwise addition of AgOTf (10.2 mL, 4.078 mmol, 0.4 mol/L in anhydrous toluene/CH_2Cl_2, 3:1). After another 5 min, a solution of **28** (350.0 mg, 0.971 mmol) in anhydrous CH_2Cl_2 (4 mL) was slowly added. The resulting mixture was slowly warmed to room temperature within 2 h and cooled back to -78 °C. 5 min later, stoichiometric *p*-TolSCl (134.0 μL, 0.971 mmol) was added, followed by dropwise addition of AgOTf (4.9 mL, 1.942 mmol, 0.4 mol/L in anhydrous toluene/CH_2Cl_2, 3:1). After another 5 min, a solution of **27** (415.6 mg, 0.922 mmol) in anhydrous CH_2Cl_2 (4 mL) was slowly added. The resulting mixture was stirred at -78 °C for 10 min, warmed to room temperature, and cooled back to -78 °C. 5 min later, stoichiometric *p*-TolSCl (127.2 μL, 0.922 mmol) was added, followed by dropwise addition of AgOTf (4.6 mL, 1.844 mmol, 0.4 mol/L in

anhydrous toluene/CH_2Cl_2, 3:1). After another 5 min, a solution of **27** (481.2 mg, 1.068 mmol) in anhydrous CH_2Cl_2 (4 mL) was slowly added. The resulting mixture was stirred at -78 °C for 10 min, warmed to room temperature, and quenched with Et_3N (1 mL) and filtered through Celite. After removal of the solvent, the desired product was purified by column chromatography on silica gel (petroleum ether/acetone, 3.5:1) to afford **15** (1.29 g, 76%) as white foam. R_f = 0.43 (petroleum ether/ethyl acetate, 1:1); $[\alpha]_D^{30}$ +114.5 (*c* 0.3, CH_2Cl_2); ^1H NMR (600 MHz, $CDCl_3$) δ 8.04 – 8.01 (m, 4H, Ar), 7.99 (d, *J* = 7.5 Hz, 2H, Ar), 7.54 – 7.50 (m, 3H, Ar), 7.43 – 7.33 (m, 10H, Ar), 7.29 – 7.13 (m, 28H, Ar), 7.06 (d, *J* = 7.9 Hz, 2H, Ar), 5.61 (s, 1H, H-1[a]), 5.57 (s, 1H, H-2[a]), 5.41 (s, 1H, , H-2[b]), 5.33 (d, *J* = 1.2 Hz, 1H, , H-2[c]), 5.31 (s, 1H, H-1[d]), 5.23 (s, 1H, H-1[c]), 5.21 (s, 1H, H-1[b]), 5.14 (s, 1H, H-2[e]), 5.12 (s, 1H, H-1[e]), 5.11 (s, 1H, H-2[d]), 4.76 (d, *J* = 11.9 Hz, 1H, $PhCH_2$), 4.63 – 4.59 (m, 3H, $PhCH_2$), 4.56 (dd, *J* = 8.9, 3.8 Hz, 1H, H-4[a]), 4.49 – 4.38 (m, 7H, $PhCH_2$, H-3[c]), 4.36 – 4.32 (m, 2H, $PhCH_2$), 4.27 (dd, *J* = 5.2, 1.0 Hz, 1H, H-3[a]), 4.24 (dd, *J* = 8.9, 4.7 Hz, 1H, H-4[e]), 4.20 – 4.14 (m, 3H, H-4[b,c,d]), 4.10 (d, *J* = 5.2 Hz, 1H, H-3[b]), 3.95 (dd, *J* = 11.4, 3.9 Hz, 1H, H-5[a]), 3.91 (dd, *J* = 11.6, 4.0 Hz, 1H, H-5[c]), 3.83 – 3.81 (m, 2H, H-3[d], H-5[b]), 3.78 (d, *J* = 5.3 Hz, 1H, H-3[e]), 3.74 (dd, *J* = 11.4, 4.0 Hz, 1H, H-5[a]), 3.70 (dd, *J* = 11.4, 2.3 Hz, 1H, H-5[c]), 3.65 (dd, *J* = 11.4, 3.9 Hz, 1H, H-5[b]), 3.54 – 3.51 (m, 2H, H-5[d,e]), 3.47 – 3.43 (m, 2H, H-5[d,e]), 2.67 – 2.53 (m, 4H, Lev CH_2), 2.46 – 2.35 (m, 4H, Lev CH_2), 2.28 (s, 3H, tolyl CH_3), 2.11 (s, 3H, Lev CH_3), 2.10 (s, 3H, Lev CH_3); ^{13}C NMR (150 MHz, $CDCl_3$) δ 206.26 (Lev C=O), 206.21 (Lev C=O), 171.53 (Lev C=O), 171.50 (Lev C=O), 165.57 (C=O), 165.49 (C=O), 165.43 (C=O), 138.27 (Ar), 138.20 (Ar), 137.99 (Ar), 137.94 (Ar), 137.92 (Ar), 137.78 (Ar), 137.62 (Ar), 133.47 (Ar), 133.42 (Ar), 133.32 (Ar), 132.56 (Ar), 130.72 (Ar), 130.00 (Ar), 129.98 (Ar), 129.94 (Ar), 129.82 (Ar), 129.63 (Ar), 129.52 (Ar), 128.65 (Ar), 128.63 (Ar), 128.54 (Ar), 128.42 (Ar), 128.40 (Ar), 128.37 (Ar), 128.33 (Ar), 128.05 (Ar), 128.02 (Ar), 127.94 (Ar), 127.88 (Ar), 127.81 (Ar), 127.76 (Ar), 127.73 (Ar), 127.70 (Ar), 127.64 (Ar), 127.62 (Ar), 106.29 (C-1[b]), 106.14 (C-1[e]), 106.12 (C-1[c]), 105.37 (C-1[d]), 91.50 (C-1[a]), 83.41 (C-3[b]), 83.34 (C-3[d]), 83.22 (C-3[e]), 83.15 (C-3[a]), 82.79 (C-2[c]), 82.56 (C-4[d]), 82.45 (C-2[a]), 82.32 (C-4[e]), 82.03 (C-4[b]), 81.97 (C-2[b]), 81.77, 81.75 (×2), 81.63 (C-2[e]),

80.05 (C-3[c]), 73.49 ($PhCH_2$), 73.45 ($PhCH_2$), 72.56 ($PhCH_2$), 72.41 ($PhCH_2$), 72.11 ($PhCH_2$), 71.81 ($PhCH_2$), 69.37 (C-5[e]), 69.15 (C-5[d]), 65.74 (C-5[b]), 65.56 (C-5[a]), 65.18 (C-5[c]), 37.91 (×2, Lev CH_2), 29.88 (×2, Lev CH_3), 28.02 (Lev CH_2), 27.95 (Lev CH_2), 21.22 (tolyl CH_3); HRMS (ESI) Calcd for $C_{105}H_{108}O_{27}SNa$ $[M + Na]^+$: 1855.6691, found: 1855.6703.

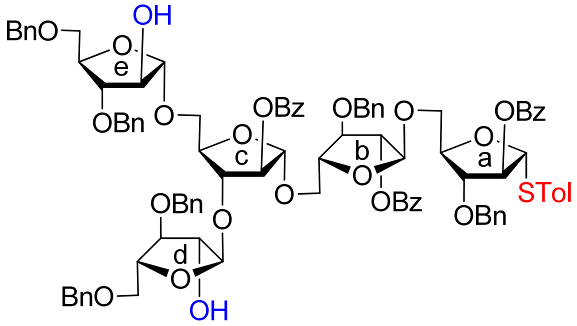

***p*-Tolyl 3,5-di-*O*-benzyl-*α*-D-arabinofuranosyl[e]-(1→5)-(3,5-di-*O*-benzyl-*α*-D-arabinofuranosyl[d])-(1→5)-2-*O*-benzoyl-*α*-D-arabinofuranosyl[c]-(1→3)-2-*O*-benzoyl-3-*O*-benzyl-*α*-D-arabinofuranosyl[b]-(1→5)-2-*O*-benzoyl-3-*O*-benzyl-1-thio-*α*-D-arabinofuranoside[a] (14):** To a solution of **15** (1.0 g, 0.545 mmol) in THF (20 mL) and CH_3OH (2 mL) was added hydrazine acetate (0.25 g, 2.725 mmol), the resulting mixture was stirred at room temperature for 5 h, quenched with acetone (1 mL) and stirred for another 10 min. After removal of the solvent, the residue was dissolved in EtOAc (50 mL), washed with a saturated aqueous $NaHCO_3$ solution (20 mL) and brine (20 mL), dried over Na_2SO_4, filtered, and concentrated. The desired product was purified by column chromatography on silica gel (petroleum ether/acetone, 3.5:1) to afford **14** (0.84 g, 94%) as white foam. R_f = 0.55 (petroleum ether/ethyl acetate, 1:1); $[\alpha]_D^{30}$ +116.8 (*c* 1.0, CH_2Cl_2); ^1H NMR (600 MHz, $CDCl_3$) δ 8.04 – 8.02 (m, 4H, Ar), 7.97 (d, *J* = 7.3 Hz, 2H, Ar), 7.55 (t, *J* = 7.4 Hz, 1H, Ar), 7.52 (td, *J* = 7.4, 1.1 Hz, 2H, Ar), 7.45 – 7.33 (m, 10H, Ar), 7.31 – 7.13 (m, 28H, Ar), 7.06 (d, *J* = 8.0 Hz, 2H, Ar), 5.61 (s, 1H, H-1[a]), 5.57 (t, *J* = 1.5 Hz, 1H, H-2[a]), 5.40 (d, *J* = 1.7 Hz, 1H, H-2[b]), 5.30 (d, *J* = 1.7 Hz, 1H, H-2[c]), 5.23 (s, 1H, H-1[c]), 5.23 (s, 1H, H-1[b]), 5.15 (s, 1H, H-2[d]), 5.05 (s, 1H, H-1[e]), 4.77 (d, *J* = 12.0 Hz, 1H, $PhCH_2$), 4.63 – 4.60 (m, 2H, $PhCH_2$), 4.57 – 4.52 (m, 3H, H-4[a], $PhCH_2$), 4.49 (d, *J* = 12.8 Hz, 1H, $PhCH_2$), 4.46 (d, *J* = 12.8

Hz, 1H, $PhCH_2$), 4.43 – 4.35 (m, 5H, H-3^c, $PhCH_2$), 4.30 (d, *J* = 12.3 Hz, 1H, $PhCH_2$), 4.25 (br s, 2H, H-4^e, H-3^a), 4.19 (dd, *J* = 9.1, 4.1 Hz, 1H, H-4^b), 4.16 (dd, *J* = 6.5, 3.0 Hz, 1H, H-4^d), 4.12 – 4.10 (m, 2H, H-2^e, H-4^c), 4.07 – 4.05 (m, 2H, H-3^b, H-2^d), 3.96 – 3.91 (m, 2H, H-5^{a,c}), 3.82 (dd, *J* = 11.3, 4.3 Hz, 1H, H-5^a), 3.78 (d, *J* = 2.8 Hz, 1H, H-3^d), 3.76 (d, *J* = 2.8 Hz, 1H, H-3^e), 3.74 (dd, *J* = 11.4, 4.0 Hz, 1H, H-5^a), 3.68 (dd, *J* = 11.5, 2.8 Hz, 1H, H-5^c), 3.63 (dd, *J* = 11.3, 3.8 Hz, 1H, H-5^b), 3.53 (dd, *J* = 10.6, 2.3 Hz, 1H, H-5^e), 3.49 (dd, *J* = 10.6, 2.5 Hz, 1H, H-5^d), 3.39 (dd, *J* = 10.6, 3.1 Hz, 1H, H-5^e), 3.36 (dd, *J* = 10.5, 2.6 Hz, 1H, H-5^d), 3.22 (d, *J* = 9.9 Hz, 1H, -OH^e), 3.00 (d, *J* = 8.9 Hz, 1H, -OH^d), 2.27 (s, 3H, tolyl CH_3); ^{13}C NMR (150 MHz, $CDCl_3$) δ 165.53 (C=O), 165.51 (C=O), 165.46 (C=O), 137.94 (Ar), 137.83 (Ar), 137.75 (Ar), 137.63 (Ar), 137.36 (Ar), 137.26 (Ar), 133.52 (Ar), 133.47 (Ar), 133.36 (Ar), 132.55 (Ar), 130.67 (Ar), 129.97 (Ar), 129.94 (Ar), 129.81 (Ar), 129.58 (Ar), 129.48 (Ar), 128.70 (Ar), 128.64 (Ar), 128.61 (Ar), 128.52 (Ar), 128.49 (Ar), 128.47 (Ar), 128.42 (Ar), 128.40 (Ar), 128.10 (Ar), 128.03 (Ar), 128.00 (Ar), 127.90 (Ar), 127.88 (Ar), 127.87 (Ar), 127.80 (Ar), 127.73 (Ar), 127.70 (Ar), 127.67 (Ar), 108.95 (C-1^e), 108.21 (C-1^d), 106.27 (C-1^b), 106.06 (C-1^c), 91.51 (C-1^a), 85.16 (C-3^d), 84.88 (C-3^e), 83.42 (C-3^b), 83.21 (C-2^c), 83.15, 83.12, 83.11, 82.41 (C-2^a), 82.08 (C-4^b), 81.90 (C-2^b), 81.82 (C-4^c), 81.73 (C-4^a), 80.71 (C-3^c), 78.59 (C-2^d), 78.29 (C-2^e), 73.78 ($PhCH_2$), 73.71 ($PhCH_2$), 72.53 ($PhCH_2$), 72.34 ($PhCH_2$), 71.91 ($PhCH_2$), 71.76 ($PhCH_2$), 69.794 (C-5^d/C-5^e), 69.786 (C-5^d/C-5^e), 65.73 (C-5^b), 65.51 (C-5^a), 64.98 (C-5^c), 21.20 (tolyl CH_3); HRMS (ESI) Calcd for $C_{95}H_{96}O_{23}SNa$ [M + Na]$^+$: 1659.5955, found: 1659.5988.

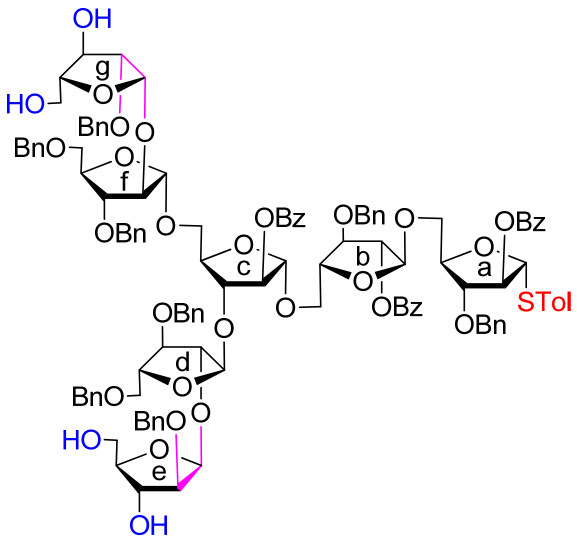

***p*-Tolyl 2-*O*-benzyl-*β*-D-arabinofuranosyl[g]-(1→2)-3,5-di-*O*-benzyl-*α*-D-arabinofuranosyl[f]-(1→5)-[2-*O*-benzyl-*β*-D-arabinofuranosyl[e]-(1→2)-3,5-di-*O*-benzyl-*α*-D-arabinofuranosyl[d]]-(1→3)-2-*O*-benzoyl-*α*-D-arabinofuranosyl[c]-(1→5)-2-*O*-benzoyl-3-*O*-benzyl-*α*-D-arabinofuranosyl[b]-(1→5)-2-*O*-benzoyl-3-*O*-benzyl-1-thio-*α*-D-arabinofuranoside[a] (31):** A mixture of **13b** (36 mg, 61.1 μmol) and freshly activated 4 Å molecular sieves (1.0 g) in anhydrous CH_2Cl_2 (8.5 mL) under argon atmosphere was stirred for 20 min at room temperature, and cooled to -78 °C. 5 min later, stoichiometric *p*-TolSCl (8.4 μL, 61.1 μmol) was added, followed by dropwise addition of AgOTf (305 μL, 122.2 μmol, 0.4 mol/L in anhydrous toluene/CH_2Cl_2, 3:1). After another 5 min, **14** (25 mg, 15.26 μmol) was added. The reaction mixture was stirred at -78 °C for 10 min, quenched with Et_3N (100 μL), warmed to room temperature, and filtered through Celite. After removal of the solvent, the residue was purified by column chromatography on silica gel (petroleum ether/ethyl acetate, 7:1) to give **30b** (*β*,*β*-isomer:other isomers = 9:1), which was dissolved in THF (3 mL), followed by the addition of TBAF (0.15 mL, 0.15 mmol, 1 M in THF). The reaction solution was stirred at room temperature for 2 h, and concentrated *in vacuo*. The residue was purified by column chromatography on silica gel (toluene/acetonitrile, 4:1) to afford **31** (23.5 mg, 74% for two steps) as white foam. R_f = 0.18 (petroleum ether/acetone, 1.5:1); $[\alpha]_D^{30}$ +47.7 (*c* 0.3, CH_2Cl_2); ^1H NMR (600 MHz, $CDCl_3$) δ 8.02 – 8.01 (m, 2H, Ar), 7.98 – 7.97 (m, 2H, Ar), 7.92 – 7.90 (m, 2H, Ar), 7.52 (t, *J* = 7.4 Hz, 1H, Ar), 7.50 – 7.46 (m, 2H, Ar), 7.42 – 7.36 (m, 6H, Ar), 7.34 – 7.18 (m, 39H,

Ar), 7.15 – 7.10 (m, 3H, Ar), 7.06 (d, *J* = 8.0 Hz, 2H, Ar), 5.60 (s, 1H, H-1^{a}), 5.56 (t, *J* = 1.9 Hz, 1H, H-2^{a}), 5.38 (d, *J* = 1.4 Hz, 1H, H-2^{b}), 5.33 (d, *J* = 1.2 Hz, 1H, H-1^{d}), 5.30 (d, *J* = 1.8 Hz, 1H, H-2^{c}), 5.26 (s, 1H, H-1^{c}), 5.20 – 5.19 (m, 2H, H-1^{b,e}), 5.07 (d, *J* = 1.4 Hz, 1H, H-1^{f}), 5.03 (d, *J* = 4.4 Hz, 1H, H-1^{g}), 4.76 (d, *J* = 11.9 Hz, 1H, $PhCH_2$), 4.67 (d, *J* = 11.7 Hz, 1H, $PhCH_2$), 4.62 – 4.53 (m, 5H, $PhCH_2$, H-4^{a}), 4.49 – 4.37 (m, 9H, $PhCH_2$), 4.35 – 4.29 (m, 5H, $PhCH_2$, H-3^{c,e,g}, H-2^{d}), 4.26 (dd, *J* = 3.7, 1.7 Hz, 1H, H-2^{f}), 4.25 (dd, *J* = 5.4, 1.3 Hz, 1H, H-3^{a}), 4.22 – 4.17 (m, 3H, H-4^{b,c,f}), 4.12 – 4.10 (m, 1H, H-4^{d}), 4.07 (dd, *J* = 6.7, 3.8 Hz, 1H, H-3^{d}), 4.03 (dd, *J* = 6.2, 4.1 Hz, 2H, H-3^{b,f}), 3.97 (dd, *J* = 11.7, 3.9 Hz, 1H, H-5^{c}), 3.92 (dd, *J* =11.3, 4.0 Hz, 1H, H-5^{a}), 3.85 – 3.70 (m, 7H, H-2^{e,g}, H-4^{e,g}, H-5^{a,c,f}), 3.65 (dd, *J* = 11.4, 4.1 Hz, 1H, H-5^{f}), 3.61 (dt, *J* = 12.4, 4.1 Hz, 1H, H-5^{e}), 3.56 – 3.43 (m, 7H, H-5^{b,d,e,g}), 2.27 (s, 3H, tolyl CH_3), 2.26 – 2.24 (m, 1H, -OH), 2.16 – 2.15 (m, 1H, -OH), 2.09 (d, *J* = 4.2 Hz, 1H, -OH), 2.08 (d, *J* = 4.2 Hz, 1H, -OH); ^{13}C NMR (150 MHz, $CDCl_3$) δ 165.75 (C=O), 165.55 (C=O), 165.39 (C=O), 138.11 (Ar), 138.09 (Ar), 137.95 (Ar), 137.90 (Ar), 137.88 (Ar), 137.80 (Ar), 137.77 (Ar), 137.73 (Ar), 137.68 (Ar), 133.50 (Ar), 133.48 (Ar), 132.59 (Ar), 130.67 (Ar), 130.00 (Ar), 129.91 (Ar), 129.89 (Ar), 129.84 (Ar), 129.57 (Ar), 129.50 (Ar), 129.46 (Ar), 128.71 (Ar), 128.63 (Ar), 128.59 (Ar), 128.54 (Ar), 128.49 (Ar), 128.48 (Ar), 128.44 (Ar), 128.38 (Ar), 128.21 (Ar), 128.11 (Ar), 128.06 (Ar), 128.03 (Ar), 127.96 (Ar), 127.88 (Ar), 127.84 (Ar), 127.80 (Ar), 127.77 (Ar), 127.72 (Ar), 106.38 (C-1^{f}), 106.34 (C-1^{b}), 106.06 (C-1^{c}), 105.82 (C-1^{d}), 99.58 (C-1^{g}), 99.12 (C-1^{e}), 91.53 (C-1^{a}), 85.98 (C-2^{f}), 85.43 (C-2^{d}), 84.30, 84.27, 83.92, 83.66, 83.33, 83.17, 82.83, 82.40 (C-2^{a}), 82.08, 82.06, 81.98, 81.93, 81.89, 81.75 (C-4^{a}), 81.38, 81.08, 81.03, 73.51 ($PhCH_2$), 73.48 ($PhCH_2$), 73.45 ($PhCH_2$), 72.61 ($PhCH_2$), 72.56 ($PhCH_2$), 72.38 ($PhCH_2$), 72.34 ($PhCH_2$), 71.95 ($PhCH_2$), 69.62 (C-5), 69.45 (C-5), 66.00 (C-5), 65.61 (C-5), 65.41 (C-5), 62.94 (C-5), 62.79 (C-5), 21.23 (tolyl CH_3); HRMS (ESI) Calcd for $C_{119}H_{124}O_{31}SNa$ [M + Na]$^+$: 2103.7740, found: 2103.7800.

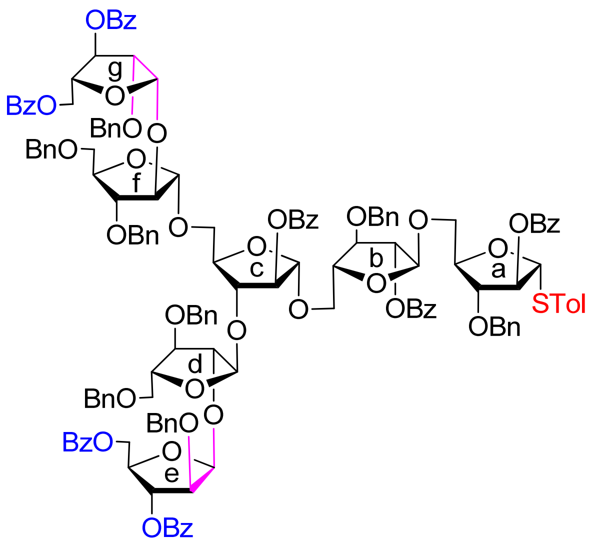

***p*-Tolyl 2-*O*-benzyl-3,5-di-*O*-benzoyl-*β*-D-arabinofuranosyl[g]-(1→2)-3,5-di-*O*-benzyl-*α*-D-arabinofuranosyl[f]-(1→5)-[2-*O*-benzyl-3,5-di-*O*-benzoyl-*β*-D-arabinofuranosyl[e]-(1→2)-3,5-di-*O*-benzyl-*α*-D-arabinofuranosyl[d]]-(1→3)-2-*O*-benzoyl-*α*-D-arabinofuranosyl[c]-(1→5)-2-*O*-benzoyl-3-*O*-benzyl-*α*-D-arabinofuranosyl[b]-(1→5)-2-*O*-benzoyl-3-*O*-benzyl-1-thio-*α*-D-arabinofuranoside[a] (10):** To a solution of **31** (500 mg, 0.24 mmol) in anhydrous CH_2Cl_2 (10 mL) was added pyridine (966 μL, 12.0 mmol), Bz_2O (272 mg, 1.2 mmol) and a catalytic amount of DMAP (2.9 mg, 0.02 mmol). The reaction mixture was refluxed overnight, quenched with CH_3OH (500 μL) and stirred for another 30 min. After removal of the solvent, the residue was dissolved in EtOAc (60 mL), washed with 0.1 N HCl (30 mL), saturated aqueous $NaHCO_3$ solution (20 mL) and brine (20 mL). The organic layer was dried over Na_2SO_4, filtered, and concentrated to give a crude residue, which was purified by column chromatography on silica gel (petroleum ether/acetone, 3:1) to afford **10** (583 mg, 97%) as white foam. R_f = 0.58 (petroleum ether/ethyl acetate, 1.5:1); $[\alpha]_D^{30}$ +22.0 (*c* 0.6, CH_2Cl_2); ^{1}H NMR (600 MHz, $CDCl_3$) δ 8.02 – 8.01 (m, 2H, Ar), 7.99 – 7.96 (m, 6H, Ar), 7.94 – 7.91 (m, 6H, Ar), 7.57 (t, *J* = 7.4 Hz, 2H, Ar), 7.51 (t, *J* = 7.4 Hz, 1H, Ar), 7.44 – 7.37 (m, 12H, Ar), 7.36 – 7.31 (m, 4H, Ar), 7.29 – 7.07 (m, 44H, Ar), 7.04 (d, *J* = 8.0 Hz, 2H, Ar), 5.61 – 5.58 (m, 3H, H-3[e,g], H-1[a]), 5.56 (t, *J* = 1.9 Hz, 1H, H-2[a]), 5.38 (d, *J* = 1.4 Hz, 1H, H-2[b]), 5.36 – 5.36 (m, 2H, H-1[d,e]), 5.34 (d, *J* = 2.0 Hz, 1H, H-2[c]), 5.26 (s, 1H, H-1[c]), 5.22 (d, *J* = 4.4 Hz, 1H, H-1[g]), 5.20 (s, 1H, H-1[b]), 5.15 (s, 1H, H-1[f]), 4.75 (d, *J* = 11.9 Hz, 1H, $PhCH_2$), 4.72 (dd, *J* = 11.7 Hz, 4.6 Hz, 1H,

H-5[e]), 4.70 – 4.66 (m, 2H, H-5[g], PhCH$_2$), 4.63 – 4.48 (m, 8H, PhCH$_2$, H-4[a]), 4.46 – 4.36 (m, 11H, PhCH$_2$, H-2[d,f], H-3[c], H-5[e,g]), 4.34 (d, *J* = 11.9 Hz, 1H, PhCH$_2$), 4.30 – 4.23 (m, 5H, H-2[e], H-3[a], H-4[e,f,g]), 4.21 – 4.16 (m, 4H, H-2[g], H-4[b,c,d]), 4.05 – 3.98 (m, 4H, H-3[b,d,f], H-5[c]), 3.93 (dd, *J* = 11.4, 4.0 Hz, 1H, H-5[a]), 3.81 (dd, *J* = 11.5, 4.6 Hz, 1H, H-5[d]), 3.74 (dd, *J* = 11.6, 2.0 Hz, 1H, H-5[c]), 3.71 (dd, *J* = 11.4, 4.1 Hz, 1H, H-5[a]), 3.64 (dd, *J* = 11.5, 4.0 Hz, 1H, H-5[d]), 3.54 – 3.46 (m, 4H, H-5[b,f]), 2.26 (s, 3H, tolyl CH$_3$); ^{13}C NMR (150 MHz, CDCl$_3$) δ 166.19 (C=O), 166.16 (C=O), 165.86 (C=O), 165.79 (C=O), 165.77 (C=O), 165.53 (C=O), 165.36 (C=O), 138.25 (Ar), 138.22 (Ar), 138.06 (Ar), 137.98 (Ar), 137.75 (Ar), 137.71 (Ar), 137.63 (Ar), 137.52 (Ar), 133.54 (Ar), 133.49 (Ar), 133.47 (Ar), 133.44 (Ar), 133.05 (Ar), 133.01 (Ar), 132.53 (Ar), 130.70 (Ar), 129.98 (Ar), 129.95 (Ar), 129.92 (Ar), 129.87 (Ar), 129.82 (Ar), 129.55 (Ar), 129.50 (Ar), 129.46 (Ar), 128.61 (Ar), 128.59 (Ar), 128.56 (Ar), 128.55 (Ar), 128.53 (Ar), 128.49 (Ar), 128.42 (Ar), 128.39 (Ar), 128.36 (Ar), 128.32 (Ar), 128.26 (Ar), 128.04 (Ar), 127.99 (Ar), 127.89 (Ar), 127.88 (Ar), 127.84 (Ar), 127.80 (Ar), 127.78 (Ar), 127.66 (Ar), 127.61 (Ar), 127.49 (Ar), 106.55 (C-1[f]), 106.33 (C-1[b]), 106.14 (C-1[c]), 106.06 (C-1[d]), 100.98 (C-1[g]), 100.62 (C-1[e]), 91.51 (C-1[a]), 86.94 (C-2[f]), 86.12 (C-2[d]), 84.43 (C-3[d]), 84.06 (×2, C-2[c], C-3[f]), 83.53 (C-3[b]), 83.13, 82.42 (C-2[a]), 82.00, 81.96, 81.93, 81.86, 81.73, 81.61, 81.54, 81.48, 81.25 (C-3[c]), 79.26 (C-4[e]), 79.22 (C-4[g]), 77.88 (C-3[e]), 77.81 (C-3[g]), 73.42 (PhCH$_2$), 73.38 (PhCH$_2$), 72.54 (PhCH$_2$), 72.49 (PhCH$_2$), 72.41 (PhCH$_2$), 72.38 (PhCH$_2$), 72.35 (PhCH$_2$), 72.18 (PhCH$_2$), 69.93 (C-5[f]), 69.81 (C-5[b]), 66.58 (C-5[e]), 66.47 (C-5[g]), 65.95 (C-5[d]), 65.56 (C-5[a]), 65.30 (C-5[c]), 21.21 (tolyl CH$_3$); HRMS (ESI) Calcd for $C_{147}H_{140}O_{35}SNa_2$ [M + 2Na]$^{2+}$: 1271.4340, found: 1271.4376.

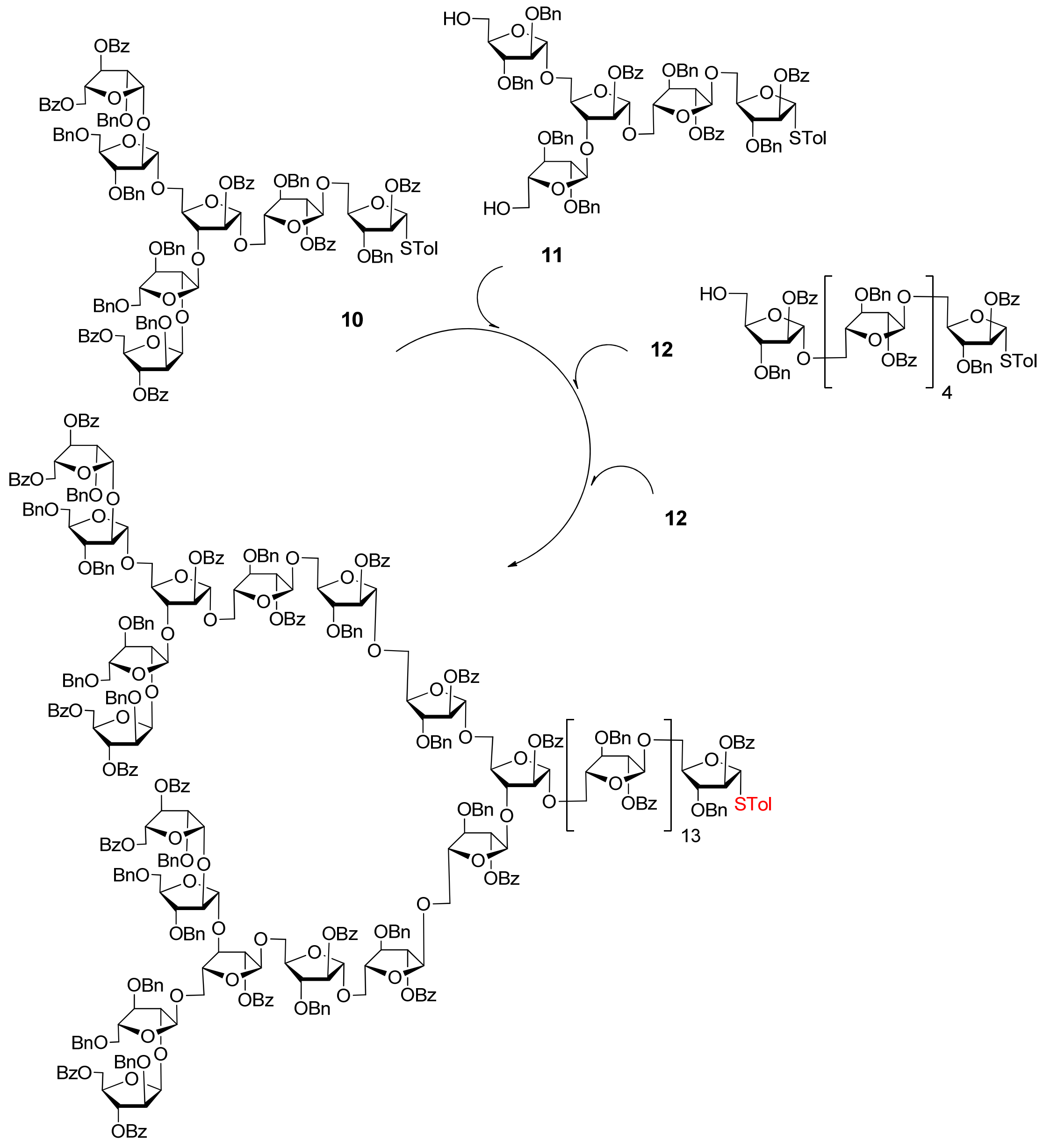

***p*-Tolyl 2-*O*-benzyl-3,5-di-*O*-benzoyl-*β*-D-arabinofuranosyl-(1→2)-3,5-di-*O*-benzyl-*α*-D-arabinofuranosyl-(1→5)-[2-*O*-benzyl-3,5-di-*O*-benzoyl-*β*-D-arabinofuranosyl-(1→2)-3,5-di-*O*-benzyl-*α*-D-arabinofuranosyl]-(1→3)-2-*O*-benzoyl-*α*-D-arabinofuranosyl-(1→5)-2-*O*-benzoyl-3-*O*-benzyl-*α*-D-arabinofuranosyl-(1→5)-2-*O*-benzoyl-3-*O*-benzyl-*α*-D-arabinofuranosyl-(1→5)-2-*O*-benzoyl-3-*O*-benzyl-*α*-D-arabinofuranosyl-(1→5)-{2-*O*-benzyl-3,5-di-*O*-benzoyl-*β*-D-arabinofuranosyl-(1→2)-3,5-di-*O*-benzyl-*α*-D-arabinofuranosyl-(1→5)-[2-*O*-benzyl-3,5-di-*O*-benzoyl-*β*-D-arabinofuranosyl-(1→2)-3,5-di-*O*-benzyl-*α*-D-arabinofuranosyl]-(1→3)-2-*O*-benzoyl-*α*-D-arabinofuranosyl-(1→5)-2-*O*-benzoyl-3-*O*-benzyl-*α*-D-arabinofuranosyl-(1→5)-2-*O*-benzoyl-3-*O*-benzyl-*α*-D-arabinofuranosyl-(1→5)-2-*O*-benzoyl-3-**

***O*-benzyl-*α*-D-arabinofuranosyl}-(1→3)-2-*O*-benzoyl-*α*-D-arabinofuranosyl-(1→5)-2-*O*-benzoyl-3-*O*-benzyl-*α*-D-arabinofuranosyl-(1→5)-2-*O*-benzoyl-3-*O*-benzyl-*α*-D-arabinofuranosyl-(1→5)-2-*O*-benzoyl-3-*O*-benzyl-*α*-D-arabinofuranosyl-(1→5)-2-*O*-benzoyl-3-*O*-benzyl-*α*-D-arabinofuranosyl-(1→5)-2-*O*-benzoyl-3-*O*-benzyl-*α*-D-arabinofuranosyl-(1→5)-2-*O*-benzoyl-3-*O*-benzyl-*α*-D-arabinofuranosyl-(1→5)-2-*O*-benzoyl-3-*O*-benzyl-*α*-D-arabinofuranosyl-(1→5)-2-*O*-benzoyl-3-*O*-benzyl-*α*-D-arabinofuranosyl-(1→5)-2-*O*-benzoyl-3-*O*-benzyl-*α*-D-arabinofuranosyl-(1→5)-2-*O*-benzoyl-3-*O*-benzyl-*α*-D-arabinofuranosyl-(1→5)-2-*O*-benzoyl-3-*O*-benzyl-*α*-D-arabinofuranosyl-(1→5)-2-*O*-benzoyl-3-*O*-benzyl-*α*-D-arabinofuranosyl-(1→5)-2-*O*-benzoyl-3-*O*-benzyl-*α*-D-arabinofuranoside (3):** A mixture of **10** (68.96 mg, 27.60 μmol), TTBP (15 mg, 60.2 μmol) and freshly activated 4 Å molecular sieves (700 mg) in anhydrous CH_2Cl_2 (3 mL) under argon atmosphere was stirred for 20 min at room temperature, and cooled to -78 °C. 5 min later, stoichiometric *p*-TolSCl (95.2 μL, 27.60 μmol, 4% in anhydrous toluene) was added, followed by dropwise addition of AgOTf (207 μL, 82.8 μmol, 0.4 mol/L in anhydrous toluene/CH_2Cl_2, 3:1). After another 5 min, a solution of **11** (19.99 mg, 12.00 μmol) in anhydrous CH_2Cl_2 (0.6 mL) was slowly added. The resulting mixture was slowly warmed to room temperature within 2 h and cooled back to -78 °C. 5 min later, stoichiometric *p*-TolSCl (41.4 μL, 12.00 μmol, 4% in anhydrous toluene) was added, followed by dropwise addition of AgOTf (60 μL, 24.0 μmol, 0.4 mol/L in anhydrous toluene/CH_2Cl_2, 3:1). After another 5 min, a solution of **12** (21.99 mg, 10.56 μmol) in anhydrous CH_2Cl_2 (0.6 mL) was slowly added. The resulting mixture was slowly warmed to room temperature within 2 h and cooled back to -78 °C. 5 min later, stoichiometric *p*-TolSCl (36.4 μL, 10.56 μmol, 4% in anhydrous toluene) was added, followed by dropwise addition of AgOTf (53 μL, 21.12 μmol, 0.4 mol/L in anhydrous toluene/CH_2Cl_2, 3:1). After another 5 min, a solution of **12** (25.06 mg, 12.00 μmol) in anhydrous CH_2Cl_2 (0.6 mL) was slowly added. The resulting mixture was slowly warmed to room temperature within 2 h, quenched with Et_3N (100 μL) and filtered through Celite. After removal of the solvent, the desired product was purified by column chromatography on silica gel (petroleum

ether/acetone, 1.7:1) to afford **3** (76.8 mg, 70%) as white foam. R_f = 0.36 (petroleum ether/acetone, 1.3:1); $[\alpha]_D^{30}$ +69.8 (*c* 0.6, CH_2Cl_2); ^{1}H NMR (600 MHz, $CDCl_3$) δ 8.03 – 7.89 (m, 62H, Ar), 7.57 – 7.06 (m, 257H, Ar), 5.614 (s, 1H, H-1α), 5.608 – 5.57 (m, 4H, H-3), 5.57 (t, *J* = 2.0 Hz, 1H, H-2), 5.45 (s, 1H, H-1α), 5.40 (d, *J* = 1.1 Hz, 2H, H-2), 5.38 (br s, 2H, H-2), 5.36 – 5.29 (m, 22H, H-1α ×2, H-1β ×2, H-2 ×18), 5.28 (s, 1H, H-1α), 5.24 (s, 1H, H-1α), 5.23 (s, 1H, H-1α), 5.22 (s, 1H, H-1α), 5.22 – 5.19 (m, 15H, H-1α ×13, H-1β ×2), 5.16 (s, 1H, H-1α), 5.14 (br s, 3H, H-1α), 5.12 (s, 1H, H-1α), 5.11 (s, 1H, H-1α), 4.77 (d, *J* = 11.8 Hz, 1H, $PhCH_2$), 4.72 – 3.45 (m, 191H), 5.27 (s, 3H, tolyl CH_3); ^{13}C NMR (150 MHz, $CDCl_3$) δ 166.17 (C=O), 166.14 (C=O), 165.84 (C=O), 165.77 (C=O), 165.74 (C=O), 165.55 (C=O), 165.44 (C=O), 165.42 (C=O), 165.38 (C=O), 165.36 (C=O), 165.29 (C=O), 165.26 (C=O), 165.24 (C=O), 165.20 (C=O), 138.24 (Ar), 138.21 (Ar), 138.05 (Ar), 138.03 (Ar), 138.00 (Ar), 137.98 (Ar), 137.96 (Ar), 137.92 (Ar), 137.75 (Ar), 137.68 (Ar), 137.62 (Ar), 137.50 (Ar), 133.54 (Ar), 133.50 (Ar), 133.48 (Ar), 133.39 (Ar), 133.32 (Ar), 133.27 (Ar), 133.23 (Ar), 133.20 (Ar), 133.04 (Ar), 133.00 (Ar), 132.56 (Ar), 130.70 (Ar), 129.98 (Ar), 129.92 (Ar), 129.85 (Ar), 129.82 (Ar), 129.71 (Ar), 129.69 (Ar), 129.66 (Ar), 129.63 (Ar), 129.58 (Ar), 129.51 (Ar), 129.49 (Ar), 129.45 (Ar), 129.44 (Ar), 128.62 (Ar), 128.58 (Ar), 128.54 (Ar), 128.48 (Ar), 128.42 (Ar), 128.36 (Ar), 128.34 (Ar), 128.30 (Ar), 128.25 (Ar), 128.02 (Ar), 128.01 (Ar), 127.88 (Ar), 127.85 (Ar), 127.82 (Ar), 127.79 (Ar), 127.76 (Ar), 127.74 (Ar), 127.71 (Ar), 127.70 (Ar), 127.66 (Ar), 127.63 (Ar), 127.60 (Ar), 127.56 (Ar), 127.48 (Ar), 106.52 (C-1), 106.47 (C-1), 106.25 (C-1), 106.15 (C-1), 106.10 (C-1), 105.37 (C-1), 100.96 (×2, C-1β), 100.60 (×2, C-1β), 91.50 (C-1), 86.92, 86.07, 84.4, 84.12, 84.04, 83.35, 83.28, 83.23, 83.17, 83.12, 83.05, 82.94, 82.50, 82.45, 82.18, 82.12, 82.08, 82.03, 81.97, 81.91, 81.81, 81.75, 81.72, 81.69, 81.59, 81.58, 81.48, 81.24, 80.16, 79.24, 79.20, 77.88, 77.79, 73.41, 73.37, 72.54, 72.47, 72.37, 72.35, 72.30, 72.26, 72.16, 72.12, 72.05, 72.03, 71.90, 69.92, 69.82, 66.54, 66.45, 65.81, 65.79, 65.69, 65.55, 65.47, 65.43, 65.25, 65.14, 65.12, 65.09, 21.21 (tolyl CH_3); MALDI-TOF MS Calcd for $C_{603}H_{572}O_{155}SNa$ $[M + Na]^+$ *m/z*: 10354.0, found: 10352.4.

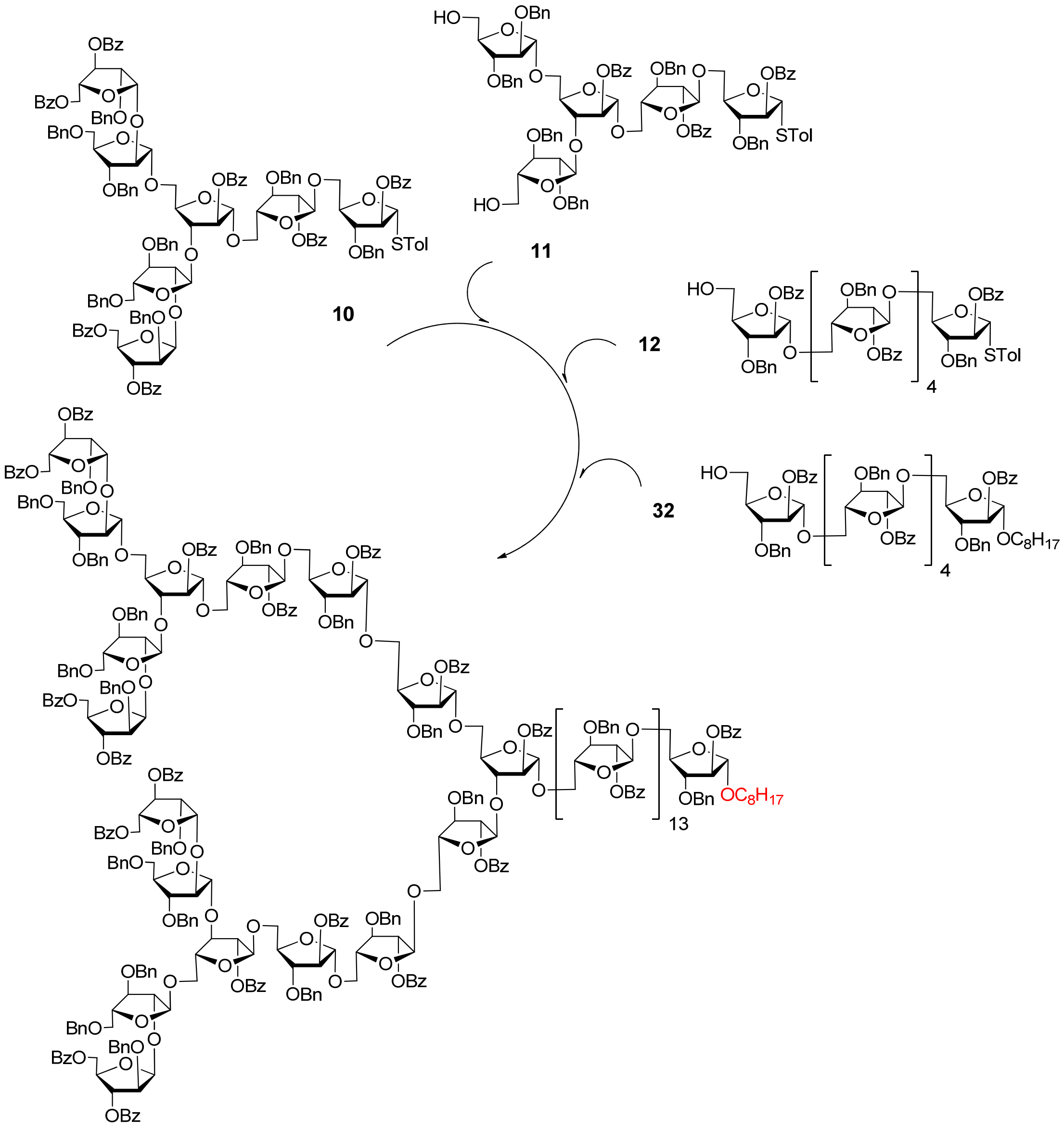

Octyl 2-*O*-benzyl-3,5-di-*O*-benzoyl-*β*-D-arabinofuranosyl-(1→2)-3,5-di-*O*-benzyl-*α*-D-arabinofuranosyl-(1→5)-[2-*O*-benzyl-3,5-di-*O*-benzoyl-*β*-D-arabinofuranosyl-(1→2)-3,5-di-*O*-benzyl-*α*-D-arabinofuranosyl]-(1→3)-2-*O*-benzoyl-*α*-D-arabinofuranosyl-(1→5)-2-*O*-benzoyl-3-*O*-benzyl-*α*-D-arabinofuranosyl-(1→5)-2-*O*-benzoyl-3-*O*-benzyl-*α*-D-arabinofuranosyl-(1→5)-2-*O*-benzoyl-3-*O*-benzyl-*α*-D-arabinofuranosyl-(1→5)-{2-*O*-benzyl-3,5-di-*O*-benzoyl-*β*-D-arabinofuranosyl-(1→2)-3,5-di-*O*-benzyl-*α*-D-arabinofuranosyl-(1→5)-[2-*O*-benzyl-3,5-di-*O*-benzoyl-*β*-D-arabinofuranosyl-(1→2)-3,5-di-*O*-benzyl-*α*-D-arabinofuranosyl]-(1→3)-2-*O*-benzoyl-*α*-D-arabinofuranosyl-(1→5)-2-*O*-benzoyl-3-*O*-benzyl-*α*-D-arabinofuranosyl-(1→5)-2-*O*-benzoyl-3-*O*-benzyl-*α*-D-arabinofuranosyl-(1→5)-2-*O*-benzoyl-3-

***O*-benzyl-*α*-D-arabinofuranosyl}-(1→3)-2-*O*-benzoyl-*α*-D-arabinofuranosyl-(1→5)-2-*O*-benzoyl-3-*O*-benzyl-*α*-D-arabinofuranosyl-(1→5)-2-*O*-benzoyl-3-*O*-benzyl-*α*-D-arabinofuranosyl-(1→5)-2-*O*-benzoyl-3-*O*-benzyl-*α*-D-arabinofuranosyl-(1→5)-2-*O*-benzoyl-3-*O*-benzyl-*α*-D-arabinofuranosyl-(1→5)-2-*O*-benzoyl-3-*O*-benzyl-*α*-D-arabinofuranosyl-(1→5)-2-*O*-benzoyl-3-*O*-benzyl-*α*-D-arabinofuranosyl-(1→5)-2-*O*-benzoyl-3-*O*-benzyl-*α*-D-arabinofuranosyl-(1→5)-2-*O*-benzoyl-3-*O*-benzyl-*α*-D-arabinofuranosyl-(1→5)-2-*O*-benzoyl-3-*O*-benzyl-*α*-D-arabinofuranosyl-(1→5)-2-*O*-benzoyl-3-*O*-benzyl-*α*-D-arabinofuranosyl-(1→5)-2-*O*-benzoyl-3-*O*-benzyl-*α*-D-arabinofuranosyl-(1→5)-2-*O*-benzoyl-3-*O*-benzyl-*α*-D-arabinofuranosyl-(1→5)-2-*O*-benzoyl-3-*O*-benzyl-*α*-D-arabinofuranoside (33):** A mixture of **10** (68.96 mg, 27.60 μmol), TTBP (15 mg, 60.2 μmol) and freshly activated 4 Å molecular sieves (700 mg) in anhydrous CH_2Cl_2 (3 mL) under argon atmosphere was stirred for 20 min at room temperature, and cooled to -78 °C. 5 min later, stoichiometric *p*-TolSCl (95.2 μL, 27.60 μmol, 4% in anhydrous toluene) was added, followed by dropwise addition of AgOTf (207 μL, 82.8 μmol, 0.4 mol/L in anhydrous toluene/CH_2Cl_2, 3:1). After another 5 min, a solution of **11** (19.99 mg, 12.00 μmol) in anhydrous CH_2Cl_2 (0.6 mL) was slowly added. The resulting mixture was slowly warmed to room temperature within 2 h and cooled back to -78 °C. 5 min later, stoichiometric *p*-TolSCl (41.4 μL, 12.00 μmol, 4% in anhydrous toluene) was added, followed by dropwise addition of AgOTf (60 μL, 24.0 μmol, 0.4 mol/L in anhydrous toluene/CH_2Cl_2, 3:1). After another 5 min, a solution of **12** (21.99 mg, 10.56 μmol) in anhydrous CH_2Cl_2 (0.6 mL) was slowly added. The resulting mixture was slowly warmed to room temperature within 2 h and cooled back to -78 °C. 5 min later, stoichiometric *p*-TolSCl (36.4 μL, 10.56 μmol, 4% in anhydrous toluene) was added, followed by dropwise addition of AgOTf (53 μL, 21.12 μmol, 0.4 mol/L in anhydrous toluene/CH_2Cl_2, 3:1). After another 5 min, a solution of **32** (24.99 mg, 12.00 μmol) in anhydrous CH_2Cl_2 (0.6 mL) was slowly added. The resulting mixture was slowly warmed to room temperature within 2 h, quenched with Et_3N (100 μL) and filtered through Celite. After removal of the solvent, the desired product was purified by column chromatography on silica gel (petroleum

ether/acetone, 1.8:1) to give a crude product, which was further purified by size exclusion chromatography (Bio-Beads S-X1, toluene/ethyl acetate, 1:1) to afford **33** (71.4 mg, 65%) as white foam. R_f = 0.39 (petroleum ether/acetone, 1.3:1); $[\alpha]_D^{30}$ +64.5 (*c* 0.7, CH_2Cl_2); ^{1}H NMR (600 MHz, $CDCl_3$) δ 8.04 – 7.89 (m, 62H, Ar), 7.57 – 7.06 (m, 253H, Ar), 5.61 – 5.57 (m, 4H, H-3), 5.45 (s, 1H, H-1α), 5.42 (d, *J* = 1.3 Hz, 1H, H-2), 5.40 (s, 1H, H-2), 5.38 – 5.37 (m, 3H, H-2), 5.36 – 5.29 (m, 22H, H-1α ×2, H-1β ×2, H-2 ×18), 5.28 (s, 1H, H-1α), 5.26 (s, 1H, H-1α), 5.23 (br s, 2H, H-1α), 5.21 – 5.19 (m, 15H, H-1α ×13, H-1β ×2), 5.16 (s, 1H, H-1α), 5.14 (br s, 3H, H-1α), 5.13 (s, 1H, H-1α), 5.12 (s, 1H, H-1α), 5.10 (s, 1H, H-1α), 4.77 (d, *J* = 12.1 Hz, 1H, $PhCH_2$), 4.71 – 3.43 (m, 193H), 1.63 – 1.58 (m, 2H, octyl CH_2), 1.34 – 1.25 (m, 10H, octyl CH_2), 0.86 (t, *J* = 6.9 Hz, 3H, octyl CH_3); ^{13}C NMR (150 MHz, $CDCl_3$) δ 166.17 (C=O), 166.15 (C=O), 165.85 (C=O), 165.78 (C=O), 165.75 (C=O), 165.67 (C=O), 165.43 (C=O), 165.39 (C=O), 165.36 (C=O), 165.30, 165.27 (C=O), 165.25 (C=O), 165.21 (C=O), 138.25 (Ar), 138.22 (Ar), 138.06 (Ar), 138.04 (Ar), 138.01 (Ar), 137.99 (Ar), 137.97 (Ar), 137.96 (Ar), 137.93 (Ar), 137.76 (Ar), 137.69 (Ar), 137.51 (Ar), 133.54 (Ar), 133.50 (Ar), 133.39 (Ar), 133.32 (Ar), 133.28 (Ar), 133.24 (Ar), 133.21 (Ar), 133.05 (Ar), 133.00 (Ar), 130.00 (Ar), 129.93 (Ar), 129.86 (Ar), 129.72 (Ar), 129.64 (Ar), 129.59 (Ar), 129.56 (Ar), 129.50 (Ar), 129.46 (Ar), 129.45 (Ar), 128.63 (Ar), 128.59 (Ar), 128.57 (Ar), 128.55 (Ar), 128.49 (Ar), 128.46 (Ar), 128.43 (Ar), 128.37 (Ar), 128.34 (Ar), 128.31 (Ar), 128.25 (Ar), 128.03 (Ar), 127.99 (Ar), 127.89 (Ar), 127.86 (Ar), 127.84 (Ar), 127.80 (Ar), 127.77 (Ar), 127.71 (Ar), 127.67 (Ar), 127.64 (Ar), 127.60 (Ar), 127.56 (Ar), 127.48 (Ar), 106.53 (C-1), 106.48 (C-1), 106.26 (C-1), 106.16 (C-1), 106.11 (C-1), 106.08 (C-1), 105.38 (C-1), 100.97 (×2, C-1β), 100.61 (×2, C-1β), 86.93, 86.08, 84.46, 84.12, 84.05, 83.44, 83.35, 83.33, 83.30, 83.22, 83.17, 83.06, 82.95, 82.51, 82.43, 82.36, 82.24, 82.19, 82.13, 82.08, 82.04, 81.97, 81.92, 81.82, 81.73, 81.69, 81.60, 81.59, 81.48, 81.25, 80.17, 79.25, 79.21, 77.89, 77.80, 73.42, 73.38, 72.47, 72.38, 72.36, 72.27, 72.17, 72.13, 72.06, 72.04, 71.91, 69.93, 69.83, 67.73 (octyl OCH_2), 66.55, 66.46, 65.82, 65.79, 65.71, 65.56, 65.52, 65.43, 65.25, 65.16, 65.13, 31.97 (octyl CH_2), 29.58 (octyl CH_2), 29.53 (octyl $OCH_2\underline{C}H_2$), 29.39 (octyl CH_2), 26.20 (octyl CH_2), 22.79 (octyl CH_2), 14.25

(octyl CH_3); MALDI-TOF MS Calcd for $C_{604}H_{582}O_{156}Na$ [M + Na]$^+$ *m/z*: 10360.0, found: 10357.5.

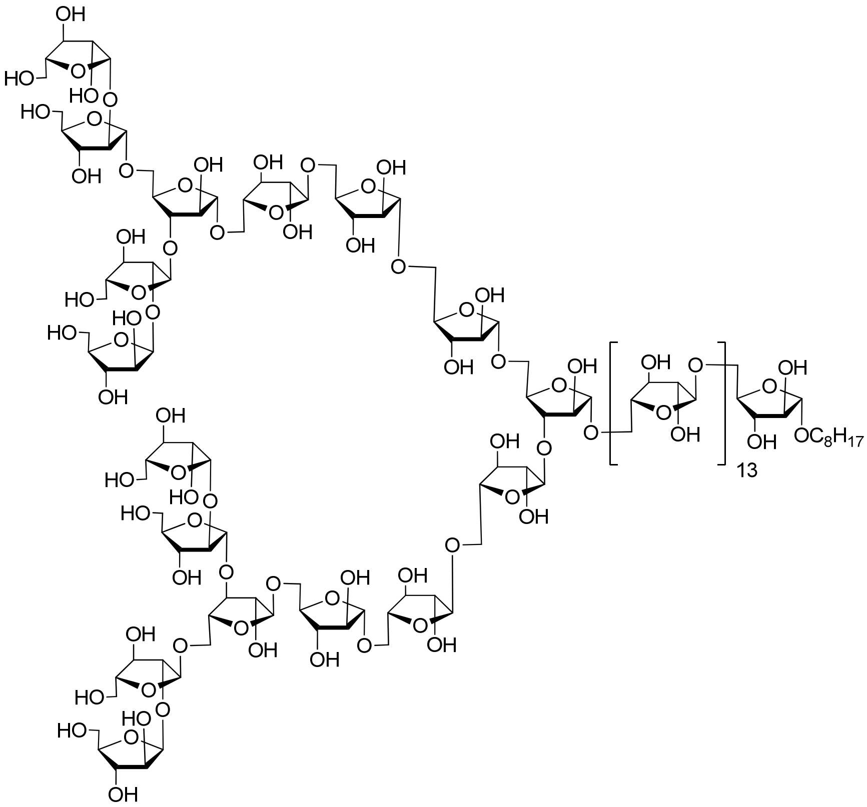

Octyl *β*-D-arabinofuranosyl-(1→2)-*α*-D-arabinofuranosyl-(1→5)-[*β*-D-arabinofuranosyl-(1→2)-*α*-D-arabinofuranosyl]-(1→3)-*α*-D-arabinofuranosyl-(1→5)-*α*-D-arabinofuranosyl-(1→5)-*α*-D-arabinofuranosyl-(1→5)-*α*-D-arabinofuranosyl-(1→5)-{*β*-D-arabinofuranosyl-(1→2)-*α*-D-arabinofuranosyl-(1→5)-[*β*-D-arabinofuranosyl-(1→2)-*α*-D-arabinofuranosyl]-(1→3)-*α*-D-arabinofuranosyl-(1→5)-*α*-D-arabinofuranosyl-(1→5)-*α*-D-arabinofuranosyl-(1→5)-*α*-D-arabinofuranosyl}-(1→3)-*α*-D-arabinofuranosyl-(1→5)-*α*-D-arabinofuranosyl-(1→5)-*α*-D-arabinofuranosyl-(1→5)-*α*-D-arabinofuranosyl-(1→5)-*α*-D-arabinofuranosyl-(1→5)-*α*-D-arabinofuranosyl-(1→5)-*α*-D-arabinofuranosyl-(1→5)-*α*-D-arabinofuranosyl-(1→5)-*α*-D-arabinofuranosyl-(1→5)-*α*-D-arabinofuranosyl-(1→5)-*α*-D-arabinofuranosyl-(1→5)-*α*-D-arabinofuranosyl-(1→5)-*α*-D-arabinofuranosyl-(1→5)-*α*-D-arabinofuranosyl-(1→5)-*α*-D-arabinofuranoside (34): To a solution of **33** (35.0 mg, 3.39 μmol) in CH_2Cl_2 (3 mL) and CH_3OH (6 mL) was added CH_3ONa (5.0 M in

CH_3OH) to adjust pH to ~10, the reaction mixture was stirred for 24 h at room temperature, and neutralized by the addition of Dowex®50WX4 ion-exchange resin. The solution was filtered and concentrated. Purification by size exclusion chromatography (Bio-Beads S-X1, ethyl acetate as eluent) gave a residue, which was dissolved in a mixture of EtOAc/THF/1-PrOH/H_2O (9 mL, 2:1:1:1) with Pd/C (150 mg, 10% Pd content). The resulting solution was stirred under an atmosphere of hydrogen (0.4 MPa) for 40 h at room temperature, filtered through Celite, and concentrated to give a crude product, which was purified by gel filtration (Sephadex LH-20, CH_3OH/H_2O, 1:1) to afford **34** (11.9 mg, 83% for two steps) as glassy solid. ^1H NMR (600 MHz, D_2O) δ 5.24 (d, *J* = 1.5 Hz, 2H, H-1α), 5.17 – 5.16 (m, 3H, H-1α), 5.140 (d, *J* = 4.5 Hz, 2H, H-1β), 5.136 (d, *J* = 4.5 Hz, 2H, H-1β), 5.11 (br s, 3H, H-1α), 5.08 (br s, 18H, H-1α), 5.01 (d, *J* = 1.8 Hz, 1H, H-1α), 4.31 –3.69 (m, 156H), 3.57 (dt, *J* = 9.9, 6.5 Hz, 1H, octyl OCH_2), 1.61 – 1.57 (m, 2H, octyl CH_2), 1.34 – 1.24 (m, 10H, octyl CH_2), 0.86 (t, *J* = 6.8 Hz, 3H, octyl CH_3); ^{13}C NMR (150 MHz, D_2O) δ 108.34, 108.29, 108.24, 108.04, 107.94, 106.48, 106.33, 101.59 (×2, C-1β), 101.46 (×2, C-1β), 87.92, 87.64, 83.73, 83.66, 83.33, 83.23, 83.17, 83.03, 83.00, 82.84, 82.82, 82.59, 82.51, 82.49, 81.93, 81.68, 81.64, 79.94, 79.86, 77.62, 77.56, 77.40, 77.31, 77.09, 75.66, 75.57, 74.99, 74.94, 69.45, 67.70, 67.62, 67.44, 67.25, 67.10, 67.02, 63.80, 63.76, 61.42, 61.40, 31.92 (octyl CH_2), 29.43 (octyl CH_2), 29.22 (octyl CH_2), 29.17 (octyl CH_2), 25.99 (octyl CH_2), 22.84 (octyl CH_2), 14.26 (octyl CH_3); MALDI-TOF MS Calcd for $C_{163}H_{266}O_{125}Na$ $[M + Na]^+$ monoisotopic *m/z*: 4246.4, found: 4246.3, for $C_{163}H_{266}O_{125}K$ $[M + K]^+$ monoisotopic *m/z*: 4262.4, found: 4262.2.

Assembly of arabinogalactan 1:

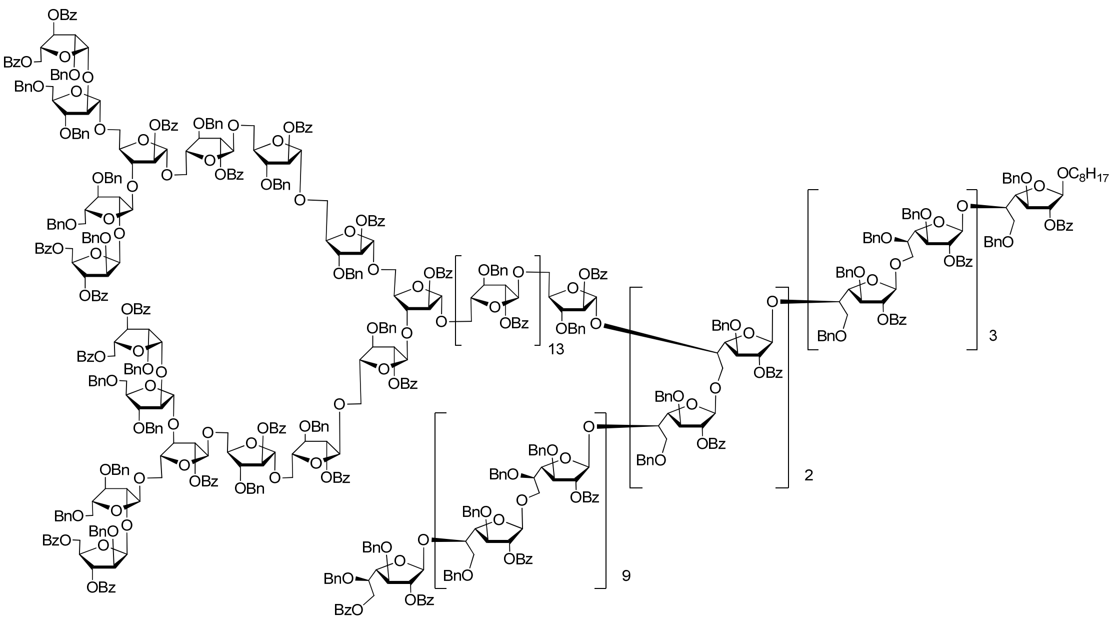

Fully protected 92mer (35): A mixture of **3** (76.8 mg, 7.45 μmol), **2** (20.0 mg, 1.49 μmol), BSM (2.4 mg, 11.18 μmol) and freshly activated 4 Å molecular sieves (200 mg) in anhydrous CH_2Cl_2 (2 mL) under argon atmosphere was stirred for 20 min at room temperature, and cooled to -40 °C. 5 min later, Tf_2O (1.9 μL, 11.18 μmol) was added. The resulting mixture was stirred at -40 °C for 10 h, quenched with Et_3N (20 μL) and filtered through Celite. After removal of the solvent, the desired product was purified by column chromatography on silica gel (petroleum ether/acetone, 1.3:1) to afford **35** (42.5 mg, 84%) as white foam. R_f = 0.24 (petroleum ether/acetone, 1.2:1); $[\alpha]_D^{30}$ +4.6 (*c* 0.3, CH_2Cl_2); ^{1}H NMR (600 MHz, $CDCl_3$) δ 8.01 – 7.82 (m, 186H, Ar), 7.57 – 6.93 (m, 889H, Ar), 5.62 (s, 1H), 5.61 – 5.45 (m, 39H), 5.40 – 5.01 (m, 135H), 4.98 (s, 1H), 4.72 – 3.47 (m, 651H), 3.41 (dt, *J* = 9.6, 6.6 Hz, 1H, octyl OCH_2), 1.58 – 1.54 (m, 2H, octyl CH_2), 1.26 – 1.23 (m, 10H, octyl CH_2), 0.85 (t, *J* = 6.8 Hz, 3H, octyl CH_3); ^{13}C NMR (150 MHz, $CDCl_3$) δ 166.19 (C=O), 166.16 (C=O), 165.85 (C=O), 165.79 (C=O), 165.75 (C=O), 165.68 (C=O), 165.55 (C=O), 165.50 (C=O), 165.45 (C=O), 165.43 (C=O), 165.42 (C=O), 165.39 (C=O), 165.37 (C=O), 165.33 (C=O), 165.31 (C=O), 165.28 (C=O), 165.25 (C=O), 165.22 (C=O), 138.51 (Ar), 138.48 (Ar), 138.45 (Ar), 138.43 (Ar), 138.25 (Ar), 138.21 (Ar), 138.17 (Ar), 138.16 (Ar), 138.14 (Ar), 138.09 (Ar), 138.05 (Ar), 138.04 (Ar), 137.99 (Ar), 137.97 (Ar), 137.93 (Ar), 137.88 (Ar), 137.87 (Ar), 137.80 (Ar), 137.76 (Ar), 137.69 (Ar), 137.63

(Ar), 137.51 (Ar), 133.55 (Ar), 133.51 (Ar), 133.40 (Ar), 133.35 (Ar), 133.24 (Ar), 133.05 (Ar), 133.01 (Ar), 129.93 (Ar), 129.86 (Ar), 129.70 (Ar), 129.64 (Ar), 129.59 (Ar), 129.57 (Ar), 129.52 (Ar), 129.51 (Ar), 129.46 (Ar), 129.45 (Ar), 128.63 (Ar), 128.59 (Ar), 128.57 (Ar), 128.49 (Ar), 128.42 (Ar), 128.37 (Ar), 128.30 (Ar), 128.25 (Ar), 128.20 (Ar), 128.15 (Ar), 128.12 (Ar), 128.09 (Ar), 128.06 (Ar), 128.04 (Ar), 127.94 (Ar), 127.90 (Ar), 127.87 (Ar), 127.84 (Ar), 127.79 (Ar), 127.77 (Ar), 127.75 (Ar), 127.71 (Ar), 127.67 (Ar), 127.61 (Ar), 127.56 (Ar), 127.53 (Ar), 127.49 (Ar), 127.45 (Ar), 127.40, (Ar) 107.04 (C-1), 106.92 (C-1), 106.74 (C-1), 106.63 (C-1), 106.58 (C-1), 106.53 (C-1), 106.48 (C-1), 106.26 (C-1), 106.16 (C-1), 106.11 (C-1), 106.00 (C-1), 105.38 (C-1), 100.97 (C-1$^{\beta\text{-Ara}f}$ ×4), 100.61 (C-1$^{\beta\text{-Ara}f}$ ×4), 86.92, 86.08, 84.46, 84.12, 84.05, 83.91, 83.85, 83.70, 83.56, 83.33, 83.25, 83.14, 83.06, 82.96, 82.77, 82.47, 82.42, 82.23, 82.18, 82.08, 82.04, 82.02, 81.98, 81.91, 81.82, 81.79, 81.73, 81.70, 81.59, 81.48, 81.26, 80.17, 79.25, 79.21, 77.89, 77.80, 75.91, 74.23, 73.98, 73.93, 73.90, 73.83, 73.75, 73.69, 73.49, 73.42, 73.38, 73.33, 73.17, 73.13, 73.04, 72.90, 73.82, 72.72, 72.67, 72.48, 72.38, 72.36, 72.27, 72.16, 72.07, 71.91, 71.81, 71.78, 71.61, 71.56, 71.05, 69.93, 69.83, 69.55, 67.63 (octyl OCH_2), 66.55, 66.46, 65.82, 65.80, 65.72, 65.70, 65.56, 65.49, 65.41, 65.31, 65.26, 65.16, 65.13, 65.10, 64.93, 31.98 (octyl CH_2), 29.58 (octyl CH_2), 29.55 (octyl $OCH_2\underline{C}H_2$), 29.39 (octyl CH_2), 26.23 (octyl CH_2), 22.80 (octyl CH_2), 14.25 (octyl CH_3); MALDI-TOF MS Calcd for $C_{2003}H_{1918}O_{492}Na$ $[M + Na]^+$ *m/z*: 33885.4, found: 33884.7.

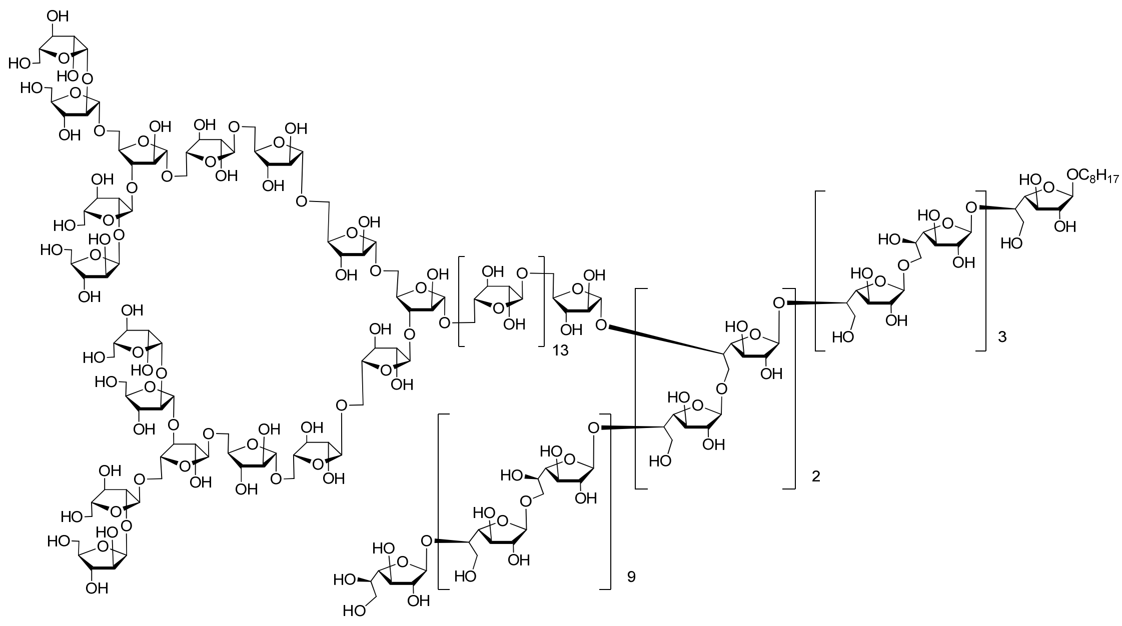

Deprotected 92mer (1): The global deprotection was started with 45.0 mg of fully protected **35**, which was divided into 3 portions (15.0 mg each portion) to carry out 3 individual reactions. To a solution of **35** (15.0 mg, 0.44 μmol) in THF (3 mL) and CH_3OH (6 mL) was added CH_3ONa (5.0 M in CH_3OH) to adjust pH to ~10, the reaction mixture was stirred for 24 h at room temperature, and neutralized by the addition of Dowex®50WX4 ion-exchange resin. The solution was filtered and concentrated. Purification by size exclusion chromatography (Bio-Beads S-X1, ethyl acetate as eluent) gave a residue, which was dissolved in a mixture of EtOAc/THF/1-PrOH/H_2O (4 mL, 2:1:1:1) with Pd/C (100 mg, 10% Pd content). The resulting solution was stirred under an atmosphere of hydrogen (0.4 MPa) for 80 h at room temperature, filtered through Celite, and concentrated to give a crude product. This global deprotection process was repeated twice, and the combined crude products were purified by gel filtration (Sephadex LH-20, CH_3OH/H_2O, 1:1) to afford **1** (13.1 mg, 75% for two steps) as glassy solid. ^1H NMR (600 MHz, D_2O) δ 5.24 – 5.22 (m, 18H), 5.21 (d, J = 2.0 Hz, 1H), 5.17 – 5.16 (m, 6H), 5.139 (d, J = 4.6 Hz, 4H, H-$1^{\beta\text{-Ara}f}$), 5.136 (d, J = 4.5 Hz, 4H, H-$1^{\beta\text{-Ara}f}$), 5.11 (br s, 6H), 5.08 – 5.07 (m, 38H), 5.01 – 5.00 (m, 14H), 4.96 (d, J = 2.1 Hz, 1H), 4.30 – 3.55 (m, 492H), 1.62 – 1.58 (m, 2H), 1.30 – 1.22 (m, 10H), 0.86 (t, J = 6.9 Hz, 1H). ^{13}C NMR (600 MHz, D_2O) δ 108.70, 108.63, 108.44, 108.35, 108.25, 107.94, 107.91, 107.82, 106.48, 106.33, 101.59 (C-$1^{\beta\text{-Ara}f}$ ×4), 101.46 (C-$1^{\beta\text{-Ara}f}$ ×4), 87.92, 87.64, 83.83, 83.72, 83.66, 83.42,

83.34, 83.18, 83.03, 82.83, 82.73, 82.68, 82.59, 82.49, 82.25, 82.14, 82.07, 82.04, 81.97, 81.93, 81.89, 81.84, 81.69, 81.64, 79.94, 79.86, 77.55, 77.50, 77.45, 77.40, 77.32, 77.27, 77.09, 76.78, 76.66, 76.49, 75.66, 75.57, 74.99, 74.94, 71.34, 70.41, 70.34, 70.11, 69.48, 67.70, 67.63, 67.54, 67.44, 67.24, 67.10, 67.03, 63.80, 63.76, 63.63, 61.94, 61.84, 61.42, 61.40, 31.95 (octyl CH_2), 29.50 (octyl CH_2), 29.26 (octyl CH_2), 29.24 (octyl CH_2), 26.05 (octyl CH_2), 22.88 (octyl CH_2), 14.31 (octyl CH_3). To improve the mass detection sensitivity, the per-acetylation of compound **1** was performed[5]. To a solution of **1** (0.5 mg) in dry pyridine (1 mL) was added Ac_2O (0.5 mL) and DMAP (0.1 mg), the resulting mixture was stirred at 80 ^oC for 2 h. After removal of the solvent, the residue was dissolved in EtOAc (5 mL), washed with 0.1 N HCl (3 mL), saturated aqueous $NaHCO_3$ solution (3 mL) and brine (3 mL), dried over Na_2SO_4, filtered, and concentrated to give a crude product, which was directly used for mass detection. MALDI-TOF MS of per-acetylated derivative of **1**: Calcd for $C_{928}H_{1244}O_{614}Na$ $[M + Na]^+$ *m/z*: 22246.4, found 22240.5.

Supplementary references

1. Zhu, S.-Y. & Yang, J.-S. Synthesis of tetra- and hexasaccharide fragments corresponding to the *O*-antigenic polysaccharide of *Klebsiella pneumoniae*. *Tetrahedron* **68**, 3795–3802 (2012).
2. Callam, C. S. & Lowary, T. L. Synthesis of methyl 2,3,5-tri-*O*-benzoyl-*α*-D-arabinofuranoside in the organic laboratory. *J. Chem. Educ*. **78**, 73–74 (2001).
3. Ishiwata, A., Akao, H. & Ito, Y. Stereoselective synthesis of a fragment of mycobacterial arabinan. *Org. Lett*. **8**, 5525–5528 (2006).
4. Schmidt, R. R. & Toepfer, A. Glycosylation with highly reactive glycosyl donors: efficiency of the inverse procedure. *Tetrahedron Lett*. **32**, 3353–3356 (1991).
5. Bhamidi, S. *et al*. Detailed structural and quantitative analysis reveals the spatial organization of the cell walls of *in Vivo* grown *Mycobacterium leprae* and *in Vitro* grown *Mycobacterium tuberculosis*. *J. Biol. Chem*. **286**, 23168–23177 (2011).
